# Synthesis, antibacterial evaluation and in silico studies of novel 2-(benzo[*d*]thiazol-2-yl)-*N*-arylacetamides and their derivatives as potential DHFR inhibitors

**DOI:** 10.1186/s13065-025-01386-5

**Published:** 2025-01-31

**Authors:** Nadia Hanafy Metwally, Galal Hamza Elgemeie, Aya Ragab Abdelrazek, Salwa Magdy Eldaly

**Affiliations:** 1 Chemistry Department, Faculty of Science, Cairo University, Giza, 12613 Egypt; 2 Chemistry Department, Faculty of Science, Helwan University, Cairo, Egypt

**Keywords:** Synthesis, ADME studies, Benzo[*d*]thiazole, Coumarins, *N*-arylpyridone, DFT, Antibacterial, Dihydrofolate reductase

## Abstract

Novel *N*-arylacetamides **2a**–**f** were synthesized based on benzo[*d*]thiazole scaffold. The compounds **2a**–**c** underwent Knoevenagel condensation through green synthetic method with different aromatic aldehydes and pyrazole-7-carbaldehydes delivered the respective arylidenes with efficient yields. Arylidenes **4** reacted with malononitrile affording the corresponding *N*-arylpyridones **11a**–**i**. Moreover, the reaction of **2a**–**c** with each of salicylaldehyde and 5-arylazo salicylaldehydes afforded the unexpected coumarins rather than quinolin-5-ones. The structure of coumarin **8** was confirmed by density functional theory (DFT) calculations using basis set B3LYP/6-311 G +  + (d,p) to obtain the suitable geometrical structure with molecular orbitals` energies revealing its planar structure and its agreement with experimental data. Besides, the antibacterial activity was tested against different bacterial strains revealing potent activity especially Gram-negative bacteria with excellent minimum inhibition concentration (MIC) value ranging from 31.25 to 250 µg/L. Additionally, compounds **2c** and **4m** showed enzyme inhibition against dihydrofolate reductase in *Escherichia coli* with greater potency (IC_50_ for **2c** = 3.796 µM, IC_50_ for **4m** = 2.442 µM) than the standard antibiotic trimethoprim (IC_50_ = 8.706 µM). Investigation of the physicochemical properties of the newly compounds exhibited their better ADME properties that can be developed for the discovery of new antibacterial agents.

**Graphical Abstract:**

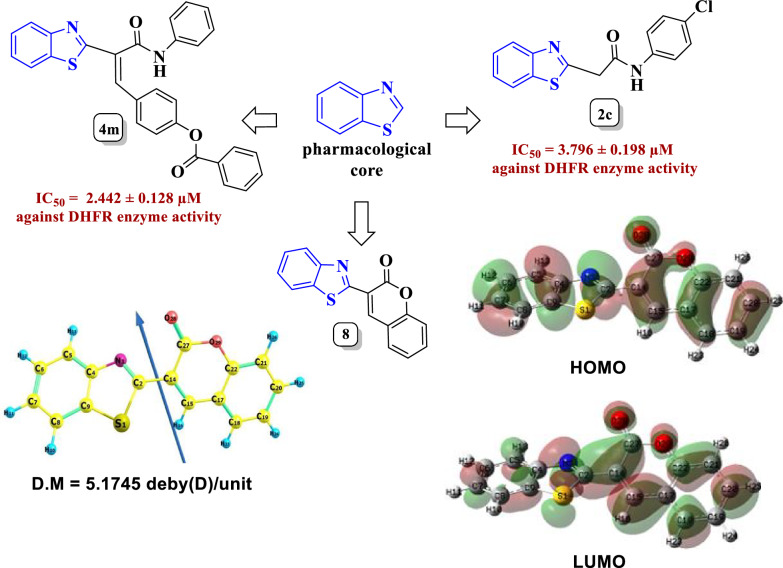

**Supplementary Information:**

The online version contains supplementary material available at 10.1186/s13065-025-01386-5.

## Introduction

Infectious diseases are major cause in many fatalities. Antimicrobial resistance is one of the largest risks to contemporary global health, and it is becoming more and more common. Bacterial resistance reduces the efficiency of antibiotics and increases the death rates associated with bacterial illnesses [1]. This problem came to the top of the list of global public health issues [2]. There were about 5 million death cases due to bacterial resistance in 2019 [3]. The bacterial strains: *E*. *coli*, *K*. *pneumonia*, *A. baumannii, P. aeruginosa*, *St*. *aureus* and *St. pneumonia* were major contributors for deaths associated with resistance, so that a major fight to combat the bacterial infections caused by these strains is the highest priority of modern medicines and many researches came up with new compounds of antibacterial activity against these strains [4–7]. In recent years, numerous nations have engaged in the fight against the microbial diseases that are now the most serious health issues [8]. These global trends contribute to develop new chemical structures with strong antibacterial capabilities, which could result in innovative and potent pharmaceutical formulations.

**Fig. 1:**
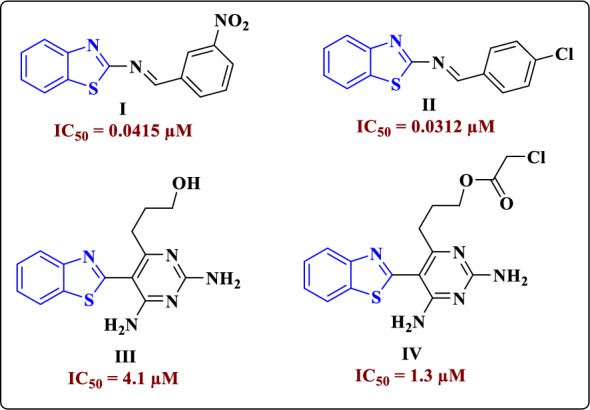
Benzothiazole derivatives of DHFR enzyme inhibition activity

DHFR is an enzyme that controls converting dihydrofolate to THF, which is a critical step in the synthetic pathway of DNA, RNA, and proteins. Also, THF takes responsibility in DNA methylation, which is necessary for the activity of folate-dependent enzymes. Additionally, DHFR is required for the intracellular conversion of synthetic folic acid, which can be found in fortified foods and supplements-into THF can be involved in the metabolism of folate and homocysteine. In *vivo*, DHFR inhibition suppresses cell growth and proliferation by preventing thymidine production and hence deactivating DNA synthesis. Therefore, targeting DHFR is an important aim for drug design and development [9]. As a result, drugs are designed to target DHFR, including benzo[*d*]thiazoles. Thakkar et al. [10] have developed *N*-(benzo[*d*]thiazol-2-yl)-1-(4-aryl) methenamine **I** and **II**, which showed effective DHFR inhibition with IC_50_ = 0.0415 and 0.0312 µM, respectively, besides being excellent antimalarial agents (Fig. 1). Moreover, benzo[*d*]thiazoles-incorporating into pyrimidine moiety as **III** and **IV**, exhibited IC_50_ against DHFR activity equals 4.1and 1.3 µM, respectively [11]. A recent report showed that certain benzothiazoles can form a more effective binding relationship with *P. aeruginosa* and *E. coli* dihydrofolate reductase's active sites through docking study [12].

**Fig. 2:**
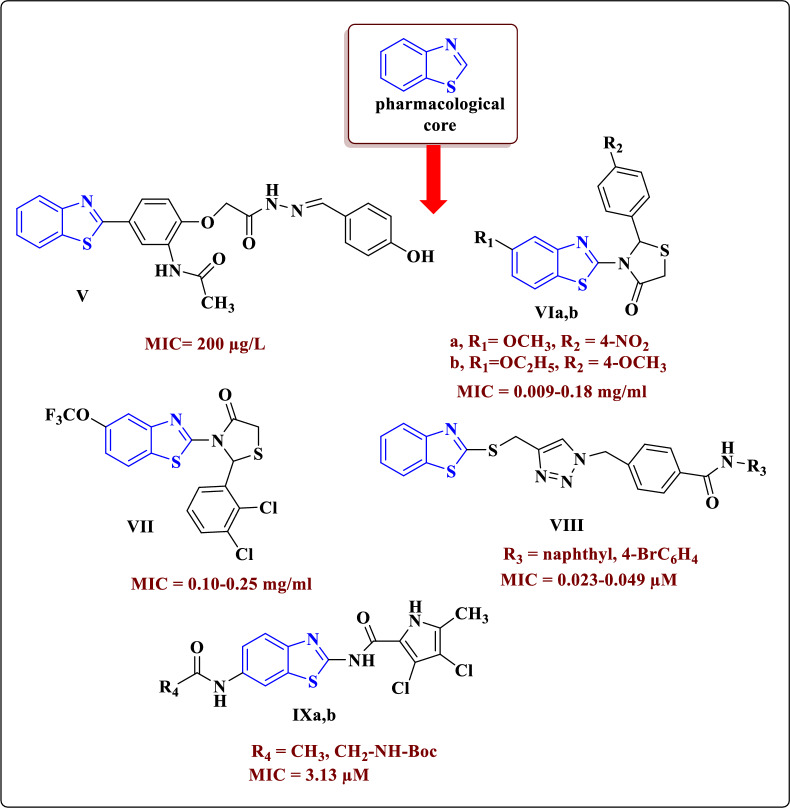
Structure of antibacterial active compounds comprising benzothiazole scaffold

Medicinal chemistry researchers are taking consideration about benzothiazoles because of their diverse and valuable range of biological activities including antimicrobial [13], anticancer [14], antidiabetic [15], anti-convulsant [16], antibacterial and antiviral activities [17]. The benzothiazole moiety was found in several other distinct chemical templates and moieties with antibacterial activity. Taking for an example hydrazone-incorporating benzothiazole **V** showed antibacterial activity against *E*. *coli* and *P. aeruginosa* with effective MIC values = 200 µg/L (Fig. 2) [18]. Tratrat et al. have reported the synthesis of thiazolidine-4-one derivatives of benzothiazole **VIa**,**b** as antibacterial agents with MIC value of 0.009–0.18 mg/ml in *E*. *coli* and *P. aeruginosa* [19]. Kaushik et al. have synthesized 1,2,3-triazoles containing benzothiazole moiety, which were assessed for their ability to combat the bacterial strains: *St*. *aureus*, *B*. *subtilis*,* K*. *pneumonia* and* E*. *coli*. They were reported of excellent antibacterial activity among those compounds the triazole-benzothiazoles **VII**, that showed to be potent with better MIC values: ~ 0.023–0.049 µg/L [20]. Moreover, thiazolidinone derivative of 2-aryl-3-(6-trifluoromethoxy)benzothiazole **VIII** revealed MIC value equals 0.023–0.049 µg/L which is more effective than the used standard against* E*. *coli, P. aeruginosa* and *St*. *aureus* [21]. Similarly, Skok et al. examined the newly synthesized benzothiazole derivatives **IXa**,**b** for being antibacterial agents showing effective MIC value (3.13 µg/L) against *E. faecalis* [22]. Numerous studies have found that benzothiazole derivatives with bacterial growth inhibition capability can also be attributed to their capability of interacting with a variety of cellular targets, especially enzymes such as DNA gyrase [23–25], dihydropteroate synthase [26, 27], dihydrofolate reductase [10–12], dihydroorotase [28], peptide deformylase [29] and aldose reductase [30].

**Fig. 3:**
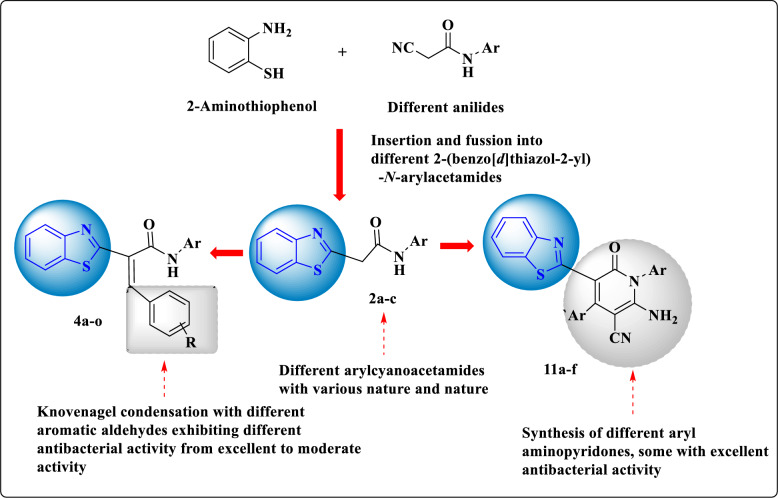
Synthetic strategy of **2a**–**c**, **4a**–**r, 6**, **8a**–**c** and **11a**–**f** comprising benzo[*d*]thiazole core **2**

According to the aforementioned facts and in keeping up with our work in synthesis of new compounds of biological activities with potential activity in protein and enzyme inhibition [31–45], herein we report the novel synthesis of 2-(benzo[*d*]thiazol-2-yl)-*N*-arylacetamides **2a**–**f** based on benzothiazole scaffold. Our design strategy depended on the contribution and blending of the benzothiazole core in the newly synthesized acetamide derivatives as a biological active scaffold with good pharmaceutical background then we introduced different substituents with variety of electronic properties and features by reaction with different chemical reagents. The starting **2a**–**c** reacted with different aromatic and heteryl aldehydes affording the respective aryl methylene derivatives through conventional method. Also, green synthetic protocol was operated for the synthesis of the aryl methylene derivative using *p*-tolyl sulphonic acid as a catalyst through a grinding method. Also, the unexpected coumarins were obtained from the reaction of **2a**–**c** with substituted salicylaldehydes rather than quinolines, the expected products. The structure and formation of **8** were proved by chemical techniques with spectral tools [IR and ^1^H NMR] and DFT using basis set B3LYP/6-311 +  + G (d, p) level of calculation. Aryl methylenes **4** reacted with malononitrile to afford *N*-aryl pyridones. The majority of the newly produced compounds were tested for their effectiveness as antibacterial agents against four strains of Gram-negative bacteria and two strains of Gram-positive bacteria and they revealed moderate to strong antibacterial activity against most of the strains. The MIC for the strongest antibacterial candidates was evaluated revealing values = 31.25–250 µg/L. then, the most antibacterial active compounds **2**, **4m** and **11c** were tested for DHFR inhibition activity showing effective potency for **2c** and **4m** than that of the standard antibiotic trimethoprim. Finally, the pharmaceutical properties and health and toxic hazards were detected using Osiris Methodology and Swiss ADME predictions displaying good and better profile for most of the newly synthesized compounds and the high possibility for **2c**, **4m** and **11c** to be as drug potential candidates (Fig. 3).

## Results and discussion

### Chemistry

**Scheme 1:**
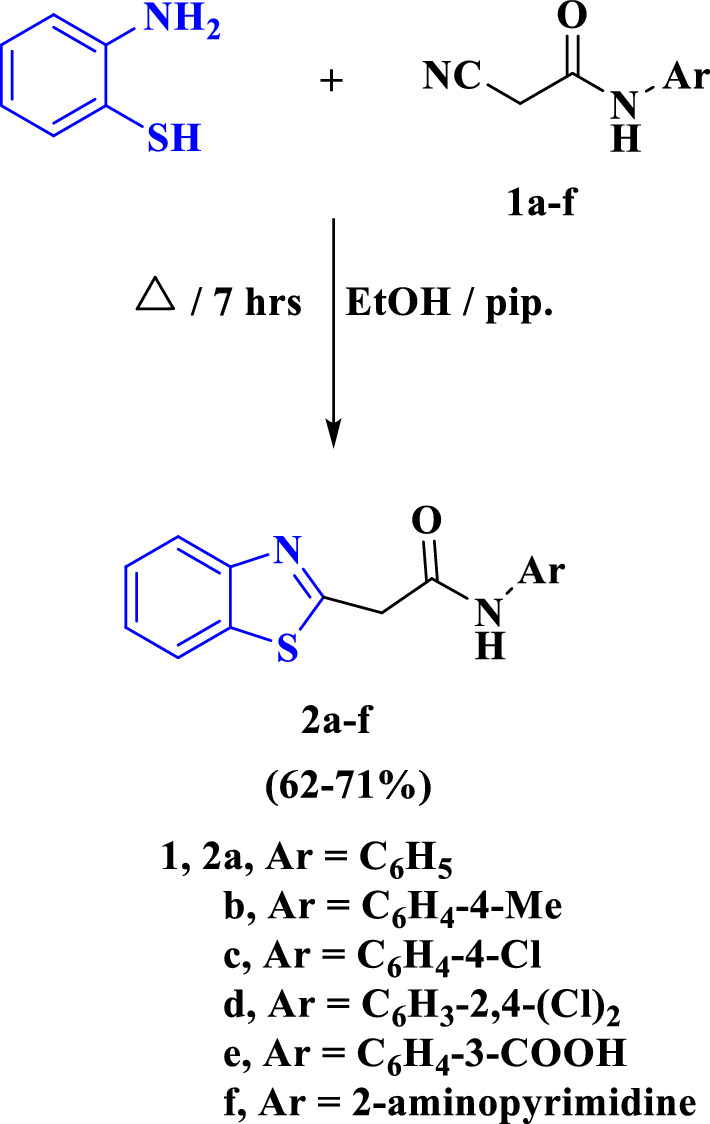
Explains the synthesis of benzo[*d*]thiazole-acetamides **2a**–**f**

A series of some new 2-(benzo[*d*]thiazol-2-yl)-*N*-arylacetamides **2a**–**f** were prepared through the reaction of 2-aminothiophenol with 2-cyano-*N*-arylacetamides **1a**–**f** (Scheme 1). The structures of the prepared compounds **2a**–**f** were proved through spectral data techniques. The IR spectra of the compounds **2a**–**f** showed the disappearance of an absorption band at ν_max_ ~ 2200–2300 cm^−1^ for the cyano group confirming the in situ heterocyclization between 2-aminothiophenol and compounds **1a**–**f**, besides the ^1^H NMR spectra of **2a**–**f** revealed a singlet signal between the region δ ~ 10.44–10.78 ppm attributed to NH`s proton and a characteristic singlet signal for CH_2_`s proton between the region δ ~ 3.78–4.14 ppm. As an example, the IR spectrum of **2a** revealed absorption bands at ν_max_ = 3448 and 1657 cm^−1^ for the imino and carbonyl functions, respectively. The ^1^H NMR spectrum of **2a** displayed a singlet signal at δ = 4.29 ppm for methylene protons besides one doublet signal at δ = 7.61ppm with *J* coupling constant equals 7.5 Hz assigned for phenyl proton. In addition, multiplet signals at δ = 7.07–7.10, 7.30–7.33, 7.39–7.42 and 7.49–7.52 ppm for aryl protons and a singlet signal at δ = 10.43 ppm for NH`s proton. Also, the ^1^H NMR spectrum of **2a** showed two multiple signals at δ = 7.96–7.98 and 8.05–8.08 ppm for benzothiazole moiety protons. The ^13^C NMR of **2a** revealed a signal at δ = 41.7 ppm for CH_2_`s carbon besides signals at δ = 165 and 166.1 ppm for carbonyl carbons and S-C = N, in addition to a signal at δ = 152.3 ppm for aryl carbon of phenyl moiety. Furthermore, the ^13^C NMR of **2a** displayed characteristic signals at δ = 119.3, 122.0, 122.3, 123.7, 125.0, 126.1, 128.9, 135.3, 138.8 ppm for aryl carbons. The Mass spectrum of **2a** revealed a molecular ion peak at m/z = 268 (M^+^, 65.3%) compatible with the molecular formula C_15_H_12_N_2_OS and a base peak at m/z = 176 (100%) besides other expected peaks (Scheme 1).

**Scheme 2:**
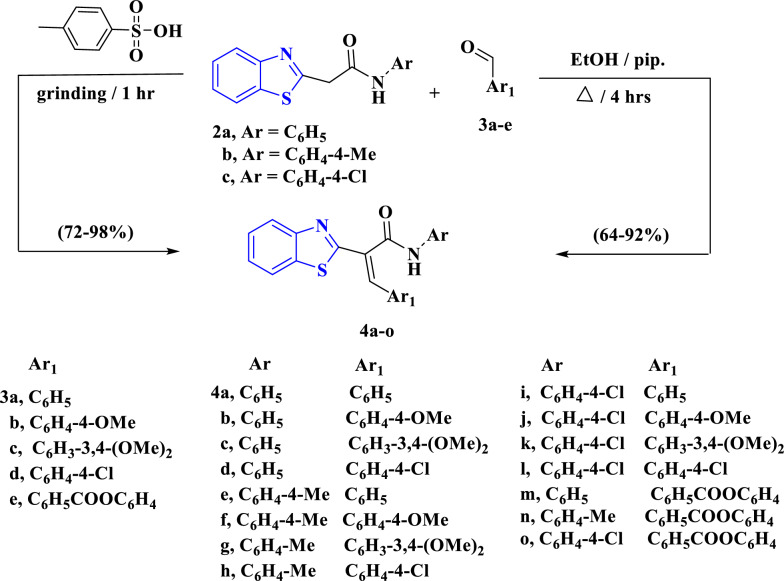
Describes the Knӧvenagel condensation of **2a**–**c** with different aromatic aldehydes **3a**–**f** afforded the corresponding arylidenes **4a**–**o**

Next, we underwent an equimolar mixture of **2a**–**c** with aromatic aldehydes **3a**–**e** under the Knӧvenagel condensation delivering the respective **4a**–**o** (Scheme 2). The spectral characterization of products **4a**–**o** were performed by considering their IR, ^1^H NMR, ^13^C NMR and MS spectra techniques. The ^1^H NMR spectra of the prepared series showed the disappearance of a singlet signal at δ ~ 3.78–4.14 ppm attributed to CH_2_`s proton that emphasizes the Knӧvenagel condensation. The IR spectrum of **4a**, as an example, showed the presence of absorption bands at ν_max_ = 3234 and 1649 cm^−1^ for imino (NH) and carbonyl (CO) functions, respectively. The ^1^H NMR spectrum of **4a** revealed two doublet signals at δ = 8.01 and 8.10 ppm with *J* coupling constants equal 7.8 and 8.1 Hz, respectively, assignable to benzothiazole moiety protons. Its ^1^H NMR of **4a** also displayed a singlet signal at δ = 10.76 ppm attributed to NH proton in addition to other expected multiplet signals at δ = 7.10–7.15, 7.30–7.41, 7.48–7.55 and 7.69–7.71 ppm for aryl protons. The ^13^C NMR spectrum of **4a** revealed characteristic signals at δ = 166.0, 164.7, 153.1, 138.8 and 134.1 ppm for the carbonyl carbon, S–C=N, carbon of benzothiazole moiety, vinylic carbon and carbon of phenyl moeity, respectively. Additionally, the ^13^C NMR spectrum showed signals for aryl carbons at δ = 119.6, 122.2, 122.8, 124.0, 125.8, 126.7, 128.8, 128.9, 129.2, 129.7, 132.5 and 133.9 ppm. Alternatively, the arylidenes **4a**–**o** were obtained through the reaction of **2a**–**c** with the aromatic aldehydes **3a**–**e** in the presence of *p*-tolyl sulfonic acid as a catalyst through the grinding method for 1 h. The formed products had the same physical and spectral aspects (Scheme 2).

**Scheme 3:**
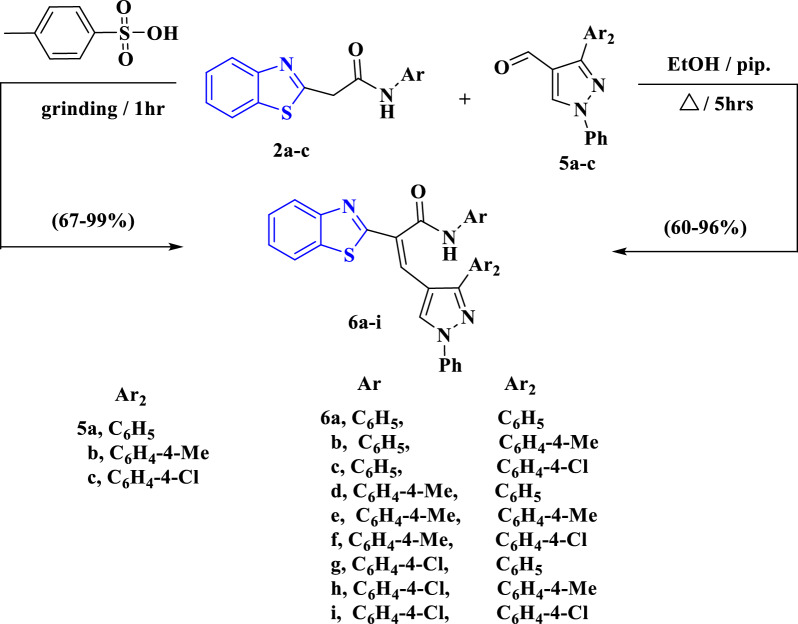
The reaction of **2a**–**c** with pyrazole-2-carbaldehydes **5a**–**c** to afford the corresponding products **6a**–**i** through both thermal and green methods

Similarly, compounds **2a**–**c** condensed with pyrazole-2-carbaldehydes **5a**–**c** to furnish the corresponding arylidene derivatives **6a**–**i** (Scheme 3). Then, a green synthetic approach was operated to obtain the compounds **6a**–**i** with the aid of *p*-tolyl sulfonic acid as a catalyst through a grinding method for 1 h. The IR spectrum of **6a** displayed expected absorption bands at ν_max_ = 3436 and 1670 cm^−1^ for imino (NH) and carbonyl (CO) functions, successively. Its ^1^H NMR spectrum showed two doublet signals at δ = 7.99 and 8.08 ppm with *J* coupling constants equal 8.1 and 7.5 Hz, respectively, for benzothiazole moiety protons. Also, the ^1^H NMR spectrum showed multiple bands at δ = 7.16–7.18 and 7.37–7.78 ppm referring to aryl protons besides a singlet signal at δ = 10.81 ppm for NH`s proton, in addition to two singlet signals at δ = 7.52 and 8.41 ppm for vinylic proton (=CH) and pyrazole`s proton. The ^13^C NMR spectrum of **6a** revealed characteristic signals for aryl and vinylic protons at δ = 115.5, 118.7, 119.5, 122.1, 122.7, 124.1, 124.4, 125.6, 126.7, 127.1, 127.3, 128.6, 128.9, 129.0, 129.7, 131.4, 131.6, 133.9, 138.7 ppm, besides, it showed signals at δ = 138.8, 153.0, 153.1, 164.7 and 165.3 ppm attributed to vinylic, pyrazole, benzothiazole, S–C=N and carbonyl carbons, respectively. The mass spectrum of **6a** showed a molecular ion peak at m/z = 498 (M^+^, 32.5%) attributed to the molecular formula C_31_H_22_N_4_OS besides other expected peaks, also the mass spectrum showed two base peaks at m/z = 91.0 and 188.4 (100%) (Scheme 3).

**Scheme 4:**
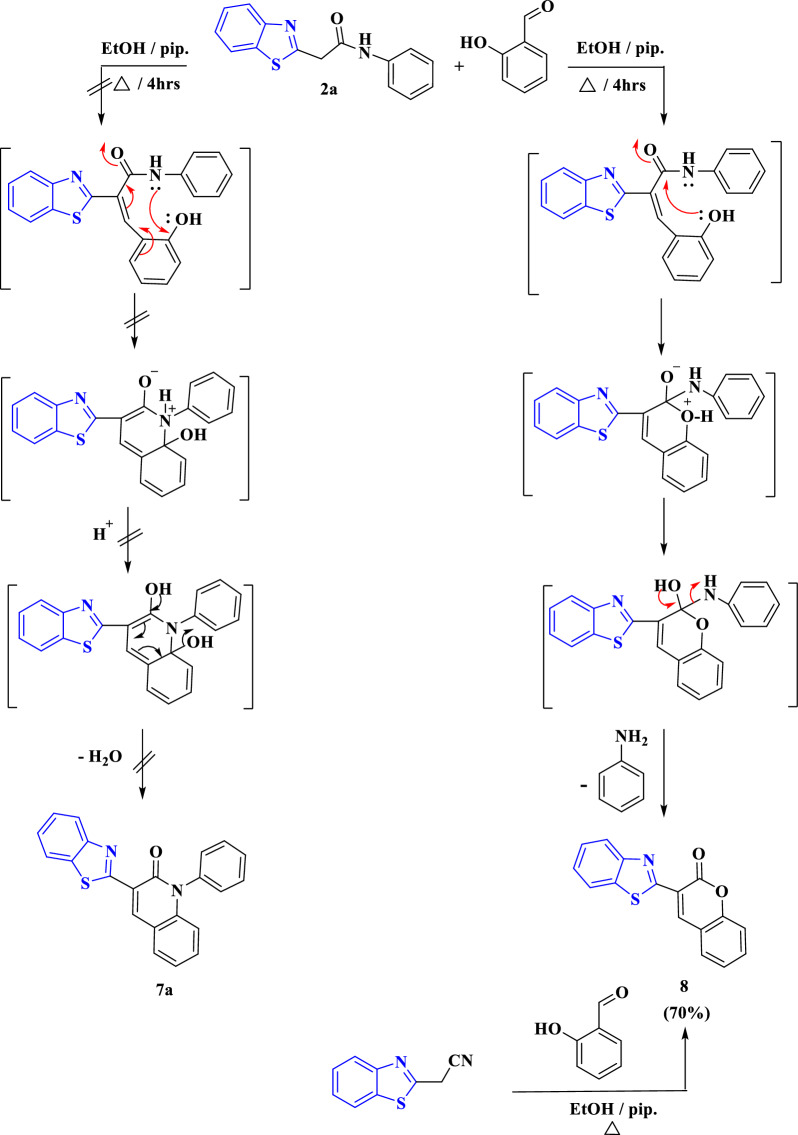
The suggested formation of 3-(benzo[*d*]thiazol-2-yl)-1-phenylquinolin-2(1*H*)-one **7a** and 3-(benzo[*d*]thiazol-2-yl)-2*H*-chromen-2-one **8**

**Scheme 5:**
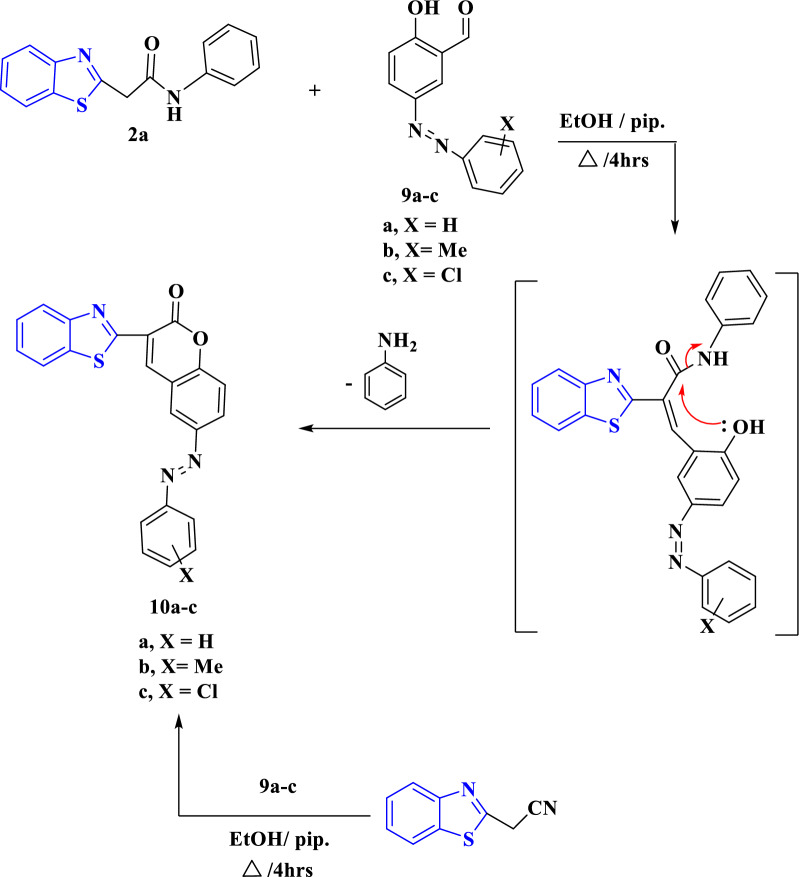
Pathway for the formation of arylazo coumarins **10a**–**c**

Furthermore, compounds **2a**–**c** reacted with salicylaldehyde in ethanol in the presence of piperidine to obtain the same product **8** for **2a**–**c**, as identified from its physical properties as melting and mixed melting points and IR spectra (Scheme 4). From the reaction equation shown in Scheme 4, coumarin derivative instead of the expect products 1-arylquinolines **7a**–**c** was obtained. The proposed mechanism for the formation of compounds **7a** and **8** is shown in Scheme 4. It was suggested that the formation of **8** progressed by Knӧvenagel condensation of compounds **2a**–**c** with salicylaldehyde with removal of water molecule, followed by nucleophilic addition of oxygen atom on the carbonyl moiety with elimination of aniline molecule as presented in Scheme 4. Compound **8** [46] can be obtained alternatively through the reaction of 2-cyanomethylbenzo[*d*]thiazole with salicylaldehyde under the previous conditions (Scheme 4). Similarly, the reaction of **2a**–**c** with 5-arylazosalicylaldehydes **9a**–**c** under the previous conditions afforded the arylazo coumarins **10a**–**c** (Scheme 5) [47]. Spectroscopic analyses were used to validate the structure of **10a**–**c** (Scheme 5). The structure of **10b** was emphasized by an absorption band at 1727 cm^−1^ in its IR spectrum for carbonyl group. As well as, its ^1^H NMR chart showed a singlet signal at chemical shift equals 2.43 ppm for methyl protons, in addition to multiplet signals corresponding to aromatic protons ranging from 7.63 to 7.79 ppm related to aryl protons. Also, its ^1^H NMR revealed two doublet signals at δ equal 7.38 and 7.89 ppm for aromatic protons with *J* coupling constant equal 8.1 and 7.5 Hz, respectively. The benzothiazole protons appeared in the ^1^H NMR chart as multiplet signals at δ = 8.02–8.04 and 8.09–8.21 ppm.

**Fig. 4:**
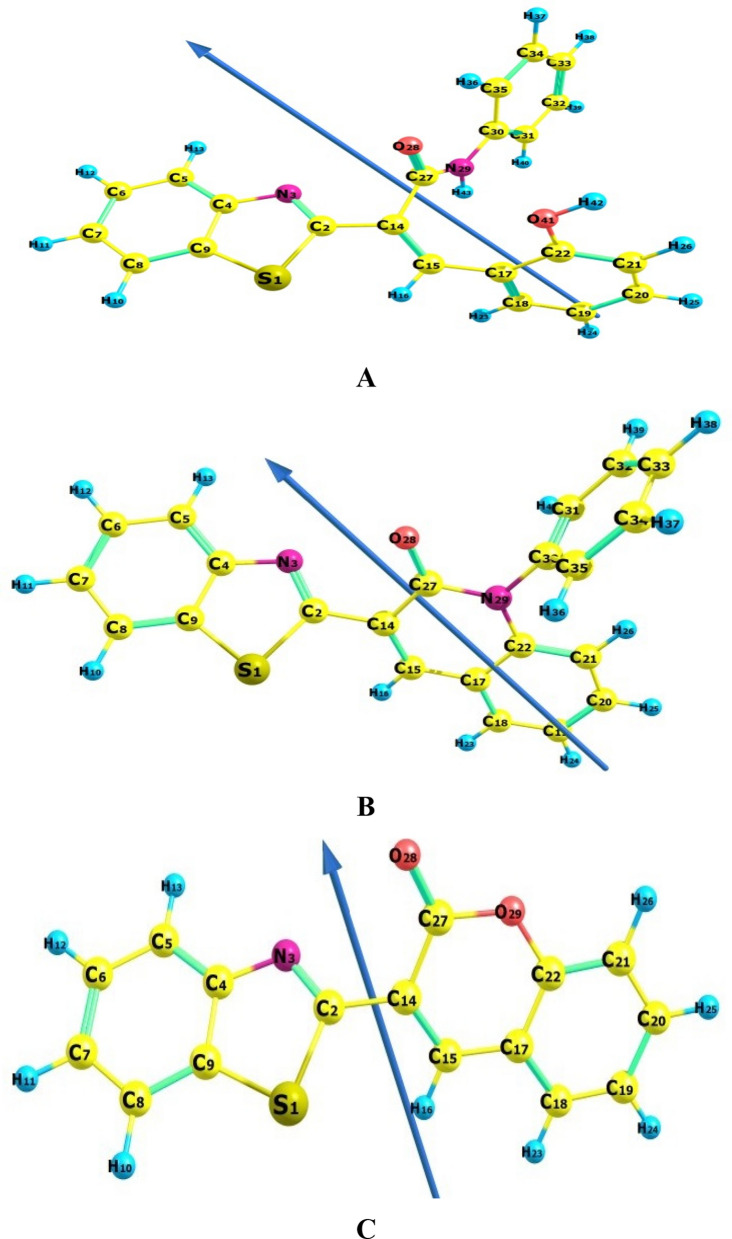
The optimized geometry, numbering system, vector of dipole moment of the newly synthesized compounds (**2a** (**A**), **7a** (**B**) and** 8** (**C**)) using B3LYP/6-311 +  + G (d, p) level of calculation

The formation of compounds **7a** and **8** was also theoretically confirmed by DFT. So, during this study, *Gaussian 09W* software package [48] was used to run the molecular modeling calculations of potential target derivatives (**2a**, 7**a**, and 8) using the DFT study and the basis set B3LYP set 6-311 +  + G (d, p) [49–53]. The molecular structures of target derivatives (**2a, 7a,** and 8) were geometrically optimized and their D. M. are detailed in Table 1. During the geometry optimization, no symmetry constrains were applied [54, 55]. The same level of theory has been applied to compute vibrational frequencies for each compound, and the molecular structure of target compounds were found correspond to real minima of the potential energy surface. GaussView (v6.1) [56] and ChemCraft (v1.6) package [57] were used to visualize the optimized structure and molecular orbitals revealing the value of HOMO` and LUMO`s energies for **2a**, **7a** and **8** (Table 2). From optimized geometry data using DFT, it clarified that the 3-(benzo[*d*]thiazol-2-yl)-2*H*-chromen-2-one **8** has planner structure which was computable to experimental and the deviation of dipole moment indicates charge flow in case of electronic transition, while the structure of starting compound **2a** wasn’t planner (Fig. 4).

**Table 1:** The D.M. of the newly synthesized compounds **2a**, **7a** and** 8**

Compd No.	D. M
**2a**	4.3368
**7a**	3.7572
**8**	5.1745
Water	2.1591
Ph-NH_2_	1.5908

**Table 2:** The optimized energy of the newly synthesized compounds **2a**, **7a** and** 8** together with relative energies for reaction using B3LYP/6-311 +  + G (d, p) level of calculation

	Total energy, H		Total energy, H	Relative energy, H	Relative energy, kcal/mol
**2a**	− 1506.444106	Reactant, **2a**	− 1506.444106		
**7a**	− 1430.001816	**7a** + water	− 1506.460347	− 0.016240671	− 10.19118352
**8**	− 1218.762542	**8** + Ph-NH_2_	− 1506.450273	− 0.006166797	− 3.869726786
Water	− 76.45853077				
Ph-NH_2_	− 287.6877311

**Fig. 5:**
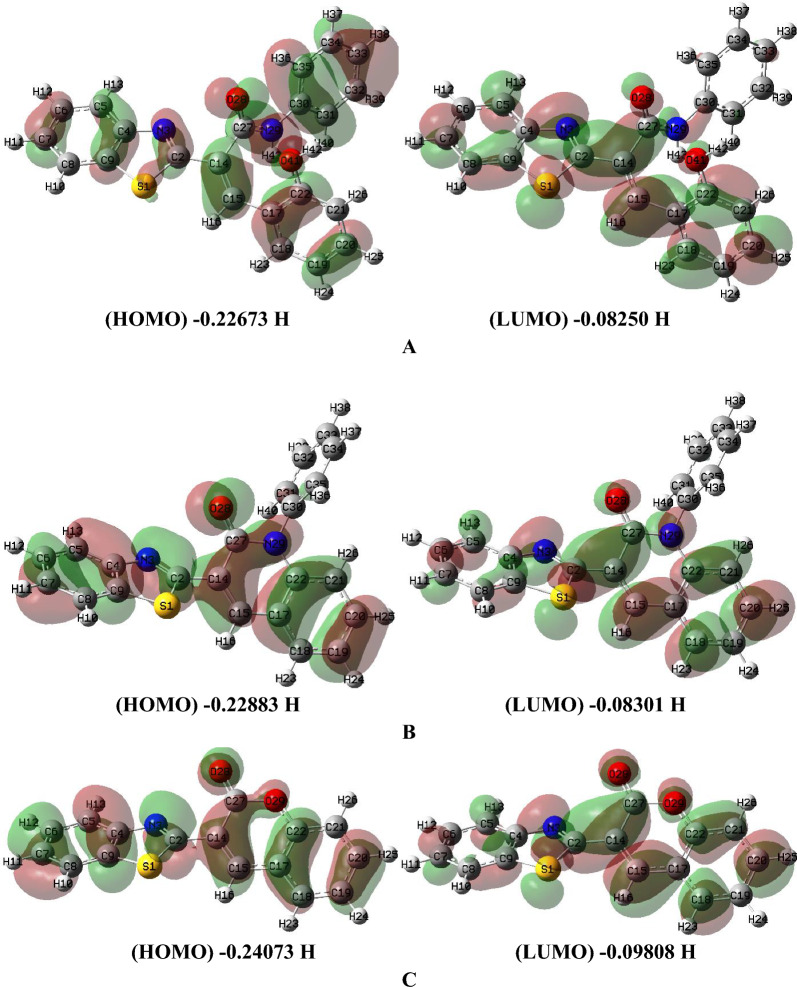
Frontier molecular orbitals and Energy (Hartree) of the newly synthesized compounds **2a** (**A**), **7a** (**B**) and** 8** (**C**) using B3LYP/6-311 +  + G (d, p) level of calculation

Accordingly, it was concluded that B3LYP in predicting bond lengths and angles is the most satisfactory functional with preferred time CPU. In particular, B3LYP was found to be the most adequate functional with respect to computational time and power uses. Therefore, B3LYP was selected for geometry optimizations and all calculations for ground state rather than using transition state searching. Form the frontier molecular orbitals surfaces and energies combined with experimental spectra reported in the work the coumarin derivative is best pathway rather than other pathways as shown in Fig. 5. Also, the formation of compound **8** was confirmed alternatively by the reaction of 2-cyanomethyl-benzothiazole with salicylaldehyde under the same reaction conditions to afford product identical in all respective of **8** (m.p, mixed m.p and IR spectrum) as demonstrated in Scheme 4.

**Scheme 6:**
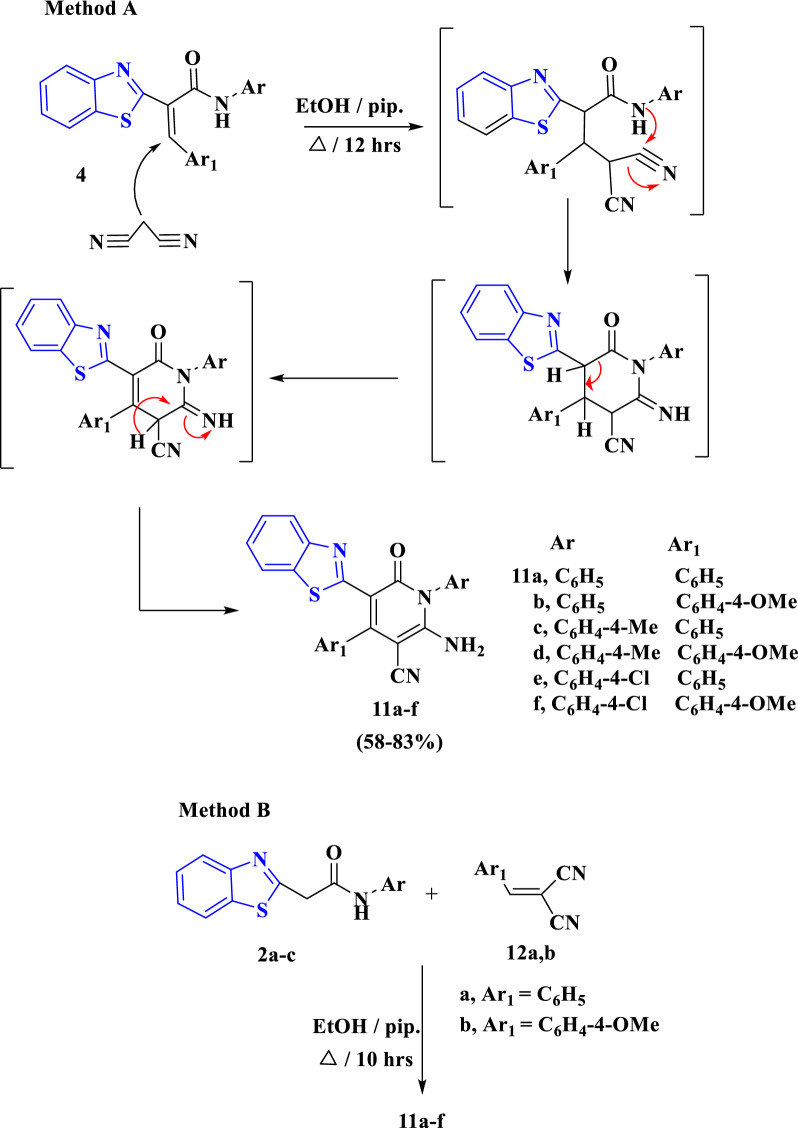
The pathway for formation of *N*-aryl aminopyridones **11a**–**f** through method A and B

Finally, we performed synthetic pathway for the preparation of *N*-arylpyridone derivatives **11a**–**f** through the reaction of 2-(benzo[*d*]thiazol-2-yl)-*N*-phenylacetamides **2a**–**c** with malononitrile in ethanol in the presence of piperidine under reflux for 12 h (Scheme 6). The formation of **11** was suggested to proceed firstly by the *Micheal* addition step of the methylene group of malononitrile, followed by the nucleophilic addition of NH to cyano group. Finally, heterocyclization and aromatization. Taken **11a**, as a representative example, its IR spectrum displayed a broad band at ν_max_ = 3448–3302 cm^−1^ attributed for the amino group besides two sharp bands at ν_max_ = 2206 and 1643 cm^−1^ for CN and CO functions, respectively. The ^1^H NMR spectrum of **11a** revealed two doublet signals at δ = 7.37 and 7.43 ppm for aryl protons with *J* coupling constants equal 8.0 and 7.5 Hz, respectively, besides two doublet signals at chemical shift equals 7.57 and 7.86 ppm assignable for benzothiazole protons with *J* coupling constant equals 7.5 Hz. In addition to multiplet signals appeared at δ = 7.21–7.28 ppm for aryl and amino protons with other multiple signals at δ = 7.33–7.34 and 7.52–7.55 ppm for aryl protons. The Mass spectrum of **11a** revealed a molecular ion peak at m/z = 420 (M^+^, 77.9%), which was compatible with the molecular formula C_25_H_16_N_4_OS and a base peak at m/z = 89 (100%) besides other expected signals. Alternatively, compounds **11a**–**f** were obtained through the reaction of anilides **2a**–**c** with arylmethylene malononitrile derivatives **12a**,**b** in absolute ethanol in the presence of a catalytic amount of piperidine for 10 h (Scheme 6).

### Biological evaluation

#### Antibacterial activity determination through agar diffusion method

The bacterial growth inhibition activity of most of the newly synthesized compounds was examined against different Gram-positive and Gram-negative strains. The Agar diffusion method was used to assess the antibacterial and antifungal activity [56]. The Gram-negative strains were: *E. coli, K. pneumonia, P. aeruginosa* and *A. baumannii*, the Gram-positive strains were: *St. aureus, St. mutans* and the standard antibiotics were gentamicin, tigecycline and ampicillin. The IZD values were measured through the assay for the tested compounds, are shown in Table 3. The revealed data concluded that most of the tested compounds exhibited moderate to excellent antibacterial activity against the selected strains, but a few showed a broad-spectrum activity against both Gram-negative and Gram-positive bacteria.

**Table 3:** Revealing IZD values for the tested compounds in the Gram-negative bacteria, Gram-positive bacterial and fungal strains measured in mm

Cpd. No.	Gram-negative bacteria				Gram-positive bacteria	
	*E. coli*	*K. pneumonia*	*P. aeruginosa*	*A. baumannii*	*St. aureus*	*St. mutans*
St. Anti-biotic	Gentamicin	Gentamicin	Gentamicin	Tigecycline	Ampicillin	Ampicillin
	27 ± 0.1	29 ± 0.5	32 ± 0.4	23 ± 0.4	29 ± 0.2	22 ± 0.1
***2a***	*34 ± 0.5*	*34 ± 0.8*	*NA*	*NA*	*31 ± 1*	*NA*
**2b**	NA	NA	NA	NA	NA	NA
***2c***	*35 ± 1*	*35 ± 1*	*36 ± 1*	*35 ± 1*	*28 ± 1*	*25 ± 1*
***2e***	*35 ± 1*	*36 ± 1*	*36 ± 1*	*36 ± 1*	*20 ± 1*	*NA*
**4a**	11 ± 1	NA	NA	NA	NA	NA
**4b**	NA	NA	NA	13 ± 1	NA	NA
**4c**	NA	NA	NA	NA	NA	NA
***4d***	*20 ± 1*	*20 ± 1*	*NA*	*15 ± 1*	*NA*	*NA*
**4e**	NA	NA	NA	NA	NA	NA
***4f***	*25 ± 1.1*	*25 ± 1.1*	*20 ± 1*	*15 ± 1.1*	*25 ± 1.1*	*18 ± 1.1*
**4g**	18 ± 1	NA	NA	NA	NA	NA
***4i***	*20 ± 1*	*23 ± 1*	*NA*	*15 ± 1*	*12 ± 1*	*NA*
**4j**	NA	NA	NA	NA	NA	NA
***4k***	*22 ± 1.1*	*25 ± 1.1*	*20 ± 1*	*23 ± 1.1*	*25 ± 1.1*	*18 ± 1.1*
***4m***	*35 ± 1.1*	*35 ± 1.1*	*30 ± 1*	*35 ± 1.1*	*25 ± 1.1*	*28 ± 1.1*
**4n**	NA	NA	NA	NA	NA	NA
**4o**	NA	13 ± 1	NA	10 ± 1	NA	NA
***6a***	*20 ± 1*	*20 ± 1*	*23 ± 1*	*NA*	*NA*	*NA*
**6b**	NA	13 ± 1	NA	10 ± 1	NA	NA
**6c**	NA	NA	NA	13 ± 1	NA	NA
**6d**	NA	13 ± 1	NA	10 ± 1	NA	NA
***6e***	*31 ± 1*	*36 ± 0.8*	*NA*	*NA*	*29 ± 0.5*	*NA*
**6g**	NA	NA	NA	NA	NA	NA
**6h**	NA	NA	NA	13 ± 1	NA	NA
**6i**	18 ± 0.5	NA	NA	19 ± 0.5	14 ± 1	15 ± 1
**11a**	18 ± 0.5	NA	NA	19 ± 0.5	14 ± 1	15 ± 1
**11b**	19 ± 1	NA	NA	15 ± 1	NA	NA
***11c***	*35 ± 1*	*36 ± 1*	*35 ± 1*	*35 ± 1*	*NA*	*NA*
***11d***	*35 ± 1*	*23 ± 1*	*13 ± 1*	*10 ± 1*	*NA*	*NA*
**11e**	NA	NA	NA	NA	NA	NA
**11f**	17 ± 1.1	20 ± 1.1	16 ± 1	15 ± 1.1	20 ± 1.1	20 ± 1.1

It is worth noting that the 4-(2-(benzo[*d*]thiazol-2-yl)acetamido) benzoic acid **2e** exhibited excellent antibacterial activity with a broad spectrum profile against both Gram-negative and Gram-positive bacteria except *St. mutans* with better IZD values that are most better than the used standards, for example the IZDs of **2e** in *E. coli, K. pneumonia, P. aeruginosa* and *A. baumannii* were 35 ± 1, 36 ± 1, 36 ± 1 and 36 ± 1 mm, respectively compared to gentamicin and tigecycline that exhibited IZDs in *E. coli, K. pneumonia, P. aeruginosa and A. baumannii*: 27 ± 0.1, 29 ± 0.5, 32 ± 0.4 and 23 ± 0.4 mm, respectively. Also, compounds **2a** and **2c** revealed excellent antibacterial activities in almost all of the strains while the compound **2b** showed no activity against any bacterial strain. Thus, replacing the moieties: phenyl, 4-chlorophenyl and benzoic (compound **2a**, **2c** and **2e**, respectively) with tolyl (compound **2c**) deactivates the antibacterial activity.

Interesting results were obtained for the series of arylidenes **4**, which displaying good bacterial growth inhibition. The introduction of phenyl, 4-methoxyphenyl and dimethoxyphenyl in compound **2a** as in compounds **4a**, **4b** and **4c**, respectively didn`t enhance its antibacterial activity although it suppressed the activity. While, the introduction of 4-chlorophenyl (compound **4d**) enhanced the activity but not as potent as the parent compound **2a**. The arylidene derivatives of **2b**, **4e**–**g** revealed good antibacterial activity among them, the arylidene **4f**. Hence, introduction of the 4-methoxyphenyl moiety (compound **4f**) in the compound **2b** (contains methylphenyl moiety) activated the bacterial growth inhibition for the compound **4f** in more potent way than the parent **2b** (See Table 3). The compound **4k** revealed good broad-spectrum activity in both Gram-negative and Gram-positive strains. The compound **4k** is the arylidene derivative of **2c** that contains dimethoxyphenyl moiety. A similar behavior was revealed by both **4d** and **4i** towards the tested strains with the IZD values almost the same. The IZDs for **4d** and **4i** in *E. coli* were 20 ± 1 mm and in *A. baumannii* were 15 ± 1 mm for both **4d** and **4i**, also both compounds showed no activity against *P. aeruginosa*.

Furthermore, an excellent inhibition can be observed for 4-formylphenyl benzoate moiety as in case of **4m** that showed excellent IZD values better than the standard antibiotics in five strains: *E. coli, K. pneumonia, P. aeruginosa, A. baumannii and St. mutans* with IZD values: 35 ± 1.1, 35 ± 1.1, 30 ± 1, 35 ± 1.1 and 28 ± 1.1 mm, respectively compared to the standard antibiotics. The derivative **4o** showed moderate activity only against two strains: *K. pneumonia and A. baumannii* with IZD values: 13 ± 1 and 10 ± 1 mm, respectively. In contrast, the compound **4n** revealed no antibacterial activity toward any bacterial strains.

Introducing a phenyl pyrazole moiety in compound **2a** as in the arylidene derivative **6a** revealed good antibacterial activity in the strains; *E. coli, K. pneumonia and P. aeruginosa* with IZD values equal 20 ± 1, 20 ± 1 and 23 ± 1 mm, respectively, meanwhile, the arylidenes **6b** and **6c** which contains *p*-tolyl-pyrazole and 4-choloropyrazole, respectively showed moderate antibacterial activity. The arylidene derivatives **6a**–**c** suppressed the antibacterial activity of the parent compound **2a**. The compound **6e** which contains *p*-tolyl-pyrazole moiety revealed excellent IZD values in *E. coli*, *K. pneumonia and St. aureus* equal 31 ± 1, 36 ± 0.8 and 29 ± 0.5 mm, respectively, compared to the standard antibiotics that enhanced the antibacterial activity of the parent compound **2b** in marked manner. The arylidene derivatives **6g**–**i** of compound **2c** showed moderate to no activity in the tested strains except **6i** that showed good bacterial growth inhibition. The introduction of pyrazole derivatives in the compound **2c** suppressed its antibacterial activity. Furthermore, the compounds **6b** and **6d** showed the same antibacterial activity against the same bacterial strains, they showed only bacterial growth inhibition in *K. pneumonia* and *A. baumannii* with the same IZD values: 13 ± 1 and 10 ± 1 mm, respectively. The derivative **6c** showed an IZD value of 13 ± 1 mm in *A. baumannii* (See Table 3).

None of the tested pyridone derivatives **11** exhibited excellent inhibition against the tested strains except compounds **11c** and **11d**, pyridone derivatives of **2b**, which were more potent in bacterial growth inhibition. The compound **11c** was found to be more potent than the used standards in the Gram-negative strains: *E. coli, K. pneumonia, P. aeruginosa* and *A. baumannii* with IZD values: 35 ± 1, 36 ± 1, 35 ± 1 and 35 ± 1 mm, respectively. Excellent bacterial growth inhibition in *E. coli* was observed in the treated bacterial strain with **11d** showing an IZD value of 35 ± 1 mm higher than that of the standard gentamicin [IZD value = 27 ± 0.1 mm].

The MIC for the most potent compounds was detected to detect that there is no bacterial growth for 24 h at 37 °C [57] and detailed in Table 4. Serial dilution was operated for each compound to detect the least concentration that can cause bacterial death which be observed by unaided eye. It was observed that the MIC values of the tested compounds revealed better MIC values ~ 31.25–250 µg/L. For the parent compound **2**, compounds **2c** and **2e** were the most active antibacterial agents. Compound **2c** exhibited MIC value equals 31.25 µg/L in all tested Gram-negative and Gram-positive strains which was as the same as that of the used standard: gentamicin, tigecycline and ampicillin (See Table 4). The same was for compound **2e** except in showed MIC value equals 125 µg/L in *A. baumannii* while the standard tigecycline has MIC value equals 31.25 µg/L. The arylidene derivative **4m** was the most active antibacterial candidate among the other arylidene series **4**. The MIC of compound **4m** equals 31.25 µg/L in all bacterial strains. The second active antibacterial agent was **4f** that revealed MIC value in *E. coli* equals 62.5 µg/L (MIC of gentamicin = 31.25 µg/L) and in *K. pneumonia* equals 31.25 µg/L which was more potent than the used antibiotic (MIC of gentamicin = 62.5 µg/L). In contrast, compound **4f** showed MIC value in *P. aeruginosa* equals 125 µg/L while the MIC of gentamicin = 31.25 µg/L. The same behavior of **4f** was observed in the pyrazolyl derivative **6a** (See Table 4). The pyridone **11c** showed excellent MIC values in *E*. *coli, K. pneumonia, P. aeruginosa* and *A. baumannii* that was more potent than the used antibiotics, while **11d** was as active as gentamicin in *E. coli* and *K. pneumonia* with the same MIC values. As concluded, the compounds **2c**, **4m** and **11c** were the most active antibacterial candidates among the newly synthesized compounds.

**Table 4:** Revealing MIC values for the tested compounds in the Gram-negative and Gram-positive bacteria measured in µg/L

Cpd. No.	Gram-negative bacteria				Gram-positive bacteria	
	*E. coli*	*K. pneumonia*	*P. aeruginosa*	*A. baumannii*	*St. aureus*	*St. mutans*
St. Antibiotic	Gentamicin	Gentamicin	Gentamicin	Gentamicin	Ampicillin	Ampicillin
	31.25	62.5	31.25	31.25	62.5	62.5
**2a**	250	125	–	125	250	62.5
***2c***	*31.25*	*31.25*	*31.25*	*31.25*	*31.25*	*31.25*
**2e**	31.25	31.25	31.25	125	–	–
**4d**	125	125	–	250	–	–
**4f**	62.5	62.5	125	–	–	–
**4i**	125	125	–	–	–	–
**4k**	125	62.5	125	31.25	62.5	–
***4m***	*31.25*	*31.25*	*31.25*	*31.25*	*31.25*	*31.25*
**6a**	62.5	62.5	125	–	–	–
**6e**	–	–	–	–	–	–
***11c***	*31.5*	*31.5*	*31.5*	*31.5*	*–*	*–*
**11d**	31.5	62.5	–	–	–	–

#### SAR for antibacterial activity

**Fig. 6:**
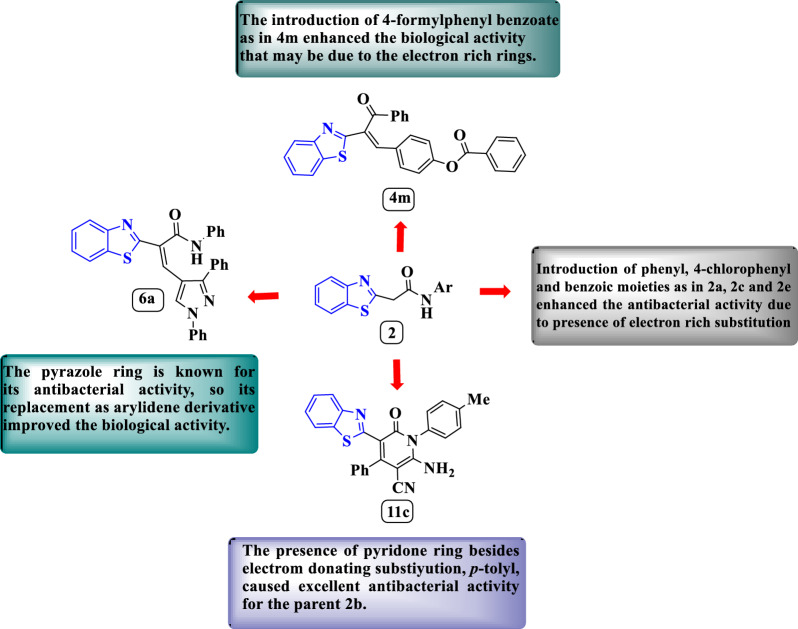
the SAR diagram explains the antibacterial activity

The biological activity data resulted from the Agar diffusion method and MIC assays was explained according to the SAR and diagrammed as in Fig. 6. From the antimicrobial activity assessment`s results, we concluded that the hybridization between the benzo[*d*]thiazole moeity and each of *N*-phenylacetamide, *N*-(4-chlorophenyl)-acetamide and *N*-(3-carboxyphenyl)acetamide enhanced the antibacterial activity in the compounds **2a**, **2c** and **2e**, respectively, that may be their electron rich nature. The introduction of 4-formylphenyl benzoate into the parent compound **2a** was the cause of the excellent antimicrobial growth as observed in **4m** it may be reasoned for the benzoate antibacterial activity [58]. The benzo[*d*]thiazole-pyrazole derivative **6a** exerted excellent antimicrobial activity that may be due to the presence of pyrazolyl moiety which known for enhancing the antibacterial activity [59] besides being electron rich ring. Similarly, the excellent antimicrobial activity of **11c** and **11d** may be due to the presence of *p*-tolyl and *p*-methoxy phenyl moieties which are donating substitutions, also, the introduction of pyridone ring, proved to have antibacterial activity property [60], containing electron donating moiety such as amino group besides *p*-tolyl and *p*-methoxy phenyl moieties improved the antibacterial activity of the parent compound **2b** that found inactive antibacterial agent.

### In vitro DHFR inhibition assessment for 2c, 4m and 11c

The DHFR inhibition activity of the most active antibacterial candidates; **2c**, **4m** and **11c** were tested in vitro using trimethoprim as a reference drug. The obtained data are detailed with IC_50_ in Table 5. The tested compounds revealed potent inhibitory activity than trimethoprim except compound **11c**. As displayed in Table 5, all the tested compounds showed effective DHFR activity inhibition with IC_50_ values ranging from 2.442 to 15.04 μM, relative to trimethoprim reference drug (IC_50_ = 8.706 ± 0.455 μM). Compounds **2c** and **4m** displayed excellent DHFR inhibitory activity with effective down regulation in the DHFR concentration (IC_50_ = 3.796 ± 0.198 and 2.442 ± 0.128 μM, respectively), highly equipotent in compared to trimethoprim. Additionally, compound **11c**'s efficacy was almost half that of trimethoprim against DHFR with IC_50_ = 15.04 ± 0.785 μM. Significantly, compound **4m** proved to be the most effective derivative among the examined compounds in the assessment, with an IC_50_ that was lower than the reference drug trimethoprim.

**Table 5:** Revealed data of inhibitory investigation of compounds **2c**, **4 m** and **11c** for DHFR enzyme using trimethoprim as a standard drug

Cpd. No.	DHFR IC_50_ (mean ± SD) μM
Trimethoprim	8.706 ± 0.455
**2c**	3.796 ± 0.198
**4m**	2.442 ± 0.128
**11c**	15.04 ± 0.785

#### SAR for enzyme inhibition investigation

**Fig. 7:**
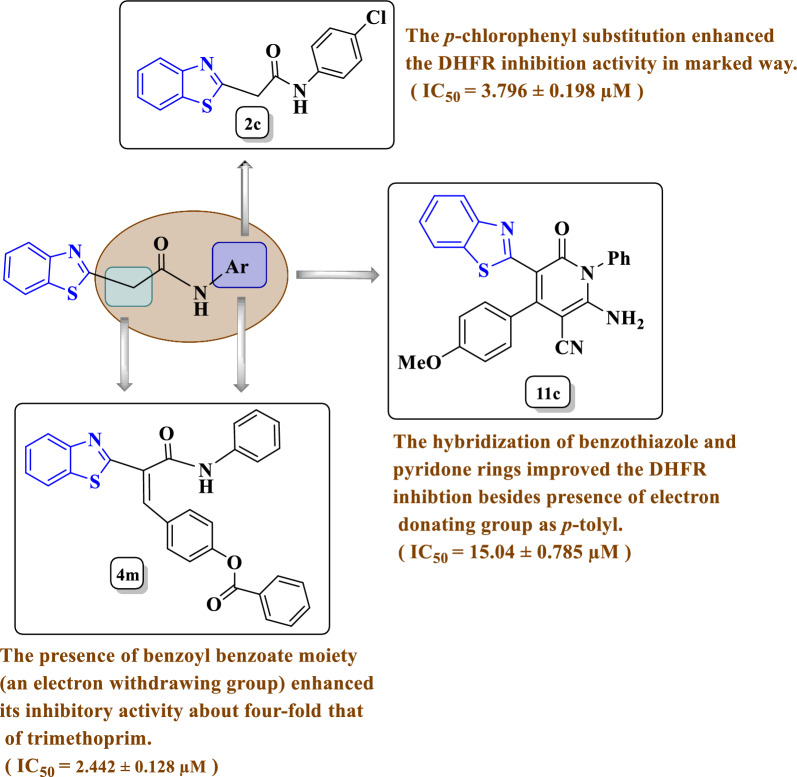
the SAR diagram explains the enzyme inhibition activity for **2c**, **4m** and **11c**

The SAR study has focused on the influence of occurrence of substitution of the parent acetamide **2** on its inhibitory activity against DHFR enzyme. The SAR diagram describing the DHFR activity inhibition is displayed in Fig. 7. As mentioned in Table 5, the compound **2c** excreted excellent DHFR down regulation that may be due to the presence of *N*-(4-chlorophenyl) group in **2c** enhanced its inhibitory activity against DHFR about two-fold that of trimethoprim that may be due to the electron withdrawing effect exerted by *N*-(4-chlorophenyl) group found in benzo[*d*]thiazole-acetamide **2c**. For compound **4m**, it was the most active DHFR enzyme inhibitor and more potent than trimethoprim and that may be reasoned for the presence of benzoyl benzoate moiety (an electron withdrawing group) that improved its inhibitory activity about four-fold that of trimethoprim. As noted, the benzo[*d*]thiazole-*N*-arylpyridone **11c** revealed effective inhibitory activity against DHFR but not as equipotent as trimethoprim that may be due to the presence of *p*-tolyl moiety which is an electron donating group, also, the hybridization between benzothiazole and pyridone caused improvement in the inhibitory activity (Fig. 7).

## Computational studies

### In silico toxicity potential by Osiris property explorer

All newly synthesized compounds have been checked for toxic effects because drug design considers a compound's potential toxicity such as mutagenic, tumorigenic properties and skin irritants, besides their effect on the reproductive system via Osiris methodology [61]. The Osiris methodology is a prediction technique that relies on a precomputed set of structural fragments that, when present in the drawn structure, cause toxicity alerts. In addition to completely decomposing all compounds identified as active in a particular toxicity class (e.g., mutagenicity) in the RTECS database. Prediction results are valued and color coded; green color: shows less toxic, orange color: shows mid toxic, red color: shows high tendency of toxicity. The obtained results are detailed in Table 6. The results revealed that the majority of the synthesized compounds are safe as drug candidates. The compounds 2-(benzo[*d*]thiazol-2-yl)-*N*-arylacetamides **2a**–**f** showed good pharmaceutical properties with no health hazards or toxic effects except **2d** revealed the potential for medium risk in causing irritation as it containing chlorine atom at the ortho position in the phenyl moiety of the anilide, in addition being with no risk as tumor causing products, mutagenic agents or with reproductive effect. Also, the derivatives **4a**–**o** found to be safe with excellent pharmacochemical properties in all aspects. The benzo[*d*]thiazole-pyrazole derivatives **6a**–**i** exhibited no health hazards or toxic effects with good properties. In contrast, the *N*-aryl pyridone derivatives **11a**–**f**, some of them showed toxicity in some aspects such as **11b**, **11d** and **11f**, while the rest of the derivatives revealed good properties (Table 6). The compound **11b**, **11d** and **11f** showed high risk on the reproductive system due to containing p-methoxy group attached to the phenyl moeity attached to pyridine ring.

**Table 6:** Predicted toxicity risks and physicochemical properties obtained according to Osiris property explorer software

Comp. no	Toxicity risks				Solubility	Drug-likeness	Drug score	TPSA
	Mutagenicity	Tumorgenicity	Irritancy	Reproductive effect				
**2a**	Green	Green	Green	Green	− 3.36	2.0	0.8	70.23
**2b**	Green	Green	Green	Green	− 3.7	2.88	0.77	70.23
**2c**	Green	Green	Green	Green	− 4.09	4.54	0.75	70.23
**2d**	Green	Green	Orange	Green	− 4.83	4.31	0.5	70.23
**2e**	Green	Green	Green	Green	− 3.37	2.83	0.82	107.5
**2f**	Green	Green	Green	Green	− 2.62	3.97	0.91	96.01
**4a**	Green	Green	Green	Green	− 4.72	2.22	0.56	70.23
**4b**	Green	Green	Green	Green	− 4.74	3.43	0.57	79.46
**4c**	Green	Green	Green	Green	− 4.75	5.18	0.56	88.69
**4d**	Green	Green	Green	Green	− 5.45	3.98	0.45	70.23
**4e**	Green	Green	Green	Green	− 5.06	2.44	0.5	70.23
**4f**	Green	Green	Green	Green	− 5.08	3.63	0.5	79.46
**4g**	Green	Green	Green	Green	− 5.1	5.41	0.49	88.69
**4h**	Green	Green	Green	Green	− 5.8	4.17	0.4	70.23
**4i**	Green	Green	Green	Green	− 5.45	4.63	0.46	70.23
**4j**	Green	Green	Green	Green	− 5.47	5.8	0.45	79.46
**4k**	Green	Green	Green	Green	− 5.49	7.5	0.43	88.69
**4l**	Green	Green	Green	Green	− 6.19	5.98	0.35	70.23
**4m**	Green	Green	Green	Green	− 6.19	4.13	0.31	96.53
**4n**	Green	Green	Green	Green	− 6.53	4.34	0.27	96.53
**4o**	Green	Green	Green	Green	− 6.92	6.44	0.24	96.53
**6a**	Green	Green	Green	Green	− 6.23	5.97	0.32	88.05
**6b**	Green	Green	Green	Green	− 6.25	5.88	0.31	97.28
**6c**	Green	Green	Green	Green	− 6.97	6.9	0.25	88.05
**6d**	Green	Green	Green	Green	− 6.59	6.05	0.27	97.28
**6e**	Green	Green	Green	Green	− 6.92	5.93	0.25	88.05
**6f**	Green	Green	Green	Green	− 7.31	7.02	0.22	88.05
**6g**	Green	Green	Green	Green	− 6.97	8.22	0.25	88.05
**6h**	Green	Green	Green	Green	− 7.31	6.66	0.22	88.05
**6i**	Green	Green	Green	Green	− 7.7	8.77	0.2	88.05
**11a**	Green	Green	Green	Green	− 6.1	1.74	0.48	111.2
**11b**	Green	Green	Green	Red	− 6.12	1.9	0.27	120.4
**11c**	Green	Green	Green	Green	− 6.45	− 0.29	0.32	111.2
**11d**	Green	Green	Green	Red	− 6.46	− 0.14	0.19	120.4
**11e**	Green	Green	Green	Green	− 6.84	1.54	0.37	111.2
**11f**	Green	Green	Green	Red	− 6.86	1.67	0.21	120.4

Green color: shows less toxic, orange color: shows mid toxic, red color: shows high tendency of toxicity

#### Swiss ADME predictions of 2c, 4m and 11c

The most antibacterial active candidates **2c**, **4m** and **11c** were subjected for more in silico studies using Swiss ADME free application (http://www.swissadme.ch). The ADME is mainly used in fields such as pharmacokinetics and pharmacology. The four letter stands for descriptors quantifying how a given drug interacts within body over time, that defines the compounds' drug-likeness and consequently their medical chemical characteristics by establishing these assessments on their pharmacokinetics, physicochemical properties, lipophilicity, and solubility. The data obtained from Swiss ADME application for **2c**, **4m** and **11c** was detailed in Table S1 (see Electronic supplemental file).

The absorption factor describes how a substance enters a tissue, which is typically through the circulation and frequently occurs through intestinal absorption, which occurs when mucous surfaces in the digestive tract absorb a component before the target cells absorb it. For that the compound`s solubility is an important factor that affects its absorption. From the revealed data, the water solubility of these compounds found to be insoluble except **2c**, which is water moderately soluble. The number of rotatable bonds is low for **2c**, **4m** and **11c** as the following 4, 8 and 3, respectively, their molar refractivity equals 83.43, 140.22 and 130.17 for **2c**, **4m** and **11c**, respectively. The lipophilicity properties for **2c**, **4m** and **9c** depends on calculating five factors ILOGP, XLOGP3, WLOGP, MLOGP and SILICOS-IT and they range from 2.68 to 6.72. The compound **2c** is found to be high gastrointestinal absorption, while **4m** and **9c** were found to of low gastrointestinal absorption. Besides, Absorption critically determines how the compound can be administrated; either by oral intravenously or by inhalation administration. Compounds that absorb poorly when taken orally must be administered in some less desirable way, like intravenously or by inhalation, so that compound** 2c** can by administrated orally due to its high gastrointestinal absorption. Absorption critically determines the compound's bioavailability. The bioavailability refers to the extent a substance or drug becomes completely available to its intended biological destination. The bioavailability score equals ≥ 0.55 is considered ideal and absorbed very well by the body [62], so compounds **2c**, **4m** and **11c** are considered ideal drug from the absorption aspects as they have bioavailability score equals 0.55. The Distribution factor is defined as the reversible transfer of a compound between one compartments to another. Some factors affecting drug distribution include regional blood flow rates, molecular size, polarity and binding to serum proteins, forming a complex. Distribution can be a serious problem at some natural barriers like the blood–brain barrier. As noted from the obtained data, the TPSA for **2c** is 70.23 Å^2^ and its molecular weight is lower than 500 g/mol while that of **4m** and **9c** are 96.53 and 112.94 Å^2^, respectively with molecular weights equal 476.55 and 434.51 g/mol, respectively.

The drug's breakdown is determined by the metabolism factor since the chemical breaks down into metabolites as soon as it enters the body. That usually occurs in the liver by redox enzymes termed cytochrome P450. All the compounds **2c**, **4m** and **9c** are cytochrome P450 inhibitors leading to drug-drug interactions. According to Drug-likeness rules, compound **2** obeyed all the rules; Lipinski, Veber, Muegge, Ghose and Egan rules it showed no violation. The Lipinski rule states: MW less than 500, no more than 5 hydrogen bond donors, no more than 10 hydrogen bond acceptors and A calculated Clog P that does not exceed 5. Compound **2c** obeyed all the rules; Lipinski, Veber, Muegge, Ghose and Egan rules it showed no violation, while **4m** obeyed Lipinski and Muegge rules` parameters, it showed violations in other rules in compare to compound **9c** it obeyed Lipinski, Muegge and Ghose rules only. The synthetic accessibility for a compound was stated that the value equals 1 for synthetic accessibility means it is very easy to obtain while the value equals 10 means it is very difficult to obtain. The synthetic accessibility for **2c**, **4m** and **9c** are 2.48, 3.55 and 3.66, respectively.

## Conclusion

In this context, Novel *N*-arylacetamides **2a**–**f** were synthesized bearing benzo[*d*]thiazole moiety. The starting compounds **2a**–**c** underwent Knoevenagel condensation through green synthetic method with different aromatic aldehydes and pyrazole-7- carbaldehydes delivered the respective arylidenes with efficient yields. The arylidenes **4** reacted with malononitrile to afford the corresponding *N*-arylpyridones **11a**–**i**. Furthermore, **2a**–**c** reacted with each of salicylaldehyde and 5-arylazo salicylaldehydes giving the unexpected coumarins rather than the expected quinolin-5-ones. The structure of coumarin **8** was confirmed by DFT calculations using basis set B3LYP/6-311 G +  + (d,p) to obtain the suitable geometrical structure with molecular orbitals` energies revealing its planar structure and its agreement with experimental data. The antibacterial activity was investigated against different bacterial strains revealing potent activity especially Gram-negative bacteria with excellent MIC value ranging from 31.25 to 250 µg/L. Moreover, compounds **2c** and **4m** showed enzyme inhibition against dihydrofolate reductase in *E. coli* with greater potency (IC_50_ for **2c** = 3.796 µM, IC_50_ for **4m** = 2.442 µM) than the standard antibiotic trimethoprim (IC_50_ = 8.706 µM). Investigation of the physicochemical properties of the newly compounds exhibited their better ADME properties that can be developed for the discovery of new antibacterial agents.

## Supplementary Information

Supplementary Material 1.

## Data Availability

No datasets were generated or analysed during the current study.

## Abbreviations

*E. coli*: *Escherichia coli*
*K. pneumonia*: *Klebsiella pneumonia*
*A. baumannii*: *Acinetobacter baumannii*
*P. aeruginosa*: *Pseudomonas aeruginosa*
*St. aureus*: *Staphylococcus aureus*
*St. pneumonia*: *Streptococcus pneumonia*
DHFR: Dihydrofolate reductase
THF: Tetrahydrofolate
*E. faecalis*: *Enterococcus faecalis*
DFT: Density functional theory
D. M.: Dipole moment
HOMO: The acronym for the highest occupied molecular orbital
LUMO: The acronym for the lowest unoccupied molecular orbital
IZD: Inhibition zone diameter
MIC: Minimum inhibition concentration
SAR: Structure activity relationship
IC_50_: Inhibition concentration of the compounds causing 50% down regulation of the enzyme activity
RTECS: Registry of Toxic Effects of Chemical Substances
ADME: The four-letter abbreviation for absorption, distribution, metabolism, and excretion
TPSA: Topological polar surface area
MW: Molecular weight
Clog P: Octanol-water partition coefficient

## References

[1] CDC Antibiotic resistance threats in the United States, Report. 2019; 2019.

[2] Prestinaci F, Pezzotti P, Pantosti A. Antimicrobial resistance: a global multifaceted phenomenon. Pathog Glob Health. 2015;109:309–18. 26343252 10.1179/2047773215Y.0000000030 PMC4768623

[3] Antimicrobial Resistance Collaborators. Global burden of bacterial antimicrobial resistance in 2019: a systematic analysis. Lancet. 2022;399:629–55. 35065702 10.1016/S0140-6736(21)02724-0 PMC8841637

[4] Ponnampalli S. Synthesis of new benzothiazole derivatives as potential antimicrobial agents. CVR J Sci Technol. 2022;23:150–4.

[5] Rakesh KP, Vivek HK, Manukumar HM, Shantharam CS, Bukhari SNA, Qin HL, Sridhara MB. Promising bactericidal approach of dihydrazone analogues against bio-film forming Gram-negative bacteria and molecular mechanistic studies. RSC Adv. 2018;8:5473–83. 35542417 10.1039/c7ra13661g PMC9078102

[6] Rakesh KP, Ramesh S, Kumar HMM, Chandan S, Gowda DC. Quinazolinones linked amino acids derivatives as a new class of promising antimicrobial, antioxidant and anti-inflammatory agents. Eur J Chem. 2015;6:254–60.

[7] Li C, Sridhara MB, Rakesh KP, Vivek HK, Manukumar HM, Shantharam CS, Qin HL. Multi-targeted dihydrazones as potent biotherapeutics. Bioorg Chem. 2018;81:389–95. 30199841 10.1016/j.bioorg.2018.08.024

[8] Lungu L, Blaja S, Cucicova C, Ciocarlan A, Barba A, Kulcițki V, Shova S, Vornicu N, Geana EI, Mangalagiu II, Aricu A. Synthesis and antimicrobial activity evaluation of homodrimane sesquiterpenoids with a benzimidazole unit. Molecules. 2023;28:933. 36770601 10.3390/molecules28030933 PMC9921711

[9] Sharma M, Chauhan PMS. Dihydrofolate reductase as a therapeutic target for infectious diseases: opportunities and challenges. Future Med Chem. 2012;4:1335–65. 22800373 10.4155/fmc.12.68

[10] Thakkar SS, Thakor P, Ray A, Doshi H, Thakkar VR. Benzothiazole analogues: Synthesis, characterization, MO calculations with PM6 and DFT, in silico studies and in vitro antimalarial as DHFR inhibitors and antimicrobial activities. Bioorg Med Chem. 2017;25:5396–406. 28789907 10.1016/j.bmc.2017.07.057

[11] Khilya OV, Milokhov DS, Kononets LA, Kobzar OL, Vovk AI, Volovenko YM. Synthesis and evaluation of new 2,6-diamino-5-hetarylpyrimidines as inhibitors of dihydrofolate reductase. Monatsh Chem. 2018;149:813–22.

[12] Suyambulingam JK, Karvembu R, Bhuvanesh NSP, Enoch IVMV, Selvakumar PM, Premnath D, Subramanian C, Mayakrishnan P, Kim SH, Chung IM. Synthesis, structure, biological/chemosensor evaluation and molecular docking studies of aminobenzothiazole schiff bases. J Adhes Sci Technol. 2020;34:2590–612.

[13] Sumit, Kumar A, Mishra AK. Advancement in pharmacological activities of benzothiazole and its derivatives: an up to date review. Mini Rev Med Chem. 2021;21:314–35. 32819243 10.2174/1389557520666200820133252

[14] Ballari MS, Cano NH, Wunderlin DA, Feresin GE, Santiago AN. One-pot sequential synthesis and antifungal activity of 2-(benzylsulfonyl)benzothiazole derivatives. RSC Adv. 2019;9:29405–13.

[15] Baht M, Belagali SL. Structural activity relationship and importance of benzothiazole derivatives in medicinal chemistry: a comprehensive review. Mini Rev Org Chem. 2020;17:323–50.

[16] Dhumal ST, Deshmukh ARK, Kharat R, Sathe BR, Chavan SS, Mane RA. Copper fluorapatite assisted synthesis of new 1,2,3-triazoles bearing a benzothiazolyl moiety and their antibacterial and anticancer activities. New J Chem. 2019;43:7663–73.

[17] Sun XT, Hu ZG, Huang Z, Zhou LL, Weng JQ. A Novel PIFA/KOH promoted approach to synthesize C2-arylacylated benzothiazoles as potential drug scaffolds. Molecules. 2022;27:726. 35163992 10.3390/molecules27030726 PMC8838045

[18] Mai NTN, Huan TT, Huan NV, Hoan DQ. Synthesis and antimicrobial activities of hydrazones derived from 4-hydroxy-3-nitrobenzaldehyde. Vietnam J Sci Technol. 2023;6:373–81.

[19] Tratrat C, Petrou A, Geronikaki A, Ivanov M, Kostić M, Soković M, Vizirianakis IS, Theodoroula NF, Haroun M. Thiazolidin-4-ones as potential antimicrobial agents: experimental and in silico evaluation. Molecules. 2022;27:1930. 35335296 10.3390/molecules27061930 PMC8954104

[20] Kaushik CP, Chanal M. Synthesis and antibacterial activity of benzothiazole and benzoxazole-appended substituted 1,2,3-triazoles. J Chem Sci. 2020;132:142.

[21] Haroun M, Tratrat C, Petrou A, Geronikaki A, Ivanov M, Ciric A, Sokovic M. 2-Aryl-3-(6-trifluoromethoxy)benzo[*d*]thiazole-Based thiazolidinone hybrids as potential anti-infective agents: Synthesis, biological evaluation and molecular docking studies. Bioorg Med Chem Lett. 2021;32:1–12. 10.1016/j.bmcl.2020.127718 33253880

[22] Skok Z, Barančoková M, Benek O, Cruz CD, Tammela P, Tomašič T, Zidar N, Mašič LP, Zega A, Stevenson CEM, Mundy JEA, Lawson DM, Maxwell A, Kikelj D, Ilaš J. Exploring the chemical space of benzothiazole—based DNA gyrase B inhibitors. ACS Med Chem Lett. 2020;11:2433–40. 33329764 10.1021/acsmedchemlett.0c00416 PMC7734788

[23] Nehra N, Tittal RK, Ghule VD. 1,2,3-Triazoles of 8-hydroxyquinoline and HBT: Synthesis and studies DNA binding, antimicrobial, molecular docking, ADME, and DFT. ACS Omega. 2021;6:27089–100. 34693129 10.1021/acsomega.1c03668 PMC8529673

[24] Ghannam IAY, El-Meguid EAA, Ali IH, Sheir DH, ElKerdawy AM. Novel 2-arylbenzothiazole DNA Gyrase inhibitors: synthesis, antimicrobial evaluation, QSAR and molecular docking Studies. Bioorg Chem. 2019;93:1–44. 10.1016/j.bioorg.2019.103373 31698294

[25] Ghanavatkar CW, Mishra VR, Mali SN, Chaudhari HK, Sekar N. Synthesis, bioactivities, DFT and in-Silico appraisal of azo glubbed benzothiazole derivatives. J Mol Struct. 2019;1192:162–71.

[26] Azzam RA, Elboshi HA, Elgemeie GH. Synthesis, physicochemical properties and molecular docking of new benzothiazole derivatives as antimicrobial agents targeting DHPS enzyme. Antibiotics. 2022;11:1799. 36551457 10.3390/antibiotics11121799 PMC9774648

[27] Naaz F, Srivastava R, Singh A, Singh N, Verma R, Singh VK, Singh RK. Molecular modeling, synthesis, antibacterial and cytotoxicity evaluation of sulfonamide derivatives of benzimidazole, indazole, benzothiazole and thiazole. Bioorg Med Chem. 2018;26:3414–28. 29778528 10.1016/j.bmc.2018.05.015

[28] Morsy MA, Ali EM, Kandeel M, Venugopala KN, Nair AB, Greish K, El-Daly M. Screening and molecular docking of novel benzothiazole derivatives as potential antimicrobial agents. Antibiotics. 2020;9:221. 32365587 10.3390/antibiotics9050221 PMC7277330

[29] Mishra R, Chaurasia H, Singh VK, Naaz F, Singh RK. Molecular modeling, QSAR analysis and antimicrobial properties of Schiff base derivatives of isatin. J Mol Struct. 2021;1243:130763.

[30] Kousaxidis A, Kovacikova L, Nicolaou I, Stefek M, Geronikaki A. Non-acidic bifunctional benzothiazole-based thiazolidinones with antimicrobial and aldose reductase inhibitory activity as a promising therapeutic Strategy for sepsis. Med Chem Res. 2022;30:1837–48. 10.1007/s00044-021-02778-7 PMC8335715 34366640

[31] Metwally NH, Abdelrazek FM, Eldaly SM, Metz P. 3-(3, 5-Dimethyl-1*H*-pyrazol-1-yl)-3-oxopropanenitrile as precursor for some new mono-heterocyclic and bis-heterocyclic compounds. J Heterocycl Chem. 2017;54:289–94.

[32] Metwally NH, Badawy MA, Okpy DS. Synthesis and anticancer activity of some new thiopyrano[2,3-*d*]thiazoles incorporating pyrazole moiety. Chem Pharm Bull. 2015;63:495–503. 10.1248/cpb.c14-00885 26133066

[33] Metwally NH, Abdelrazek FM, Eldaly SM. Synthesis and anticancer activity of some new heterocyclic compounds based on 1-cyanoacetyl-3,5-dimethylpyrazole. Res Chem Intermed. 2016;42:1071–89.

[34] Metwally NH, Deeb EA. Synthesis, anticancer assessment on human breast, liver and colon carcinoma cell lines and molecular modeling study using novel pyrazolo[4,3-*c*]pyridine derivatives. Bioorg Chem. 2018;77:203–14. 29367077 10.1016/j.bioorg.2017.12.032

[35] Metwally NH, Abdelrazek FM, Eldaly SM. Synthesis, molecular docking, and biological evaluation of some novel bis-heterocyclic compounds based *N*, *N*′-([1,1′-biphenyl]-4,4′-diyl)bis(2-cyanoacetamide) as potential anticancer agents. J Heterocycl Chem. 2018;55:2668–82.

[36] Metwally NH, Radwan IT, El-Serwy WS, Mohamed MA. Design, synthesis, DNA assessment and molecular docking study of novel 2-(pyridin-2-ylimino) thiazolidin-4-one derivatives as potent antifungal agents. Bioorg Chem. 2019;84:456–67. 30576909 10.1016/j.bioorg.2018.11.050

[37] Metwally NH, Mohamed MS, Ragab EA. Design, synthesis, anticancer evaluation, molecular docking and cell cycle analysis of 3-methyl-4,7-dihydropyrazolo[1,5-*a*]pyrimidine derivatives as potent histone lysine demethylases (KDM) inhibitors and apoptosis inducers. Bioorg Chem. 2019;88:102929. 31015179 10.1016/j.bioorg.2019.102929

[38] Metwally NH, Saad GR, Abd El-Wahab EA. Grafting of multiwalled carbon nanotubes with pyrazole derivatives: characterization, antimicrobial activity and molecular docking study. Int J Nanomed. 2019;14:6645–59. 10.2147/IJN.S182699 PMC6709384 31686804

[39] Metwally NH, Abdelrazek FM, Eldaly SM. Synthesis, reactions, and antimicrobial activity of 2-cyano-*N*`-(4-(2-oxo-2-phenylethoxy)benzylidene)acetohydrazide deivatives. J Heterocycl Chem. 2020;57:3653–63.

[40] Metwally NH, Abdallah SO, Mohsen MM. Design, green one-pot synthesis and molecular docking study of novel *N, N*-bis(cyanoacetyl)hydrazines and bis-coumarins as effective inhibitors of DNA gyrase and topoisomerase IV. Bioorg Chem. 2020;97:103672. 32145481 10.1016/j.bioorg.2020.103672

[41] Metwally NH, Mohamed MS, Deeb EA. Synthesis, anticancer evaluation, CDK2 inhibition, and apoptotic activity assessment with molecular docking modeling of new class of pyrazolo[1,5-*a*]pyrimidines. Res Chem Intermed. 2021;47:5027–60.

[42] Metwally NH, Abd-Elmoety AS. Novel fluorinated pyrazolo[1,5-*a*]pyrimidines: In a way from synthesis and docking studies to biological evaluation. J Mol Struct. 2022;1257:132590.

[43] Metwally NH, Badawy MA, Okpy DS. Synthesis, biological evaluation of novel thiopyrano[2,3-*d*]thiazoles incorporating arylsulfonate moiety as potential inhibitors of tubulin polymerization, and molecular modeling studies. J Mol Struct. 2022;1258:132648.

[44] Metwally NH, Eldaly SM. Design, synthesis of new pyrazoles and chromenes as ERK-2 inhibitors, apoptosis inducers and cell cycle interrupters based on thiophene-chalcone scaffold. ChemistrySelect. 2022;7: e202202257.

[45] Eldaly SM, Zakaria DS, Metwally NH. Design, synthesis, anticancer evaluation and molecular modeling studies of new thiazolidinone-benzoate scaffold as EGFR inhibitors, cell cycle interruption and apoptosis inducers in HepG2. Chem Biodivers. 2023;20: e202300138. 37695095 10.1002/cbdv.202300138

[46] Chao RY, Ding FM, Chen JY, Lee CC, Lin ST. Preparation and charcterization of 3-substituted benzothiazol-2-yl coumarins. J Chin Chem Soc. 2010;57:213–21.

[47] Metwally NH, Elgemeie GH, Fahmy FG. Green synthesis: antimicrobial activity of novel benzothiazole-bearing coumarin derivatives and their fluorescence properties. Egypt J Chem. 2022;65:679–86.

[48] Frisch MJ, Trucks GW, Schlegel J, Scuseria GE, Robb MA, Cheeseman JR, Schlegel HB, Scalmani G, Barone V, Mennucci B, Petersson GA. Gaussian 09, Revis. C.01. Wallingford: Gaussian Inc.; 2010.

[49] Becke AD. Density-functional exchange-energy approximation with correct asymptotic. Behavior Phys Rev A. 1988;38:3098. 10.1103/physreva.38.3098 9900728

[50] Becke AD. Density-functional thermochemistry. III. The role of exact exchange. J Chem Phys. 1993;98:5648–52.

[51] Johnson B, Frisch MJ. Analytic second derivatives of the gradient-corrected density functional energy: effect of quadrature weight derivatives. Chem Phys Lett. 1993;216:133–40.

[52] Lee C, Yang W, Parr RG. Development of the Colle-Salvetti correlation-energy formula into a functional of the electron density. Phys Rev B. 1988;37:785. 10.1103/physrevb.37.785 9944570

[53] McLean AD, Chandler GS. Contracted Gaussian basis sets for molecular calculations. I. Second row atoms, Z = 11–18. J Chem Phys. 1980;72:5639–48.

[54] Ulic SE, Védova COD, Hermann A, Mack H-G, Oberhammer H. Preparation and properties of trifluorothioacetic acid-S-(trifluoromethyl) ester, CF_3_C (O) SCF_3_. J Phys Chem A. 2008;112:6211–6. 18547036 10.1021/jp800344m

[55] Reed AE, Weinhold F. Natural bond orbital analysis of near-Hartree-Fock water dimer. J Chem Phys. 1938;78:4066–73.

[56] Scott C, et al. Laboratory control of cntimicrobial therapy. In: Collee JG, et al., editors. Practical medical microbiology. 13th ed. Edinburgh: Churchill Livingstone; 1989. p. 161.

[57] Wiegand I, Hilpert K, Hancock REW. Agar and broth dilution methods to determine the minimal inhibitory concentration (MIC) of antimicrobial substances. Nat Protoc. 2008;3:163–75. 18274517 10.1038/nprot.2007.521

[58] Diastuti H, Chasani M, Suwandri S. Antibacterial activity of benzyl benzoate and crotepoxide from *Kaempferia rotunda* L. Rhizome. Indones J Chem. 2020;20(1):9–15.

[59] Barghady N, Chalkha M, Yamari I, Aflak N, Abchir O, Chebbac K, Nakkabi A, Chtita S, Chkirate K, Mague JT, Mabrouk EH. Synthesis, characterization, mechanistic study, in-vitro and in-silico evaluation of antibacterial and antioxidant activities of novel pyrazole-pyrazoline hybrid systems. J Mol Struct. 2024;1309:138087.

[60] Saadon KE, Taha NM, Mahmoud NA, Elhagali GA, Ragab A. Synthesis, characterization, and in vitro antibacterial activity of some new pyridinone and pyrazole derivatives with some in silico ADME and molecular modeling study. J Iran Chem Soc. 2022;19(9):3899–917.

[61] Sanders T. The OSIRIS property explorer software. http://www.organic-chemistry.org/prog/peo/.

[62] Martin YC. A bioavailability score. J Med Chem. 2005;48:3164–70. 10.1021/jm0492002. 15857122 10.1021/jm0492002

