# Supplementary material for: Synthesis, antibacterial evaluation and in silico studies of novel 2-(benzo[d]thiazol-2-yl)-N-arylacetamides and their derivatives as potential DHFR inhibitors
Source: BMC Chem. 2025 Jan 31;19(1):29. doi: 10.1186/s13065-025-01386-5 (PMC11784096; doi:10.1186/s13065-025-01386-5)
Supplement: Supplementary file 1 — Supplementary Material 1. [file 13065_2025_1386_MOESM1_ESM.docx]

**Synthesis, antibacterial evaluation and in silico studies of novel 2-(benzo[*d*]thiazol-2-yl)-*N*-arylacetamides and their derivatives as potential DHFR inhibitors**

***Nadia Hanafy Metwally^1^*, Galal Hamza Elgemeie^2^, Aya Ragab Abdelrazek^1^ and Salwa Magdy Eldaly^1^***

**Email of Corresponding author:** mnadia@sci.cu.edu.eg

**Experimental**

**Materials and Methods**

The melting points are unedited and have been established with an Electrothermal (9100) device. KBr pellets were used to work with a Perkin Elmer 1430 spectrophotometer to record the IR spectra. Utilizing tetramethyl silane as an internal reference, ^1^H and ^13^C NMR spectra in deuterated dimethylsulfoxide were captured at 300 MHz and 500 MHz using a Varian Gemini NMR spectrometer then the data were predicted as values. At 70 eV, using a Shimadzu GCMS-QP 1000 Ex mass spectrometer, mass spectra were extracted. The spectral, elemental analyses and the antibacterial analyses were obtained out at the Microanalyses Center at Cairo University, Giza, Egypt. Enzyme inhibition was performed at VACSRA

**General procedure for synthesis of 2a-f.**

In a round flask, 2-aminothiophenol (0.01 mol) reacted with 2-cyano-*N*-arylacetamides **1a-f** (0.01 mol) in absolute ethanol (10 ml) containing a catalytic amount of piperidine (1 ml) under reflux for 7 hours. The obtained products were collected through filtration and recrystallized from dioxane.

**2-(Benzo[*d*]thiazol-2-yl)-*N*-phenylacetamide** (**2a**)

Yellow crystals, m.p: 180-182 ^o^C, yield (71%); *v*_max_ / cm^-1^ = 3281 (NH), 1655 (CO); δ^1^_H_ = 4.29 (s, 2H, CH_2_), 7.07-7.10 (m, 1H, ArH), 7.30-7.33 (m, 2H, ArH), 7.39-7.42 (m, 1H, ArH), 7.49-7.52 (m, 1H, ArH), 7.61 (d, 2H, *J* = 7.5 Hz, ArH), 7.96-7.98 (m, 1H, ArH), 8.05-8.08 (m, 1H, ArH), 10.43 (s, 1H, NH); δ^13^_C_ = 41.7, 119.3, 122.0, 122.3, 123.7, 125.0, 126.1, 128.9, 135.3, 138.8, 152.3, 165.0, 166.1; m/z = 268 (M^+^, 65.3%), 252 (73.4%), 242 (71.4%), 229 (47.0%), 211 (90.1%), 178 (88.6%), 176 (100%), 159 (73.9%), 144 (55.6%), 140 (56.0%), 117 (75.1%), 106 (67.8%), 96 (85.3%), 80 (82.2%), 74 (46.1%), 56 (48.1%), 49 (58.9%); Elemental anal. For C_15_H_12_N_2_OS (268.33): Calc. = C, 67.14; H, 4.51; N, 10.44; S, 11.95%; Found = C, 67.32; H, 4.35; N, 10.70; S, 11.77%.

**2-(Benzo[*d*]thiazol-2-yl)-*N*-(*p*-tolyl)acetamide** (**2b**)

Green crystals, m.p: 160-162 ^o^C, yield (67%); *v*_max_ / cm^-1^ = 3278 (NH), 1658 (CO); δ^1^_H_ = 2.25 (s, 3H, CH_3_), 4.27 (s, 2H, CH_2_), 7.11 (d, 2H, *J* = 8.4 Hz, ArH), 7.39-7.52 (m, 4H, ArH), 7.95 (d, 1H, *J* = 7.8 Hz, ArH), 8.06 (d, 1H, *J* = 7.5 Hz, ArH), 10.33 (s, 1H, NH); m/z = 283 (M^+^, 100%), 282 (44.4%), 267 (0.68%), 235 (0.17%), 194 (0.16%), 149 (25.4%), 148 (9.4%), 70 (0.24%), 45 (0.25%); Elemental anal. For C_16_H_14_N_2_OS (282.36): Calc. = C, 68.06; H, 5.00; N, 9.92; S, 11.35%; Found = C, 68.22; H, 5.18; N, 9.69; S, 11.19%.

**2-(Benzo[*d*]thiazol-2-yl)-*N*-(4-chlorophenyl)acetamide (2c)**

Yellowish green crystals, m.p: 165-167 ^o^C, yield (63%); *v*_max_ / cm^-1^ = 3274 NH), 1658 (CO); δ^1^_H_ = 4.30 (s, 2H, CH_2_), 7.36 (d, 2H, *J* = 8.4 Hz, ArH), 7.39-7.64 (m, 4H, ArH), 7.69 (d, 1H, *J* = 8.1 Hz, ArH), 8.05 (d, 1H, *J* = 7.8 Hz, ArH), 10.57 (s, 1H, NH); m/z = 303 (M^+^+1, 100%), 302 (M^+^, 41.2%), 300 (0.18%), 284 (0.72%), 280 (0.17%), 256 (0.15%), 209 (0.18%), 204 (0.16%), 197 (0.15%), 175 (0.18%), 149 (1.3%), 148 (3.9%), 126 (0.11%), 110 (0.37%), 106 (0.19%), 90 (0.12%); Elemental anal. For C_15_H_11_ClN_2_OS (302.78): Calc. = C, 59.50; H, 3.66; Cl, 11.71; N, 9.25; S, 10.59%; Found = C, 59.68; H, 3.49; N, 9.51; S, 10.41%.

**2-(Benzo[*d*]thiazol-2-yl)-*N*-(2,4-dichlorophenyl)acetamide (2d)**

Brown crystals, m.p: 210-212 ^o^C, yield (62%); *v*_max_ / cm^-1^ = 3434 (NH), 1636 (CO); δ^1^_H_ = 4.32 (s, 2H, CH_2_), 7.28 (d, 2H, *J* = 8.1 Hz, ArH), 7.48-7.56 (m, 2H, ArH), 7.91 (s, 1H, ArH), 8.03 (d, 1H, *J* = 8.1 Hz, ArH), 8.13 (d, 1H, *J* = 8.1 Hz, ArH), 10.64 (s, 1H, NH); m/z = 337 (M^+^, 51.3%), 335 (62.4%), 318 (75.4%), 306 (31.0%), 291 (28.9%), 249 (49.6%), 241 (100%), 234 (41.8%), 205 (63.1%), 203 (64.7%), 190 (66.0%), 170 (50.6%), 155 (47.0%), 131 (24.7%), 91 (40.9%), 80 (25.5%), 46 (46.1%); Elemental anal. For C_15_H_10_Cl_2_N_2_OS (337.22): Calc. = C, 53.43; H, 2.99; Cl, 21.02; N, 8.31; S, 9.51%; Found = C, 53.59; H, 3.15; N, 8.05; S, 9.35%.

**4-(2-(Benzo[*d*]thiazol-2-yl)acetamido)benzoic acid (2e)**

White crystals, m.p: 290-292 ^o^C, yield (64%); *v*_max_ / cm^-1^ = 3427 (broad, OH); 3343 (NH),1691 (CO); 1671 (CO); δ^1^_H_ = 4.31 (s, 2H, CH_2_), 7.39-7.42 (m, 1H, ArH), 7.42-7.45 (m, 1H, ArH), 7.47-7.50 (m, 1H, ArH), 7.65 (d, 1H, *J* = 7.8 Hz, ArH), 7.82 (d, 1H, *J* = 8.1 Hz, ArH), 7.96 (d, 1H, *J* = 8.7 Hz, ArH), 8.06 (d, 1H, *J* = 8.4 Hz, ArH), 8.22 (s, 1H, ArH), 10.62 (s, 1H, NH), 11.89 (s, 1H, OH); δ^13^_C_ = 41.7, 120.0, 122.3, 123.4, 124.5, 125.1, 126.1, 129.2, 131.4, 135.3, 139.0, 152.3, 164.8, 166.4, 167.1; Elemental anal. For C_16_H_12_N_2_O_3_S (312.34): Calc. = C, 61.53; H, 3.87; N, 8.97; S, 10.26%; Found = C, 61.69; H, 3.70; N, 9.20; S, 10.08%.

**2-(Benzo[*d*]thiazol-2-yl)-*N*-(pyrimidin-2-yl)acetamide (2f)**

Brown crystals, m.p < 300 ^o^C, yield (67%); *v*_max_ / cm^-1^ = 3244 (NH), 1660 (CO); δ^1^_H_ = 4.39 (s, 2H, CH_2_), 7.18-7.23 (m, 1H, ArH), 7.43-7.52 (m, 3H, ArH), 8.02 (d, 1H, *J* = 7.8 Hz, ArH), 8.13 (d, 1H, *J* = 8.1 Hz, ArH), 8.43 (d, 1H, *J* = 8.1 Hz, ArH), 10.61 (s, 1H, NH); Elemental anal. For C_13_H_10_N_4_OS (270.31): Calc. = C, 57.76; H, 3.73; N, 20.73; S, 11.86%; Found = C, 57.94; H, 3.57; N, 20.96; S, 11.70%.

**General procedure for synthesis of 4a-o.**

**Method A:** A mixture of compounds **2a-c** (0.01 mol) and aromatic aldehydes **3a-f** (0.01 mol) was refluxed in absolute ethanol (10 ml) in the presence of catalytic amount of piperidine (1ml) for 4 hours. The formed solids were collected by filtration and washed with ethanol then recrystallized out of ethanol-dioxane mixture.

**Method B:** A mixture of compounds **2a-c** (0.01 mol) and aromatic aldehydes **3a-f** (0.01 mol) was grinded in the presence of *p*-tolyl sulfonic acid (0.01 mol) for 1 hour. The formed solids were collected by filtration and washed with ethanol then recrystallized out of the ethanol-dioxane mixture.

**2-(Benzo[*d*]thiazol-2-yl)-*N*,3-diphenylacrylamide** (**4a**)

Yellow crystals, m.p: 225-227 ^o^C, yield (Method A; 69%, Method B; 76%); *v*_max_ / cm^-1^ = 3234 (NH), 1649 (CO); δ^1^_H_ = 7.10-7.15 (m, 2H, ArH), 7.30-7.41 (m, 3H, ArH), 7.43 (d, 2H, *J* = 7.5 Hz, ArH), 7.48-7.55 (m, 3H, ArH), 7.69-7.71 (m, 3H, ArH and =CH), 8.01 (d, 1H, *J* = 7.8 Hz, ArH), 8.10 (d, 1H, *J* = 8.1 Hz, ArH), 10.76 (s, 1H, NH); δ^13^_C_ = 119.6, 122.2, 122.8, 124.0, 125.8, 126.7, 128.8, 128.9, 129.2, 129.7, 132.5, 133.9, 134.1, 138.8, 153.1, 164.7, 166.0; Elemental anal. For C_22_H_16_N_2_OS (356.44): Calc. = C, 74.13; H, 4.52; N, 7.86; S, 8.99%; Found = C, 74.29; H, 4.32; N, 8.12; S, 8.77%.

**2-(Benzo[*d*]thiazol-2-yl)-3-(4-methoxyphenyl)-*N*-phenylacrylamide** (**4b**)

Yellow crystals, m.p: 220-222 ^o^C, yield (Method A; 76%, Method B; 83%); *v*_max_ / cm^-1^ = 3434 (NH), 1659 (CO); δ^1^_H_ = 3.75 (s, 3H, OCH_3_), 6.96 (d, 2H, *J* = 8.7 Hz, ArH), 7.12-7.17 (m, 1H, ArH), 7.40-7.56 (m, 5H, ArH and =CH), 7.66-7.71 (m, 2H, ArH), 7.77-7.83 (m, 2H, , ArH), 8.01 (d, 1H, *J* = 8.1 Hz, ArH), 8.07 (d, 1H, *J* = 7.8 Hz, ArH), 10.86 (s, 1H, NH); δ^13^_C_ = 55.2, 114.0, 119.6, 122.0, 122.6, 124.0, 126.3, 126.5, 128.6, 128.9, 130.2, 131.1, 133.8, 133.9, 139.0, 153.3, 160.5, 165.1, 166.4; Elemental anal. For C_23_H_18_N_2_O_2_S (386.47): Calc. = C, 71.48; H, 4.69; N, 7.25; S, 8.30%; Found = C, 71.66; H, 4.49; N, 7.51; S, 8.13%.

**2-(Benzo[*d*]thiazol-2-yl)-3-(3,4-dimethoxyphenyl)-*N*-phenylacrylamide** (**4c**).

Yellow crystals, m.p: 240-242 ^o^C, yield (Method A; 73%, Method B; 81%); *v*_max_ / cm^-1^ = 3162 (NH), 1655 (CO); δ^1^_H_ = 3.57 (s, 3H, OCH_3_), 3.78 (s, 3H, OCH_3_), 7.00 (d, 1H, *J* = 8.8 Hz, ArH), 7.12-7.16 (m, 1H, ArH), 7.31-7.54 (m, 6H, ArH), 7.62 (s, 1H, =CH), 7.77 (d, 2H, *J* = 8.4 Hz, ArH), 7.99 (d, 1H, *J* = 8.0 Hz, ArH), 8.10 (d, 1H, *J* = 7.6 Hz, ArH), 10.82 (s, 1H, NH); δ^13^_C_ = 55.6, 56.0, 112.2, 119.8, 122.5, 123.1, 124.2, 124.4, 126.0, 126.9, 127.0, 129.4, 130.7, 134.4, 134.5, 139.5, 149.0, 150.8, 153.7, 165.6, 166.8; m/z = 416 (61.8%), 407 (2.7%), 377 (59.1%), 363 (8.0%), 293 (2.8%), 289 (3.0%), 262 (68.3%), 241 (68.0%), 227 (6.3%), 191 (47.8%), 166 (6.5%), 102 (100%), 96 (65.4%), 76 (2.1%), 57 (57.5%). Elemental anal. For C_24_H_20_N_2_O_3_S (416.50): Calc. = C, 69.21; H, 4.84; N, 6.73; S, 7.70%; Found = C, 69.40; H, 4.68; N, 6.50; S, 7.85%.

**2-(Benzo[*d*]thiazol-2-yl)-3-(4-chlorophenyl)-*N*-phenylacrylamide** (**4d**)

Yellow crystals, m.p: 210-212 ^o^C, yield (Method A; 64%, Method B; 72%); *v*_max_ / cm^-1^ = 3434 (NH), 1660 (CO); δ^1^_H_ = 7.14-7.16 (m, 1H, ArH), 7.35-7.52 (m, 7H, ArH), 7.70-7.76 (m, 4H, ArH), 8.30 (d, 1H, *J* = 8.4 Hz, ArH), 8.09 (d, 1H, *J* = 7.8 Hz, ArH), 10.67 (s, 1H, NH); δ^13^_C_ = 66.3, 119.7, 122.1, 122.9, 124.1, 125.8, 126.7, 128.9, 130.8, 132.6, 132.8, 133.1, 134.2, 134.3, 138.7, 153.1, 164.5, 165.7; Elemental anal. For C_22_H_15_ClN_2_OS (390.89): Calc. = C, 67.60; H, 3.87; Cl, 9.07; N, 7.17; S, 8.20%; Found = C, 67.76; H, 3.68; N, 7.41; S, 8.04%.

**2-(Benzo[*d*]thiazol-2-yl)-3-phenyl-*N*-(*p*-tolyl)acrylamide** (**4e**)

Brown crystals, m.p: 210-212 ^o^C, yield (Method A; 91%, Method B; 96%); *v*_max_ / cm^-1^ = 3223 (NH), 1664 (CO); δ^1^_H_ = 2.29 (s, 3H, CH_3_), 7.17 (d, 2H. *J* = 7.5 Hz, ArH), 7.31-7.56 (m, 5H, ArH and =CH), 7.62 (d, 2H, *J* = 6.9 Hz, ArH), 7.70-7.72 (m, 3H, ArH), 8.03 (d, 1H, *J* = 7.8 Hz, ArH), 8.12 (d, 1H, *J* = 7.8 Hz, ArH), 10.71 (s, 1H, NH); δ^13^_C_ = 20.5, 119.6, 122.1, 122.8, 125.7, 126.3, 126.6, 128.5, 128.8, 129.0, 129.6, 133.0, 133.8, 133.9, 134.1, 136.3, 153.1, 164.5, 166.0; ; m/z = 370 (9.6%), 369 (38.3%), 368 (74.7%), 360 (1.2%), 293 (1.8%), 292 (3.0%), 278 (23.1%), 263 (8.5%), 260 (6.5%), 236 (16.1%), 235 (100%), 234 (33.8%), 207 (3.8%), 203 (5.8%), 195 (3.9%), 190 (4.2%), 182 (1.5%), 76 (17.4%). Elemental anal. For C_23_H_18_N_2_OS (370.47): Calc. = C, 74.57; H, 4.90; N, 7.56; S, 8.65%; Found = C, 74.75; H, 4.74; N, 7.33; S, 8.81%

**2-(Benzo[*d*]thiazol-2-yl)-3-(4-methoxyphenyl)-*N*-(*p*-tolyl)acrylamide** (**4f**)

Yellow crystals, m.p: 200-202 ^o^C, yield (Method A; 92%, Method B; 98%); *v*_max_ / cm^-1^ = 3432 (NH), 1662 (CO); δ^1^_H_ = 2.30 (s, 3H, CH_3_), 3.77 (s, 3H, OCH_3_), 6.97 (d, 2H, *J* = 8.8 Hz, ArH), 7.19 (d, 2H. *J* = 8.0 Hz, ArH), 7.42-7.53 (m, 2H, ArH), 7.65-7.69 (m, 5H, ArH and =CH), 8.01 (d, 1H, *J* = 8.0 Hz, ArH), 8.08 (d, 1H, *J* = 7.6 Hz, ArH), 10.72 (s, 1H, NH); δ^13^_C_ = 20.9, 55.7, 114.8, 120.1, 122.5, 123.1, 125.9, 126.8, 127.0, 129.7, 130.7, 131.6, 133.4, 134.1, 134.5, 136.9, 153.7, 160.9, 165.3, 166.9. Elemental anal. For C_24_H_20_N_2_O_2_S (400.50): Calc. = C, 71.98; H, 5.03; N, 6.99; S, 8.01%; Found = C, 71.83; H, 5.23; N, 6.73; S, 8.17%.

**2-(Benzo[*d*]thiazol-2-yl)-3-(3,4-dimethoxyphenyl)-*N*-(*p*-tolyl)acrylamide** (**4g**)

Yellow crystals, m.p: 240-242 ^o^C, yield (Method A; 90%, Method B; 97%); *v*_max_ / cm^-1^ = 3159 (NH), 1656 (CO); δ^1^_H_ = 2.29 (s, 3H, CH_3_), 3.58 (s, 3H, OCH_3_), 3.78 (s, 3H, OCH_3_), 6.99 (d, 1H, *J* = 8.4 Hz, ArH), 7.18 (d, 2H, *J* = 8.0 Hz, ArH), 7.31-7.35 (m, 2H, ArH), 7.42-7.54 (m, 2H, ArH), 7.63-7.07 (m, 3H, ArH and =CH), 8.00 (d, 1H, *J* = 8.0 Hz, ArH), 8.09 (d, 1H, *J* = 8.0 Hz, ArH), 10.75 (s, 1H, NH); δ^13^_C_ = 20.9, 55.6, 56.0, 112.1, 112.2, 119.8, 122.5, 123.1, 124.2, 125.9, 126.9, 127.0, 129.7, 130.8, 133.4, 134.4, 137.0, 149.0, 150.8, 153.7, 165.4, 166.9. Elemental anal. For C_25_H_22_N_2_O_3_S (430.52): Calc. = C, 69.75; H, 5.15; N, 6.51; S, 7.45%; Found = C, 69.95; H, 4.99; N, 6.77; S, 7.29%.

**2-(Benzo[*d*]thiazol-2-yl)-3-(4-chlorophenyl)-*N*-(*p*-tolyl)acrylamide** (**4h**)

Pale yellow crystals, m.p: 260-262 ^o^C, yield (Method A; 86%, Method B; 93%); *v*_max_ / cm^-1^ = 3286 (NH), 1651 (CO); δ^1^_H_ = 2.29 (s, 3H, CH_3_), 7.16 (d, 2H, *J* = 8.4 Hz, ArH), 7.45-7.71 (m, 9H, ArH and =CH), 8.02 (d, 1H, *J* = 8.0 Hz, ArH), 8.12 (d, 1H, *J* = 8.0 Hz, ArH), 10.70 (s, 1H, NH); δ^13^_C_ = 20.9, 120.2, 122.6, 123.3, 126.3, 127.2, 129.3, 129.7, 131.3, 132.9, 133.3, 133.5, 133.6, 134.6, 134.7, 136.6, 153.6, 164.7, 166.2; Elemental anal. For C_23_H_17_ClN_2_OS (404.91): Calc. = C, 68.23; H, 4.23; Cl, 8.75; N, 6.92; S, 7.92%; Found = C, 68.39; H, 4.07; N, 7.19; S, 7.72%.

**2-(Benzo[*d*]thiazol-2-yl)-*N*-(4-chlorophenyl)-3-phenylacrylamide** (**4i**)

Yellow crystals, m.p: 185-186 ^o^C, yield (Method A; 83%, Method B; 88%); *v*_max_ / cm^-1^ = 3295 (NH), 1659 (CO); δ^1^_H_ = 7.41 (d, 2H, *J* = 7.5 Hz, ArH), 7.47-7.56 (m, 8H, ArH and =CH), 7.82 (d, 2H, *J* = 8.1 Hz, ArH), 8.01 (d, 1H, *J* = 7.8 Hz, ArH), 8.13 (d, 1H, *J* = 7.8 Hz, ArH), 10.73 (s, 1H, NH); δ^13^_C_ = 121.7, 122.6, 123.4, 126.3, 127.1, 128.2, 129.0, 129.3, 129.7, 130.2, 132.8, 134.2, 134.6, 134.7, 138.2, 153.7, 165.4, 166.4; Elemental anal. For C_22_H_15_ClN_2_OS (390.89): Calc. = C, 67.60; H, 3.87; Cl, 9.07; N, 7.17; S, 8.20%; Found = C, 67.45; H, 3.93; N, 7.40; S, 8.02%.

**2-(Benzo[*d*]thiazol-2-yl)-*N*-(4-chlorophenyl)-3-(4-methoxyphenyl)acrylamide** (**4j**)

Yellow crystals, m.p: 215-217 ^o^C, yield (Method A; 68%, Method B; 74%); *v*_max_ / cm^-1^ = 3222 (NH), 1666 (CO); δ^1^_H_ = 3.76 (s, 3H, OCH_3_), 6.96 (d, 2H, *J* = 8.7 Hz, ArH), 7.38-7.53 (m, 4H, ArH and =CH), 7.62-7.65 (m, 3H, ArH), 7.73 (d, 2H, *J* = 6.9 Hz, ArH), 7.97 (d, 1H, *J* = 8.1 Hz, ArH), 8.07 (d, 1H, *J* = 7.5 Hz, ArH), 10.89 (s, 1H, NH); δ^13^_C_ = 55.3, 114.4, 121.1, 121.8, 122.1, 122.3, 122.7, 123.1, 125.6, 126.2, 126.6, 127.6, 128.5, 128.9, 131.1, 134.0, 137.9, 160.5, 165.2; Elemental anal. For C_23_H_17_ClN_2_O_2_S (420.91): Calc. = C, 65.63; H, 4.07; Cl, 8.42; N, 6.66; S, 7.62%; Found = C, 65.81; H, 3.91; N, 6.92; S, 7.44%.

**2-(Benzo[*d*]thiazol-2-yl)-*N*-(4-chlorophenyl)-3-(3,4-dimethoxyphenyl)acrylamide** (**4k**)

Yellow crystals, m.p: 245-247 ^o^C, yield (Method A; 65%, Method B; 72%); *v*_max_ / cm^-1^ = 3295 (NH), 1673 (CO); δ^1^_H_ = 3.57 (s, 3H, OCH_3_), 3.78 (s, 3H, OCH_3_), 7.01 (d, 1H, *J* = 8.8 Hz, ArH), 7.29-7.30 (m, 2H, ArH), 7.43-7.53 (m, 4H, ArH), 7.62 (s, 1H, =CH), 7.78 (d, 2H, *J* = 8.8 Hz, ArH), 7.98 (d, 1H, *J* = 8.0 Hz, ArH), 8.10 (d, 1H, *J* = 8.0 Hz, ArH), 10.95 (s, 1H, NH); δ^13^_C_ = 55.6, 56.0, 112.2, 121.3, 122.5, 123.1, 124.2, 126.0, 126.7, 127.1, 128.0, 129.3, 130.5, 134.3, 134.7, 138.4, 149.0, 150.8, 153.7, 165.7, 166.7; Elemental anal. For C_24_H_19_ClN_2_O_3_S (450.94): Calc. = C, 63.93; H, 4.25; Cl, 7.86; N, 6.21; S, 7.11%; Found = C, 63.75; H, 4.45; N, 5.98; S, 7.27%.

**2-(Benzo[*d*]thiazol-2-yl)-*N*,3-bis(4-chlorophenyl)acrylamide** (**4l**)

Orange crystals, m.p: 244-246 ^o^C, yield (Method A; 72%, Method B; 78%); *v*_max_ / cm^-1^ = 3295 (NH), 1653 (CO); δ^1^_H_ = 7.37-7.55 (m, 6H, ArH and =CH), 7.66-7.72 (m, 5H, ArH), 8.00 (d, 1H, *J* = 7.8 Hz, ArH), 8.11 (d, 1H, *J* = 7.8 Hz, ArH), 10.91 (s, 1H, NH); δ^13^_C_ = 121.2, 122.2, 122.9, 125.9, 126.8, 127.8, 128.9, 129.0, 130.8, 132.7, 132.8, 132.9, 134.1, 134.4, 137.6, 153.1, 164.6; ; m/z = 425 (1.6%), 424 (38.5%), 422 (2.3%), 408 (30.8%), 402 (33.1%), 399 (16.5%), 388 (8.6%), 384 (27.1%), 378 (32.8%), 375 (29.0%), 362 (46.7%), 359 (41.5%), 355 (31.2%), 351 (37.4%), 344 (88.4%), 332 (48.9%), 316 (32.0%), 305 (39.4%), 297 (62.3%), 284 (24.4%), 269 (34.1%), 220 (26.1%), 198 (62.1%), 157 (100%), 139 (29.4%), 126 (48.3%), 97 (61.9%), 79 (59.2%). Elemental anal. For C_22_H_14_Cl_2_N_2_OS (425.33): Calc. = C, 62.13; H, 3.32; Cl, 16.67; N, 6.59; S, 7.54%; Found = C, 62.33; H, 3.17; N, 6.82; S, 7.38%

**4-(2-(Benzo[*d*]thiazol-2-yl)-3-oxo-3-(phenylamino)prop-1-en-1-yl)phenylbenzoate** (**4m**)

Deep yellow crystals, m.p: 245-247 ^o^C, yield (Method A; 79%, Method B; 84%); *v*_max_ / cm^-1^ = 3448 (NH), 1735 (CO), 1665 (CO); δ^1^_H_ = 7.12-7.17 (m, 1H, ArH), 7.36-7.54 (m, 5H, ArH), 7.55-7.81 (m, 9H, ArH and =CH), 8.02 (d, 2H, *J* = 7.8 Hz, ArH), 8.11-8.14 (m, 2H, ArH), 10.81 (s, 1H, NH); δ^13^_C_ = 119.6, 122.2, 122.6, 122.9, 124.2, 125.9, 126.8, 128.7, 129.0, 129.8, 130.5, 131.4, 131.8, 132.6, 133.0, 134.1, 134.2, 138.7, 151.5, 153.2, 164.4, 164.7, 166.0; Elemental anal. For C_29_H_20_N_2_O_3_S (476.55): Calc. = C, 73.09; H, 4.23; N, 5.88; S, 6.73%; Found = C, 73.25; H, 4.39; N, 5.65; S, 6.55%.

**4-(2-(Benzo[*d*]thiazol-2-yl)-3-oxo-3-(*p*-tolylamino)prop-1-en-1-yl)phenylbenzoate** (**4n**)

Pale yellow crystals, m.p: 262-264 ^o^C, yield (Method A; 81%, Method B; 87%); *v*_max_ / cm^-1^ = 3441 (NH), 1768 (CO); 1651 (CO); δ^1^_H_ = 2.27 (s, 3H, CH_3_), 6.81 (d, 2H, *J* = 8.7 Hz, ArH), 7.17 (d, 2H, *J* = 8.4 Hz, ArH), 7.39-7.50 (m, 8H, ArH and =CH), 7.53 (d, 2H, *J* = 8.1 Hz, ArH), 7.63 (d, 2H, *J* = 8.1 Hz, ArH), 7.98 (d, 1H, *J* = 8.1 Hz, ArH), 8.05 (d, 1H, *J* = 7.8 Hz, ArH), 10.64 (s, 1H, NH); δ^13^_C_ = 20.6, 115.9, 119.3, 119.6, 122.1, 122.6, 124.9, 125.5, 126.6, 129.3, 129.4, 131.5, 133.0, 134.0, 134.2, 136.6, 153.3, 159.3, 165.1, 166.7; Elemental anal. For C_30_H_22_N_2_O_3_S (490.58): Calc. = C, 73.45; H, 4.52; N, 5.71; S, 6.54%; Found = C, 73.61; H, 4.68; N, 5.48; S, 6.38%.

**4-(2-(Benzo[*d*]thiazol-2-yl)-3-((4-chlorophenyl)amino)-3-oxoprop-1-en-1-yl)phenyl-benzoate (4o)**

Brown crystals, m.p: 240-242 ^o^C, yield (Method A; 62%, Method B; 68%); *v*_max_ / cm^-1^ = 3477 (NH), 1751 (CO); 1665 (CO); δ^1^_H_ = 7.43 (d, 2H, *J* = 8.4 Hz, ArH), 7.53-7.68 (m, 9H, ArH and =CH), 7.81 (d, 2H, *J* = 8.1 Hz, ArH), 8.03 (d, 2H, *J* = 8.1 Hz, ArH), 8.12-8.21 (m, 3H, ArH), 10.74 (s, 1H, NH); Elemental anal. For C_29_H_19_ClN_2_O_3_S (510.99): Calc. = C, 68.17; H, 3.75; Cl, 6.94; N, 5.48; S, 6.27%; Found = C, 68.33; H, 3.91; N, 5.30; S, 6.00%.

**General procedure of 6a-i.**

**Method A**: To a solution of absolute ethanol (10 ml) in the presence of a few amount of piperidine (1ml), compounds **2a-c** (0.01 mol) and aryl pyrazole-2-aldehydes **5a-c** (0.01 mol) were added, then the reaction mixture was heated to reflux for 5 hours. The formed products were collected by filtration and washed with ethanol then recrystallized from ethanol-dioxane mixture.

**Method B:** A mixture of compounds **2a-c** (0.01 mol) and aryl pyrazole-2-aldehydes **5a-c** (0.01 mol) was grinded in the presence of *p*-tolyl sulfonic acid (0.01 mol) for 1 hour. The formed products were collected by filtration and washed with ethanol then recrystallized from ethanol-dioxane mixture.

**2-(Benzo[*d*]thiazol-2-yl)-3-(1,3-diphenyl-1*H*-pyrazol-4-yl)-*N*-phenylacrylamide (6a)**

Yellow crystals, m.p: 250-252 ^o^C, yield (Method A; 96%, Method B; 99%); *ν*_max_ / cm^-1^ = 3436 (NH), 1670 (CO); δ^1^_H_ = 7.16-7.18 (m, 1H, ArH), 7.37-7.78 (m, 17H, ArH), 7.99 (d, 1H, *J* = 8.1 Hz, ArH), 8.08 (d, 1H, *J* = 7.5 Hz, ArH), 8.41 (s, 1H, py-H), 10.81 (s, 1H, NH); δ^13^_C_ = 115.5, 118.7, 119.5, 122.1, 122.7, 124.1, 124.4, 125.6, 126.7, 127.1, 127.3, 128.6, 128.9, 129.0, 129.7, 131.4, 131.6, 133.9, 138.7, 138.8, 153.0, 153.1, 164.7, 165.3 ; m/z = 498 (M^+^, 32.5%), 474 (41.8%), 466 (44.5%), 431 (39.8%), 415 (16.4%), 391 (24.3%), 363 (28.8%), 350 (52.81%), 347 (38.9%), 320 (66.88%), 285 (22.8%), 272 (44.8%), 253 (45.27%), 202 (59.7%), 188 (100%), 184 (59.7%), 159 (79.5%), 139 (46.7%), 91 (98.0%), 71 (74.4%), 52 (69.0%); Elemental anal. For C_31_H_22_N_4_OS (498.60): Calc. = C, 74.68; H, 4.45; N, 11.24; S, 6.43%; Found = C, 74.48; H, 4.61; N, 11.47; S, 6.23%.

**2-(Benzo[*d*]thiazol-2-yl)-*N*-phenyl-3-(1-phenyl-3-(*p*-tolyl)-1*H*-pyrazol-4-yl)acrylamide (6b)**

Yellow crystals, m.p: 244-246 ^o^C, yield (Method A; 70%, Method B; 76%); *ν*_max_ / cm^-1^ = 3239 (NH), 1671 (CO); δ^1^_H_ = 2.42 (s, 3H, CH_3_), 7.13-7.18 (m, 1H, ArH), 7.32-7.37 (m, 3H, ArH), 7.40 (d, 2H, *J* = 7.8 Hz, ArH), 7.44-7.51 (m, 7H, ArH), 7.52 (s, 1H, =CH), 7.61 (d, 2H, *J* = 8.4 Hz, ArH), 7.75 (d, 2H, *J* = 7.5 Hz, ArH), 7.99 (d, 1H, *J* = 7.8 Hz, ArH), 8.08 (d, 1H, *J* = 7.8 Hz, ArH), 8.39 (s, 1H, pyH), 10.80 (s, 1H, NH); δ^13^_C_ = 20.8, 115.4, 118.7, 119.5, 122.1, 122.7, 124.1, 124.5, 125.6, 126.6, 127.0, 127.2, 128.5, 128.8, 129.0, 129.4, 129.7, 131.3, 133.9, 138.4, 138.7, 138.8, 153.1, 153.2, 164.7, 165.3 ; m/z = 512 (M^+^, 55.6%), 499 (74.7%), 462 (100%), 432 (69.5%), 420 (97.3%), 392 (32.4%), 370 (63.5%), 356 (68.1%), 342 (80.5%), 314 (76.8%), 292 (35.0%), 255 (48.8%), 253 (59.3%), 233 (87.3%), 190 (63.9%), 184 (64.5%), 162 (65.3%), 148 (68.0%), 134 (60.4%), 65 (51.3%), 47 (82.0%); Elemental anal. For C_32_H_24_N_4_OS (512.63): Calc. = C, 74.98; H, 4.72; N, 10.93; S, 6.25%; Found = C, 74.82; H, 4.52; N, 11.18; S, 6.41%.

**2-(Benzo[*d*]thiazol-2-yl)-3-(3-(4-chlorophenyl)-1-phenyl-1*H*-pyrazol-4-yl)-*N*-phenyl-acrylamide (6c)**

Deep yellow crystals, m.p: 251-253 ^o^C, yield (Method A; 68%, Method B; 75%); *ν*_max_ / cm^-1^ = 3419 (NH), 1679 (CO); δ^1^_H_ = 7.14-7.17 (m, 1H, ArH), 7.33-7.54 (m, 8H, ArH), 7.63-7.68 (m, 4H, ArH), 7.73-7.80 (m, 4H, ArH), 8.02 (d, 1H, *J* = 8.0 Hz, ArH), 8.09 (d, 1H, *J* = 7.6 Hz, ArH), 8.43 (s, 1H, pyH), 10.82 (s, 1H, NH); δ^13^_C_ = 116.1, 119.3, 120.0, 122.6, 123.3, 124.6, 124.7, 126.2, 127.1, 127.8, 129.2, 129.4, 129.5, 130.2, 130.7, 131.0, 132.3, 134.2, 134.5, 139.1, 139.3, 152.2, 153.6, 165.1, 165.7; Elemental anal. For C_31_H_21_ClN_4_OS (533.05): Calc. = C, 69.85; H, 3.97; Cl, 6.65; N, 10.51; S, 6.01%; Found = C, 72.89; H, 4.74; N, 10.37; S, 5.90%.

**2-(Benzo[*d*]thiazol-2-yl)-3-(1,3-diphenyl-1*H*-pyrazol-4-yl)-*N*-(*p*-tolyl)acrylamide (6d)**

Yellow crystals, m.p: 235-237 ^o^C, yield (Method A; 60%, Method B; 67%); *ν*_max_ / cm^-1^ = 3439 (NH), 1667 (CO); δ^1^_H_ = 2.29 (s, 3H, CH_3_), 7.19 (d, 2H, *J* = 8.1 Hz, ArH), 7.36 (d, 2H, *J* = 7.2 Hz, ArH), 7.44 (d, 2H, *J* = 6.9 Hz, ArH), 7.49-7.52 (m, 1H, ArH), 7.53 (s, 1H, =CH), 7.54-7.56 (m, 2H, ArH), 7.58 (d, 2H, *J* = 7.5 Hz, ArH), 7.62-7.65 (m, 2H, ArH), 7.75 (d, 2H, *J* = 6.6 Hz, ArH), 7.78 (s, 1H, CH), 7.98 (d, 1H, *J* = 7.8 Hz, ArH), 8.08 (d, 1H, *J* = 7.5 Hz, ArH), 8.39 (s, 1H, pyH), 10.71 (s, 1H, NH); δ^13^_C_ = 20.56, 115.6, 118.8, 119.6, 120.2, 122.2, 122.8, 124.3, 125.7, 126.7, 127.1, 127.4, 128.4, 128.8, 128.9, 129.1, 129.4, 129.8, 131.5, 131.7, 133.3, 133.9, 136.4, 138.7, 153.2, 164.5; Elemental anal. For C_32_H_24_N_4_OS (512.63): Calc. = C, 74.98; H, 4.72; N, 10.93; S, 6.25%; Found = C, 74.78; H, 4.91; N, 10.69; S, 6.44%.

**2-(Benzo[*d*]thiazol-2-yl)-3-(1-phenyl-3-(*p*-tolyl)-1*H*-pyrazol-4-yl)-*N*-(*p*-tolyl)acrylamide (6e)**

Yellow crystals, m.p: 255-257^o^C, yield (Method A; 85%, Method B; 92%); *ν*_max_ / cm^-1^ = 3434 (NH), 1669 (CO); δ^1^_H_ = 2.28 (s, 3H, CH_3_), 2.43 (s, 3H, CH_3_), 7.19 (d, 2H, *J* = 8.7 Hz, ArH), 7.36 (d, 2H, *J* = 7.5 Hz, ArH), 7.40 (d, 2H, *J* = 7.8 Hz, ArH), 7.44 (d, 2H, *J* = 6.0 Hz, ArH), 7.47 (d, 2H, *J* = 6.3 Hz, ArH), 7.61-7.63 (m, 5H, ArH), 7.66 (s, 1H, =CH), 7.98 (d, 1H, *J* = 7.8 Hz, ArH), 8.08 (d, 1H, *J* = 6.9 Hz, ArH), 8.37 (s, 1H, pyH), 10.70 (s, 1H, NH) ; δ^13^_C_ = 20.5, 115.5, 118.7, 119.5, 122.2, 122.7, 124.4, 125.6, 126.7, 127.0, 127.3, 128.5, 128.8, 129.1, 129.4, 129.5, 129.8, 131.3, 133.2, 133.9, 136.4, 138.4, 138.7, 153.1, 153.2, 164.5, 165.4 ; m/z = 526 (M^+^, 41.4%), 515 (100%), 503 (39.8%), 453 (65.7%), 437 (83.1%), 430 (62.8%), 416 (55.8%), 384 (47.5%), 356 (34.8%), 327 (37.6%), 315 (41.0%), 292 (34.2%), 279 (31.4%), 248 (55.0%), 165 (33.8%), 139 (88.8%), 113 (97.5%), 89 (57.2%), 70 (41.4%), 42 (39.6%); Elemental anal. For C_33_H_26_N_4_OS (526.66): Calc. = C, 75.26; H, 4.98; N, 10.64; S, 6.09%; Found = C, 75.06; H, 5.14; N, 10.44; S, 6.33%.

**2-(Benzo[*d*]thiazol-2-yl)-3-(3-(4-chlorophenyl)-1-phenyl-1*H*-pyrazol-4-yl)-*N*-(*p*-tolyl)-acrylamide (6f)**

Yellow crystals, m.p: 255-257 ^o^C, yield (Method A; 78%, Method B; 84%); *ν*_max_ / cm^-1^ = 3236 (NH), 1667 (CO); δ^1^_H_ = 2.28 (s, 3H, CH_3_), 7.18 (d, 2H, *J* = 7.2 Hz, ArH), 7.34-7.36 (m, 2H, ArH), 7.47-7.51 (m, 4H, ArH), 7.62-7.63 (m, 6H, ArH), 7.77-7.79 (d, 2H, ArH), 8.00 (d, 1H, *J* = 7.6 Hz, ArH), 8.07 (d, 1H, *J* = 7.6 Hz, ArH), 8.40 (s, 1H, pyH), 10.74 (s, 1H, NH); δ^13^_C_ = 20.9, 116.1, 119.2, 120.0, 122.6, 123.2, 124.6, 126.1, 127.1, 127.8, 129.4, 129.8, 130.2, 130.7, 132.3, 133.7, 134.2, 134.5, 136.8, 139.1, 152.2, 153.6, 164.9, 165.7; Elemental anal. For C_32_H_23_ClN_4_OS (547.07): Calc. = C, 70.26; H, 4.24; Cl, 6.48; N, 10.24; S, 5.86%; Found = C, 70.46; H, 4.04; N, 10.47; S, 6.02%.

**2-(Benzo[*d*]thiazol-2-yl)-*N*-(4-chlorophenyl)-3-(1,3-diphenyl-1*H*-pyrazol-4-yl)acrylamide (6g)**

Yellow crystals, m.p: 235-255 ^o^C, yield (Method A; 70%, Method B; 76%); *ν*_max_ / cm^-1^ = 3433 (NH), 1670 (CO); δ^1^_H_ = 6.92 (d, 2H, *J* = 8.4 Hz, ArH), 7.28-7.80 (m, 13H, ArH and =CH), 7.86 (d, 2H, *J* = 8.1 Hz, ArH), 8.06 (d, 1H, *J* = 7.8 Hz, ArH), 8.15 (d, 1H, *J* = 7.8 Hz, ArH), 8.31 (s, 1H, pyH), 10.81 (s, 1H, NH); δ^13^_C_ = 115.4, 118.8, 121.1, 122.2, 122.8, 124.8, 125.7, 126.7, 127.2, 127.4, 127.8, 128.6, 128.9, 129.0, 129.8, 131.2, 131.6, 133.9, 137.8, 138.7, 153.0, 153.1, 164.8, 165.2; m/z = 534 (M^+^+1, 3.1%), 531 (7.0%), 518 (8.0%), 478 (3.6%), 437 (100%), 420 (3.9%), 383 (0.7%), 367 (5.2%), 309 (7.8%), 299 (12.9%), 277 (12.4%), 246 (8.3%), 207 (8.9%), 206 (2.9%), 186 (4.7%), 165 (4.6%), 96 (5.9%), 76 (10.8%), 70 (7.1%), 56 (4.6%); Elemental anal. For C_31_H_21_ClN_4_OS (533.05): Calc. = C, 69.85; H, 3.97; Cl, 6.65; N, 10.51; S, 6.01%; Found = C, 69.69; H, 4.17; N, 10.28; S, 6.18%.

**2-(Benzo[*d*]thiazol-2-yl)-*N*-(4-chlorophenyl)-3-(1-phenyl-3-(*p*-tolyl)-1*H*-pyrazol-4-yl)acrylamide (6h)**

Yellow crystals, m.p: 295-297 ^o^C, yield (Method A; 63%, Method B; 69%); *ν*_max_ / cm^-1^ = 3433 (NH), 1671 (CO); δ^1^_H_ = 2.42 (s, 3H, CH_3_), 7.36-7.40 (m, 3H, ArH), 7.39 (d, 2H, *J* = 8.7 Hz, ArH), 7.44-7.47 (m, 3H, ArH), 7.50 (d, 2H, *J* = 6.9 Hz, ArH), 7.63-7.66 (m, 3H, ArH), 7.67 (s, 1H, =CH), 7.77 (d, 2H, *J* = 8.7 Hz, ArH), 7.99 (d, 1H, *J* = 7.5 Hz, ArH), 8.09 (d, 1H, *J* = 7.2 Hz, ArH), 8.38 (s, 1H, pyH), 10.91 (s, 1H, NH); δ^13^_C_ = 20.9, 115.3, 118.8, 121.1, 122.2, 122.8, 124.9, 125.7, 126.7, 127.1, 127.3, 127.7, 128.5, 128.6, 128.7, 129.0, 129.5, 129.8, 131.0, 133.8, 137.8, 138.4, 138.7, 153.1, 164.8, 165.3; Elemental anal. For C_32_H_23_ClN_4_OS (547.07): Calc. = C, 70.26; H, 4.24; Cl, 6.48; N, 10.24; S, 5.86%; Found = C, 70.06; H, 4.42; N, 10.47; S, 5.70%

**2-(Benzo[*d*]thiazol-2-yl)-*N*-(4-chlorophenyl)-3-(3-(4-chlorophenyl)-1-phenyl-1*H*-pyrazol-4-yl)acrylamide(6i)**
Yellow crystals, m.p: 280-282 ^o^C, yield (Method A; 65%, Method B; 73%); *ν*_max_ / cm^-1^ = 3434 (NH), 1669 (CO); δ^1^_H_ = 7.37-7.43 (m, 4H, ArH), 7.45 (d, 2H, *J* = 7.8 Hz, ArH), 7.50-7.52 (m, 4H, ArH and =CH), 7.65 (d, 2H, *J* = 8.1 Hz, ArH), 7.74-7.79 (m, 4H, ArH), 8.00 (d, 1H, *J* = 7.8 Hz, ArH), 8.09 (d, 1H, *J* = 7.8 Hz, ArH), 8.41 (s, 1H, py-H), 10.89 (s, 1H, NH); δ^13^_C_ = 115.6, 119.0, 121.2, 122.3, 122.9, 124.7, 125.9, 126.8, 127.5, 127.8, 129.1, 129.9, 130.3, 130.6, 131.6, 133.8, 134.0, 137.8, 138.7, 151.8, 153.2, 164.8; m/z = 567 (16.0%), 552 (42.6%), 544 (46.2%), 530 (83.3%), 512 (100%), 432 (80.1%), 430 (85.5%), 424 (73.7%), 376 (61.4%), 352 (44.2%), 332 (98.4%), 330 (62.9%), 324 (60.1%), 296 (91.7%), 271 (86.9%), 267 (55.2%), 198 (45.1%), 146 (70.0%), 121 (63.2%), 86 (23.8%), 69 (43.7%), 55 (40.0%); Elemental anal. For C_31_H_20_Cl_2_N_4_OS (567.49): Calc. = C, 65.61; H, 3.55; Cl, 12.49; N, 9.87; S, 5.65%; Found = C, 65.41; H, 3.73; N, 9.64; S, 5.84%.

**Synthesis of 3-(benzo[*d*]thiazol-2-yl)-2*H*-chromen-2-one (8)**

A solution of **2a** (0.01 mol) in ethanol (10 ml) with a few drops of piperidine was added to salicylaldehyde (0.01 mol). The mixture was stirred for 4 hours. The solid product that precipitated by cooling was filtered and recrystallized from dioxane.

Yellow crystal, yield (70%); m.p: 220-222^°^C [46]; *v*_max_ / cm^-1^ = 1670 (CO); δ^1^_H_ = 7.25 (m, 2H, ArH), 7. 52-7.45 (m, 4H, ArH), 7.57 7.83 (m, 2H, ArH), 9.07 (s, 1H, ArH); Elemental anal. For C_16_H_9_NO_2_S (279.31) Calc. = C, 68.80; H, 3.25; N, 5.01; S, 11.48 %; Found: C, 68.76; H, 3.43; N, 5.21; S, 11.61 %.

**General procedure for synthesis of 10a-c.**

A solution of compound **2a** (0.01 mol) in ethanol (10 ml) containing a few drops of piperidine was added to the appropriate arylazo salicylaldehyde **2a-c** (0.01 mol). The mixture was refluxed for 4 hours. The solid product that precipitated by cooling was filtered and recrystallized from dioxane.

**3-(Benzo[*d*]thiazol-2-yl)-6-(phenyldiazenyl)-2*H*-chromen-2-one (10a)**

Orange crystals, yield (63%), m.p: 251-253^°^C, *v*_max_ / cm^-1^ = 1729 (CO), 1607 (N=N); δ^1^_H_ = 7.46 (d, 2H, *J* = 7.2 Hz, ArH), 7.68-7.74 (m, 5H, ArH and CH), 7.93 (d, 2H, *J* = 8.4 Hz, ArH), 8.06 (d, 2H, *J* = 8.4 Hz, ArH), 8.08 (d, 2H, *J* = 8.1 Hz, ArH); Elemental anal. For C_22_H_13_N_3_O_2_S (383.42) Calc. = C, 68.92; H, 3.42; N, 10.96; S, 8.36%; Found: C, 68.73; H, 3.61; N, 10.74; S, 8.54%.

**3-(Benzo[*d*]thiazol-2-yl)-6-(*p*-tolyldiazenyl)-2*H*-chromen-2-one (10b)**

Reddish brown crystals, yield (61%), m.p: 250-252^°^C, *v*_max_ / cm^-1^ = 1727 (CO), 1604 (N=N); δ^1^_H_ = 2.43 (s, 3H, CH_3_), 7.38 (d, 2H, *J* = 8.1 Hz, ArH), 7.63-7.79 (m, 4H, ArH and =CH), 7.89 (d, 2H, *J* = 7.5 Hz, ArH), 8.02-8.04 (m, 2H, ArH), 8.09-8.21 (m, 2H, ArH); Elemental anal. For C_23_H_15_N_3_O_2_S (397.45) Calc. = C, 69.51; H, 3.80; N, 10.57; S, 8.07%; Found: C, 69.32; H, 3.63; N, 10.79; S, 8.26%.

**3-(Benzo[*d*]thiazol-2-yl)-6-(4-chlorophenyldiazenyl)-2*H*-chromen-2-one (10c)**

Reddish brown crystals, yield (67%), m.p: 280-282 ^°^C, *v*_max_ / cm^-1^ = 1718 (CO), 1609 (N=N); δ^1^_H_ = 7.41 (d, 2H, *J* = 7.9 Hz, ArH), 7.45-7.60 (m, 3H, ArH), 7.88 (s, 1H, CH), 7.93 (d, 2H, *J* = 8.4 Hz, ArH), 8.0 -8.04 (m, 2H, ArH), 8.11-8.23 (m, 2H , ArH); Elemental anal. For C_22_H_12_ClN_3_O_2_S (417.86) Calc. = C, 63.24; H, 2.89; Cl, 8.48; N, 10.06; S, 7.67 %; Found: C, 63.43; H, 2.72; N, 10.30; S, 7.86%.

**General procedure for synthesis of 11a-f.**

**Method A**: The arylidene derivatives **4** (0.01 mol) were reacted with malononitrile (0.01 mol) in absolute ethanol (10 ml) in the presence of a catalytic amount of piperidine (1 ml) for 12 hours. The solid products were collected by filtration, washed with ethanol and recrystallized out of the mixture by using DMF.

**Method B:** The anilide derivatives **2a-c** (0.01 mol) were reacted with arylmethylene malononitrile derivatives **12a,b** (0.01 mol) in absolute ethanol (10 ml) in the presence of piperidine as a basic catalyst (1 ml) for 10 hours. The solid products were collected by filtration, washed with ethanol and recrystallized from DMF.

**2-Amino-5-(benzo[*d*]thiazol-2-yl)-6-oxo-1,4-diphenyl-1,6-dihydropyridine-3-carbonitrile** (**11a**)

Brown crystals, m.p: < 300 ^o^C, yield (83%); *ν*_max_ / cm^-1^ = 3448-3302 (broad, NH_2_), 2206 (CN), 1643 (CO); δ^1^_H_ = 7.21-7.28 (m, 5H, ArH and NH_2_), 7.33-7.34 (m, 2H, ArH), 7.37 (d, 2H, *J* = 8.0 Hz, ArH), 7.43 (d, 2H, *J* = 7.5 Hz, ArH), 7.52-7.55 (m, 3H, ArH), 7.57 (d, 1H, *J* = 7.5 Hz, ArH), 7.86 (d, 1H, *J* = 7.5 Hz, ArH); m/z = 420 (M^+^, 77.9%), 413 (37.2%), 397 (52.8%), 378 (58.3%), 354 (40.2%), 352 (73.4%), 349 (95.9%), 326 (71.9%), 315 (29.7%), 290 (39.9%), 285 (40.1%), 240 (66.4%), 219 (52.9%), 212 (77.9%), 206 (97.6%), 191 (74.7%), 141 (46.1%), 89 (100%), 55 (78.7%); Elemental anal. For C_25_H_16_N_4_OS (420.49) Calc. = C, 71.41; H, 3.84; N, 13.32; O, 3.80; S, 7.62 %; Found = C, 71.21; H, 4.00; N, 13.06; S, 7.78%.

**2-Amino-5-(benzo[*d*]thiazol-2-yl)-4-(4-methoxyphenyl)-6-oxo-1-phenyl-1,6-dihydro-pyridine-3-carbonitrile** (**11b**)

Brown crystals, m.p: 260-262 ^o^C, yield (60%); *ν*_max_ / cm^-1^ = 3358 (broad, NH_2_), 2185 (CN), 1714 (CO); δ^1^_H_ = 3.72 (s, 3H, OCH_3_), 6.91-6.97 (m, 4H, ArH and NH_2_), 7.22-7.30 (m, 3H, ArH), 7.33 (d, 2H, *J* = 7.2 Hz, ArH), 7.38 (d, 2H, *J* = 8.4 Hz, ArH), 7.44 (d, 2H, *J* = 7.8 Hz, ArH), 8.02 (d, 1H, *J* = 7.8 Hz, ArH), 8.11 (d, 1H, *J* = 8.1 Hz, ArH); δ^13^_C_ = 55.0, 66.4, 108.1, 114.0, 116.9, 121.4, 124.4, 125.5, 126.6, 128.0, 128.7, 129.2, 129.3, 131.6, 135.6, 151.8, 152.5, 157.3, 158.0, 158.8, 159.3, 166.6; Elemental anal. For C_26_H_18_N_4_O_2_S (450.52) Calc. = C, 69.32; H, 4.03; N, 12.44; S, 7.12%; Found = C, 69.16; H, 4.23; N, 12.18; S, 7.28%.

**2-Amino-5-(benzo[*d*]thiazol-2-yl)-6-oxo-4-phenyl-1-(*p*-tolyl)-1,6-dihydropyridine-3-carbonitrile** (**11c**)

Deep yellow crystals, m.p: 265-267 ^o^C, yield (61%); *ν*_max_ / cm^-1^ = 3402-3309 (broad, NH_2_), 2180 (CN), 1697 (CO); δ^1^_H_ = 2.31 (s, 3H, CH_3_), 6.68-7.58 (m, 13H, ArH and NH_2_), 8.01 (d, 1H, *J* = 8.1 Hz, ArH), 8.16 (d, 1H, *J* = 8.1 Hz, ArH); Elemental anal. For C_26_H_18_N_4_OS (434.52) Calc. = C, 71.87; H, 4.18; N, 12.89; O, 3.68; S, 7.38 %; Found = C, 71.70; H, 4.34; N, 12.63; S, 7.51%.

**2-Amino-5-(benzo[*d*]thiazol-2-yl)-4-(4-methoxyphenyl)-6-oxo-1-(*p*-tolyl)-1,6-dihydro-pyridine-3-carbonitrile** (**11d**)

Orange crystals, m.p: < 300 ^o^C, yield (69%); *ν*_max_ / cm^-1^ = 3346-3304 (broad, NH_2_), 2200 (CN), 1656 (CO); δ^1^_H_ = 2.29 (s, 3H, CH_3_), 3.72 (s, 3H, OCH_3_), 6.86 (d, 2H, *J* = 8.5 Hz, ArH), 7.18 (d, 2H, *J* = 9.0 Hz, ArH), 7.23-7.31 (m, 6H, ArH and NH_2_), 7.37 (d, 2H, *J* = 8.0 Hz, ArH), 7.50 (d, 1H, *J* = 8.0 Hz, ArH), 7.87 (d, 1H, *J* = 8.0 Hz, ArH); Elemental anal. For C_27_H_20_N_4_O_2_S (464.54) Calc. = C, 69.81; H, 4.34; N, 12.06; S, 6.90 %; Found = C, 69.65; H, 4.19; N, 12.29; S, 7.06%.

**2-Amino-5-(benzo[*d*]thiazol-2-yl)-1-(4-chlorophenyl)-6-oxo-4-phenyl-1,6-dihydro-pyridine-3-carbonitrile** (**11e**)

Yellow crystals, m.p: 293-295 ^o^C, yield (64%); *ν*_max_ / cm^-1^ = 3394-3302 (broad, NH_2_), 2183 (CN), 1697 (CO); δ^1^_H_ = 7.08-7.45 (m, 7H, ArH and NH_2_), 7.48 (d, 2H, *J* = 8.1 Hz, ArH), 7.53 (d, 2H, *J* = 8.7 Hz, ArH), 7.61 (d, 2H, *J* = 7.8 Hz, ArH), 8.01 (d, 1H, *J* = 8.1 Hz, ArH), 8.11 (d, 1H, *J* = 8.1 Hz, ArH); Elemental anal. For C_25_H_15_ClN_4_OS (454.93) Calc. = C, 66.00; H, 3.32; Cl, 7.79; N, 12.32; S, 7.05%; Found = C, 66.18; H, 3.16; N, 12.55; S, 7.21%.

**2-Amino-5-(benzo[*d*]thiazol-2-yl)-1-(4-chlorophenyl)-4-(4-methoxyphenyl)-6-oxo-1,6-dihydropyridine-3-carbonitrile** (**11f**)

Brown crystals, m.p: < 300 ^o^C, yield (58%); ν_max_ / cm^-1^ = 3447 and 3298 (NH_2_), 2218 (CN), 1657 (CO); δ^1^_H_ = 3.82 (s, 3H, OCH_3_), 6.48-7.58 (m, 10H, ArH and NH_2_), 7.66 (d, 2H, *J* = 8.4 Hz, ArH), 8.03 (d, 1H, *J* = 8.1 Hz, ArH), 8.17 (d, 1H, *J* = 8.1 Hz, ArH); Elemental anal. For C_26_H_17_ClN_4_O_2_S (484.96) Calc. = C, 64.39; H, 3.53; Cl, 7.31; N, 11.55; S, 6.61%; Found = C, 64.23; H, 3.37; N, 11.78; S, 6.78%.

**Biological evaluation**

**Antibacterial activity assessment [Agar Diffusion Method]**

1. The agar-based media plate surface was inoculated by spreading a volume of the microbial inoculum over the entire agar surface.
2. Using a sterile cork borer or a tip, a hole with a diameter of 6 to 8 mm was made.
3. Using a micropipette, (20 µL) volume of the antimicrobial agent or extract solution at the desired concentration was introduced into the well.
4. The inoculated plates were incubated under suitable conditions depending upon the tested microorganism. The antimicrobial agent diffused through the agar medium and inhibited the growth of the microbial strain tested.
5. After a suitable incubation period (16 to 48 hours) according to the optimum growth conditions, the resulting inhibition zone diameters (in mm) around the wells were measured to the nearest whole millimeter at the point at which there is a prominent reduction in growth. If growth is insufficient at the recommended times, the plates should be re-incubated and read later.

**Minimum Inhibition Concentration (MIC) Methodology**

For each strain, three to five isolated colonies were selected from the fresh agar plate and transferred into a tube containing 3-4 ml of the sterile broth medium. The bacterial suspension was mixed well and incubated at 35-37 °C for 2-6 hours. The turbidity of the bacterial suspension should be equal to or greater than the turbidity of a McFarland Standard 0.5. After that, 1 mg of the tested compound (antimicrobial agent) was dissolved in 1 ml of DMSO and a two-fold serial dilution was done using a broth medium. A fixed volume of the prepared bacterial inoculum was added to each tube and incubated at 37 °C for 16-20 hours. The MIC is defined as the lowest concentration of the antimicrobial agent that inhibits visible growth of the tested isolate as observed with the unaided eye [45].

**Enzyme inhibition assay for DHFR activity for 2c, 4m and 9c**

Compounds **2c**, **4m** and **11c** were examined for their inhibitory activities against DHFR enzyme. The master mixture (6 μL 5X Kinase Buffer + 1 μL ATP (500 μM) + 1 μL 50 X PTK substrate + 17 μL water) was prepared then, 25 μL to every well was added. 5 μL of Inhibitor solution of each well labeled as “Test Inhibitor” was added. However, for the “Positive Control" and “Blank”, 5 μL of the same solution without inhibitor (Inhibitor buffer) was added. 3 mL of 1X Kinase Buffer by mixing 600 μL of 5X Kinase Buffer with 2400 μL water was prepared. So, 3 mL of 1X Kinase Buffer became sufficient for 100 reactions. To the wells designated as "Blank", 20 μl of 1X Kinase Buffer was added. DHFR enzyme on ice was defrosted. Upon first defrost, briefly the tube containing enzyme was whirled to recover full content of the tube. The amount of DHFR required for the assay and dilute enzyme to 1 ng/μL with 1X Kinase Buffer was calculated. Moreover, the remaining undiluted enzyme in aliquots was stored at -80°C. The reaction was initiated by adding 20 μL of diluted DHFR enzyme to the wells designated “Positive Control” and "Test Inhibitor Control", after that it was incubated at 30 °C for 40 minutes. After the 40 minutes reaction, 50 μL of Kinase-Glo Max reagent was added to each well and the plate was covered with aluminum foil and incubated at room temperature for 15 min. Luminescence was measured using the microplate reader.

**Figure S1**. **^1^H-NMR spectrum of compound 2a**


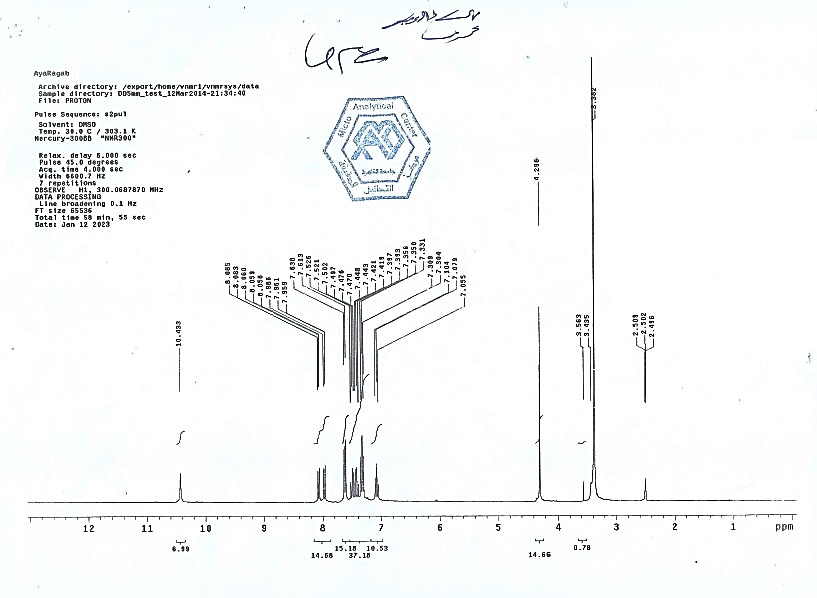


**Figure S2**. **^13^C-NMR spectrum of compound 2a**


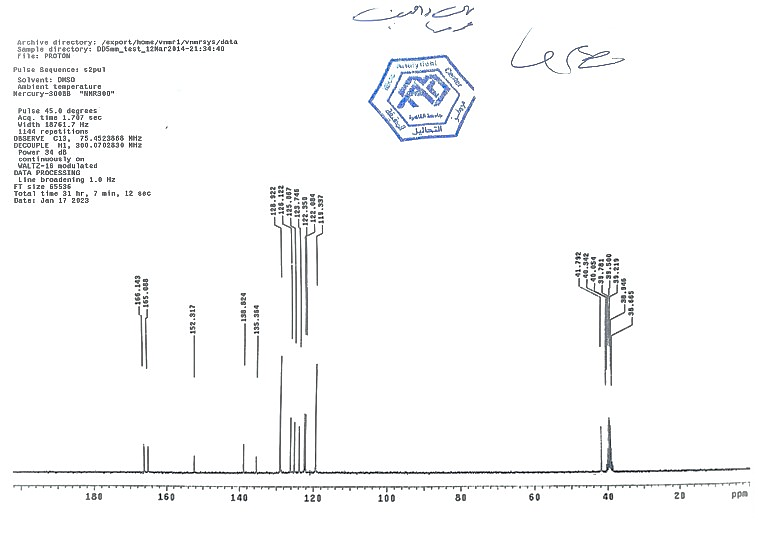


**Figure S3**. **IR spectrum of compound 2a**


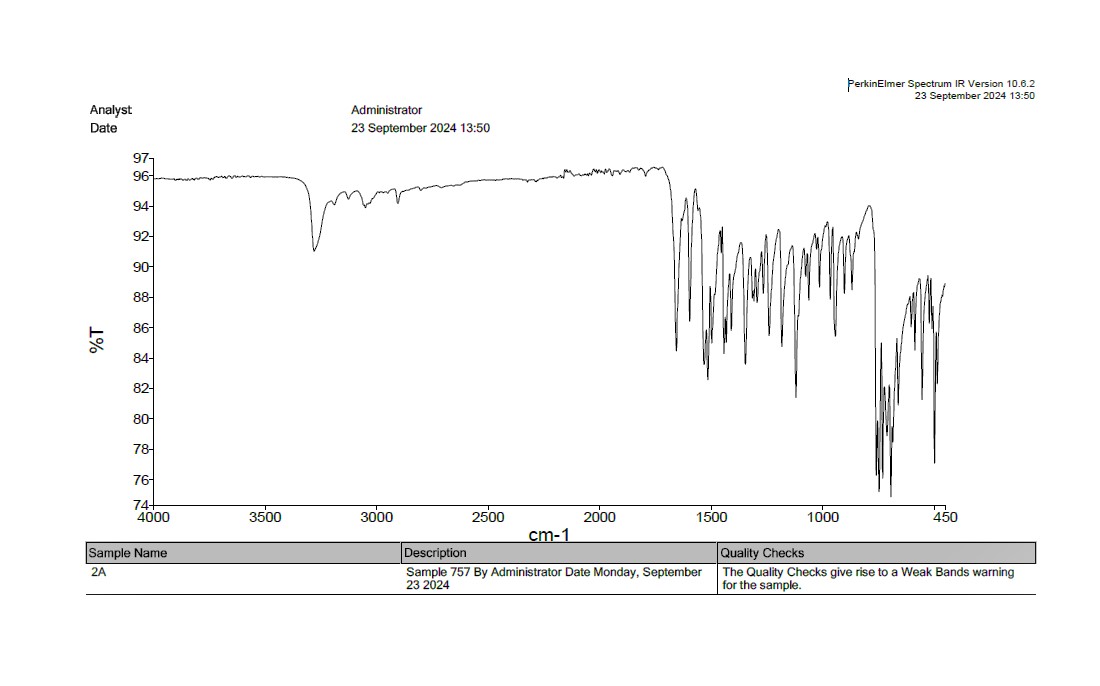


**Figure S4**. **Mass spectrum of compound 2a**


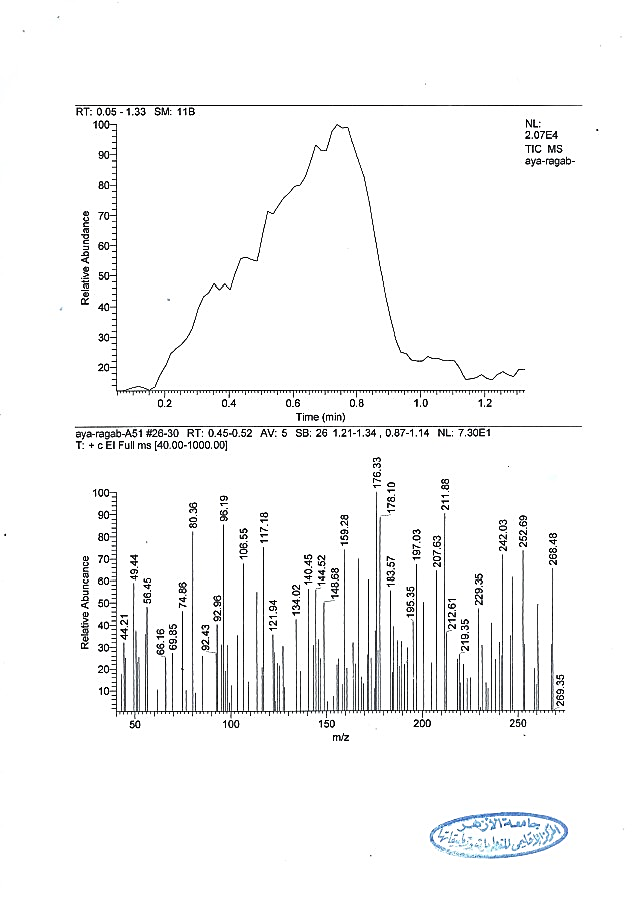


**Figure S5**. **^1^H NMR spectrum of compound 2b**


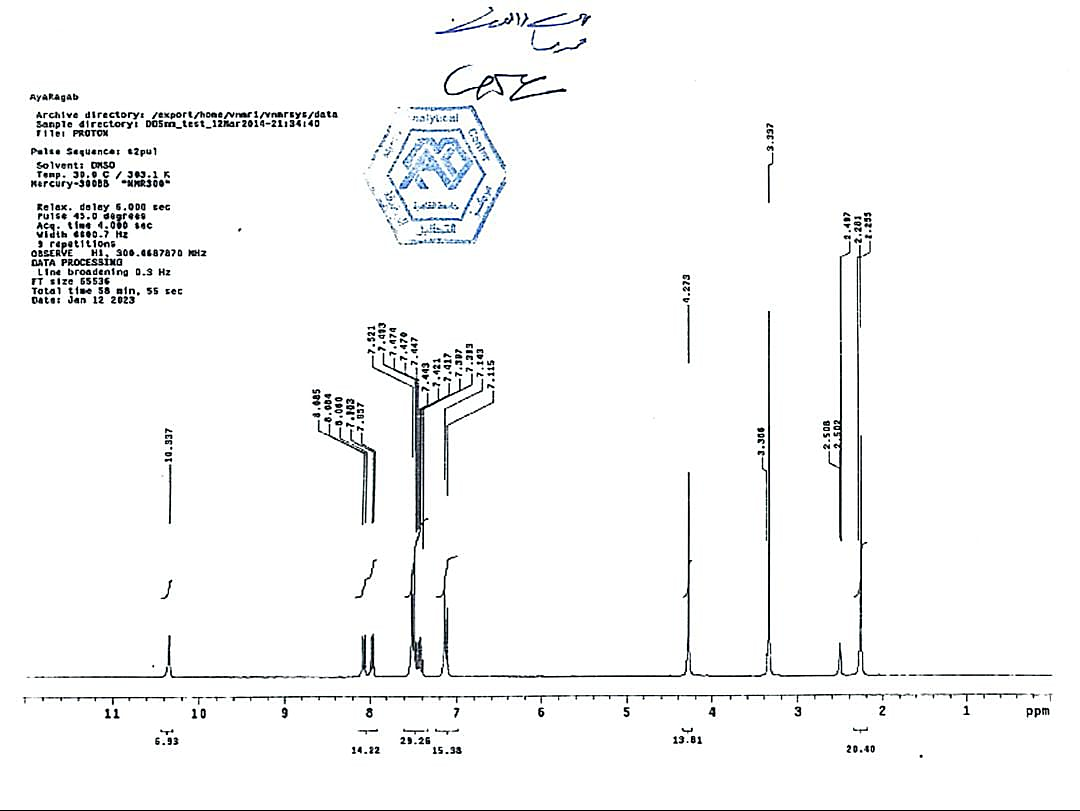


**Figure S6**. **Mass spectrum of compound 2b**


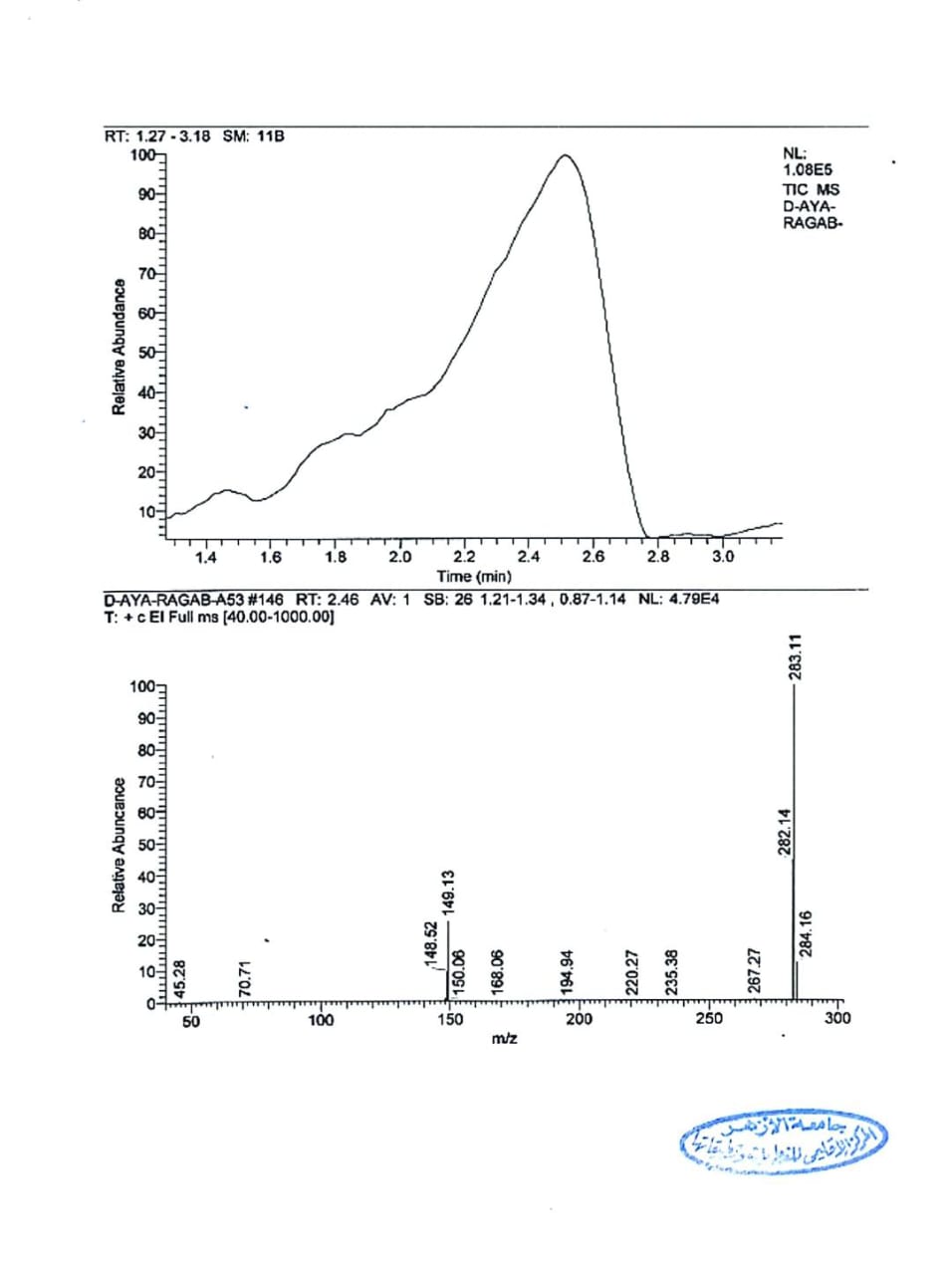


**Figure S7**. **IR spectrum of compound 2b**


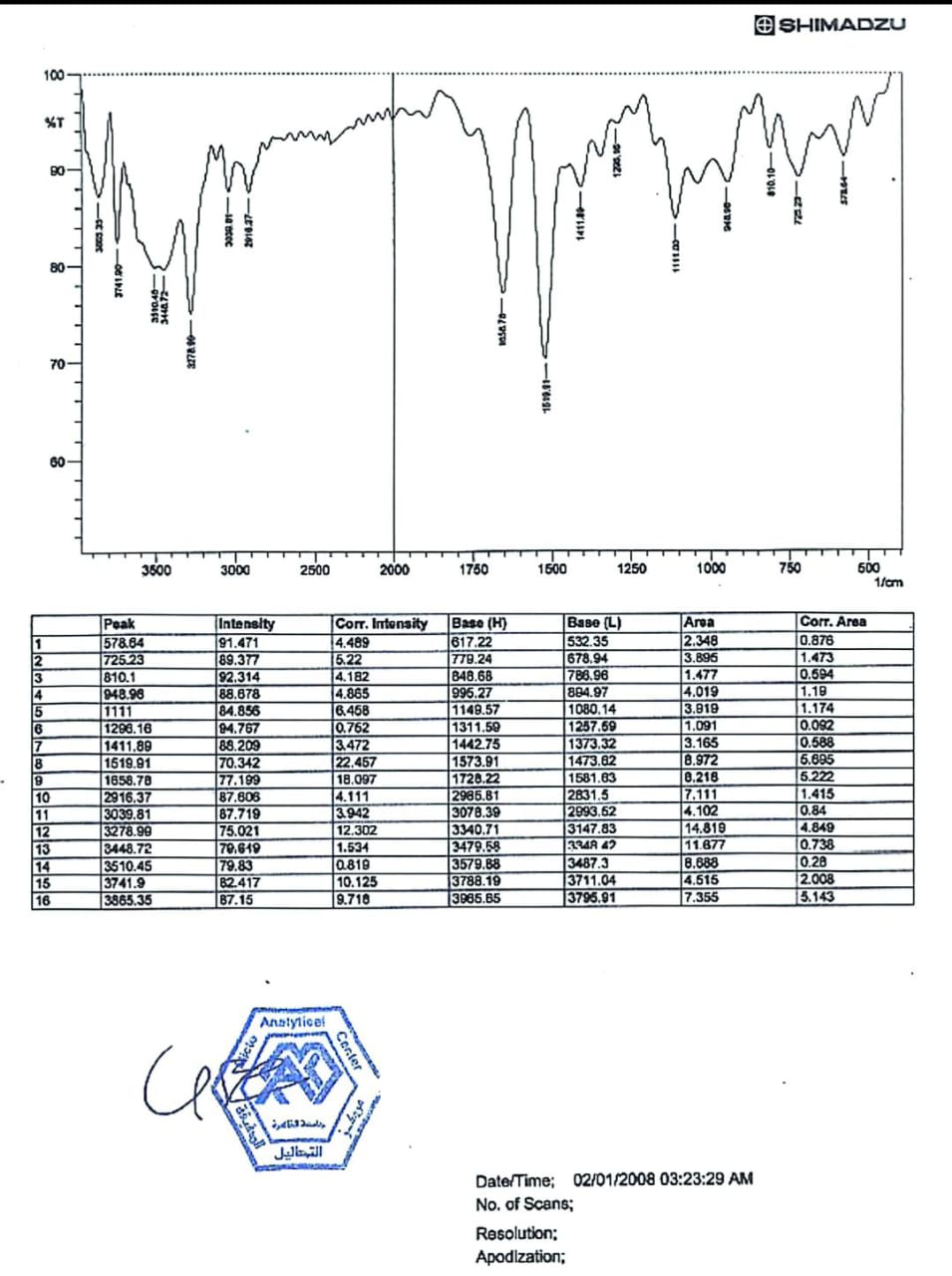


**Figure S8**. **^1^H NMR spectrum of compound 2c**


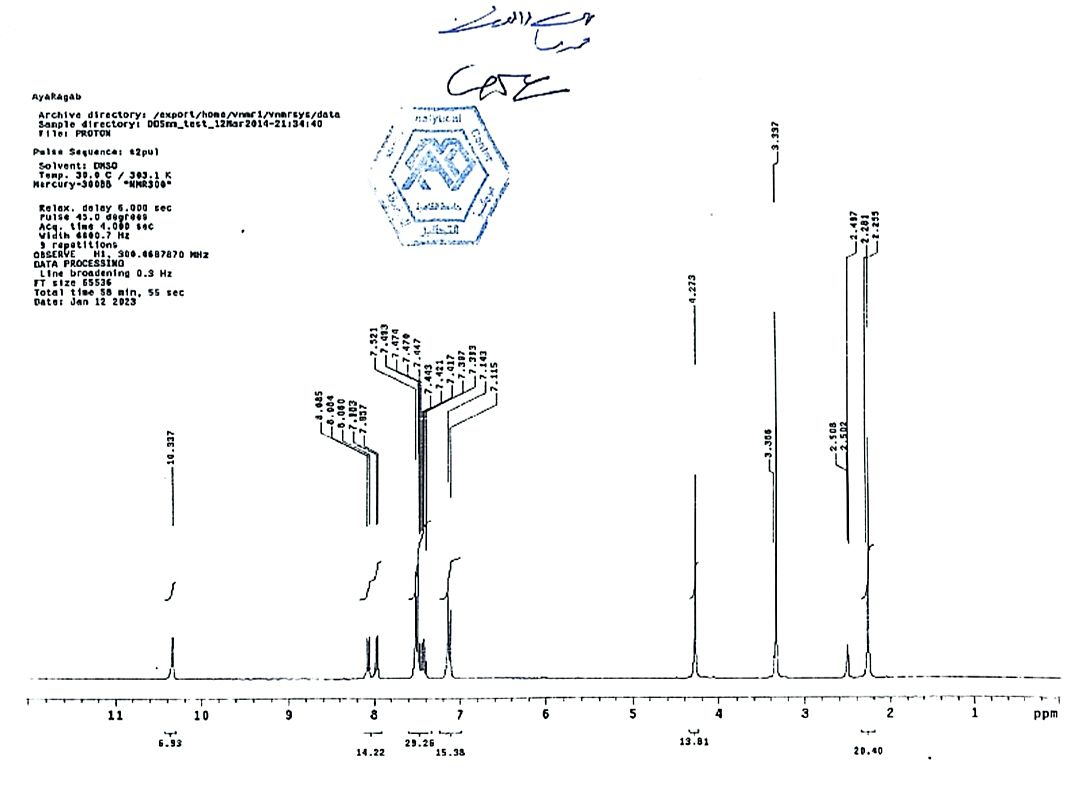


**Figure S9**. **^13^C NMR spectrum of compound 2c**


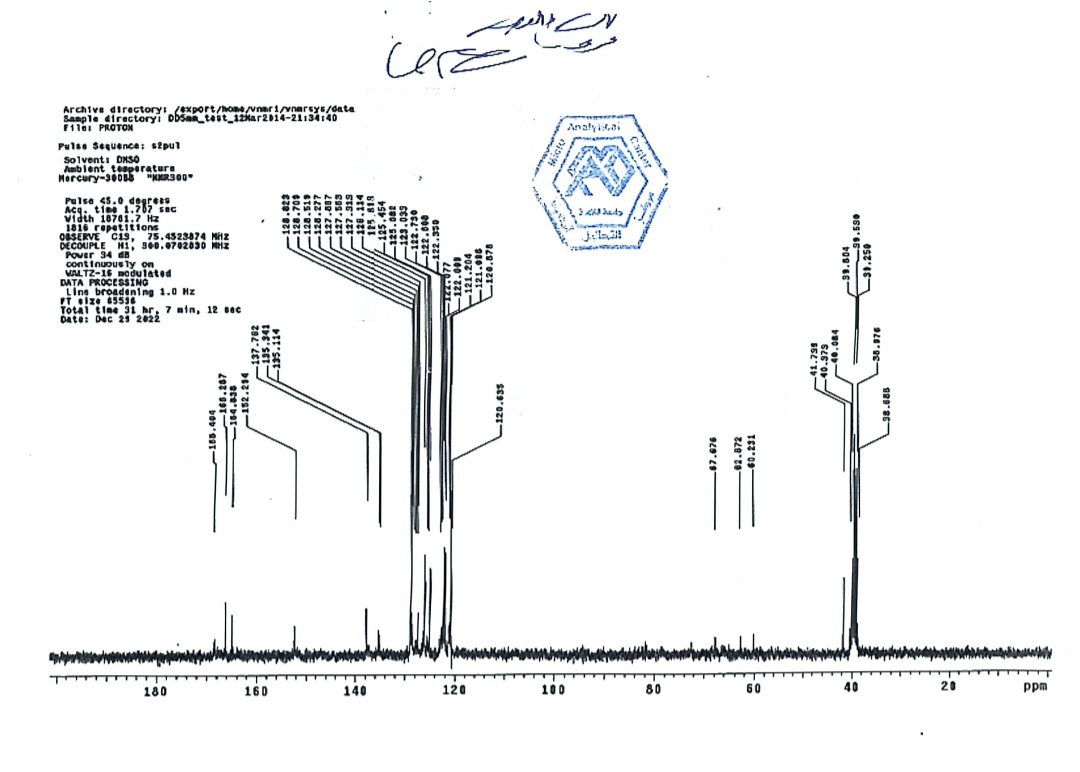


**Figure S10**. **IR spectrum of compound 2c**


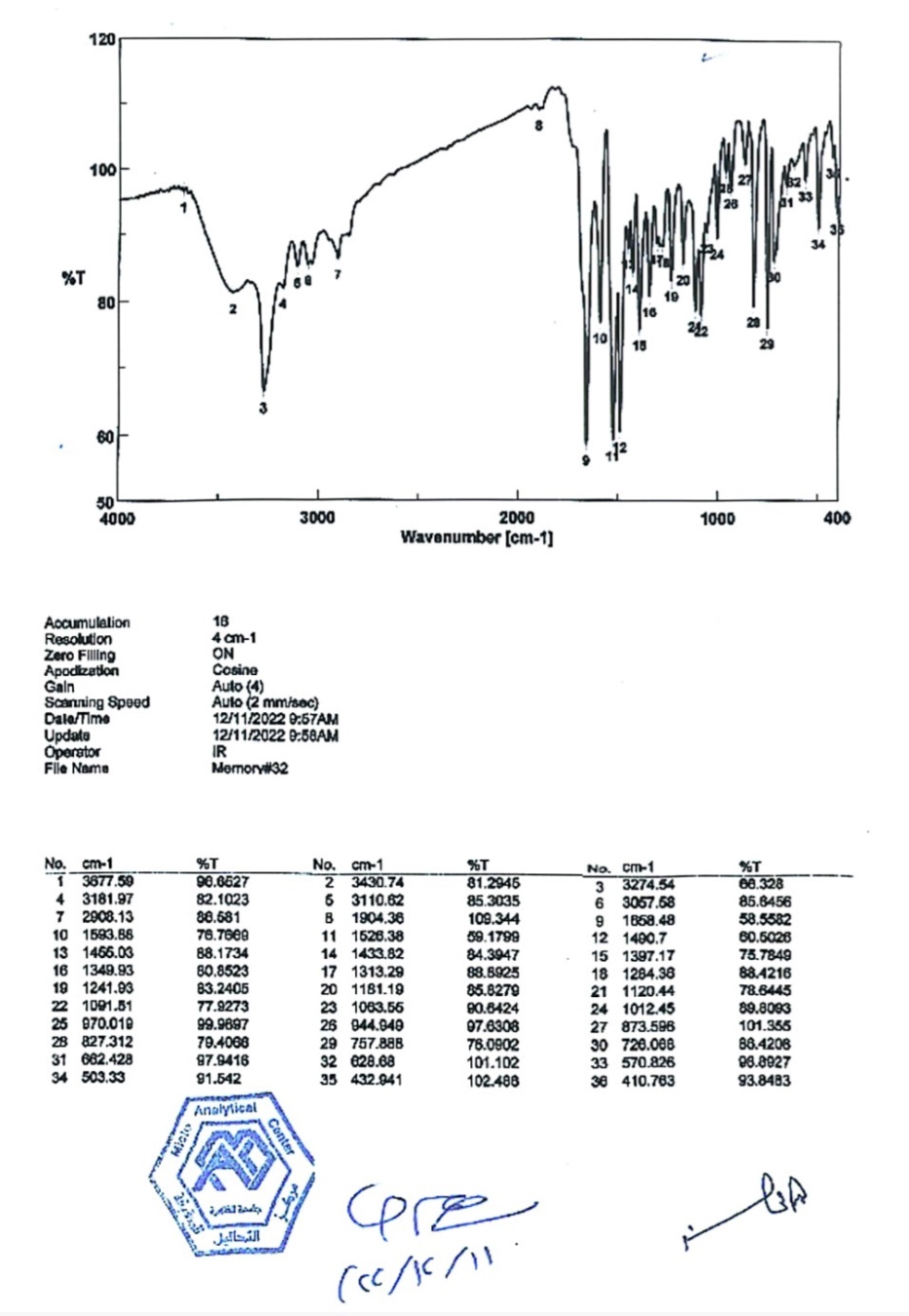


**Figure S11**. **Mass spectrum of compound 2c**


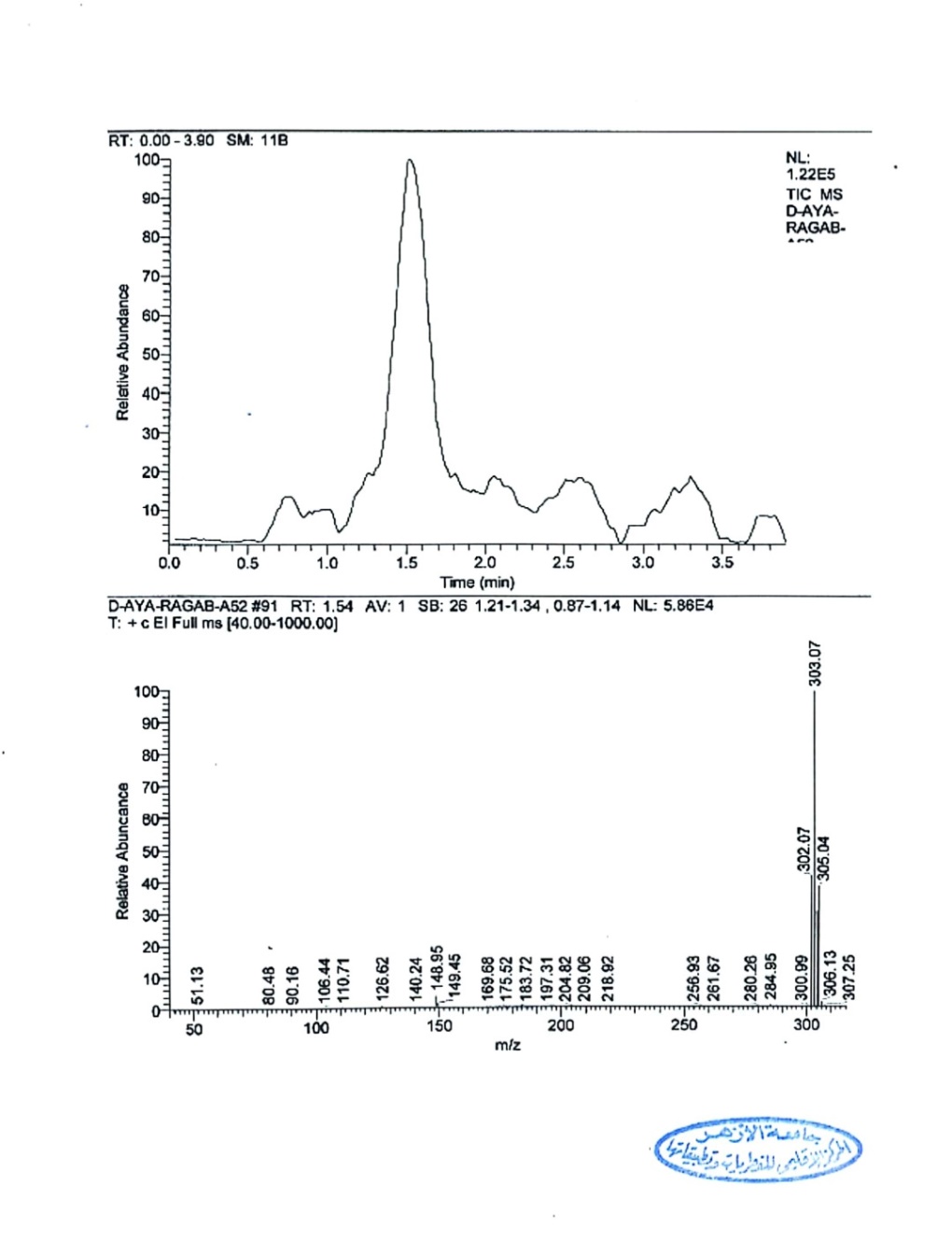


**Figure S12**. **Mass spectrum of compound 2d**


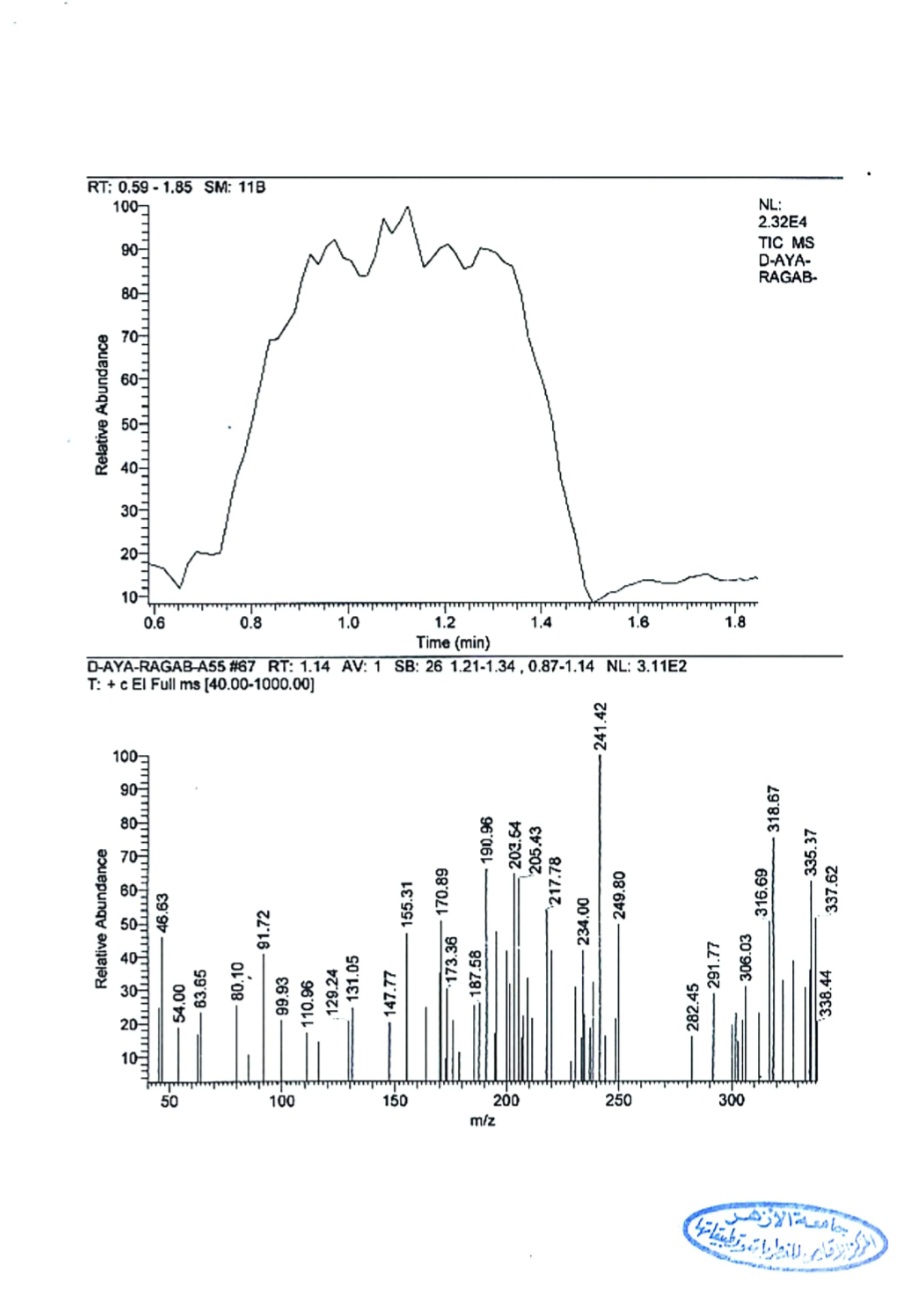


**Figure S13**. **^1^H NMR spectrum of compound 2e**


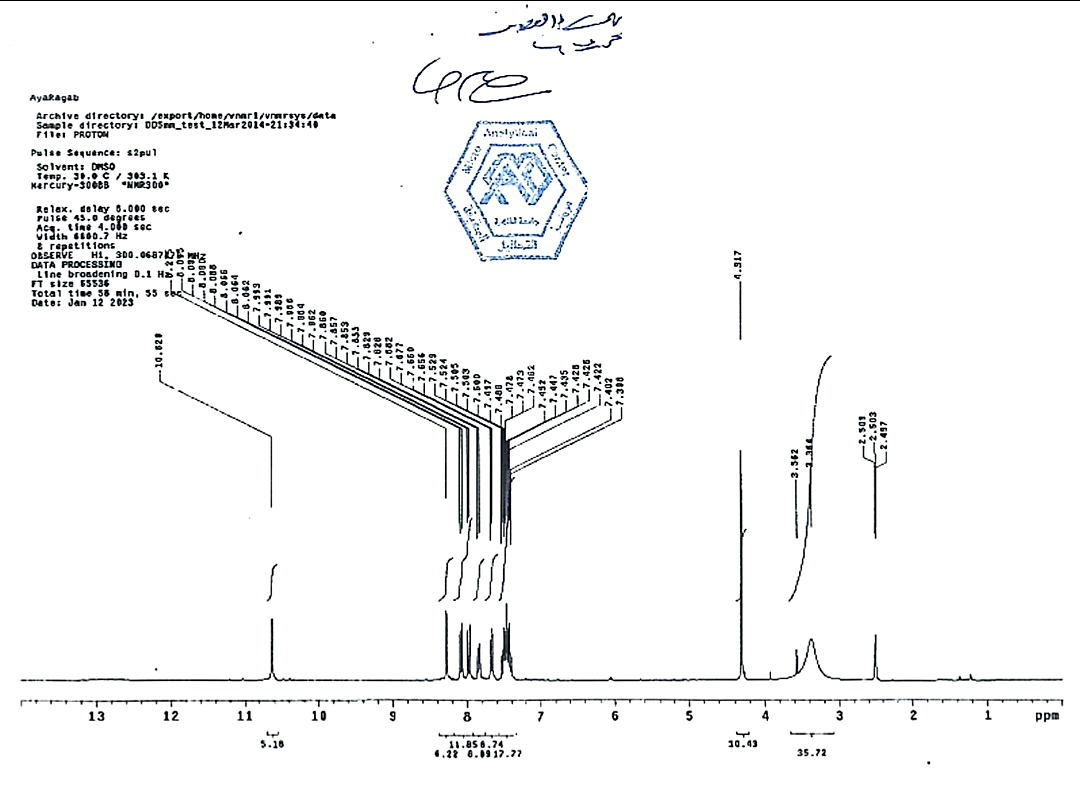


**Figure S14**. **^13^C NMR spectrum of compound 2e**

**
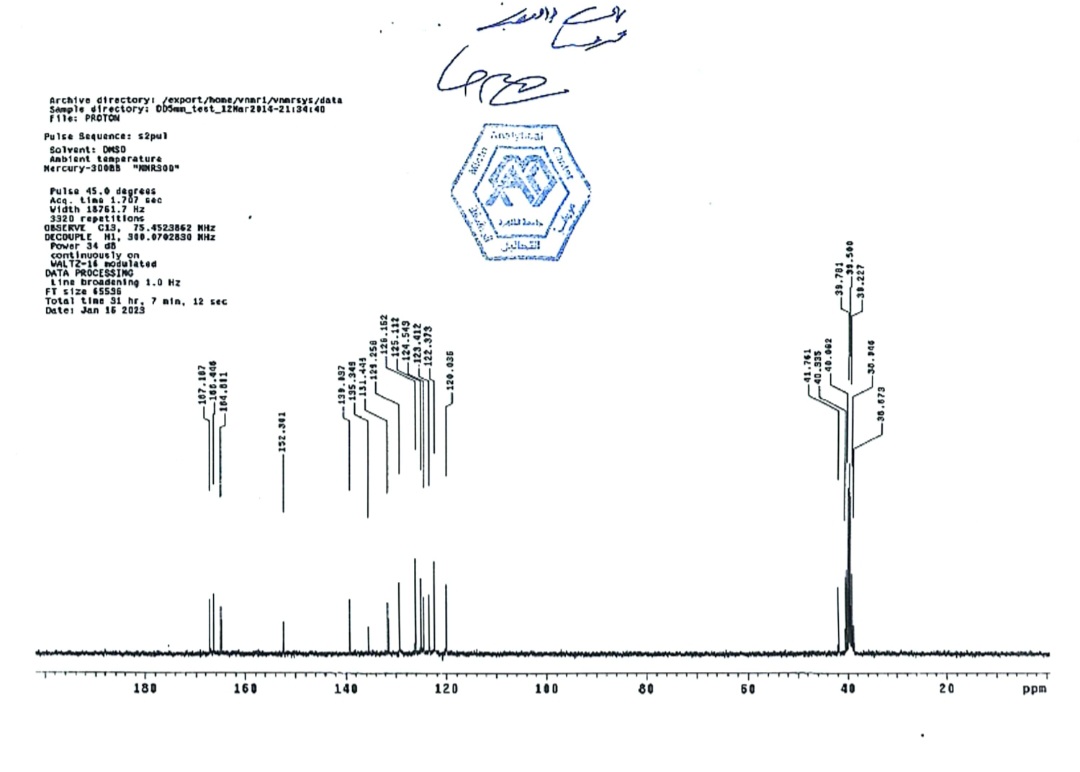
**

**Figure S15**. **IR spectrum of compound 2e**

**
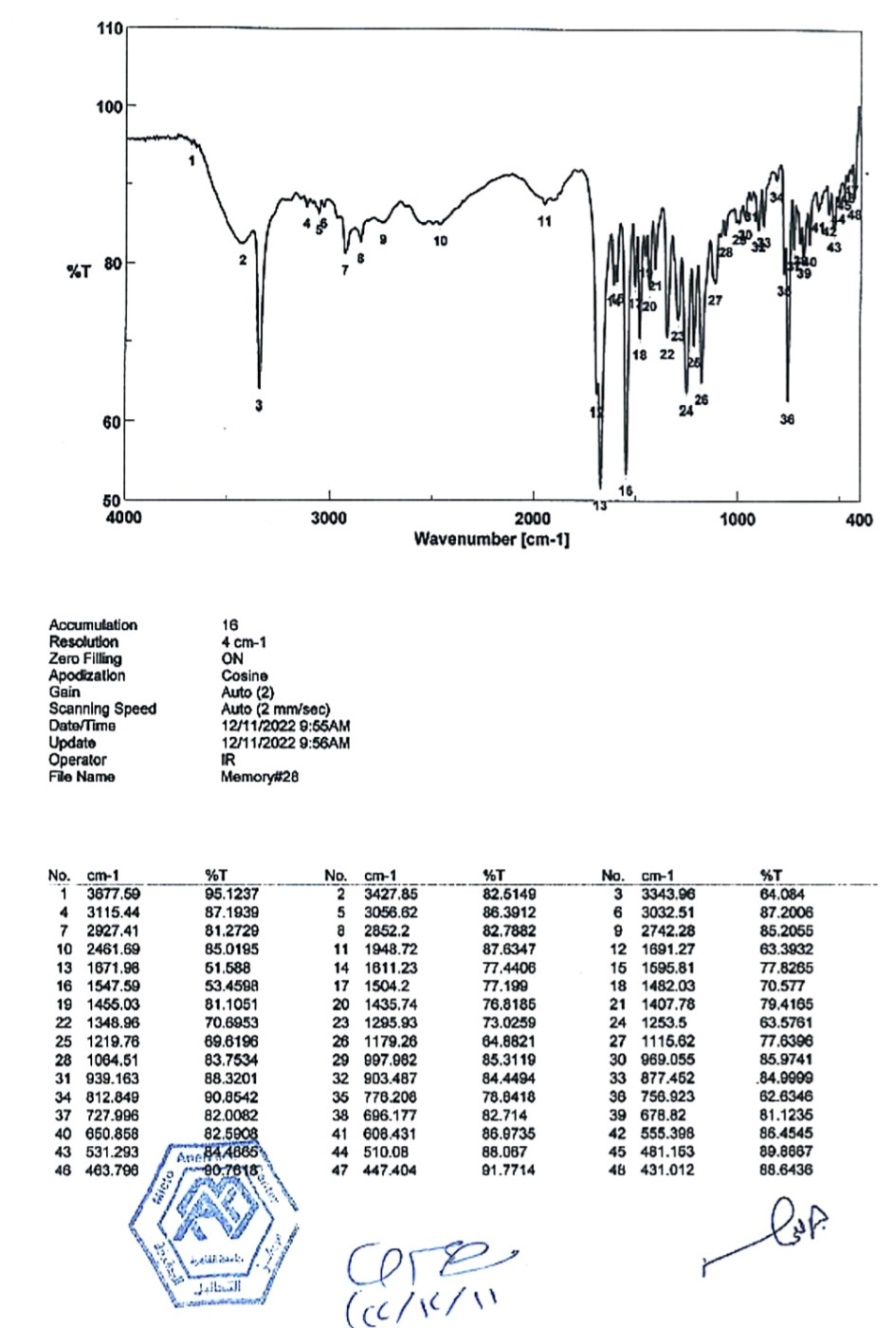
**

**Figure S16**. **^1^H NMR spectrum of compound 4a**

**
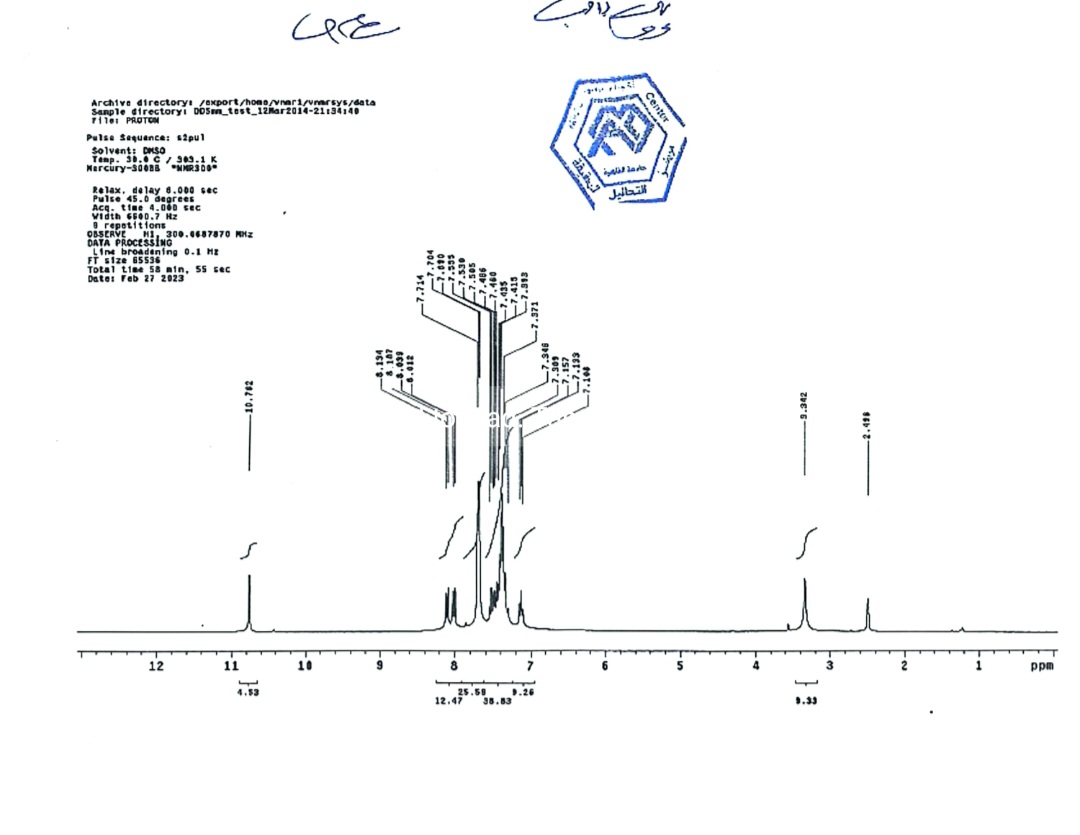
**

**Figure S17**. **^13^C NMR spectrum of compound 4a**

**
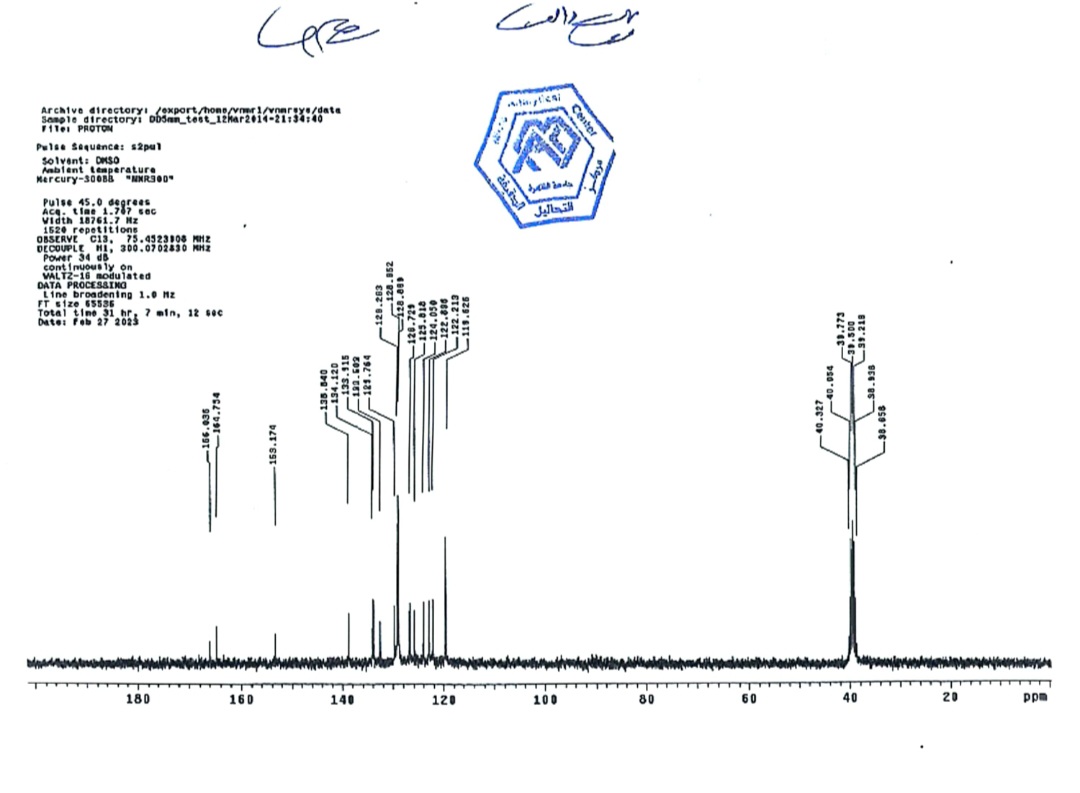
**

**Figure S18**. **IR spectrum of compound 4a**

**
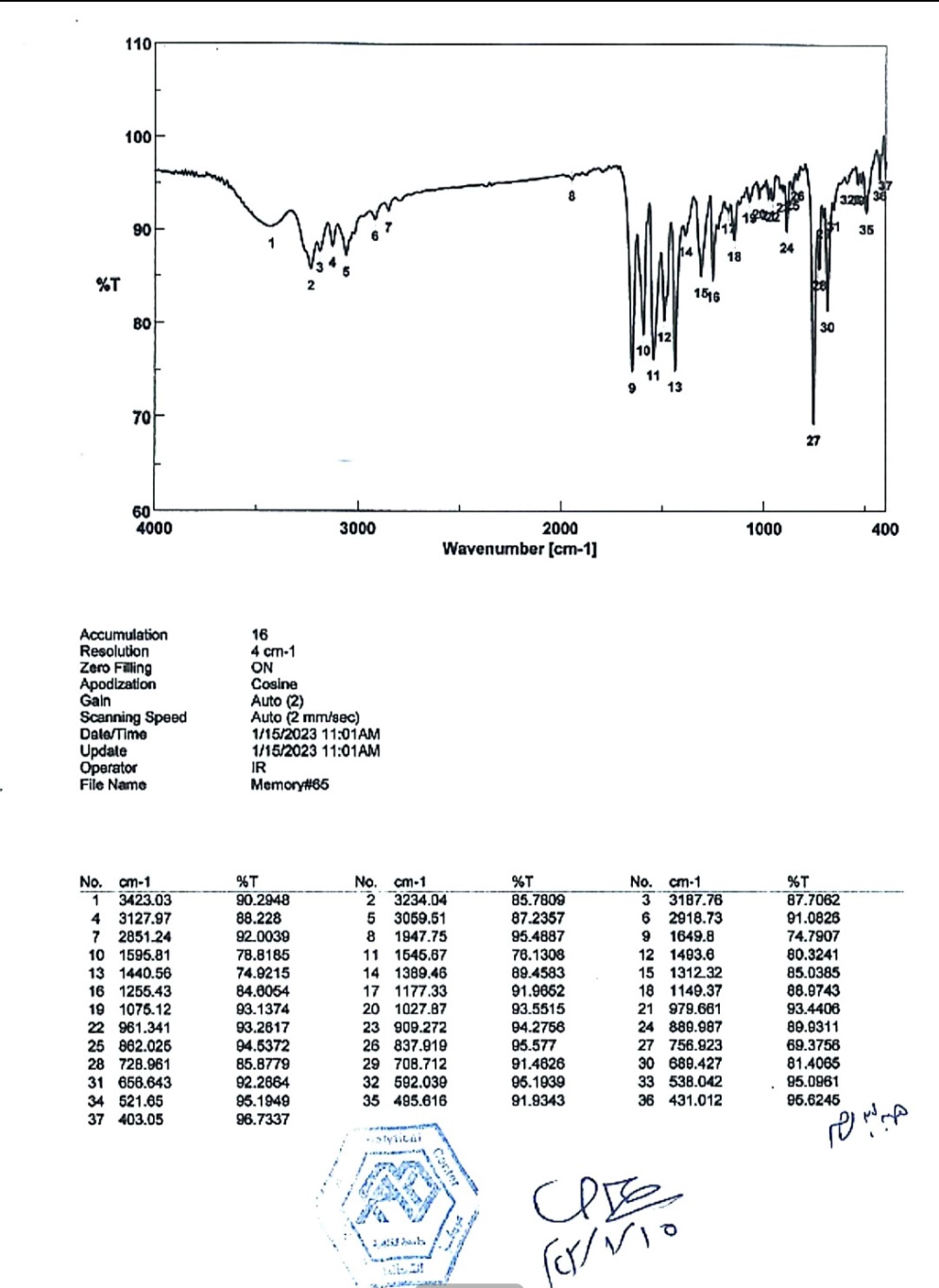
**

**Figure S19**. **^1^H NMR spectrum of compound 4b**

**
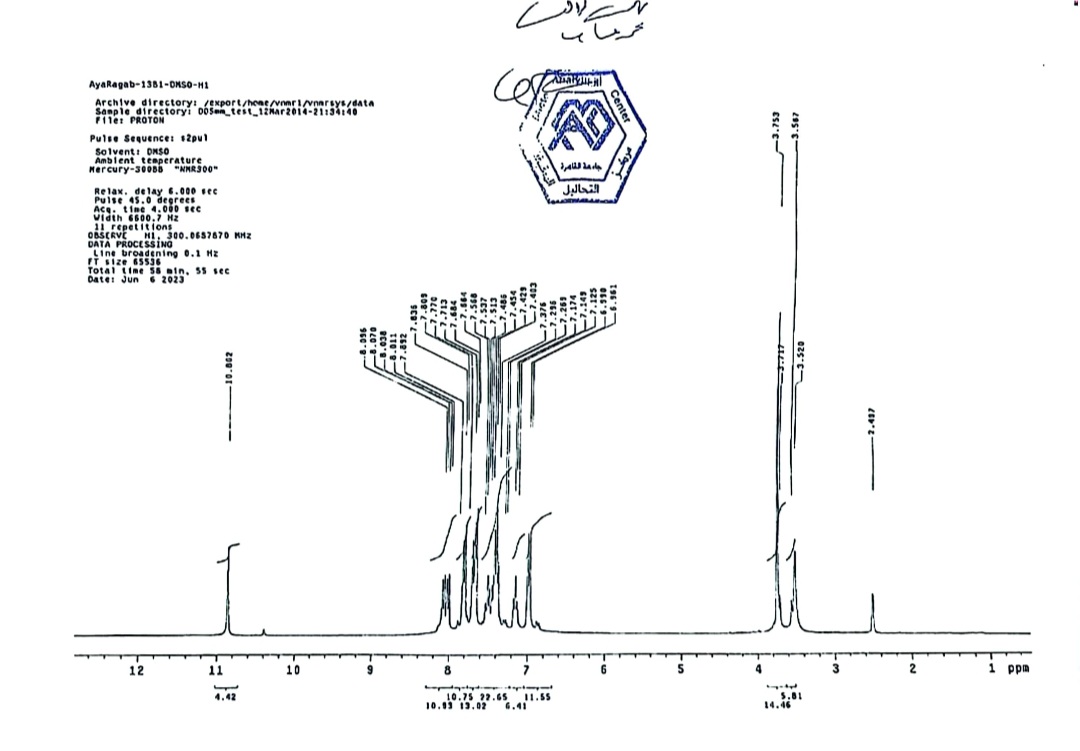
**

**Figure S20**. **^13^C NMR spectrum of compound 4b**

**
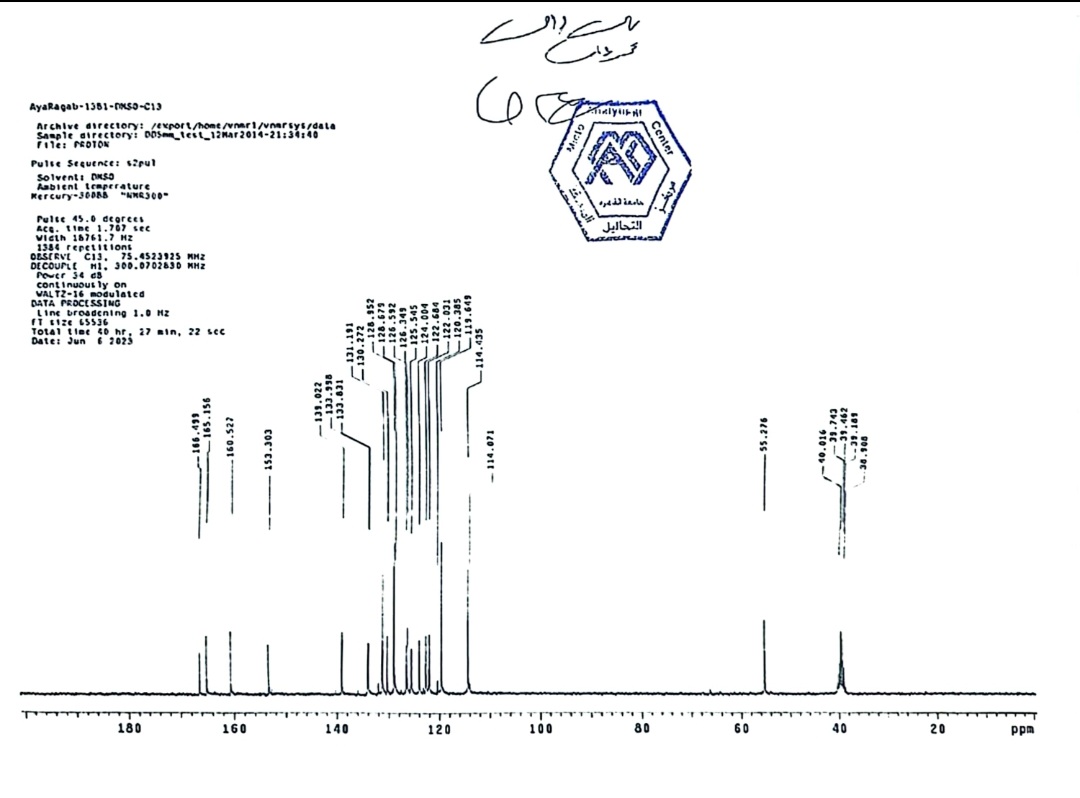
**

**Figure S21**. **IR spectrum of compound 4b**

**
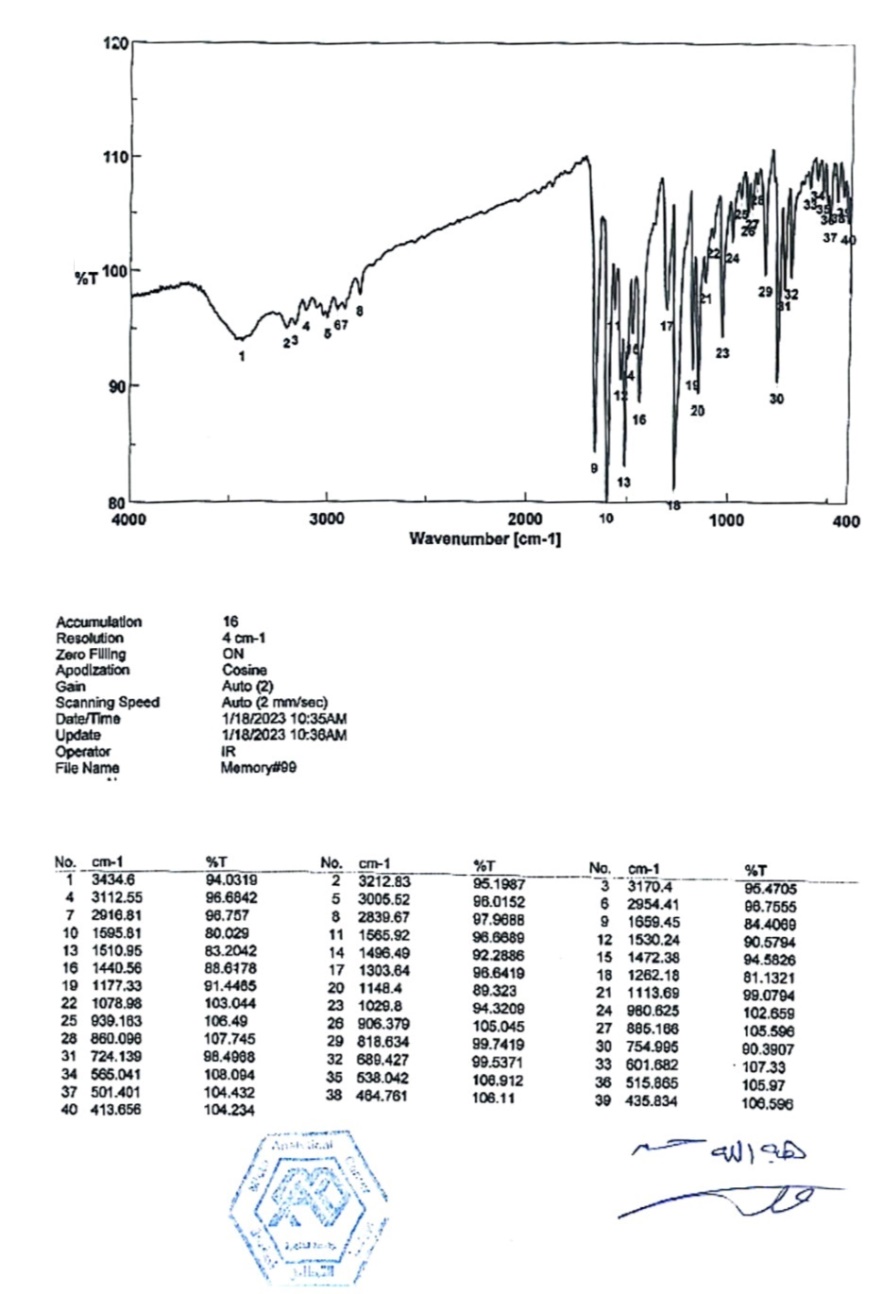
**

**Figure S22**. **^1^H NMR spectrum of compound 4c**

**
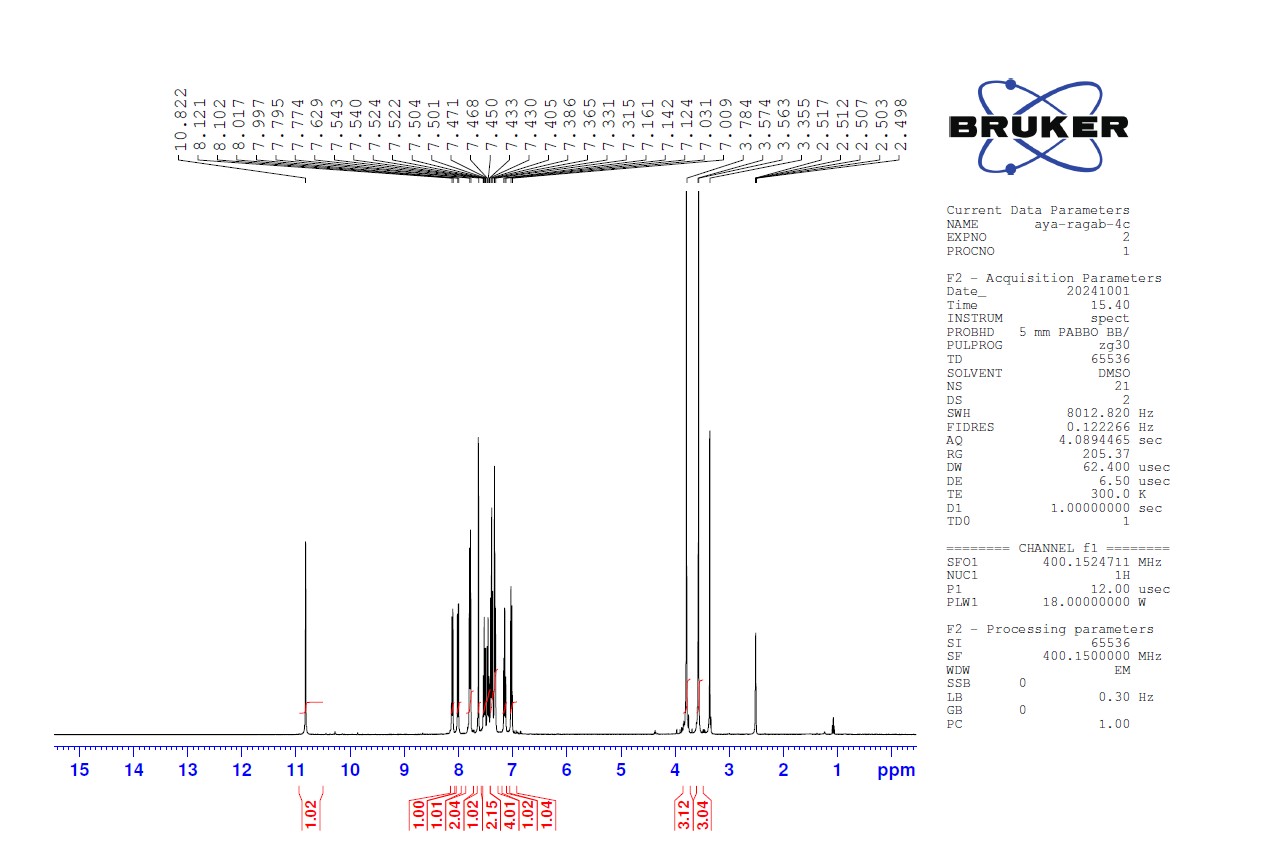
**

**Figure S23**. **^13^C NMR spectrum of compound 4c**

**
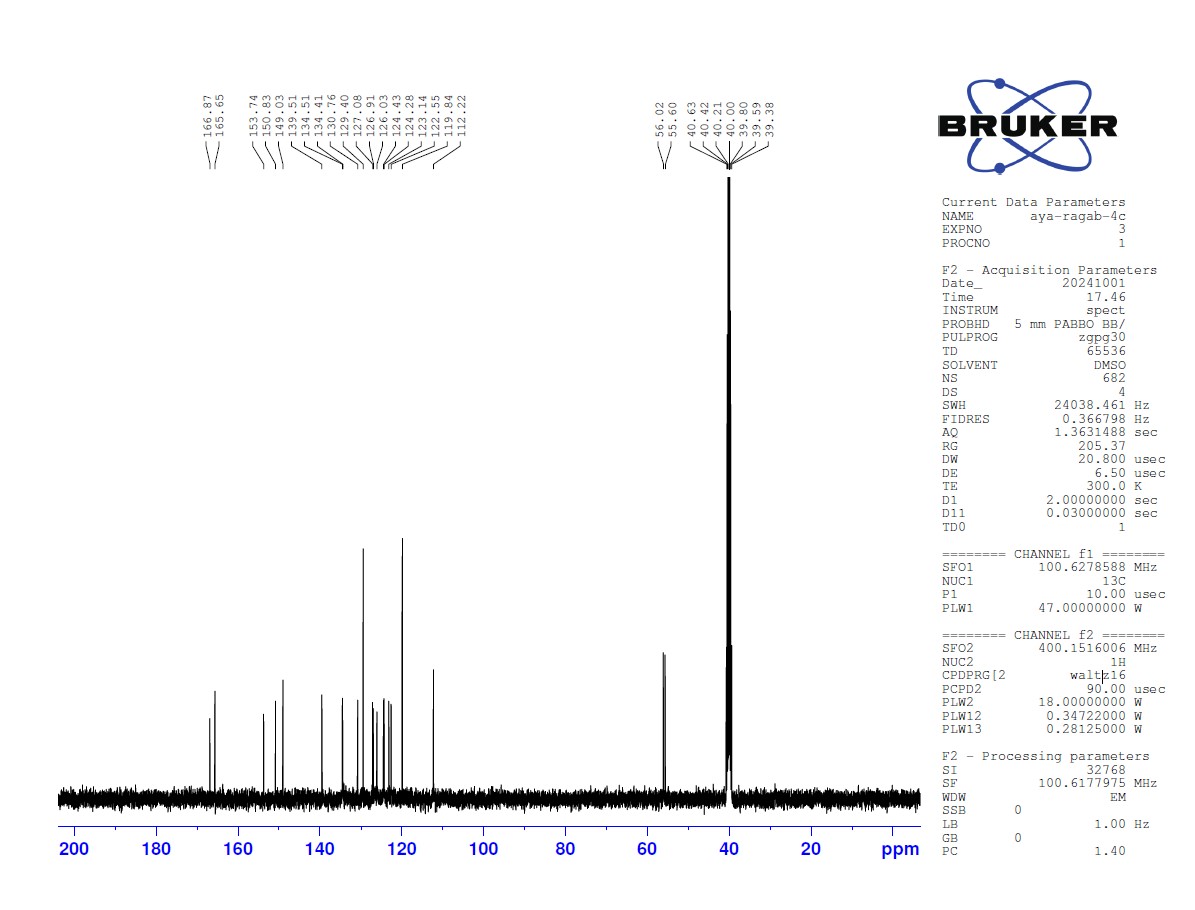
**

**Figure S24**. **Mass spectrum of compound 4c**

**
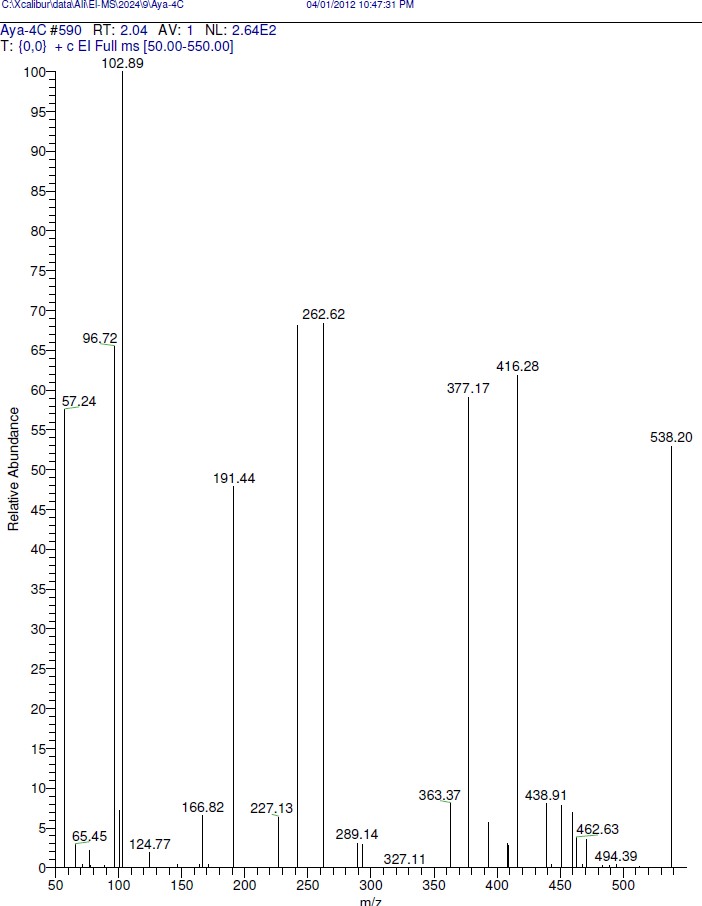
**

**Figure S25**. **^1^H NMR spectrum of compound 4d**

**
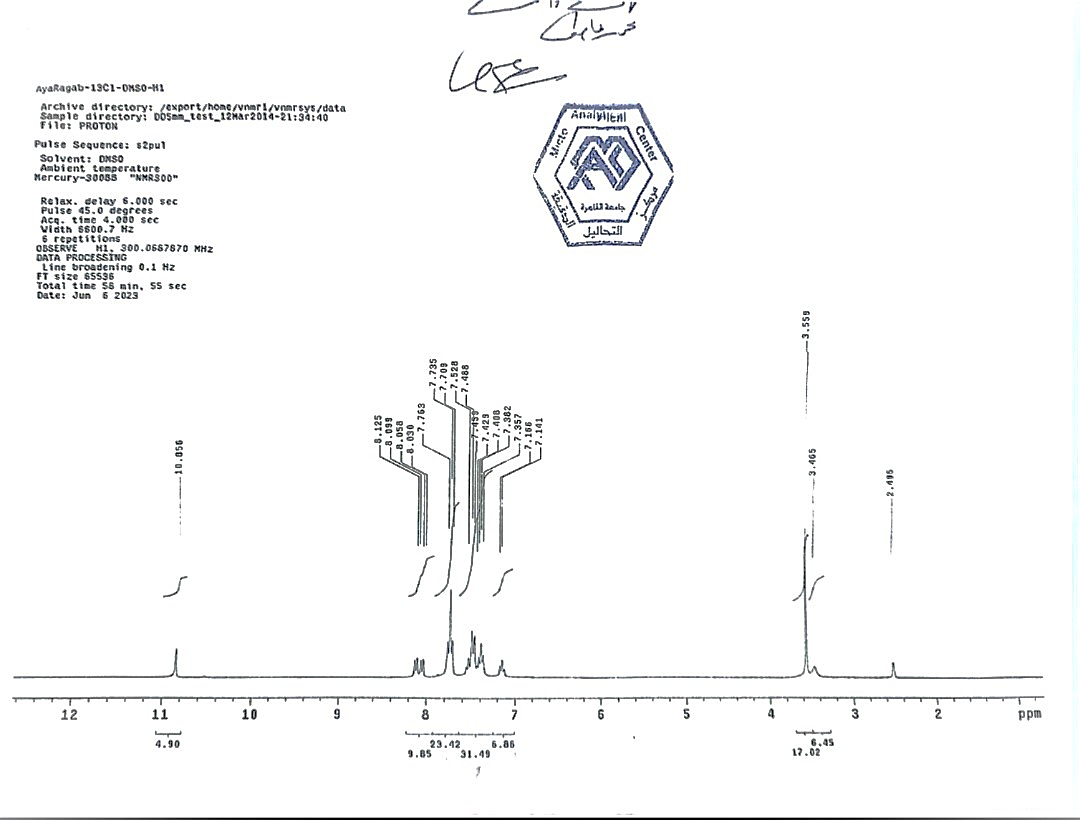
**

**Figure S26**. **^13^C NMR spectrum of compound 4d**

**
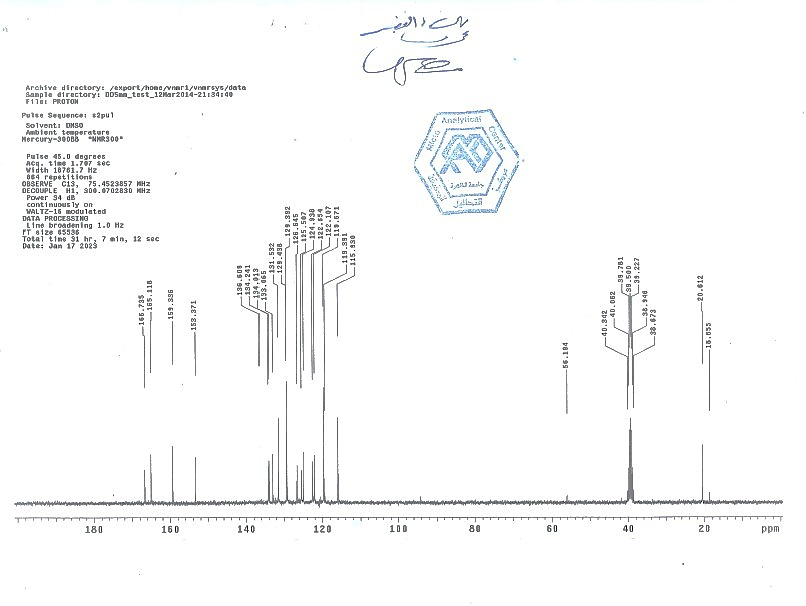
**

**Figure S27. IR spectrum of compound 4d**

**
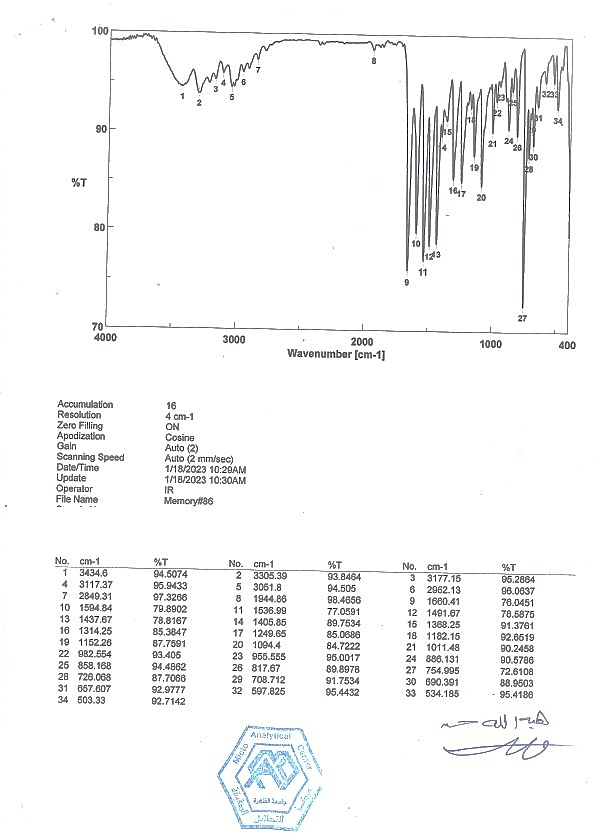
**

**Figure S28**. **^1^H NMR spectrum of compound 4e**

**
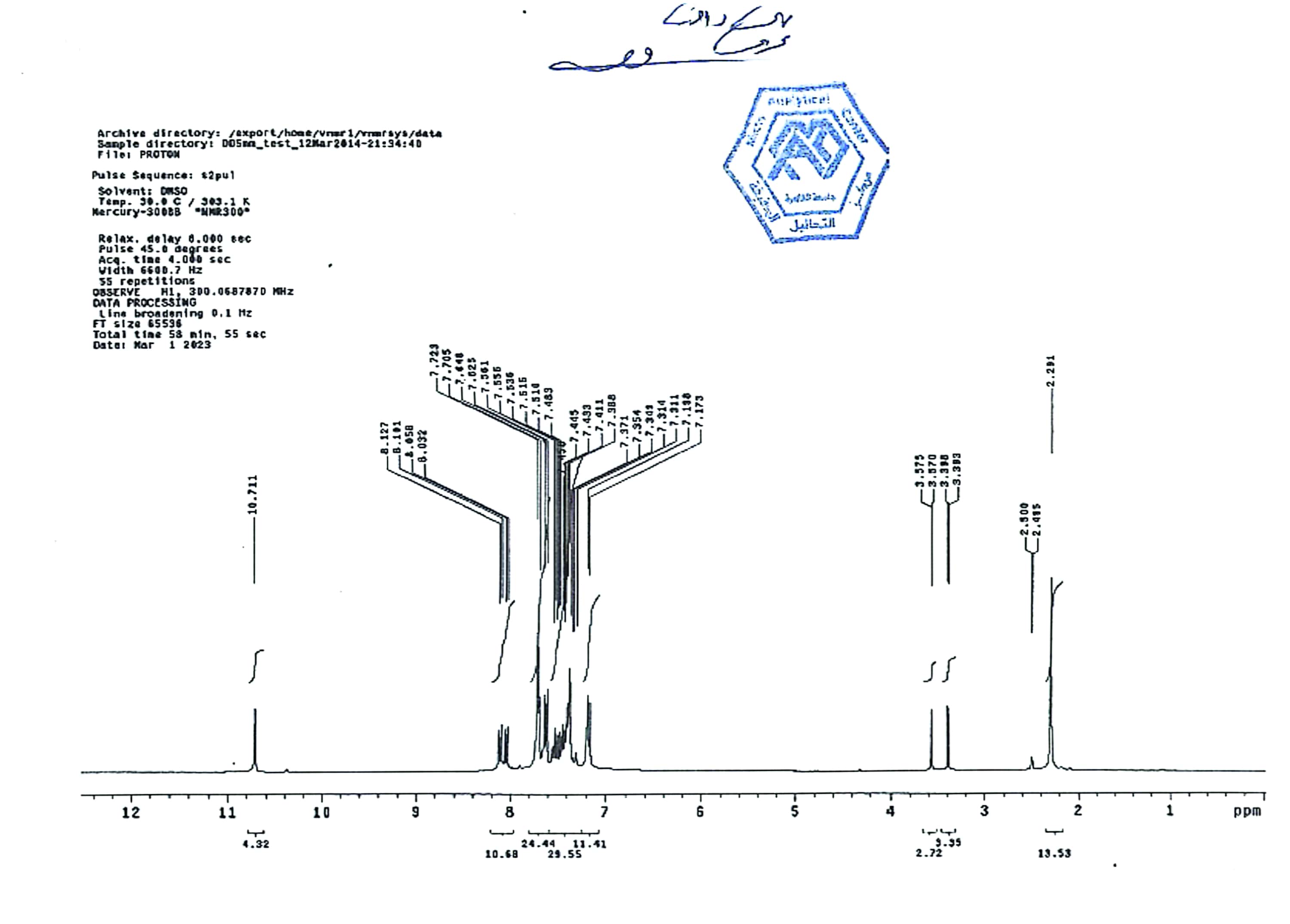
**

**Figure S29**. **^13^C NMR spectrum of compound 4e**

**
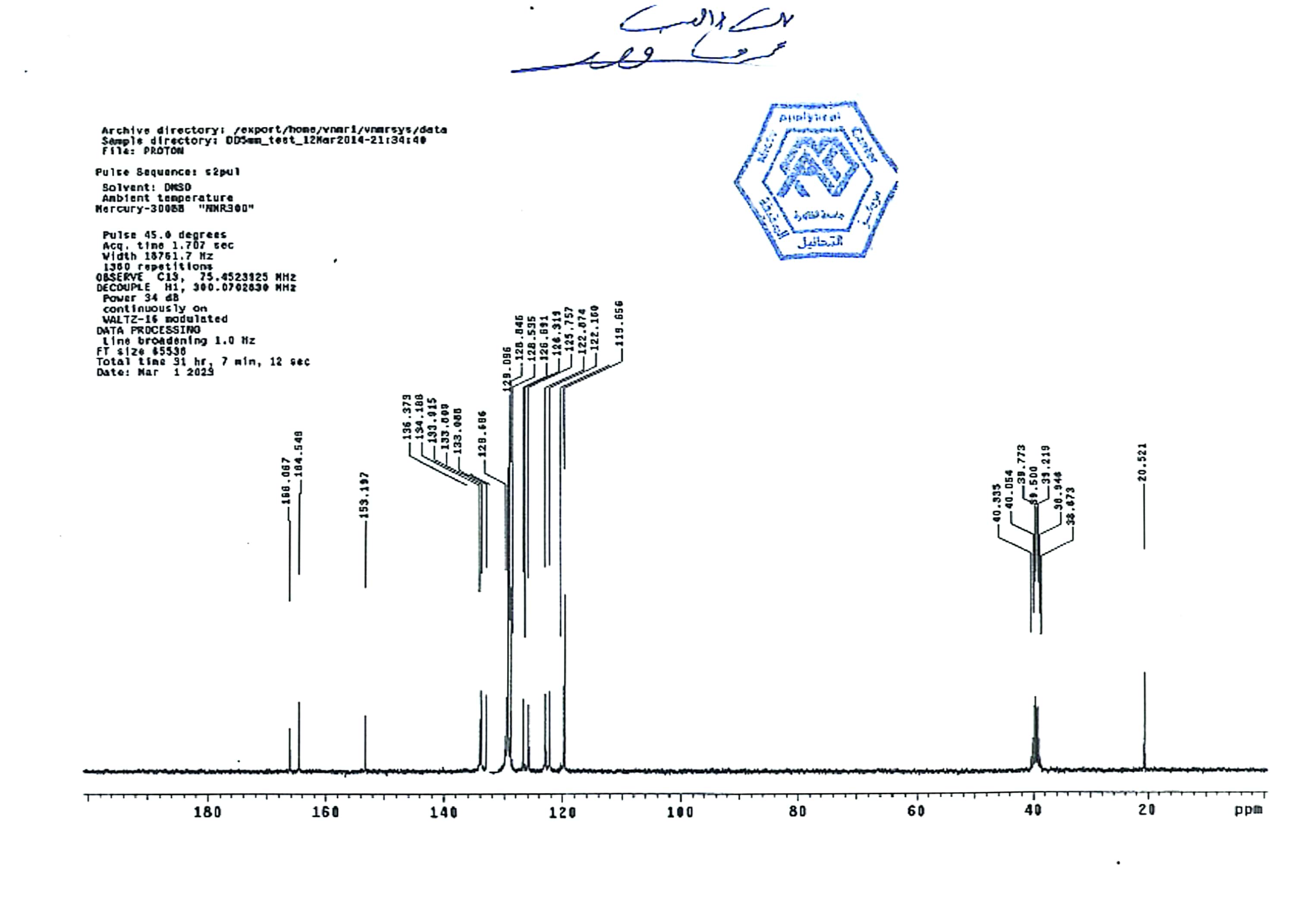
**

**Figure S30**. **IR spectrum of compound 4e**

**
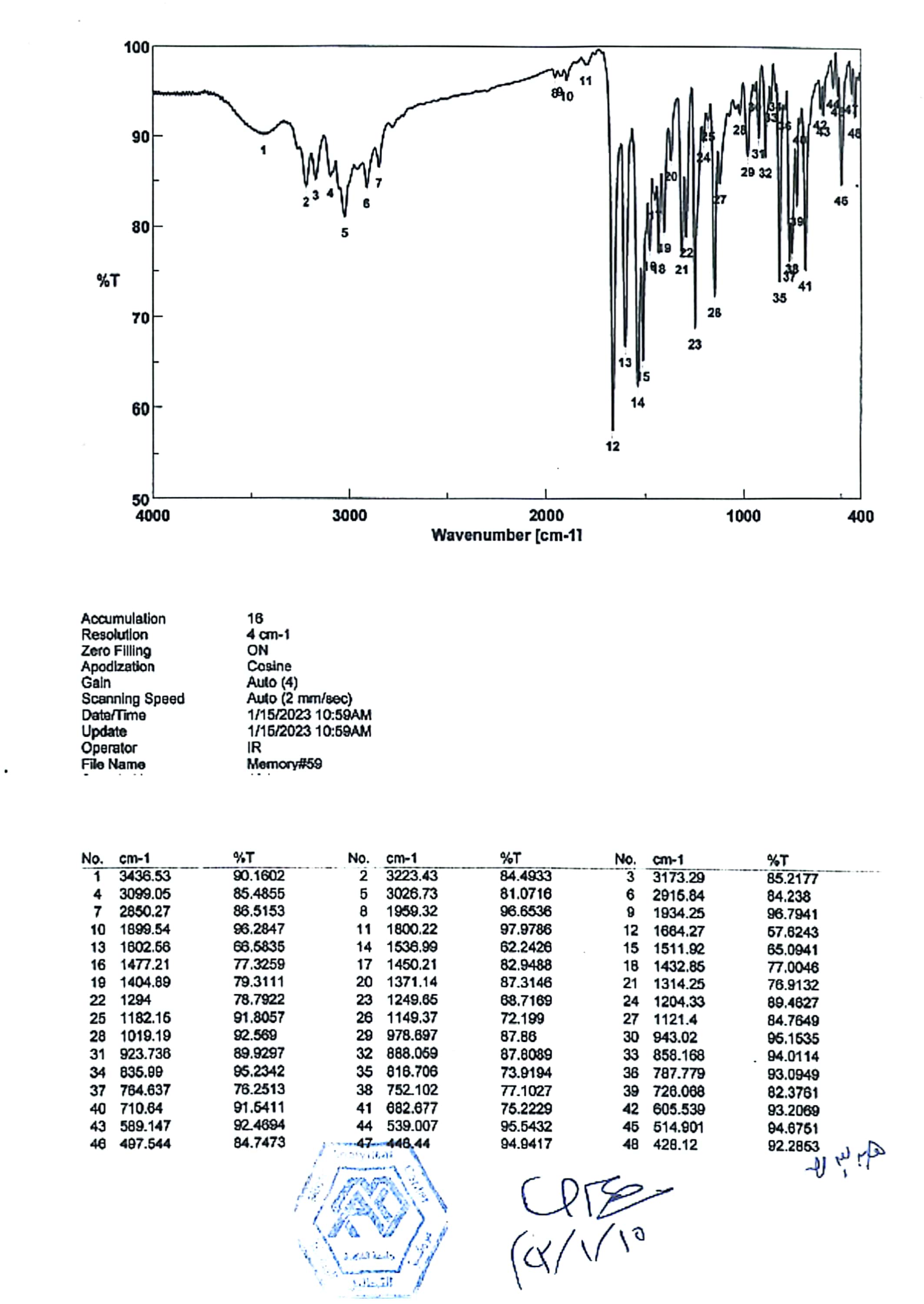
**

**Figure S31**. **Mass spectrum of compound 4e**

**
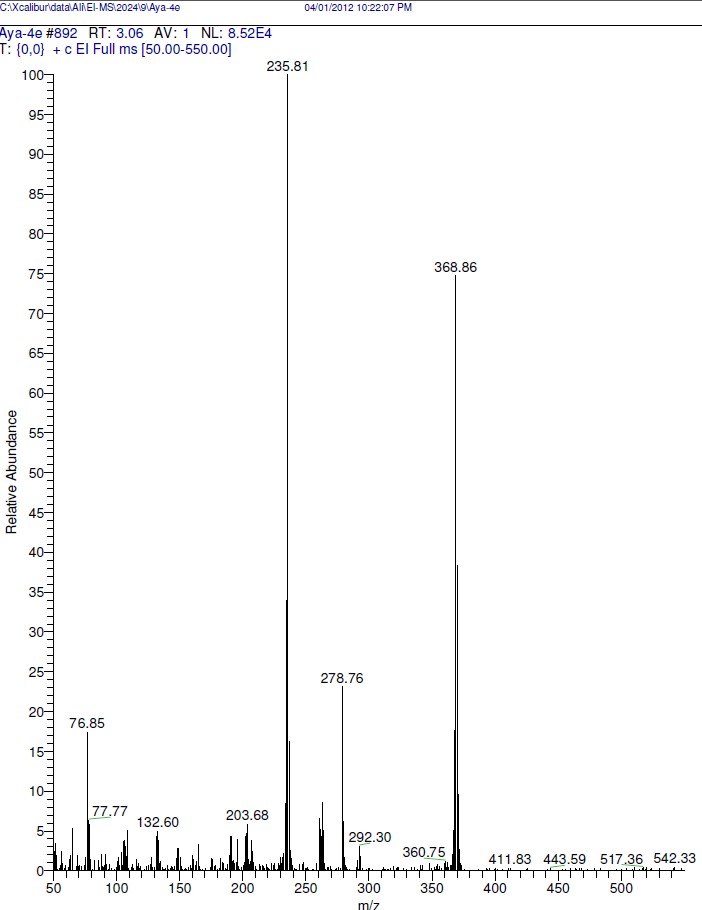
**

**Figure S32**. **^1^H NMR spectrum of compound 4f**

**
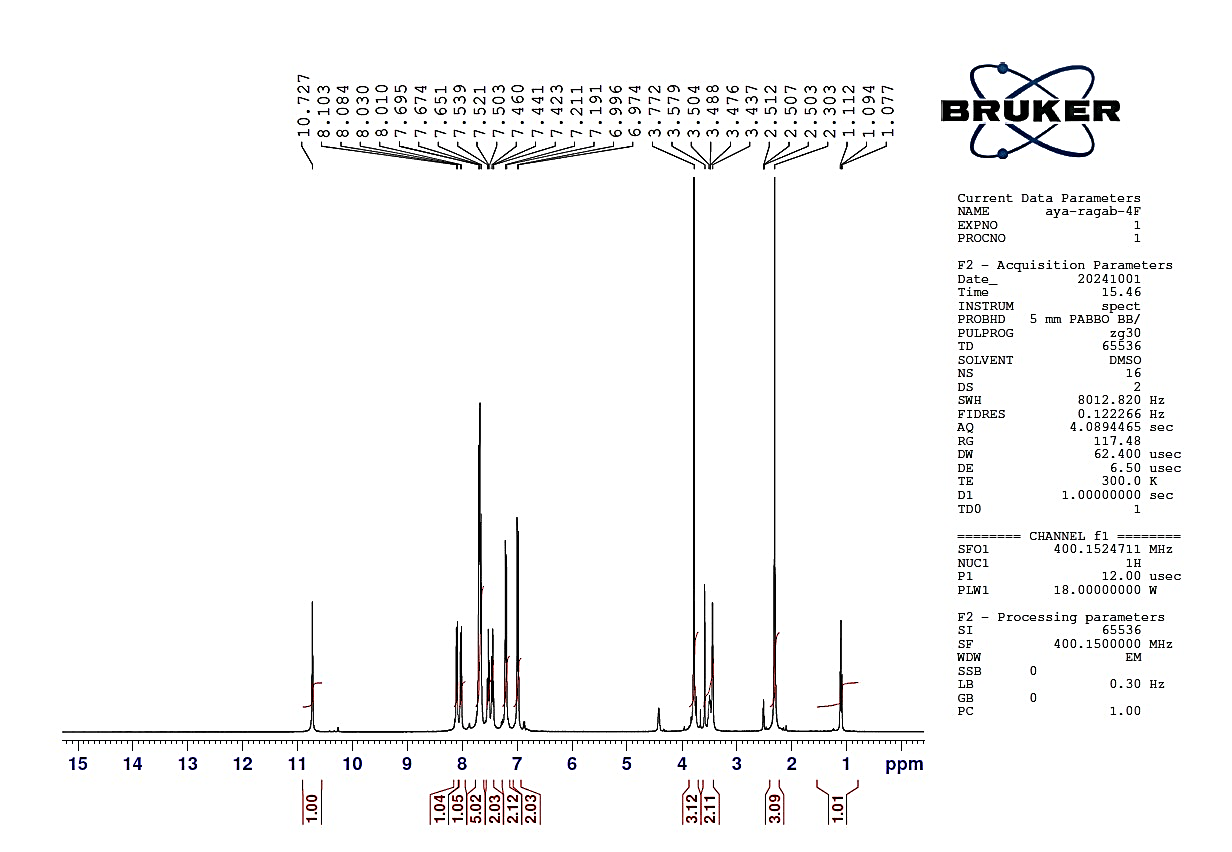
**

**Figure S33**. **^13^C NMR spectrum of compound 4f**

**
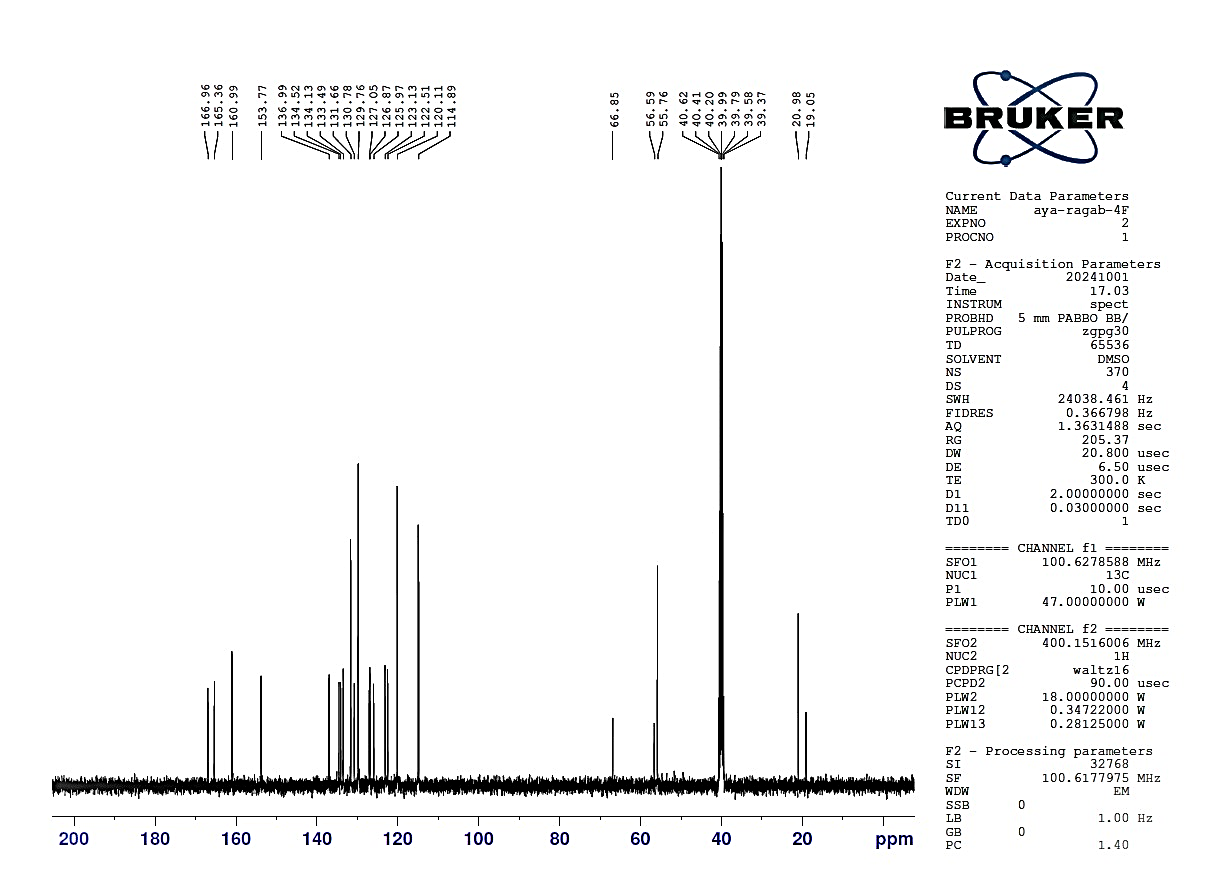
**

**Figure S34**. **IR spectrum of compound 4f**

**
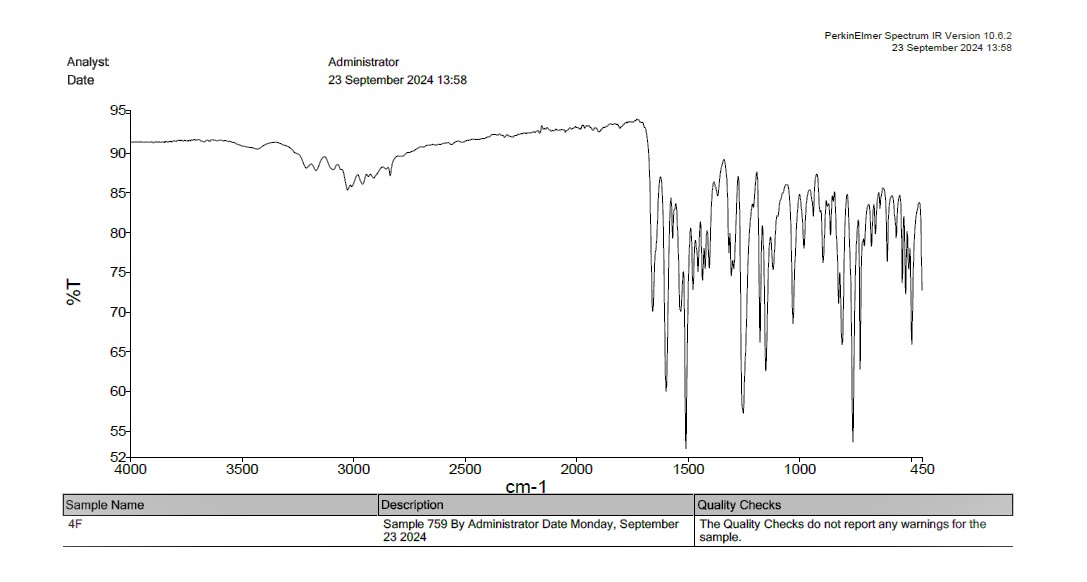
**

**Figure S35**. **^1^H NMR spectrum of compound 4g**

**
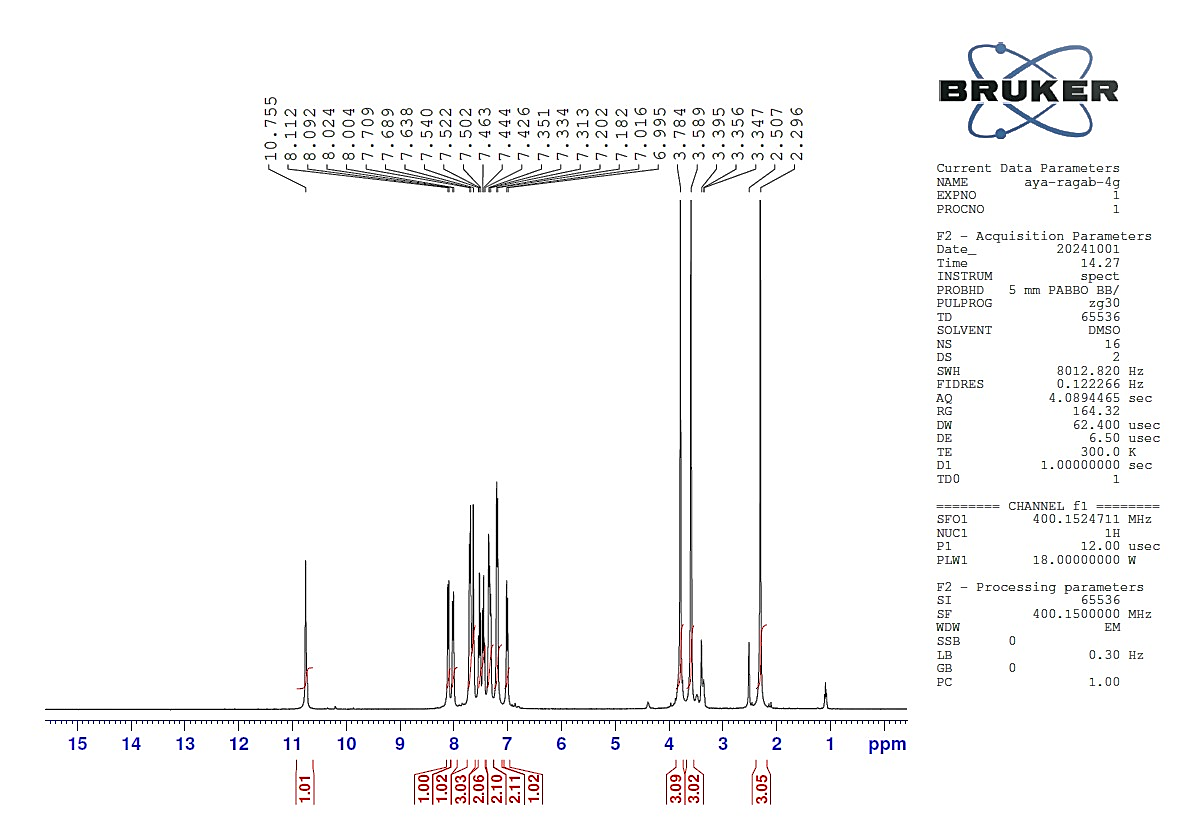
**

**Figure S36**. **^13^C NMR spectrum of compound 4g**

**
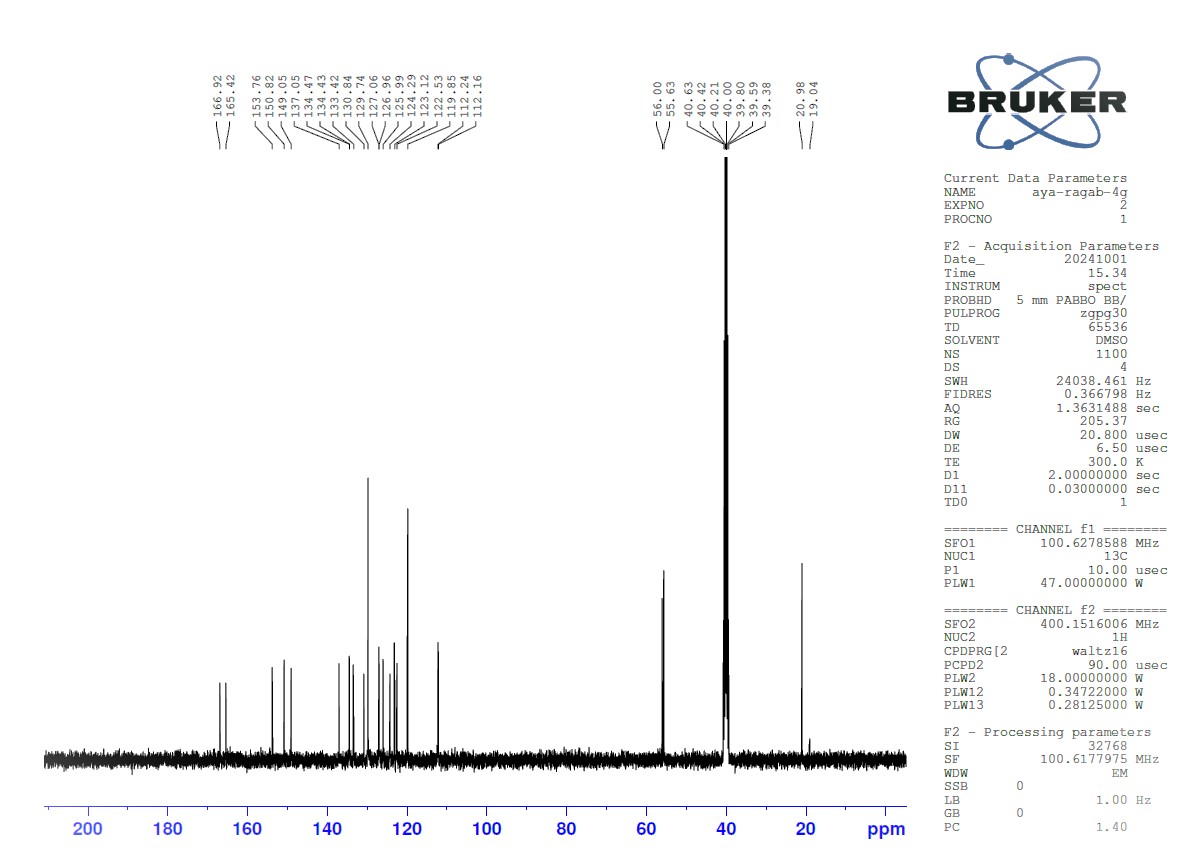
**

**Figure S37**. **IR spectrum of compound 4g**

**
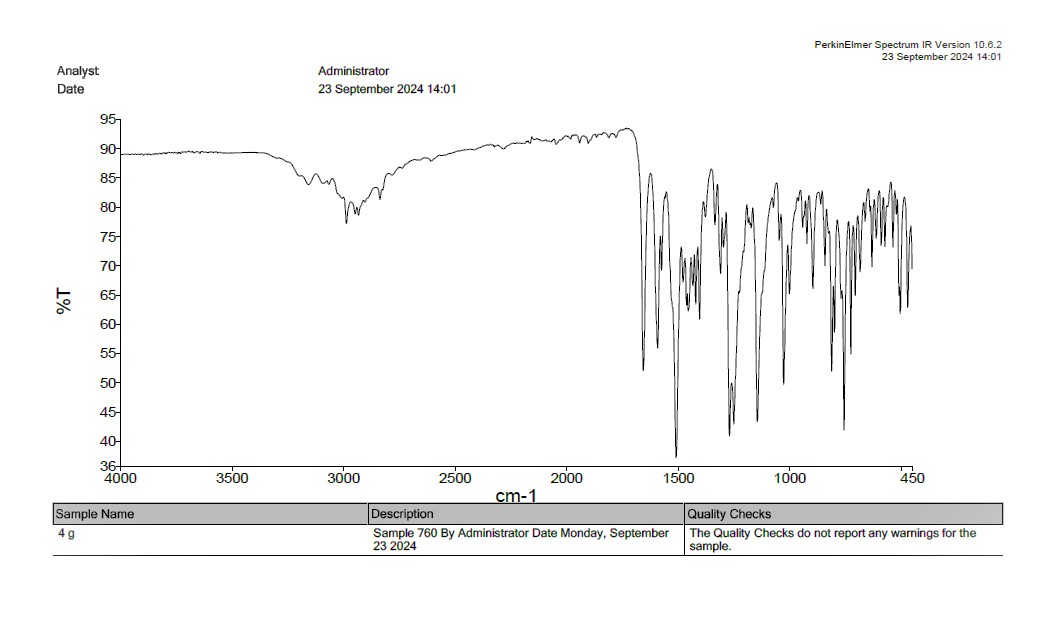
**

**Figure S38**. **^1^H NMR spectrum of compound 4h**

**
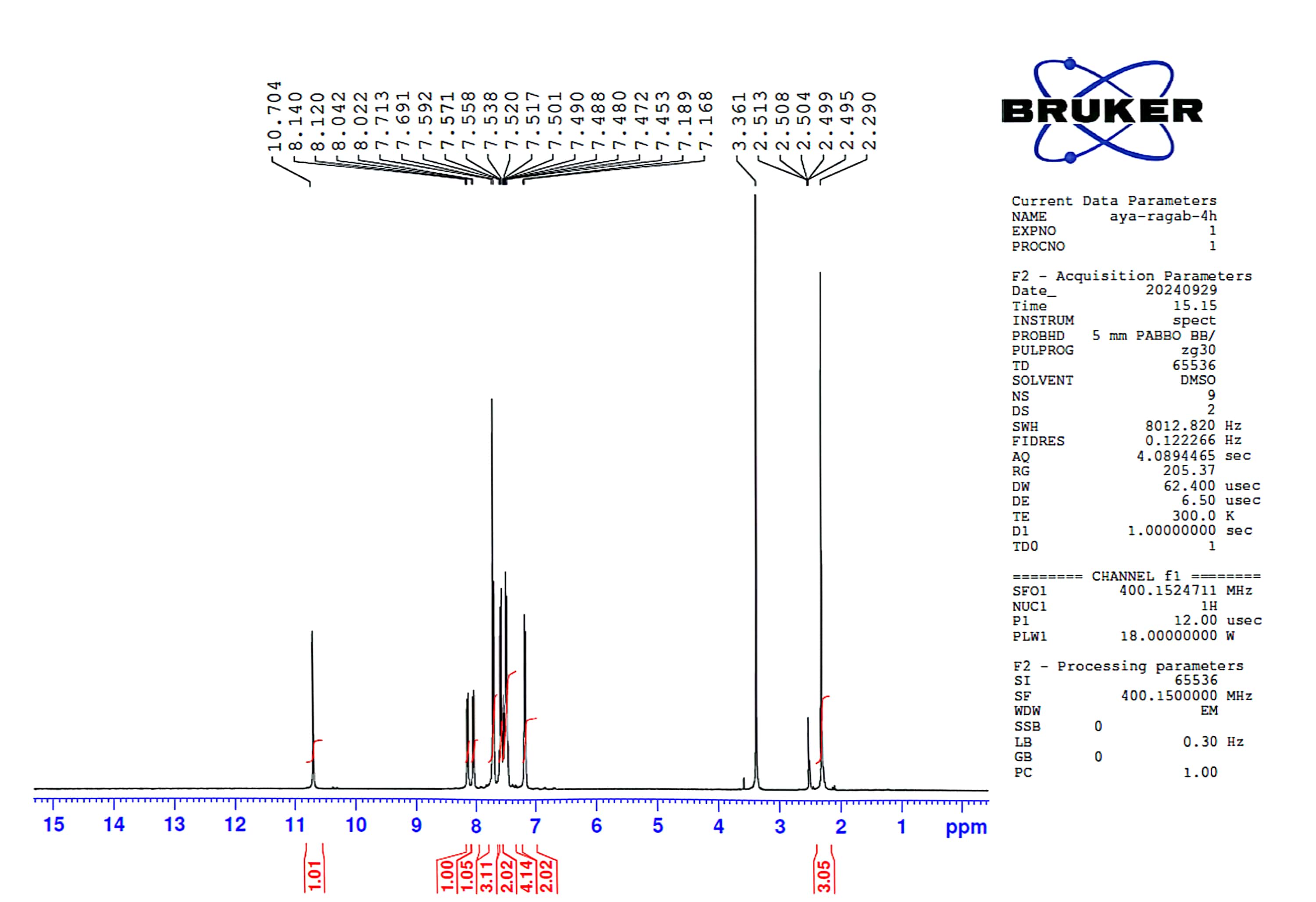
**

**Figure S39**. **^13^C NMR spectrum of compound 4h**

**
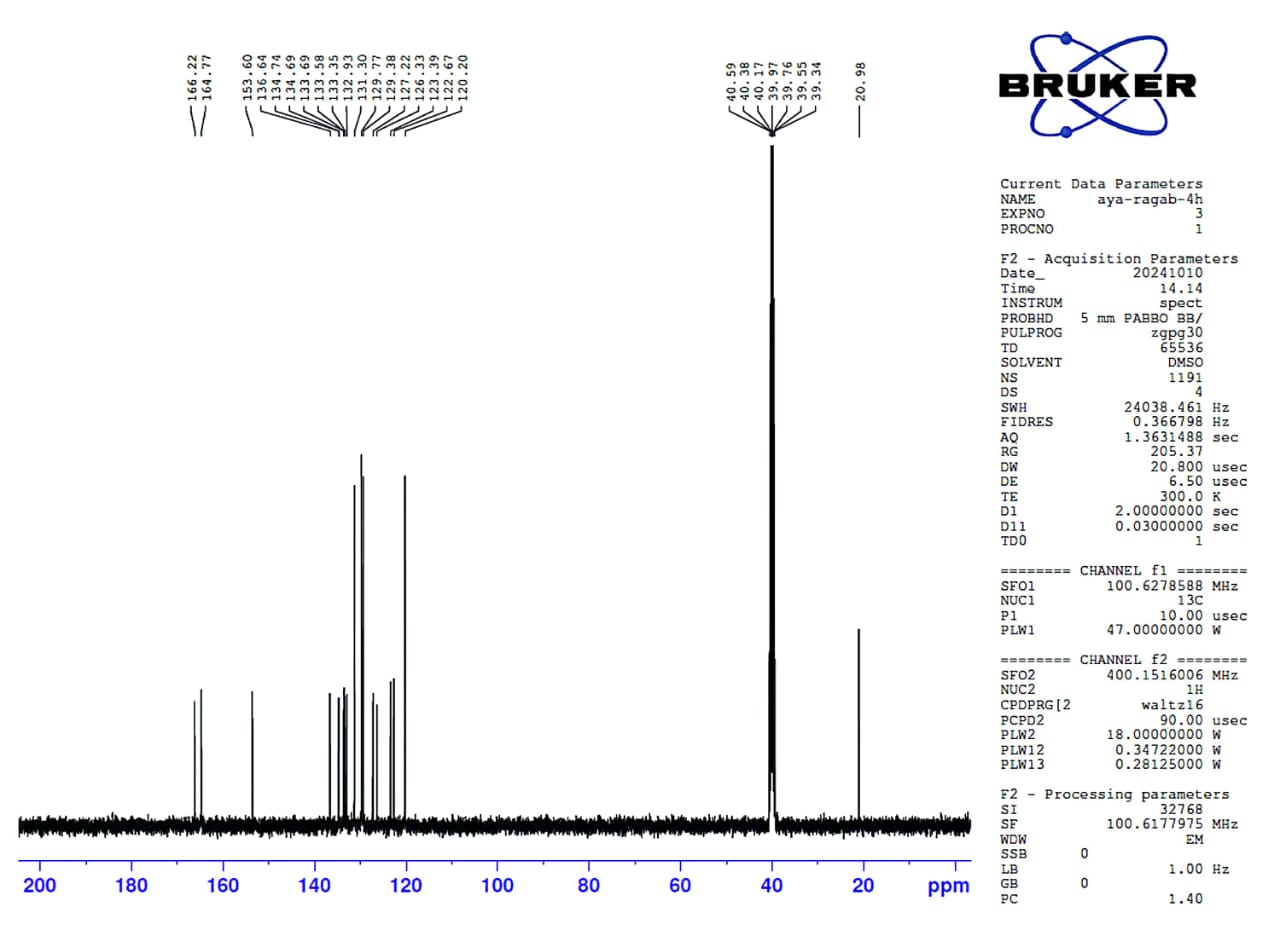
**

**Figure S40**. **IR spectrum of compound 4h**

**
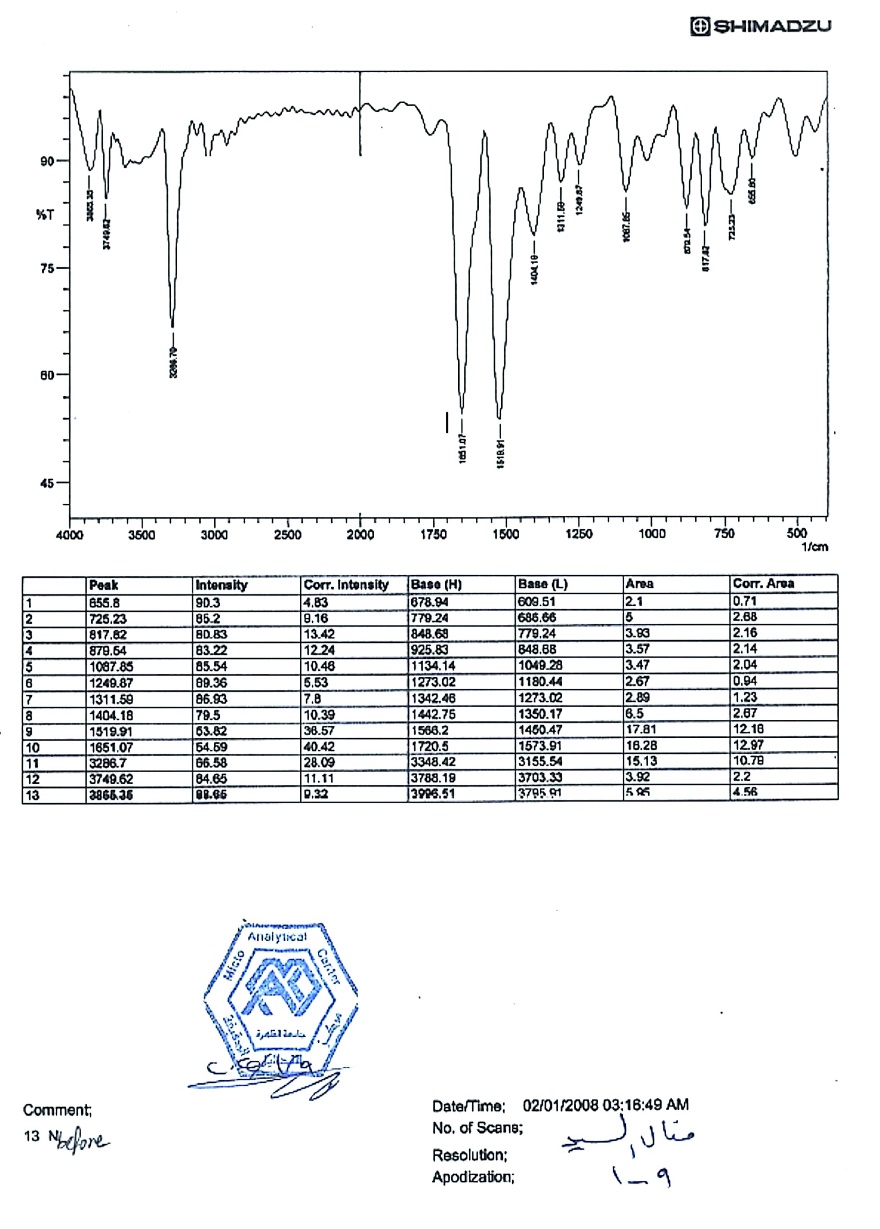
**

**Figure S41**. **^13^C NMR spectrum of compound 4i**

**
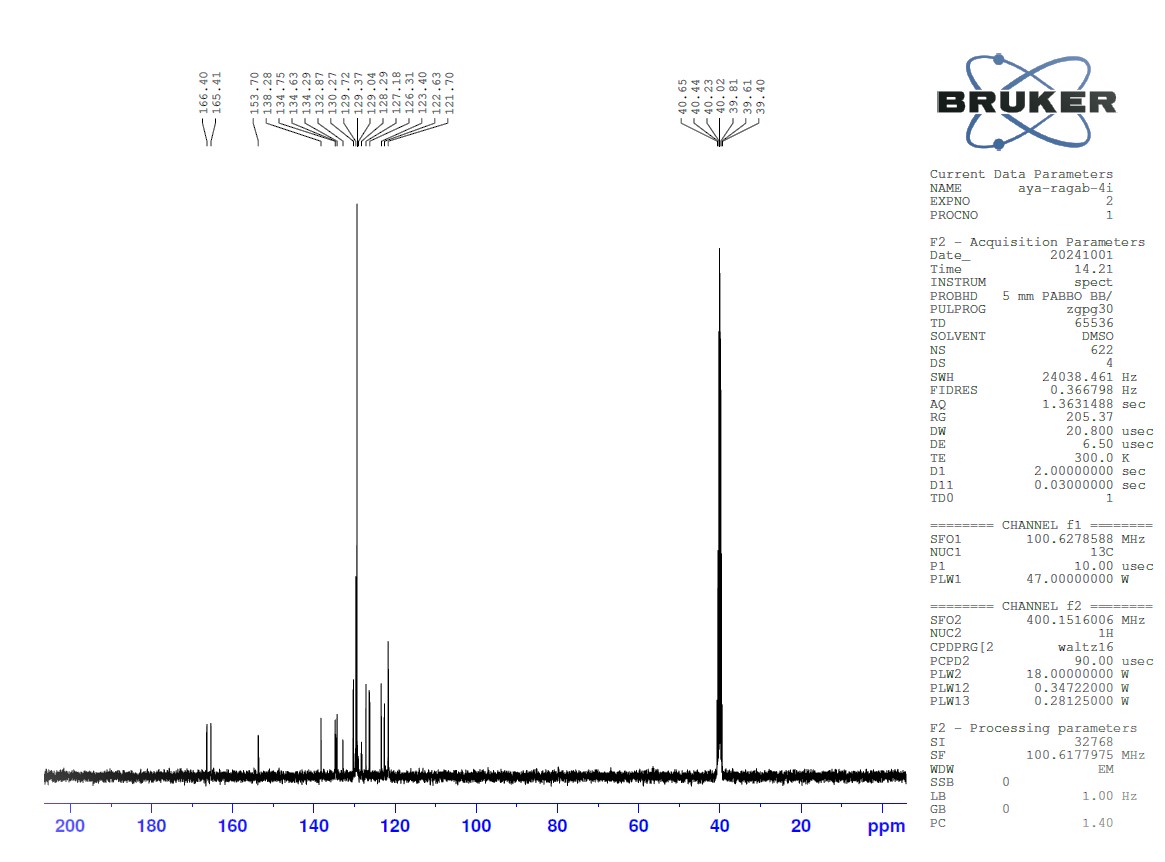
**

**Figure S42**. **IR spectrum of compound 4i**

**
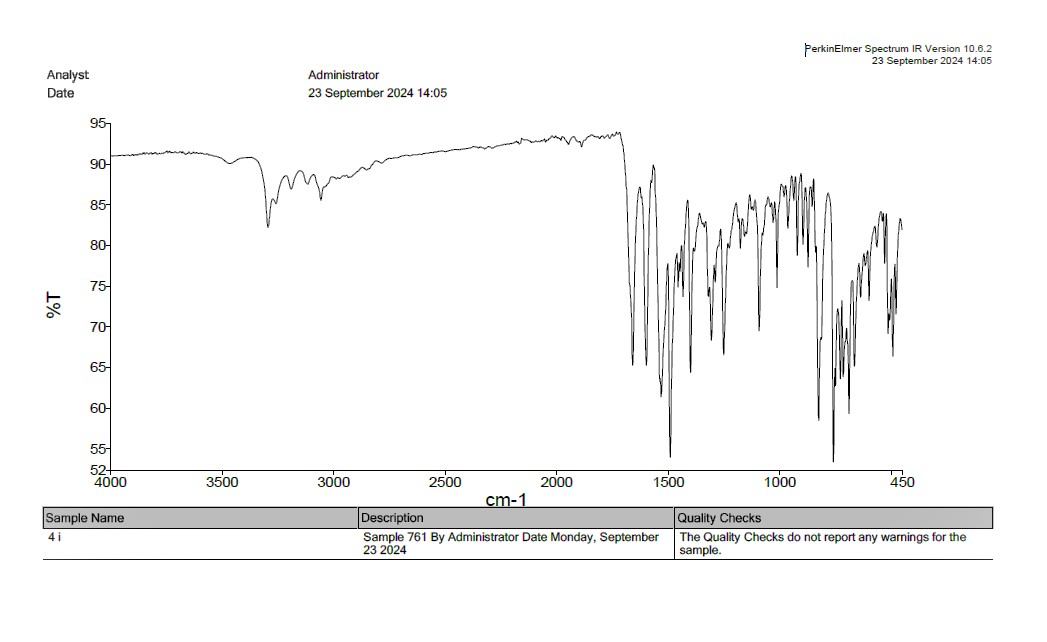
**

**Figure S43**. **^1^H NMR spectrum of compound 4j**

**
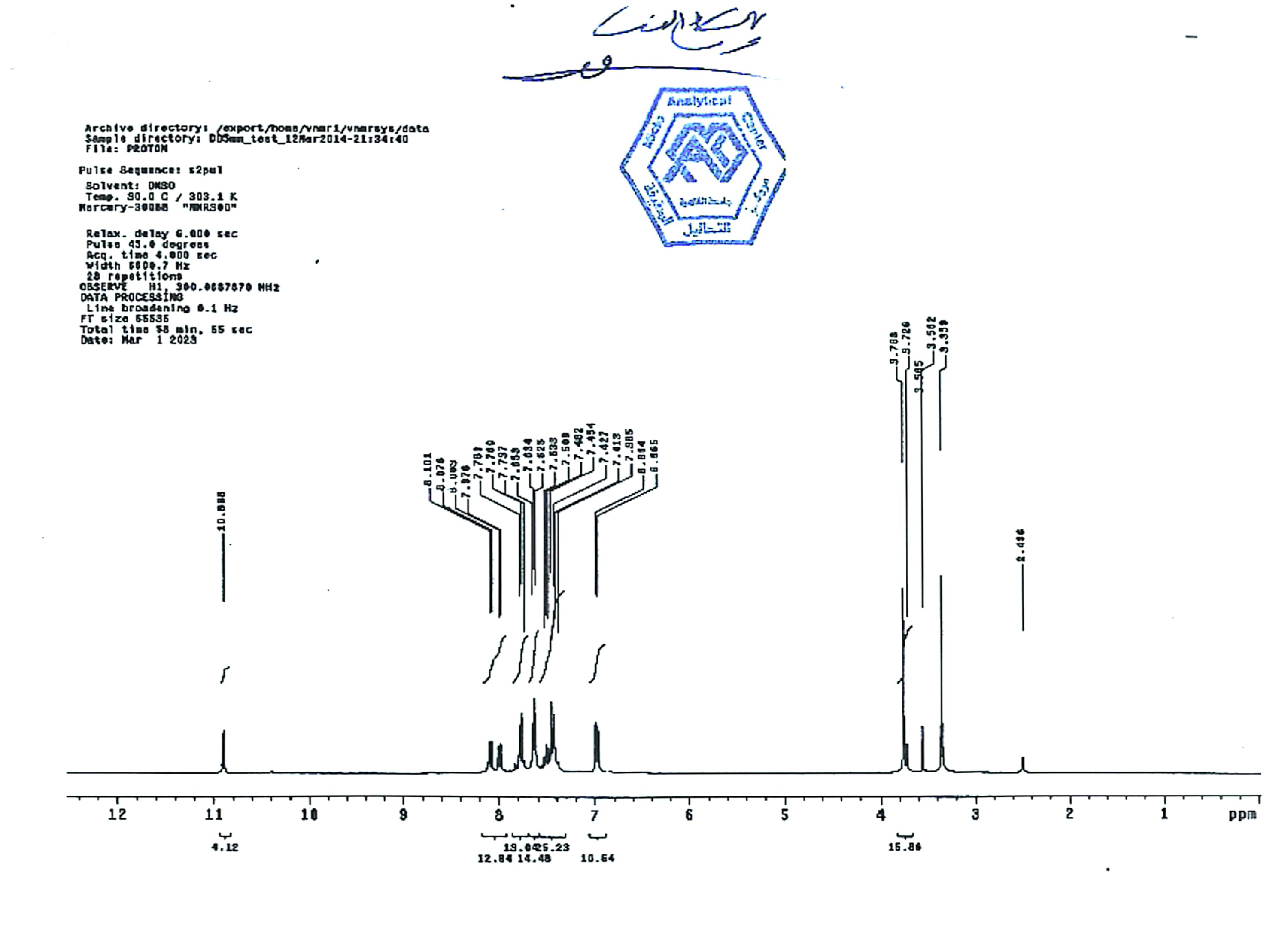
**

**Figure S44**. **^13^C NMR spectrum of compound 4j**

**
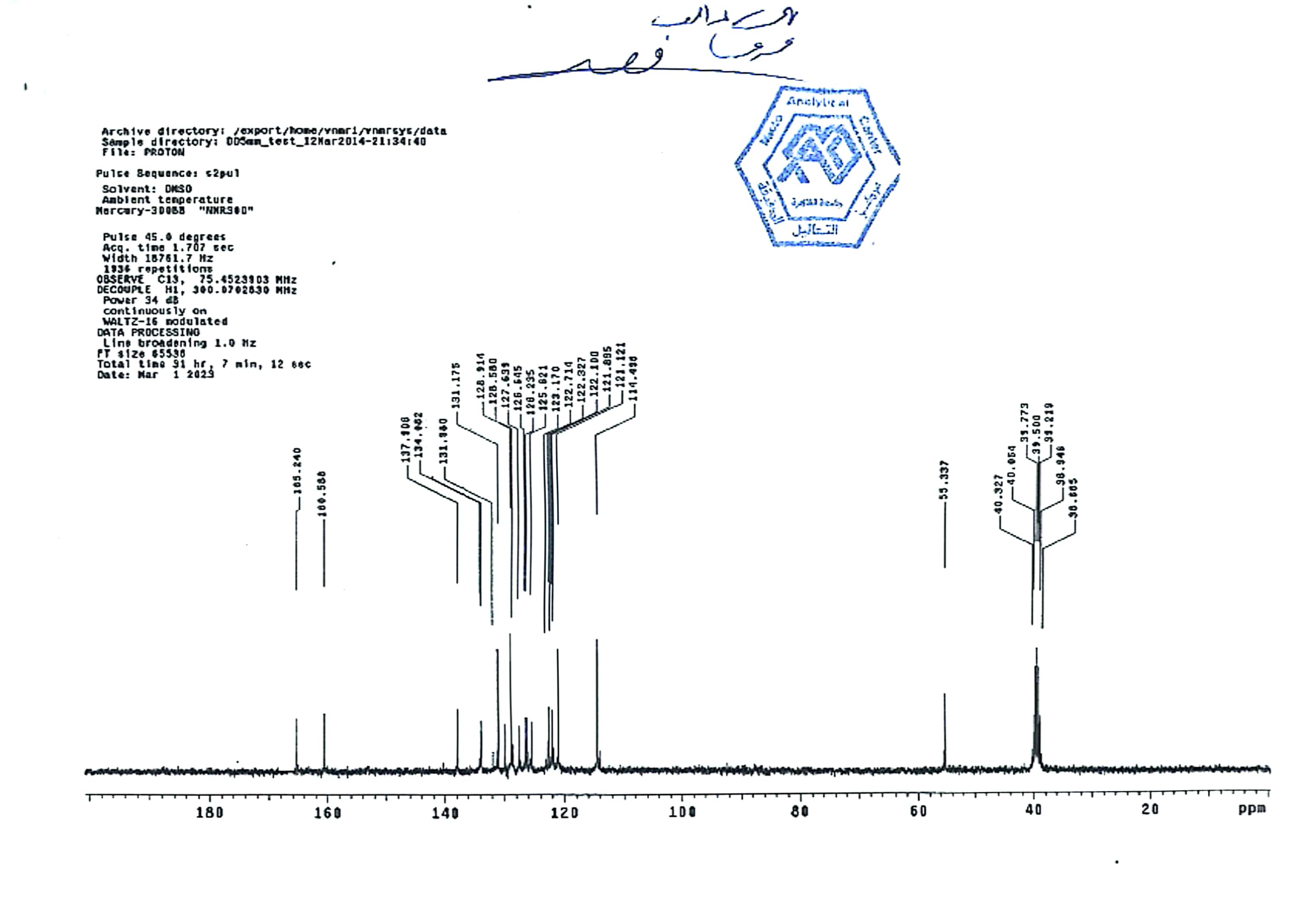
**

**Figure S45**. **IR spectrum of compound 4j**

**
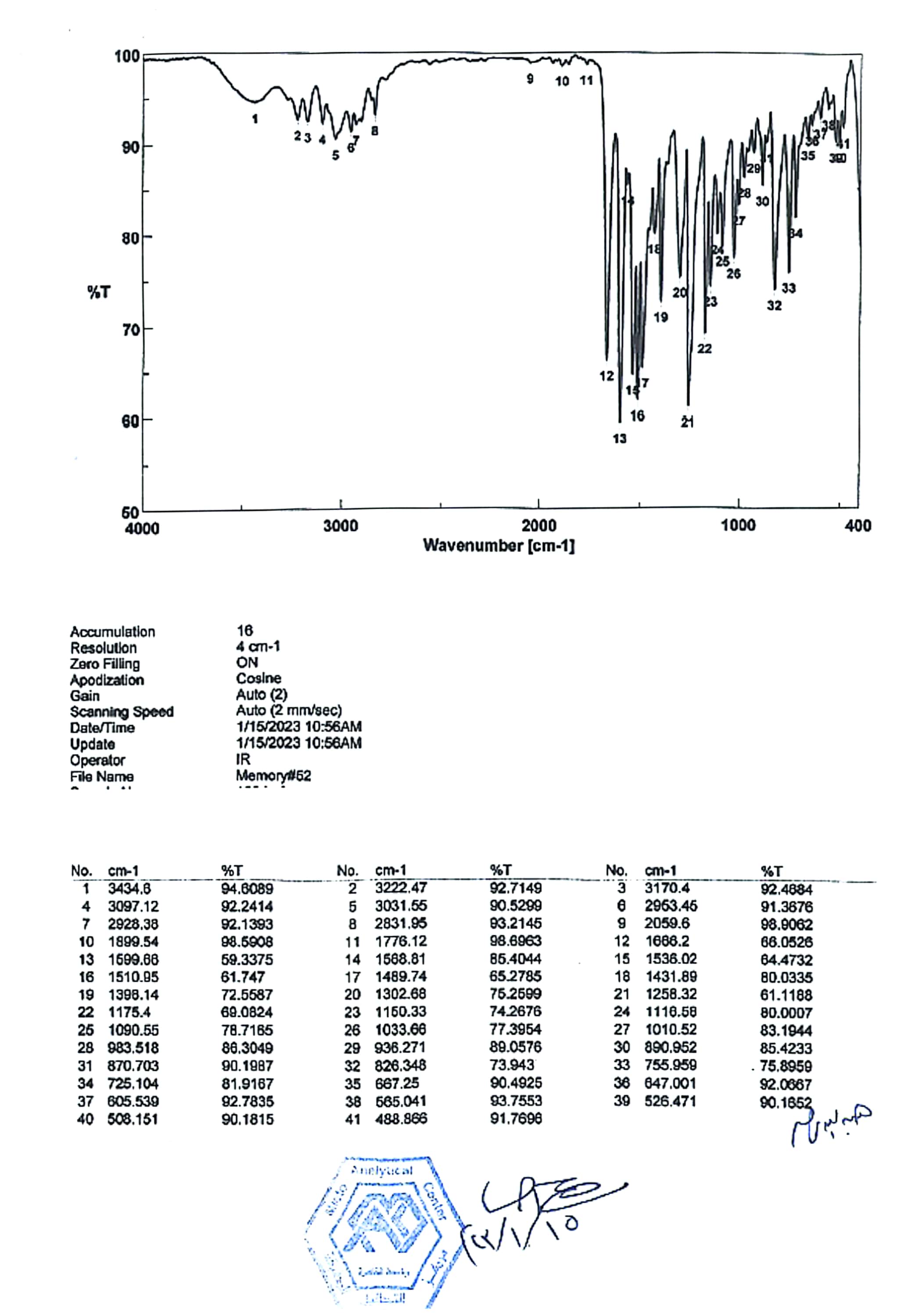
**

**Figure S46**. **^1^H NMR spectrum of compound 4k**

**
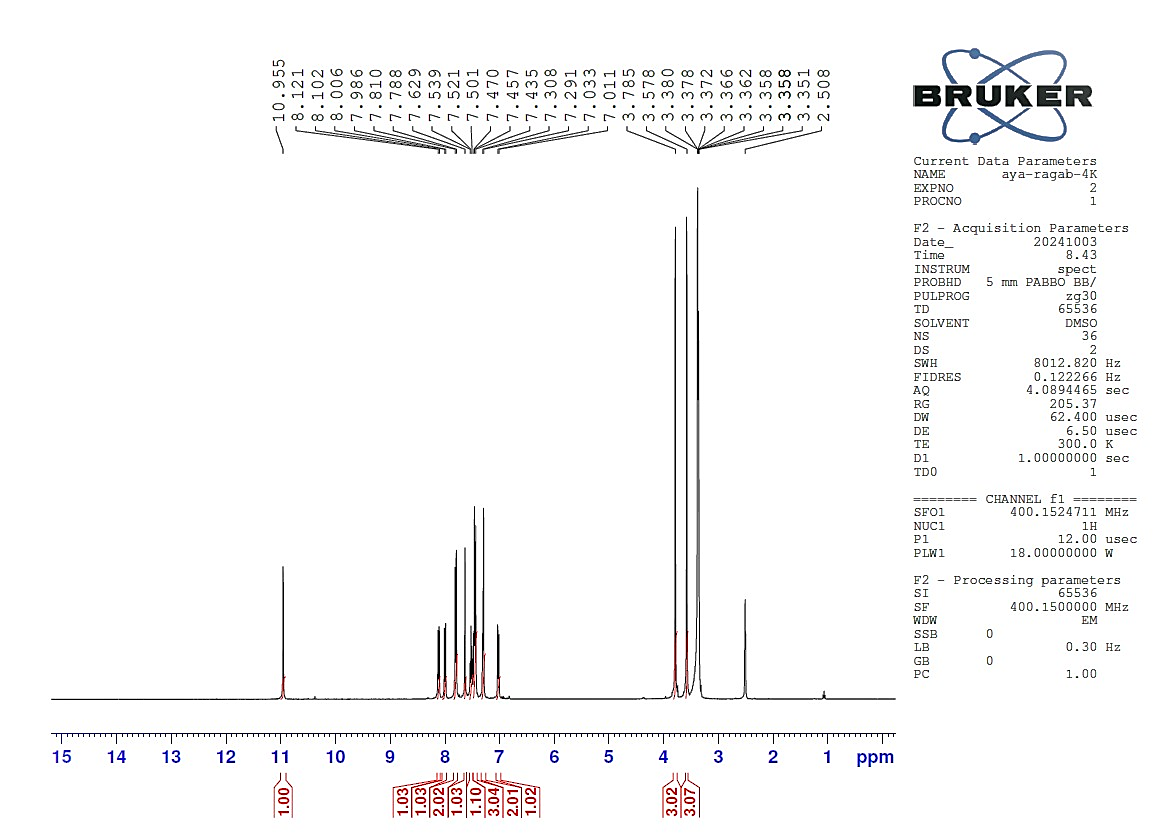
**

**Figure S47**. **^13^C NMR spectrum of compound 4k**

**
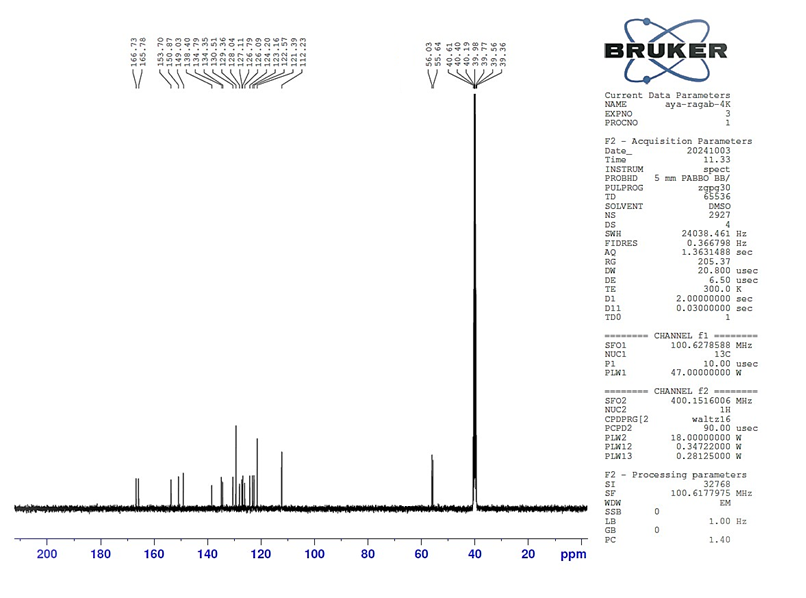
**

**Figure S48**. **IR spectrum of compound 4k**

**
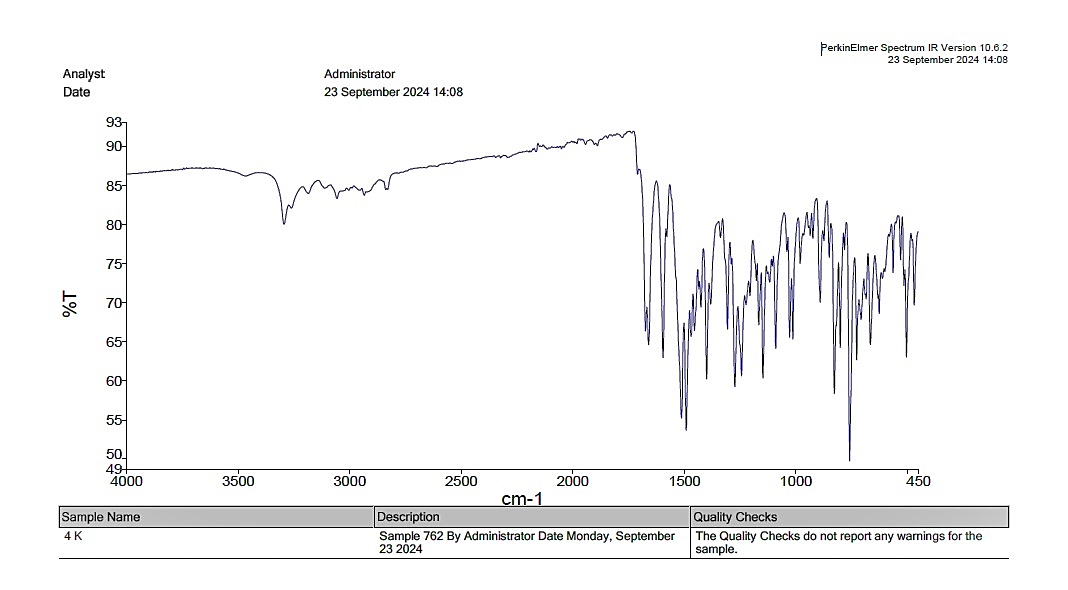
**

**Figure S49**. **^1^H NMR spectrum of compound 4l**

**
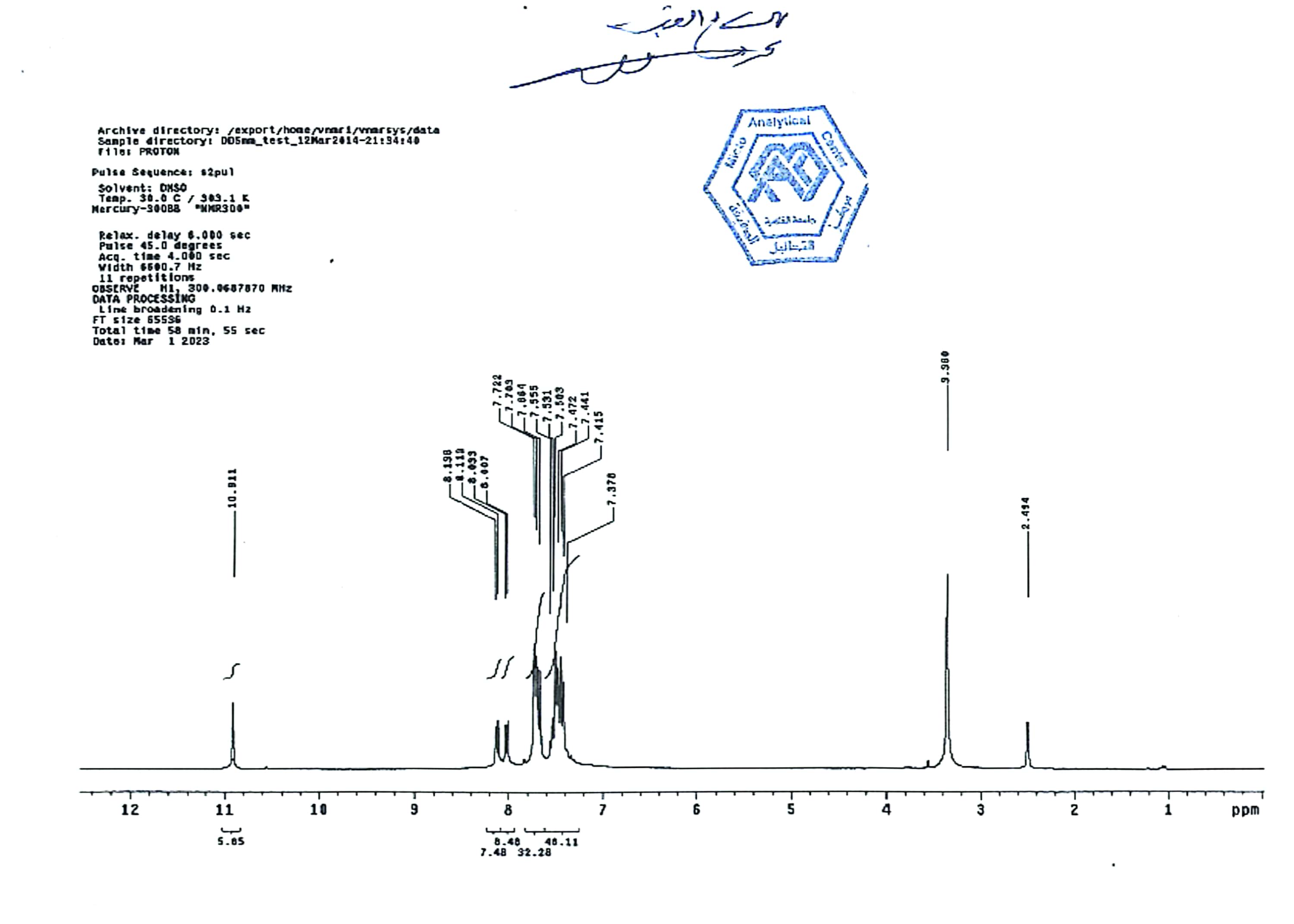
**

**Figure S50**. **^13^C NMR spectrum of compound 4l**

**
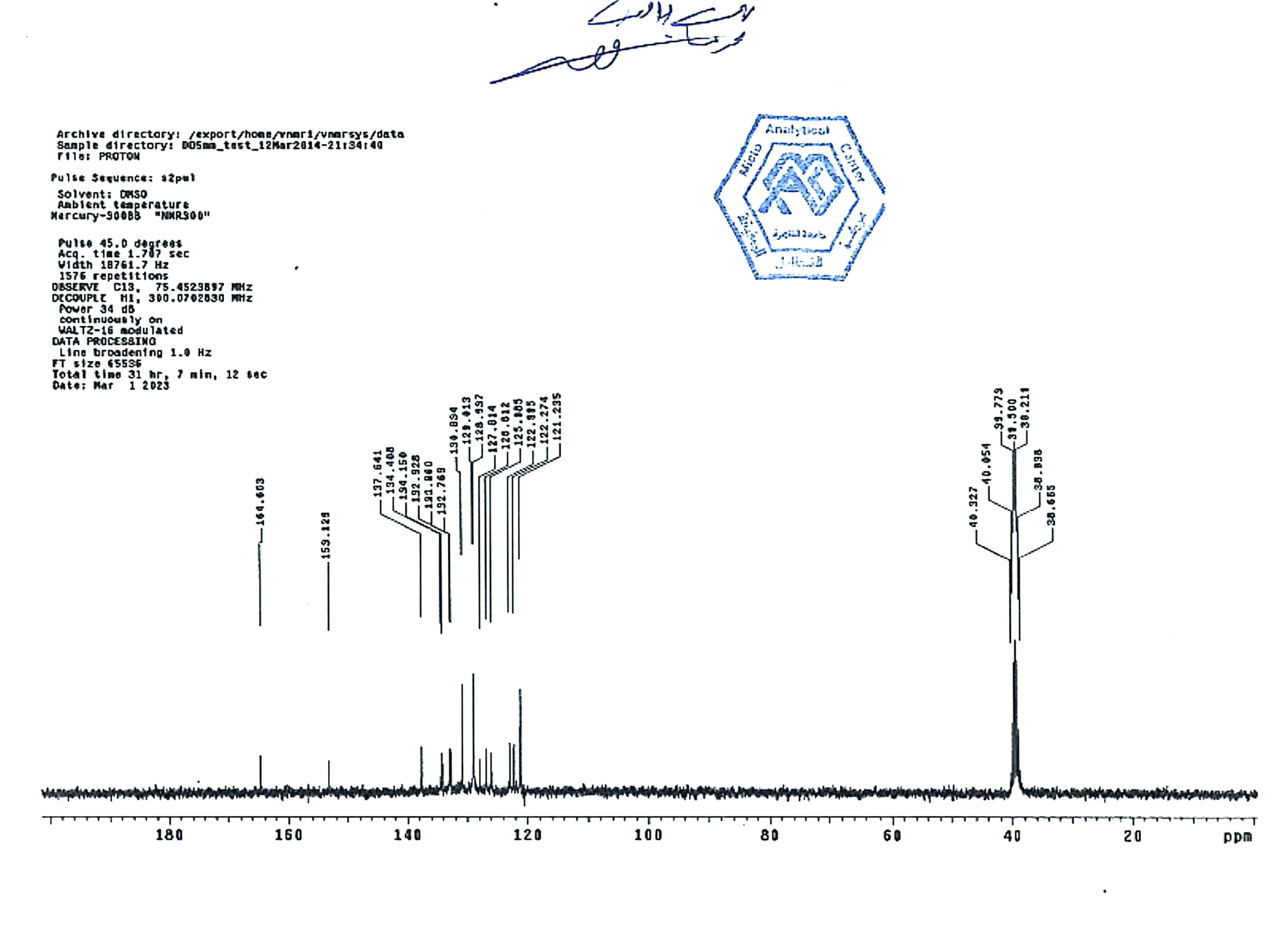
**

**Figure S51**. **IR spectrum of compound 4l**

**
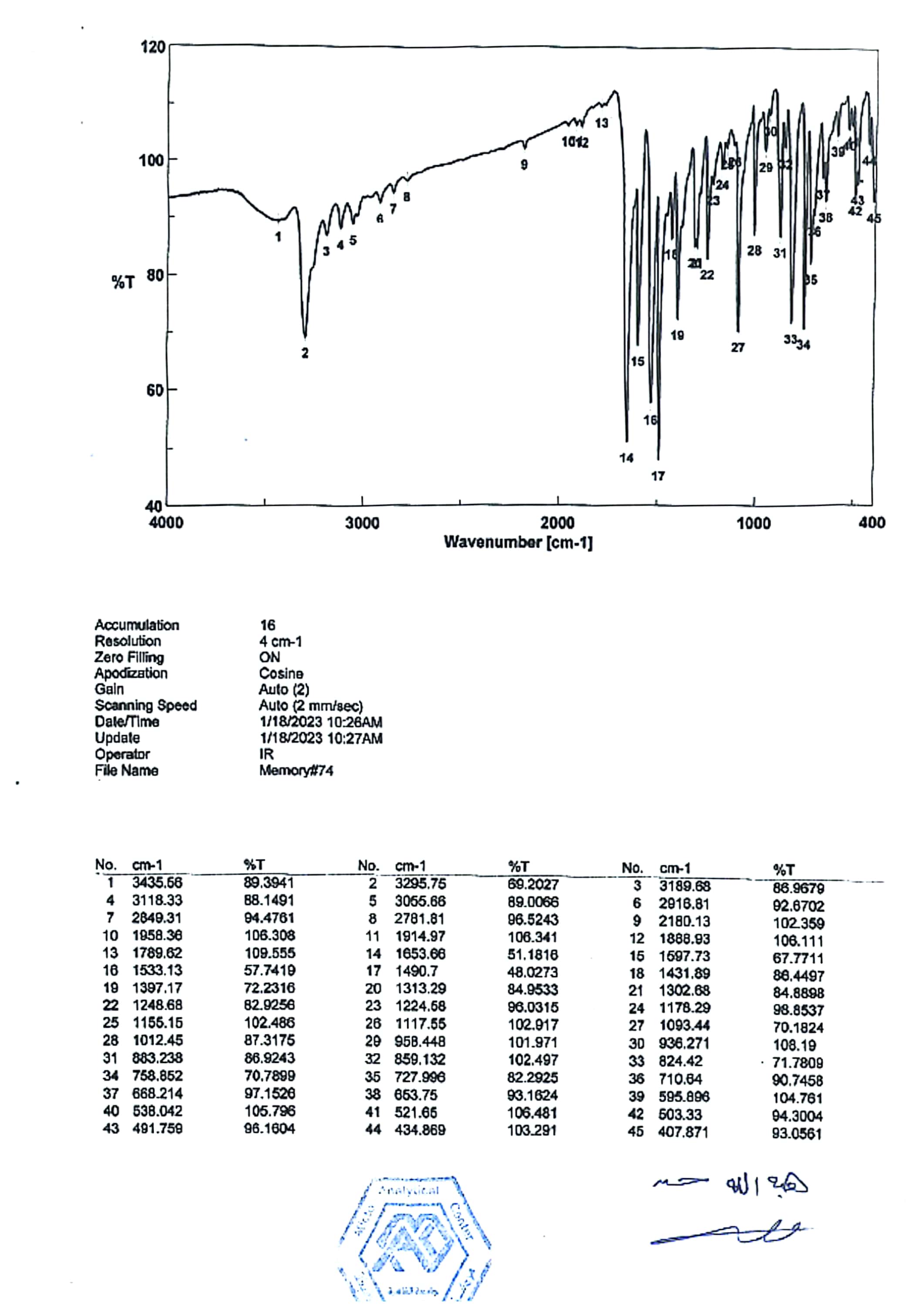
**

**Figure S52**. **Mass spectrum of compound 4l**

**
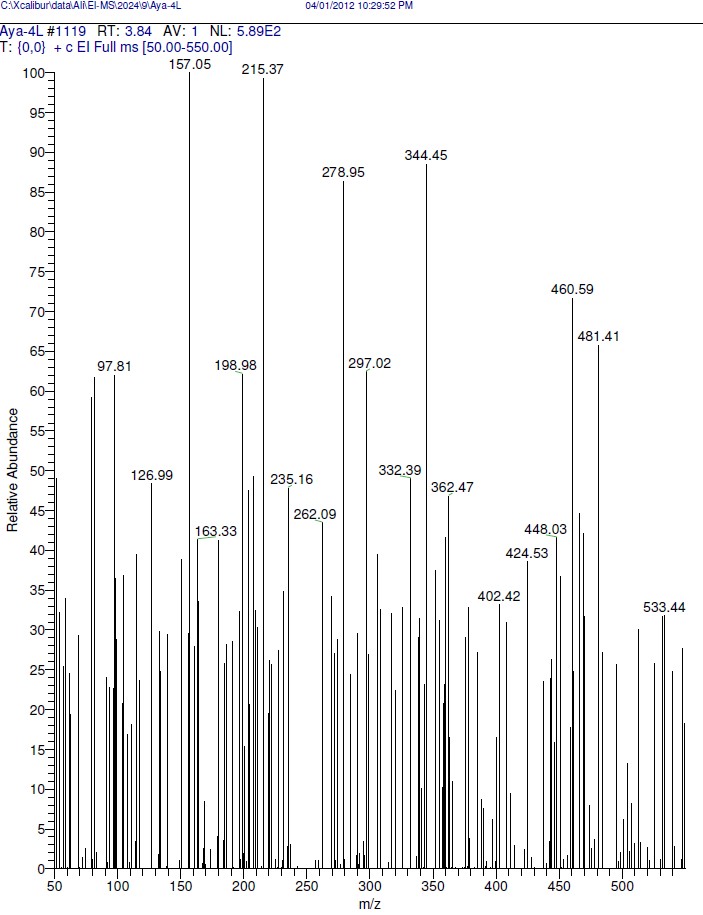
**

**Figure S53**. **^1^H NMR spectrum of compound 4m**

**
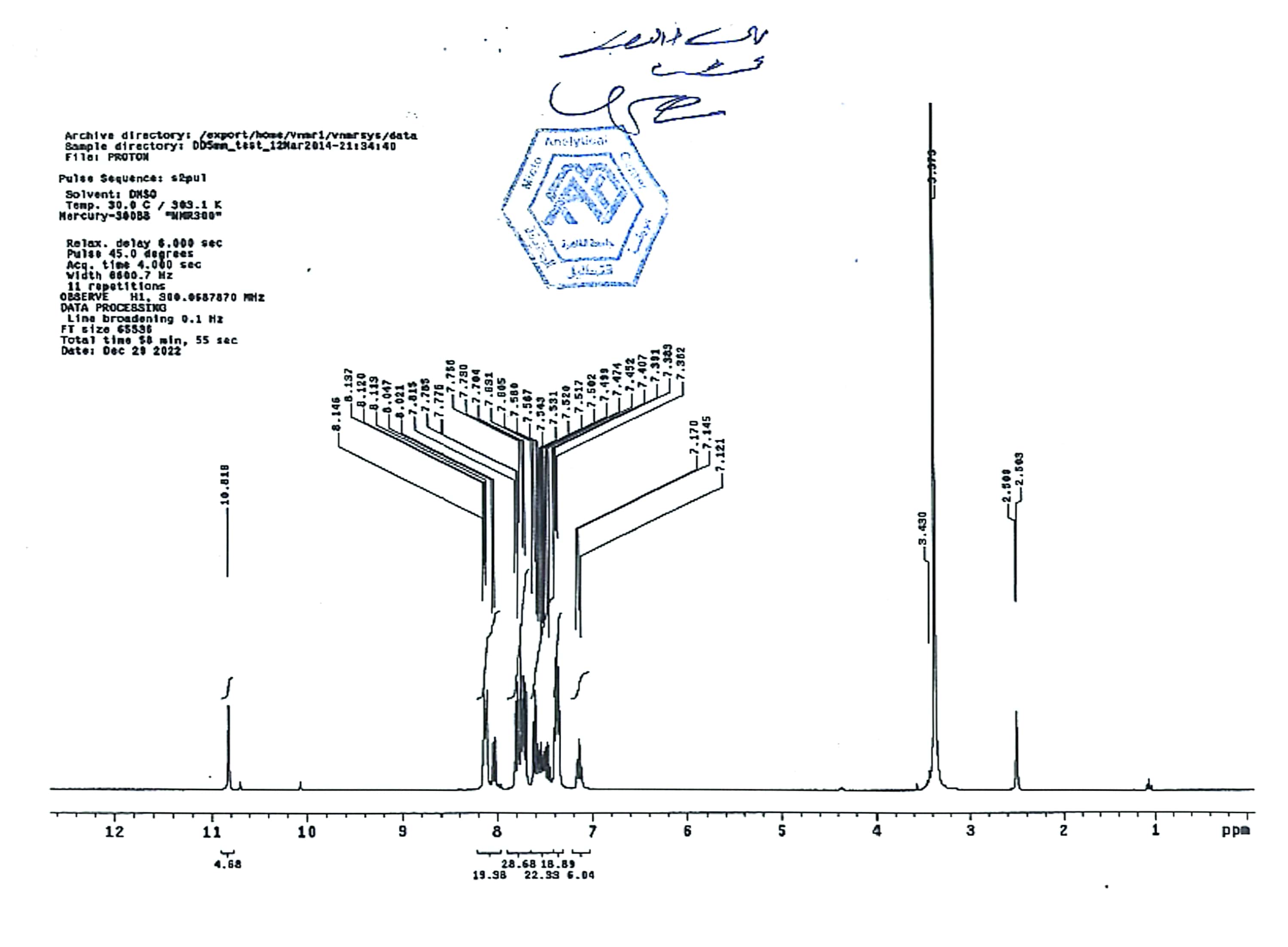
**

**Figure S54**. **^13^C NMR spectrum of compound 4m**

**
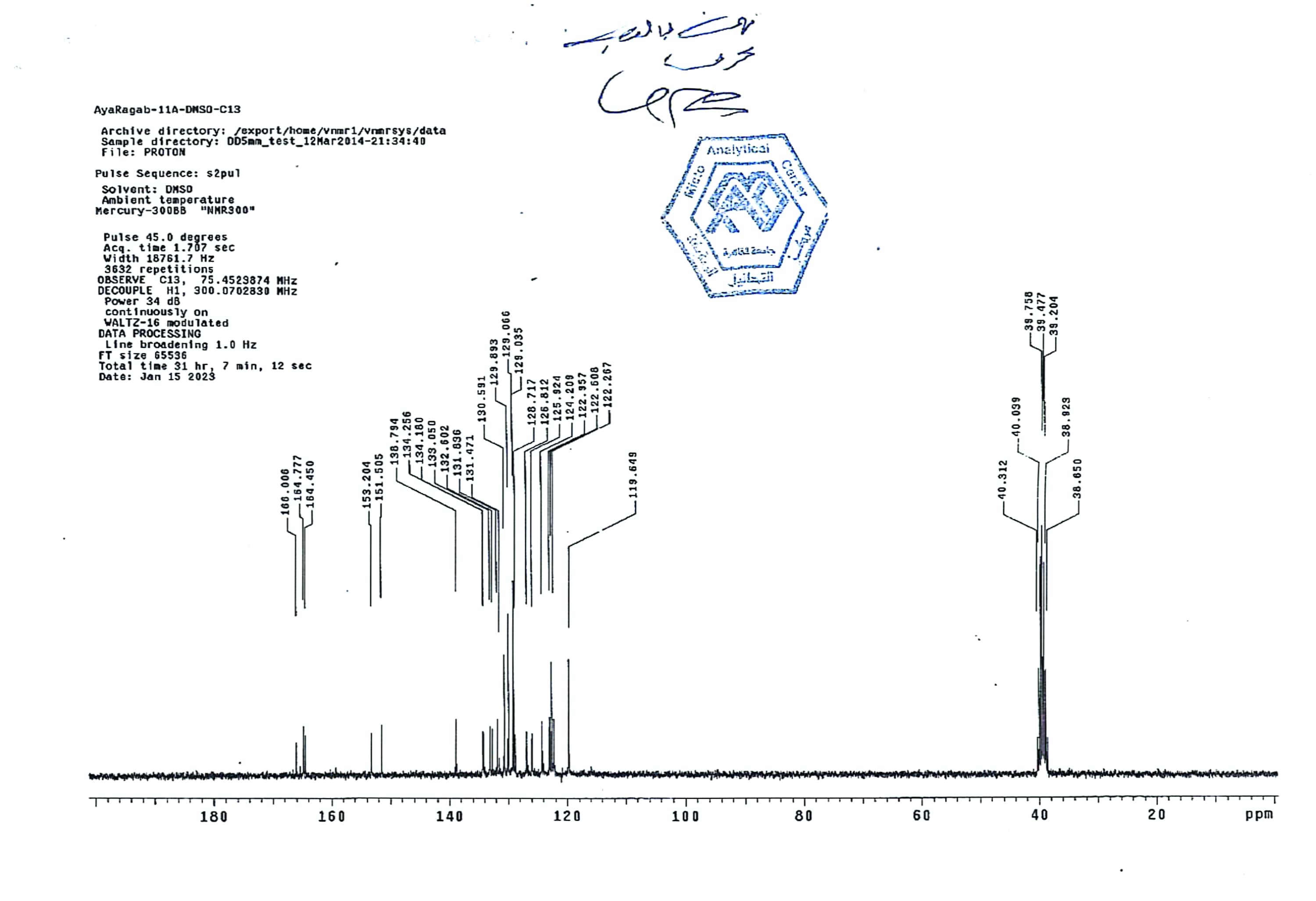
**

**Figure S55**. **^1^H NMR spectrum of compound 4n**

**
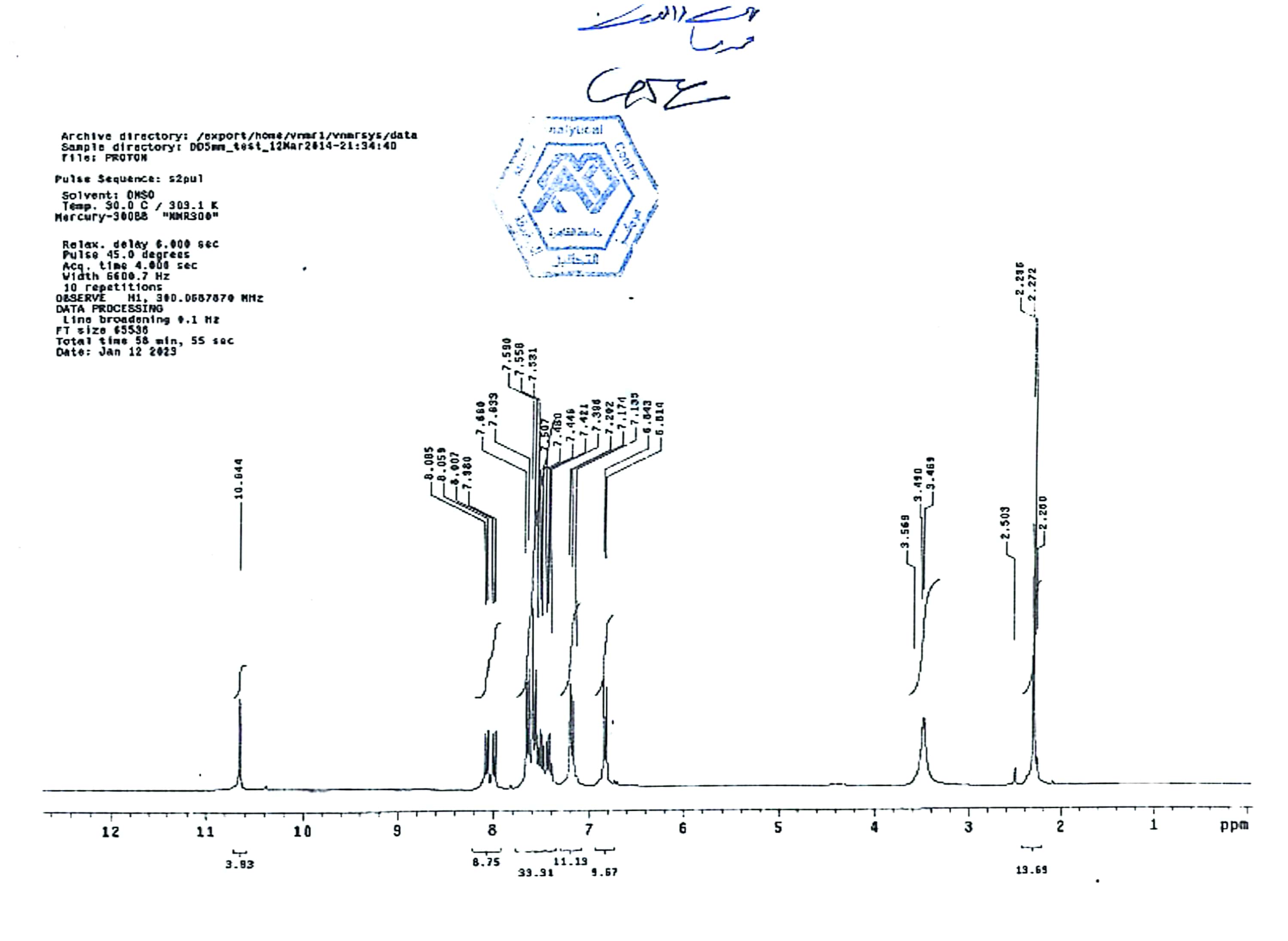
**

**Figure S56**. **^13^C NMR spectrum of compound 4n**

**
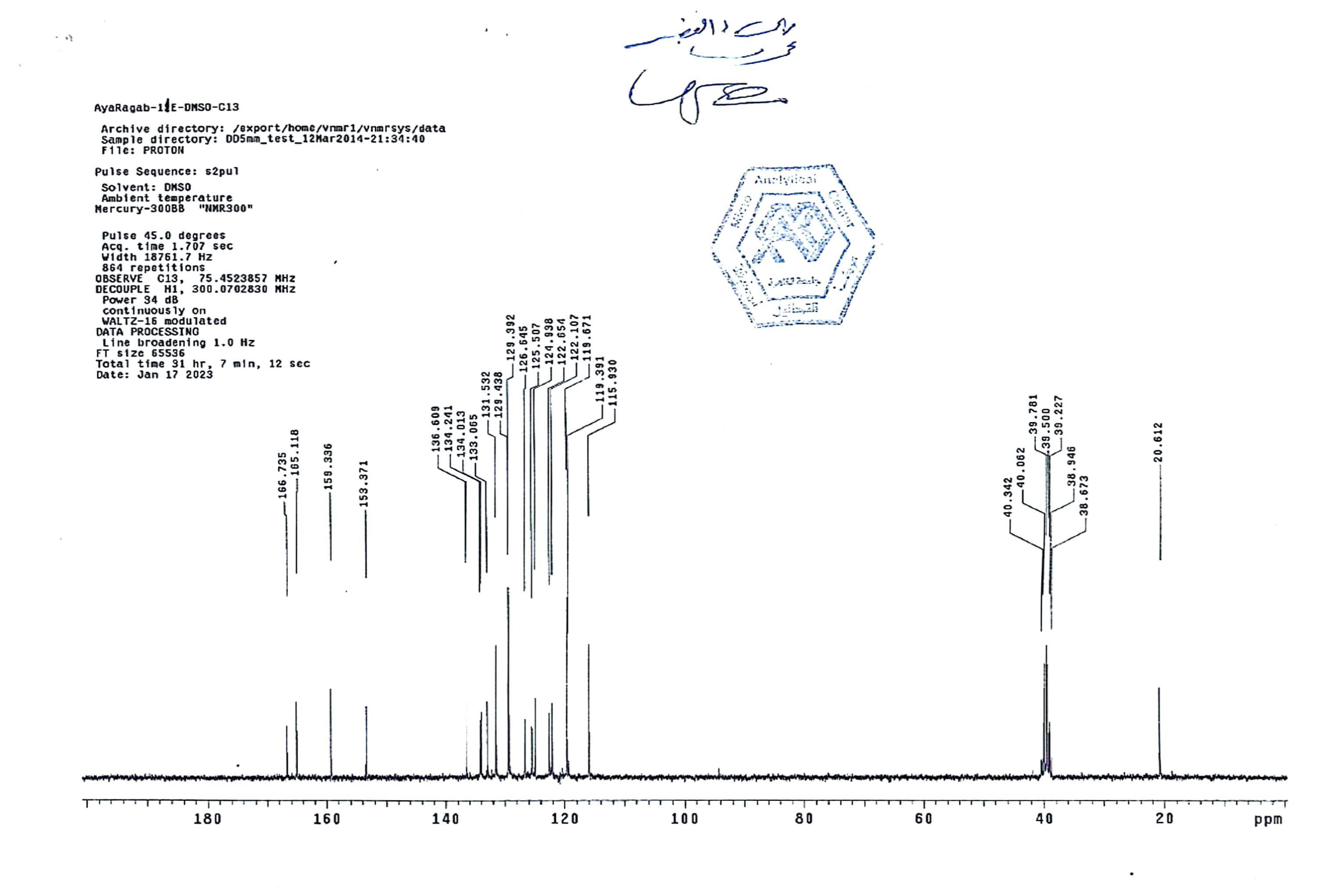
**

**Figure S57**. **IR spectrum of compound 4n**

**
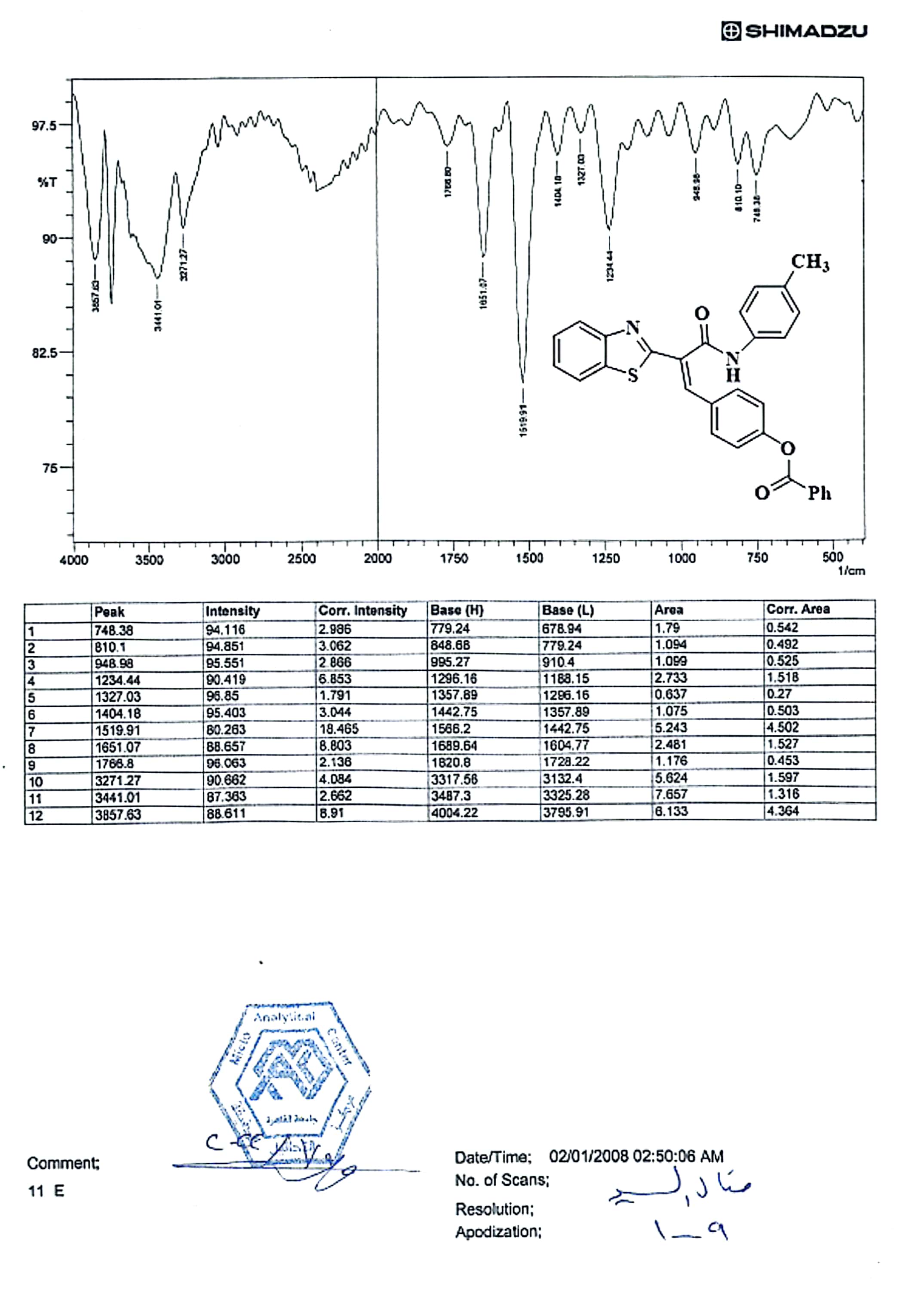
**

**Figure S58**. **^1^H NMR spectrum of compound 6a**


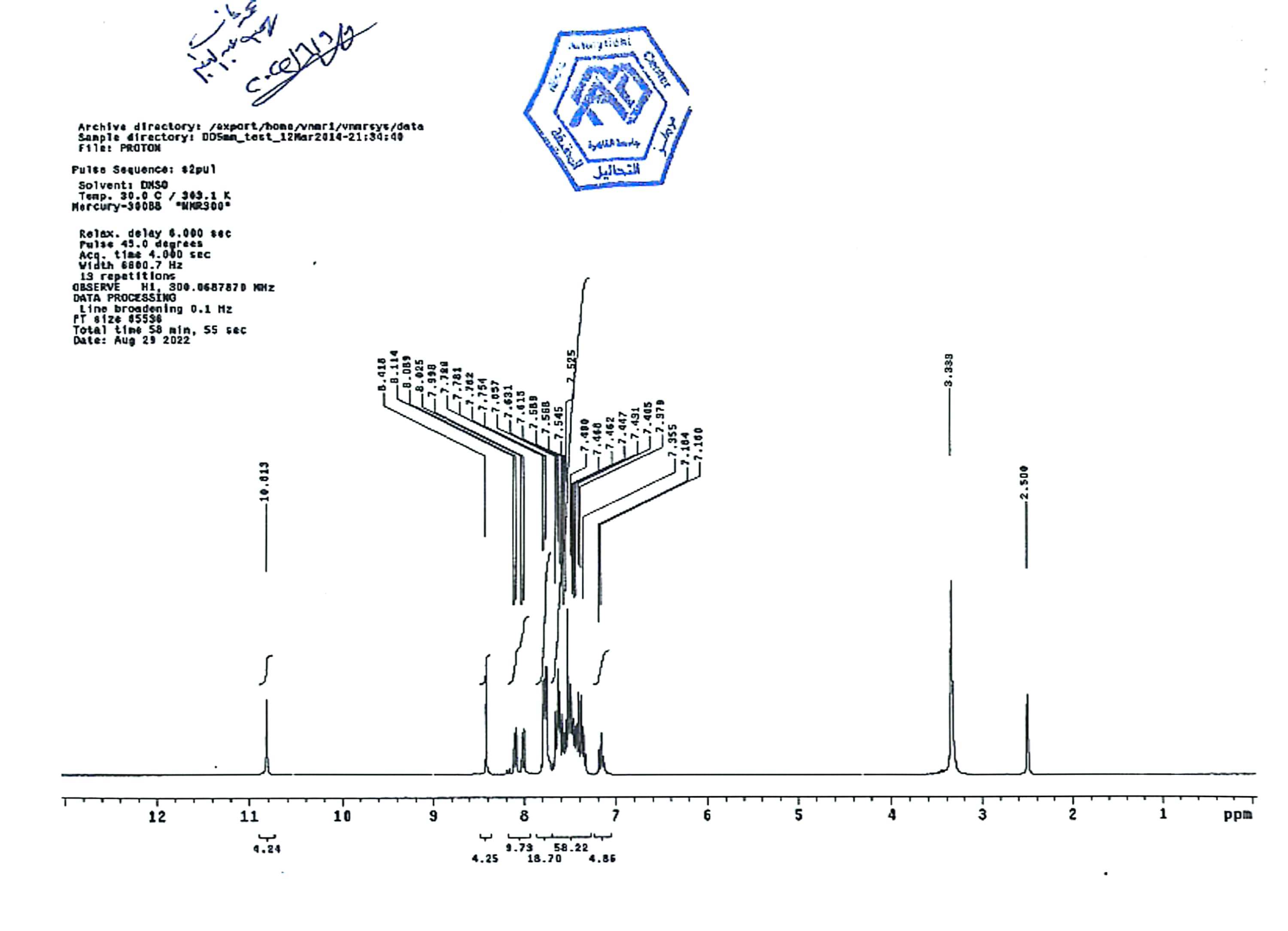


**Figure S59**. **^13^C NMR spectrum of compound 6a**


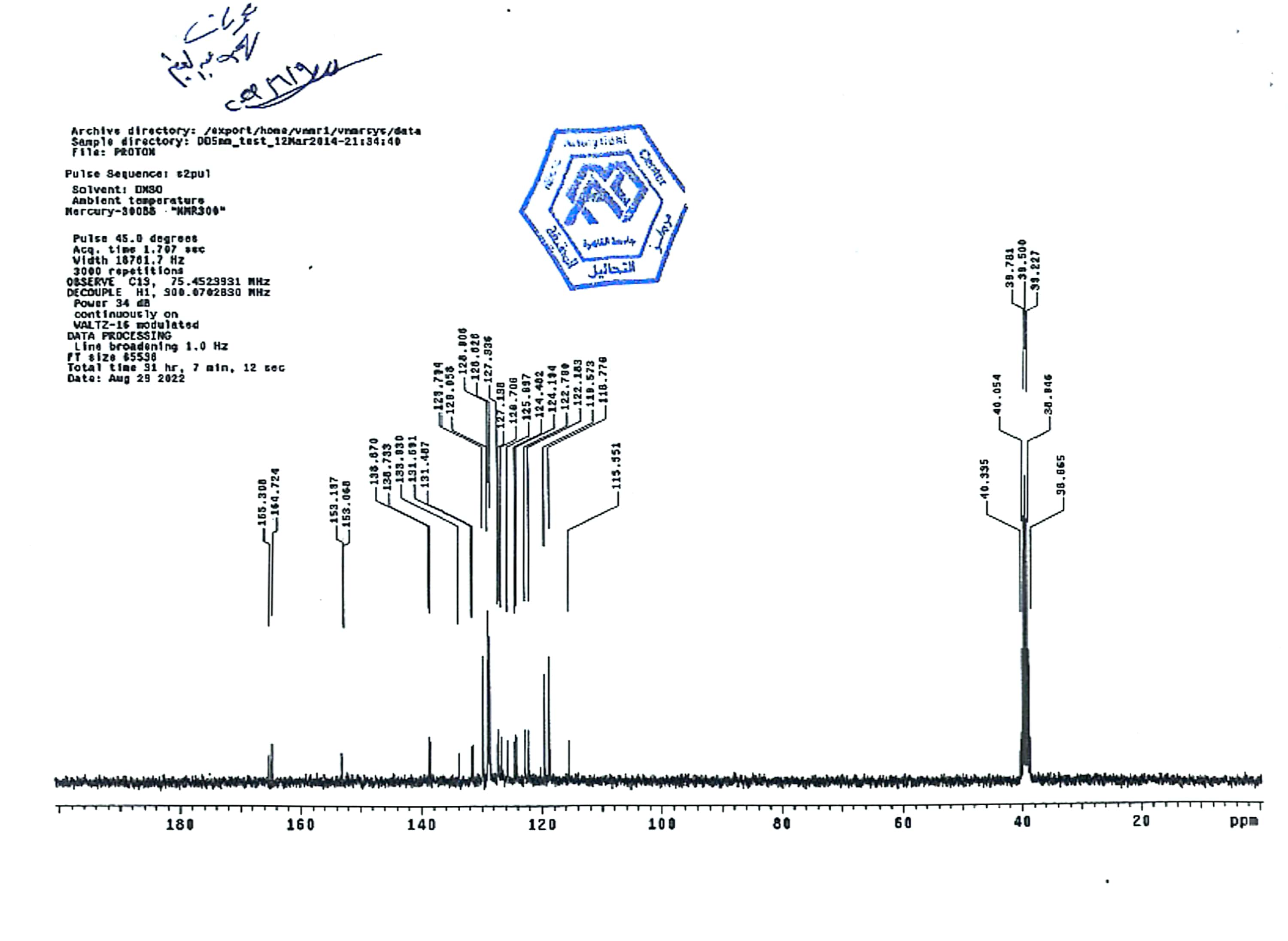


**Figure S60**. **IR spectrum of compound 6a**


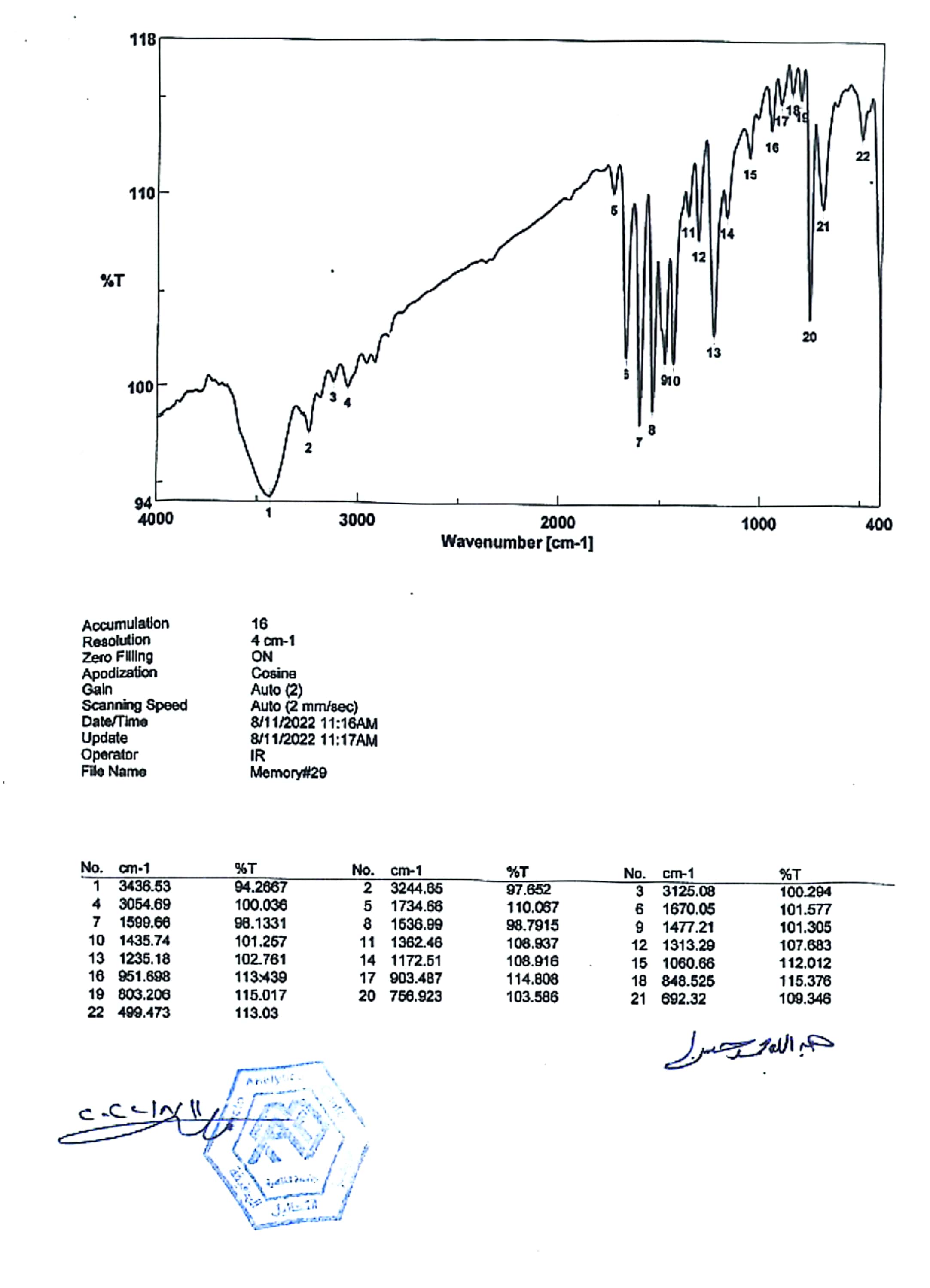


**Figure S61**. **Mass spectrum of compound 6a**


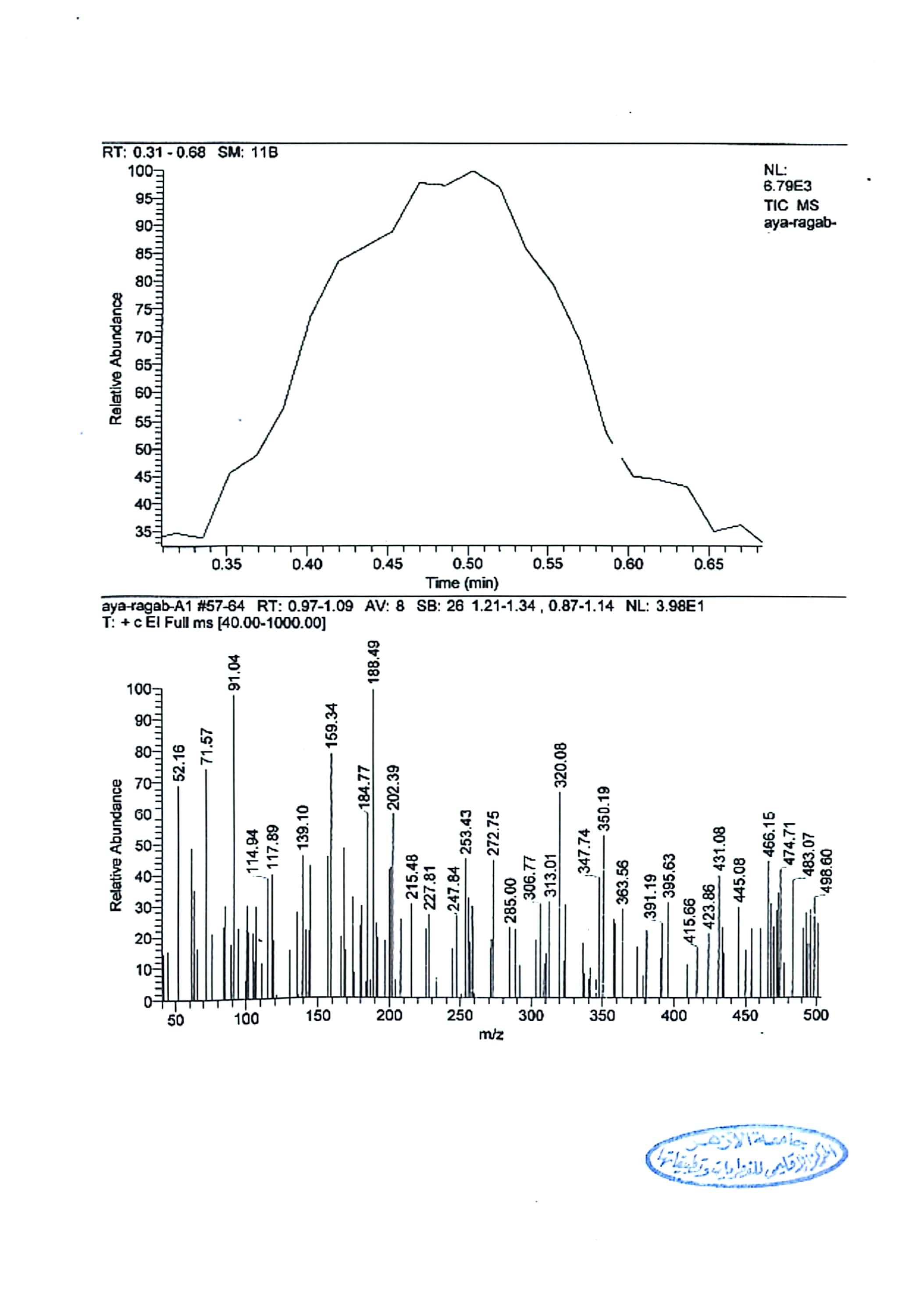


**Figure S62**. **^1^H NMR spectrum of compound 6b**


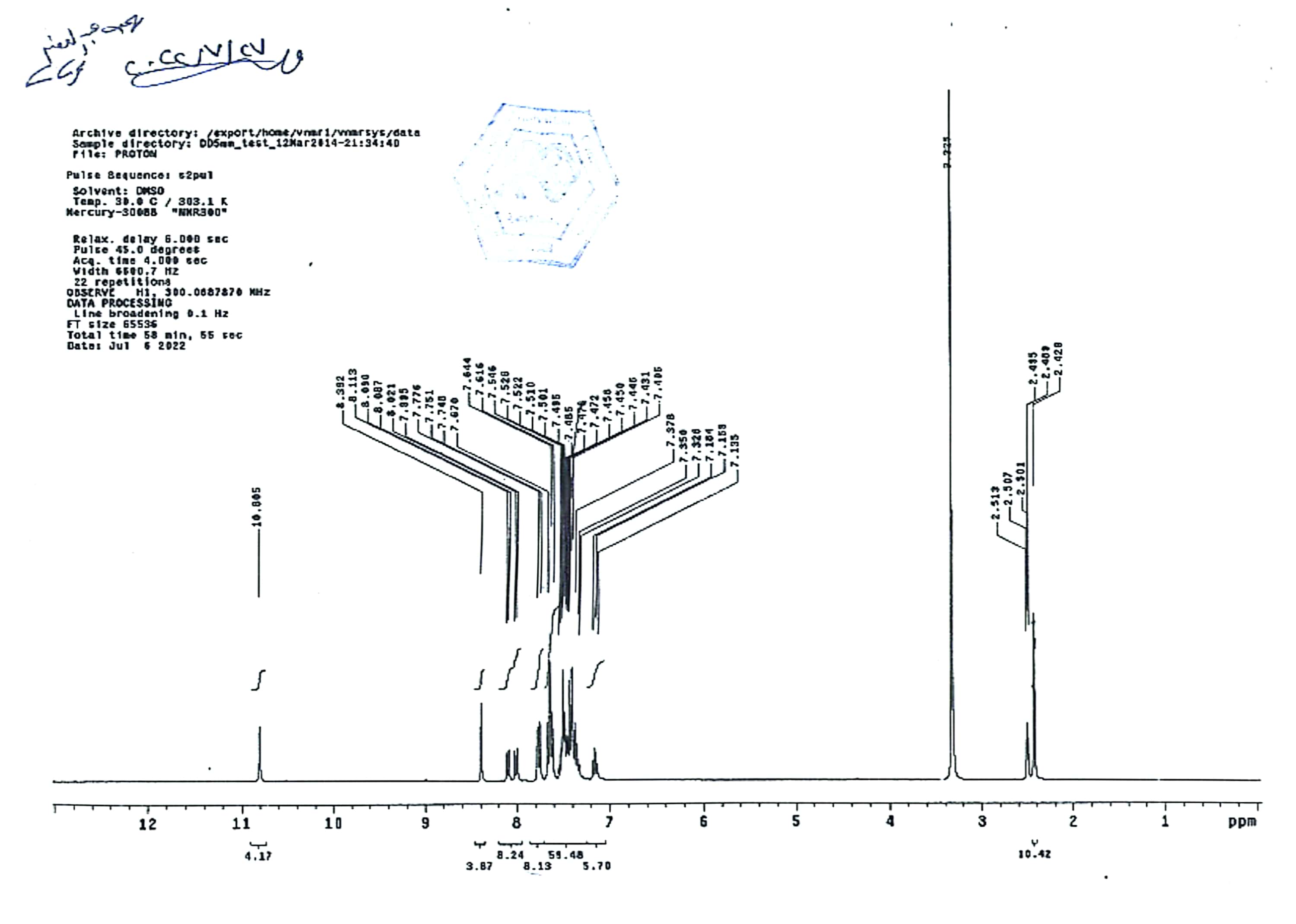


**Figure S63**. **^13^C NMR spectrum of compound 6b**


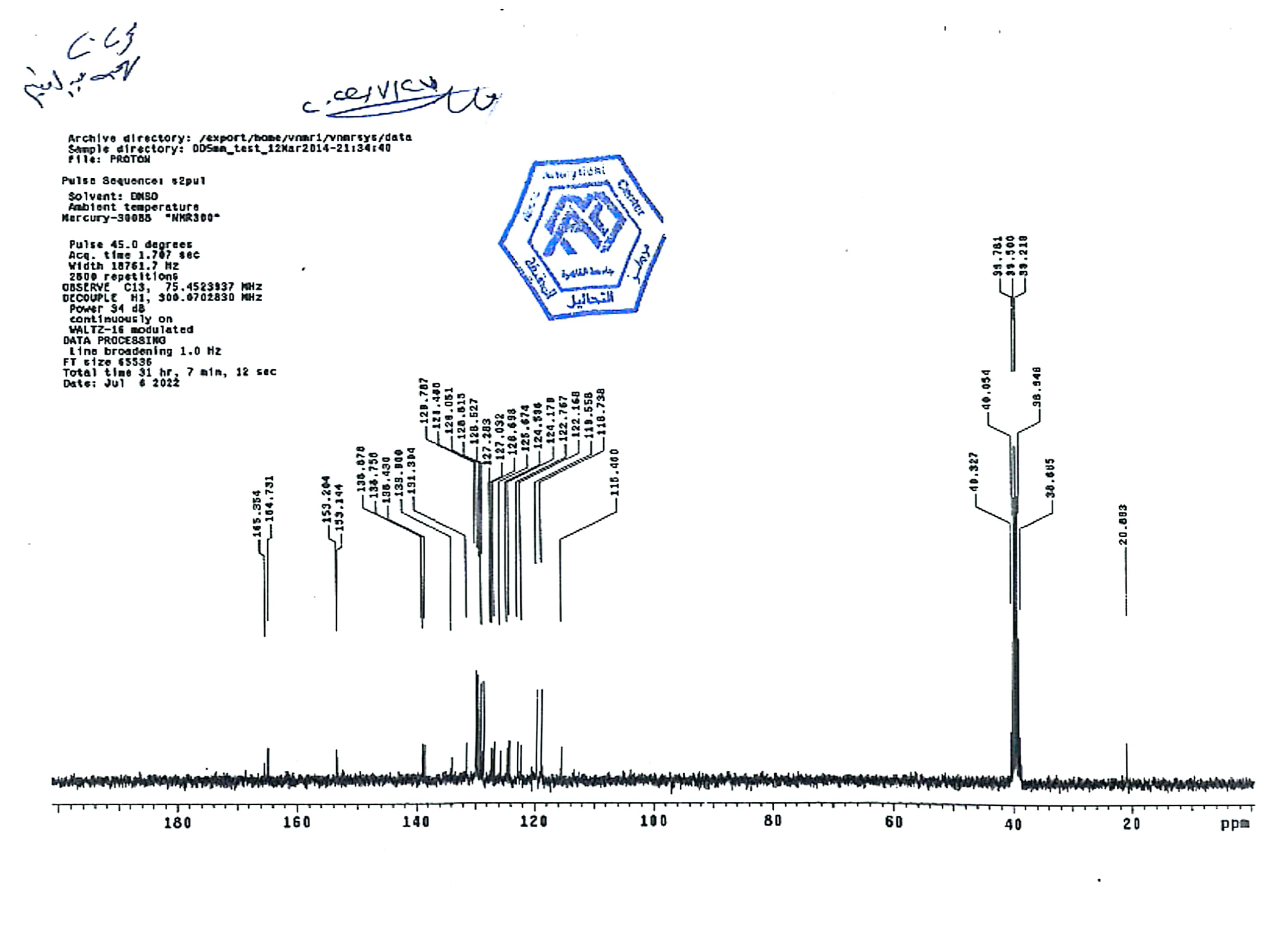


**Figure S64**. **IR spectrum of compound 6b**


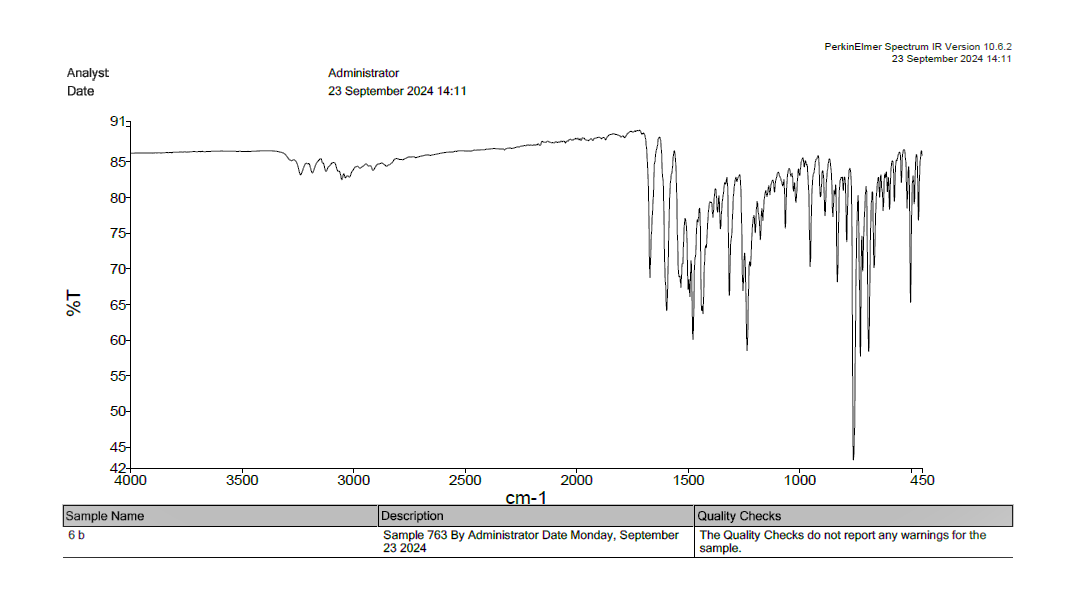


**Figure S65**. **Mass spectrum of compound 6b**


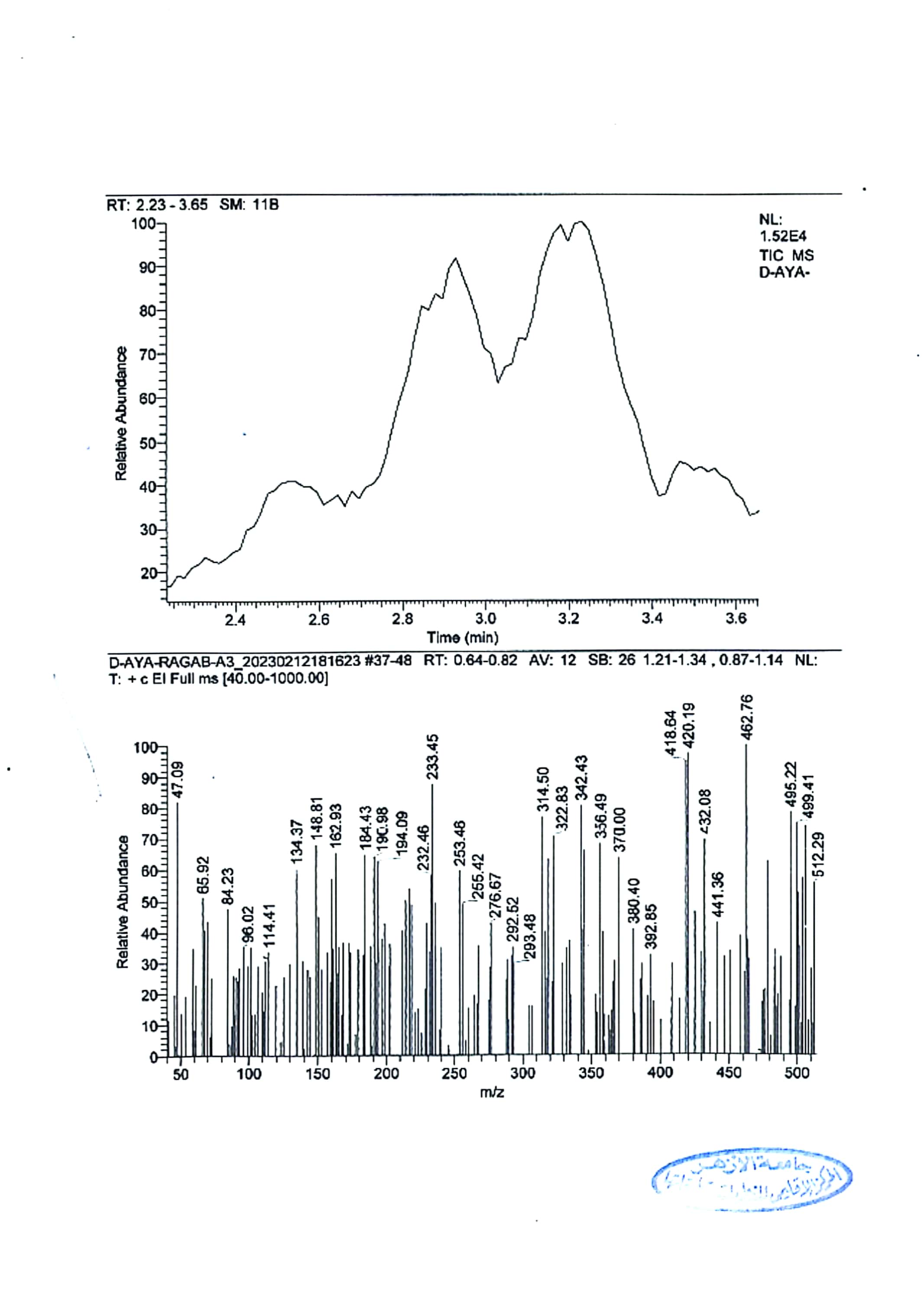


**Figure S66**. **1H NMR spectrum of compound 6c**


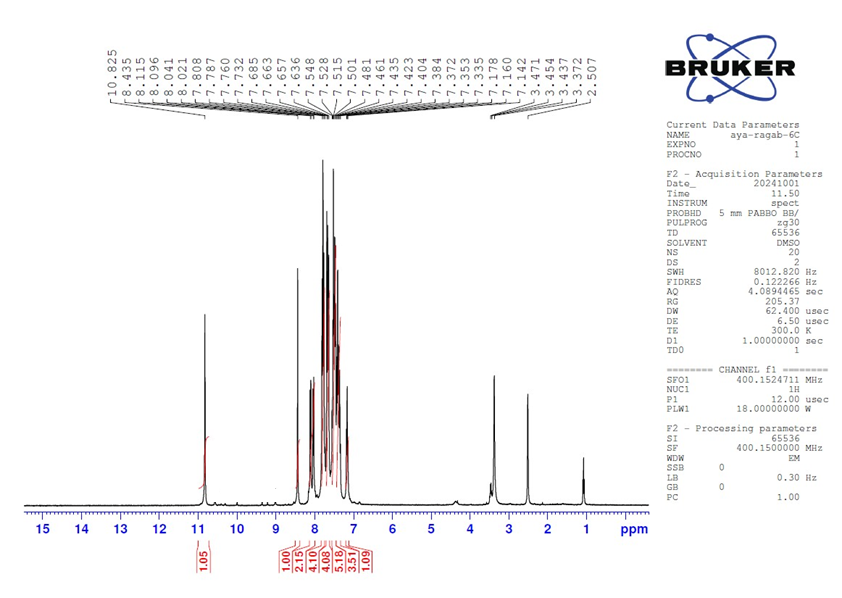


**Figure S67**. **^13^C NMR spectrum of compound 6c**


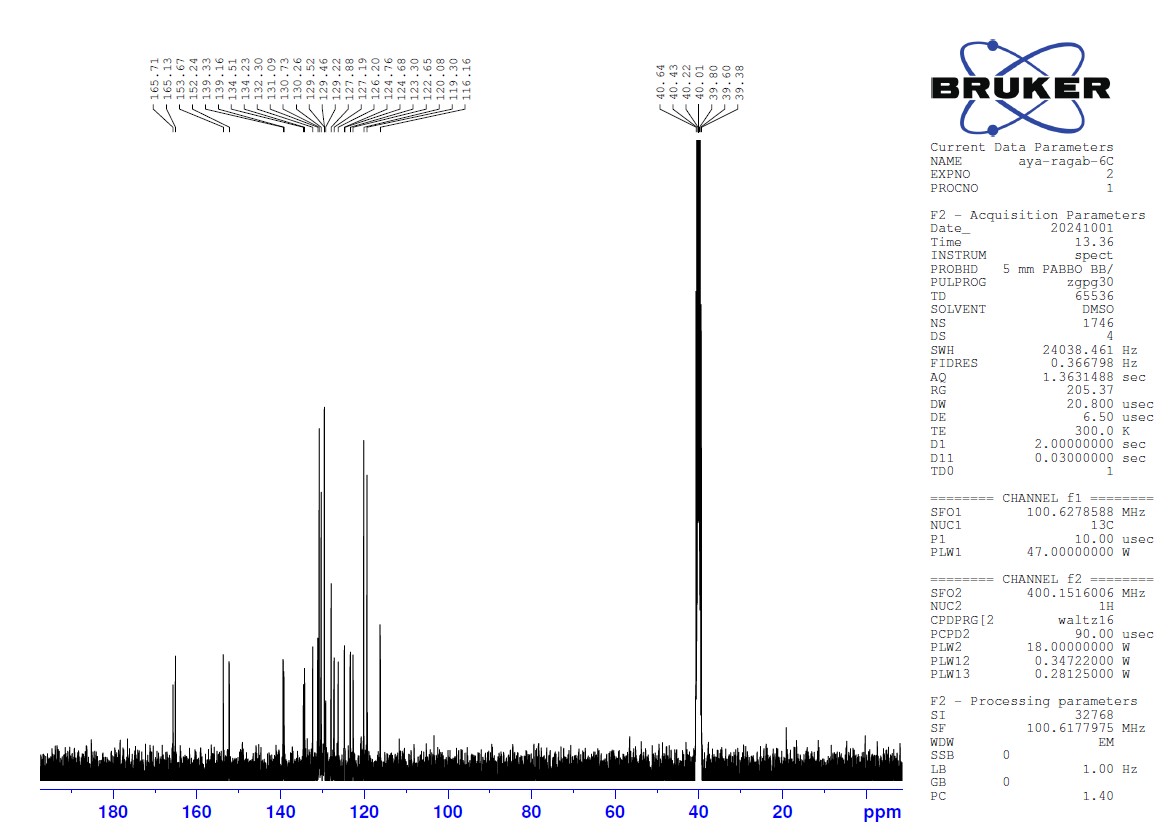


**Figure S68**. **IR spectrum of compound 6c**


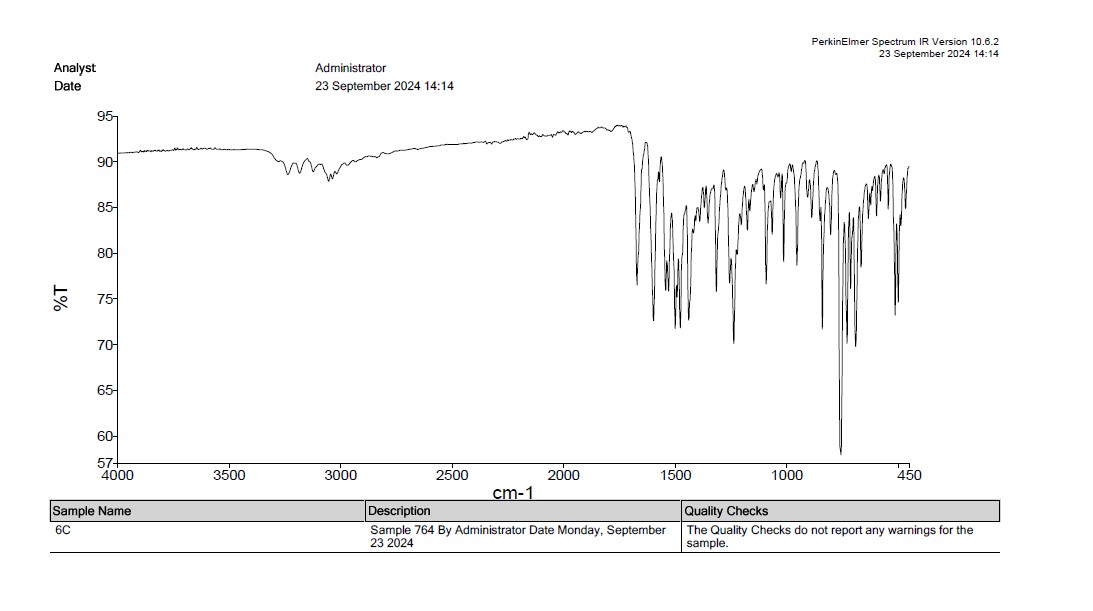


**Figure S69**. **1H NMR spectrum of compound 6d**

**
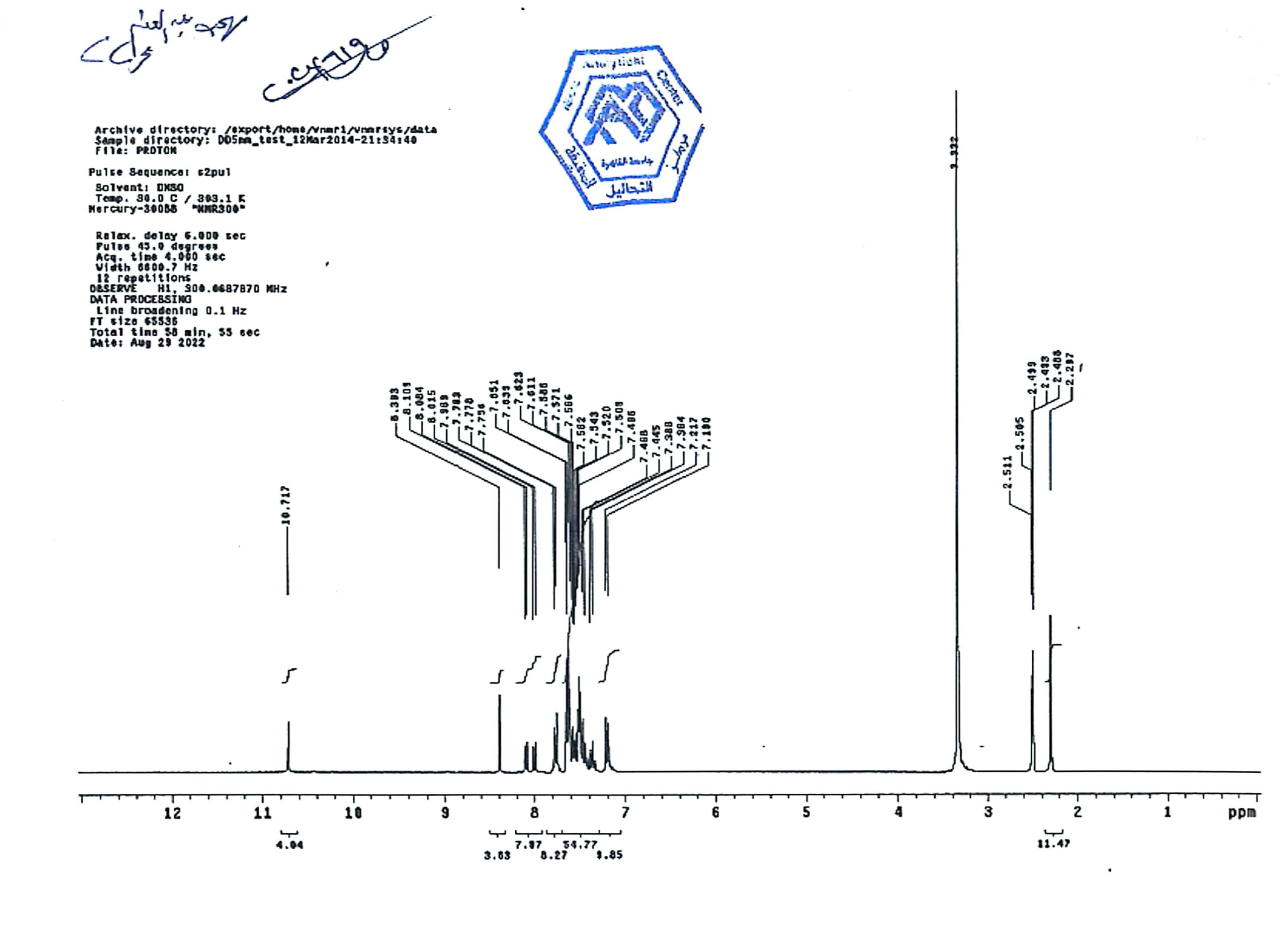
**

**Figure S70**. **^13^C NMR spectrum of compound 6d**

**
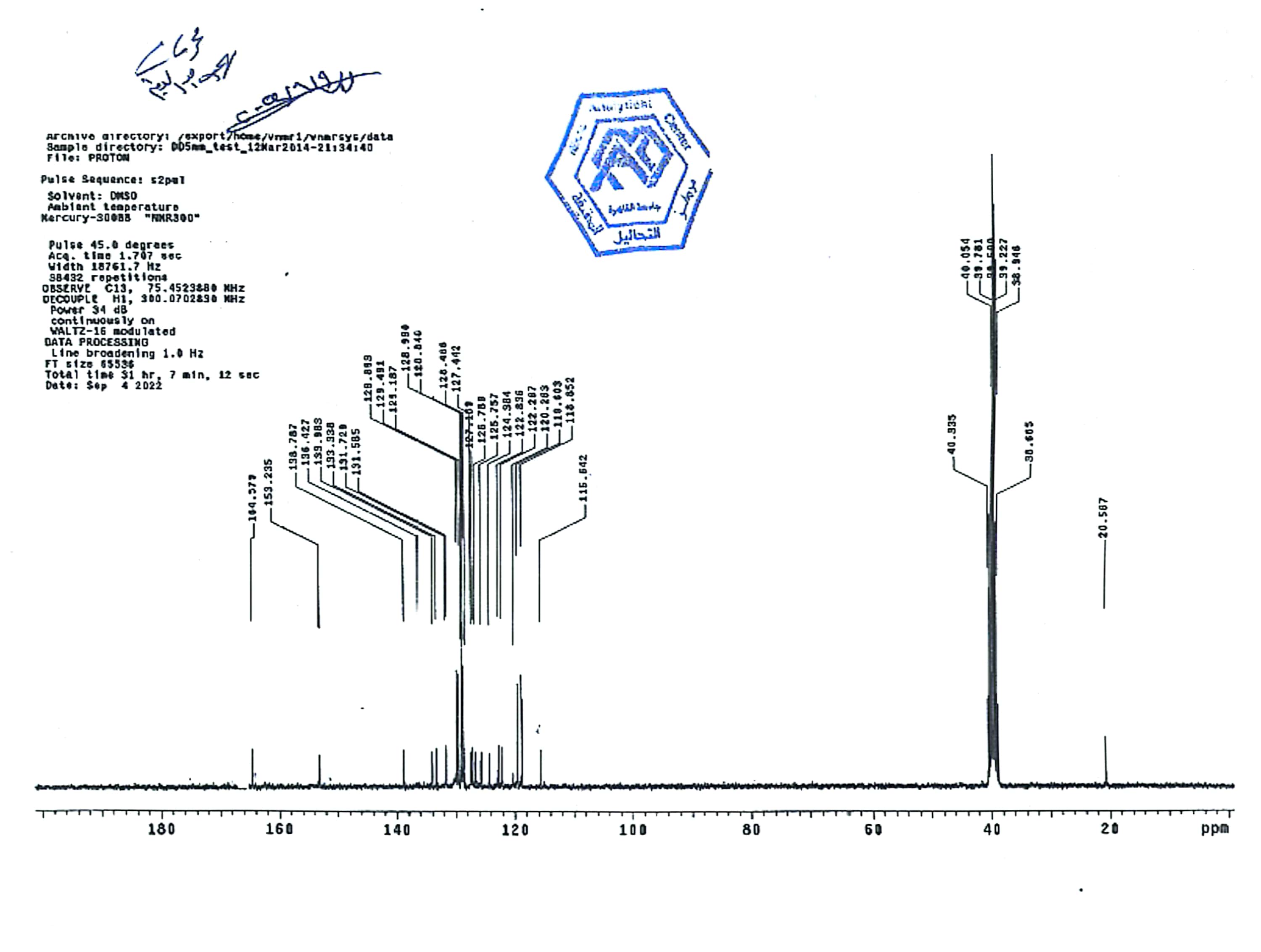
**

**Figure S71**. **IR spectrum of compound 6d**

**
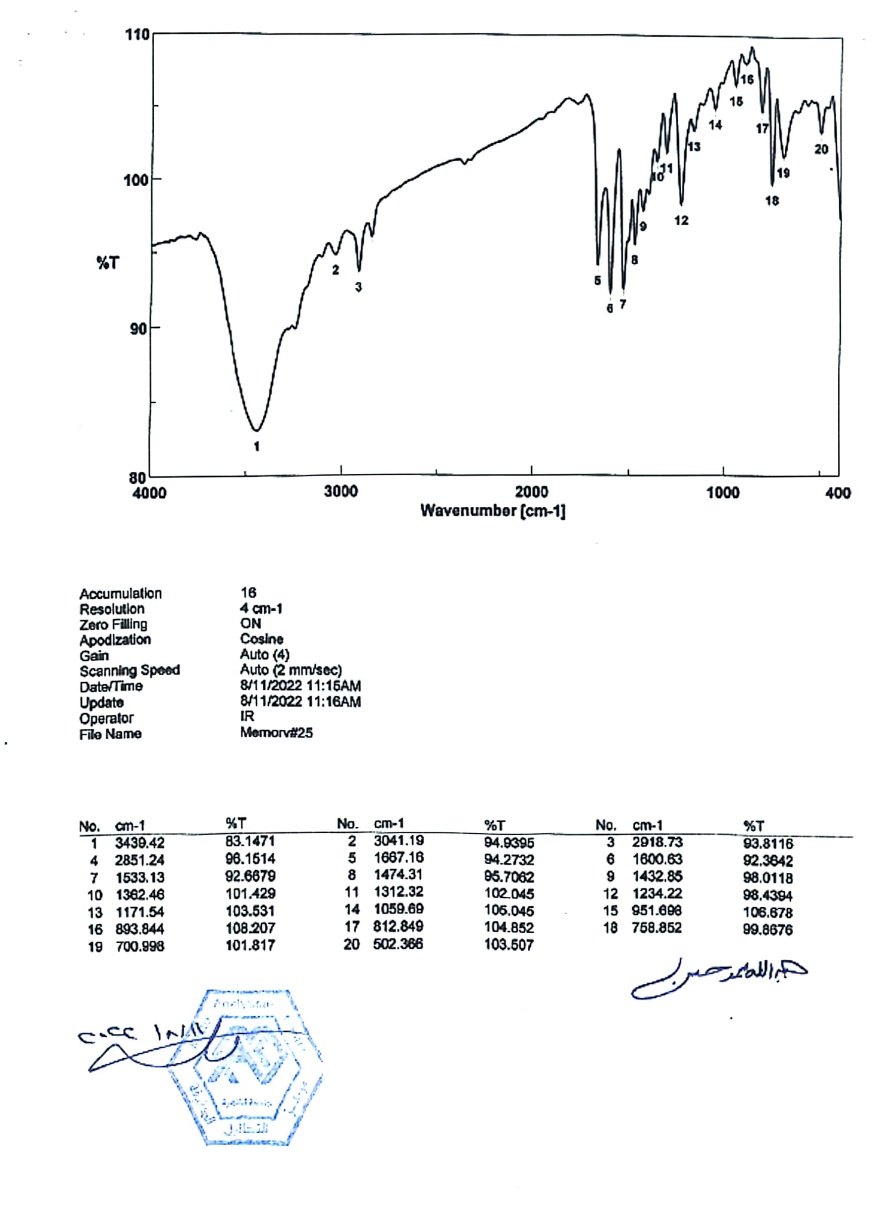
**

**Figure S72**. **^1^H NMR spectrum of compound 6e**

**
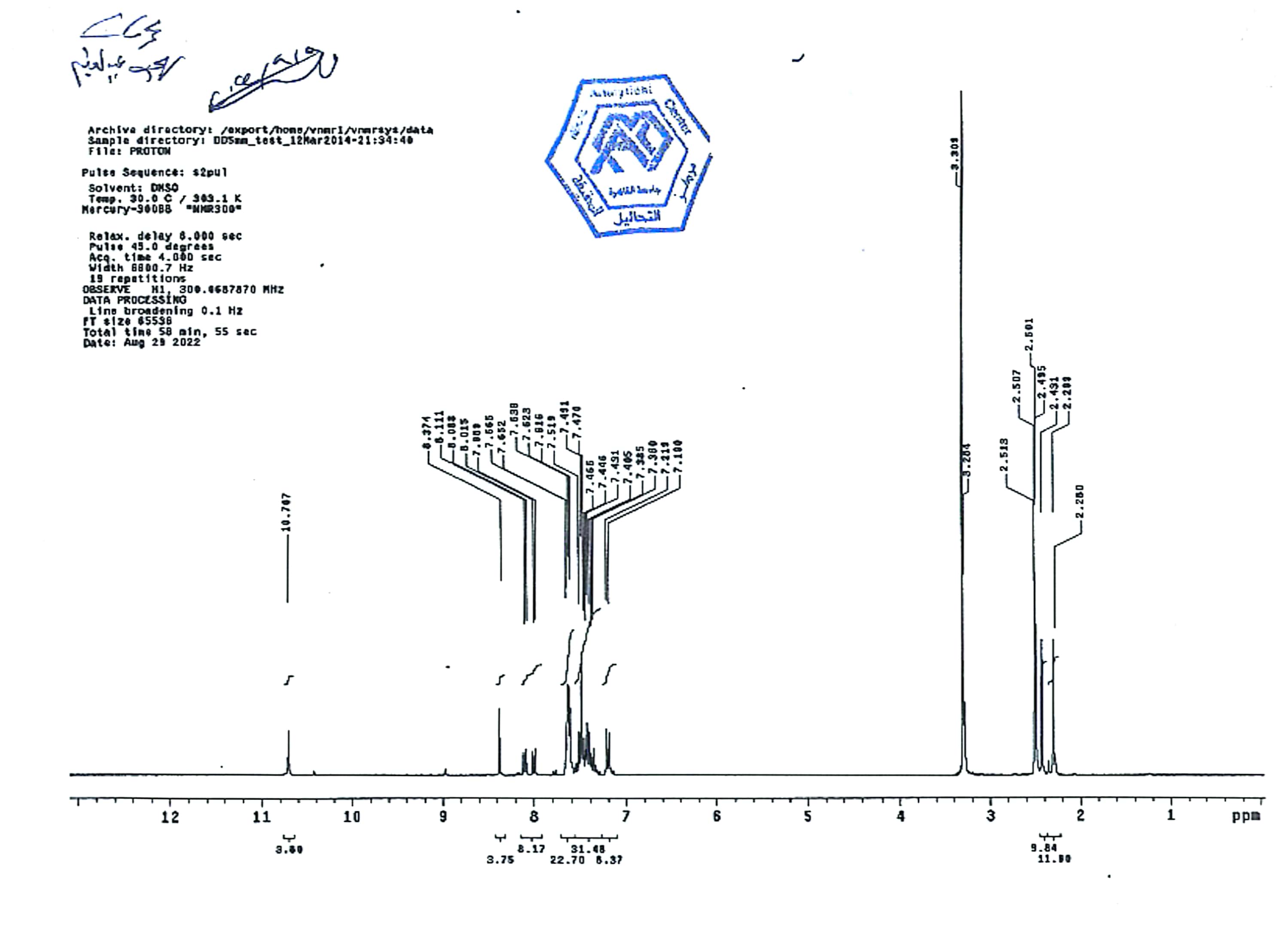
**

**Figure S73**. **^13^C NMR spectrum of compound 6e**

**
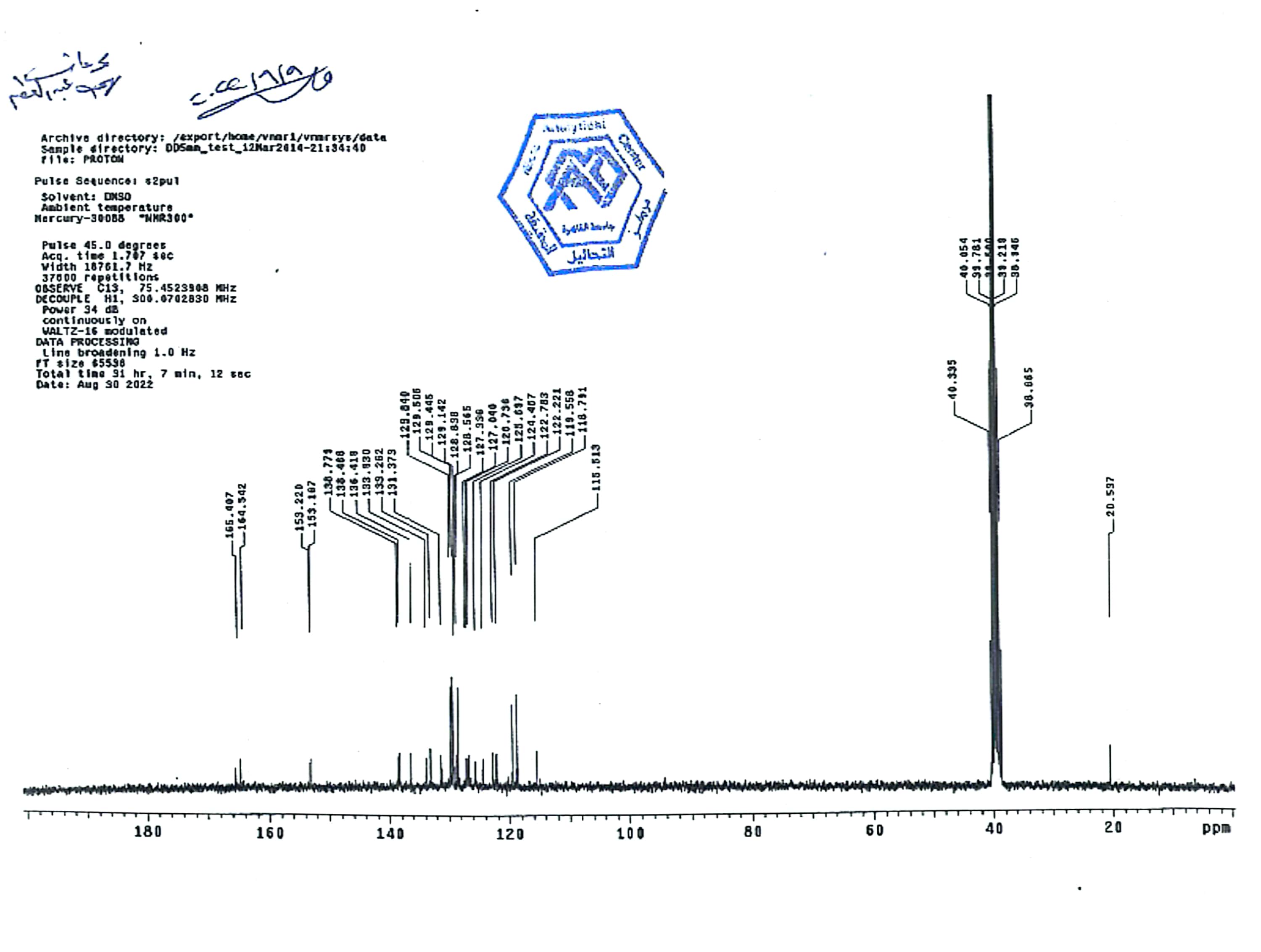
**

**Figure S74**. **IR spectrum of compound 6e**

**
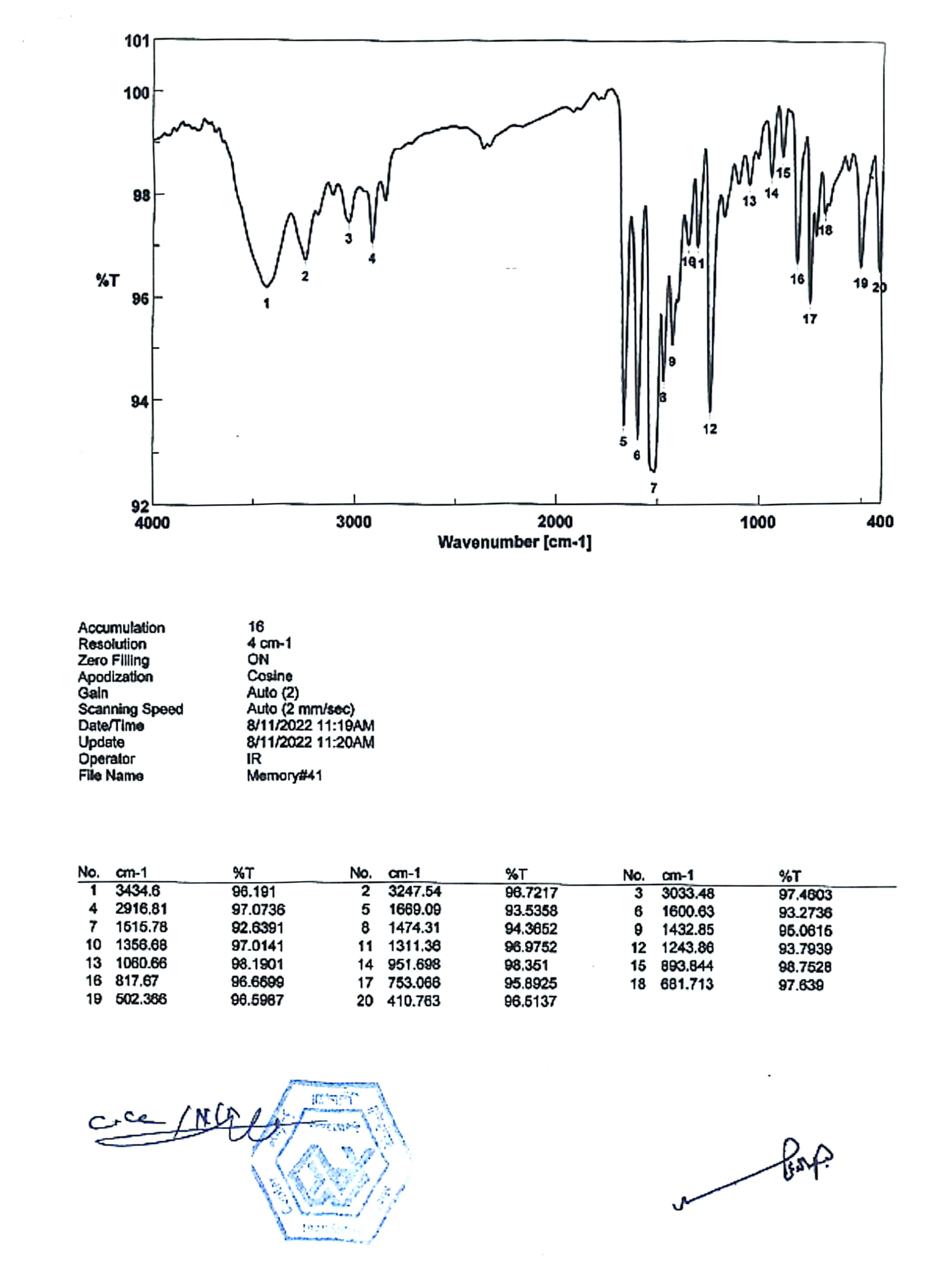
**

**Figure S75**. **Mass spectrum of compound 6e**

**
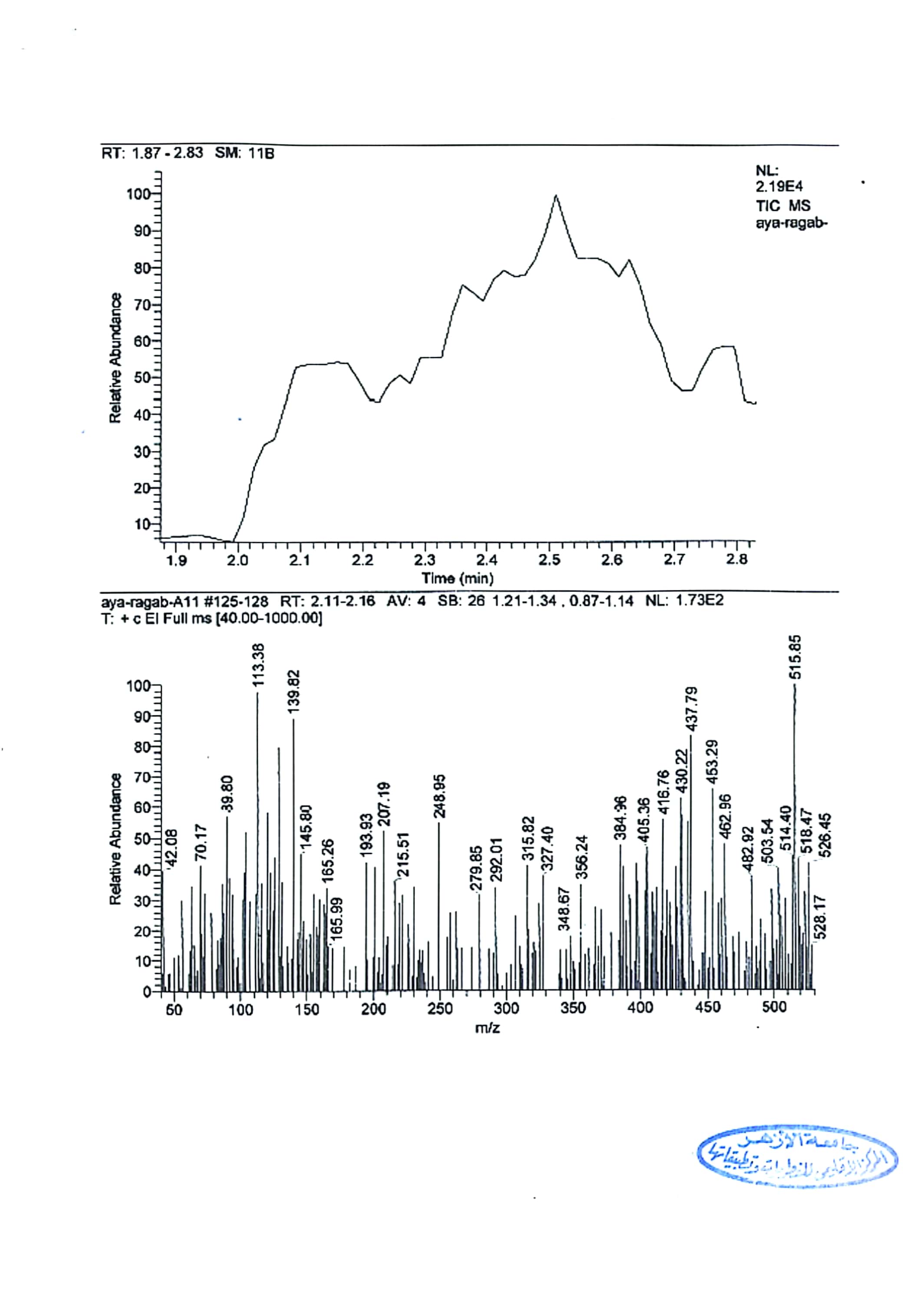
**

**Figure S76**. **^1^H NMR spectrum of compound 6f**

**
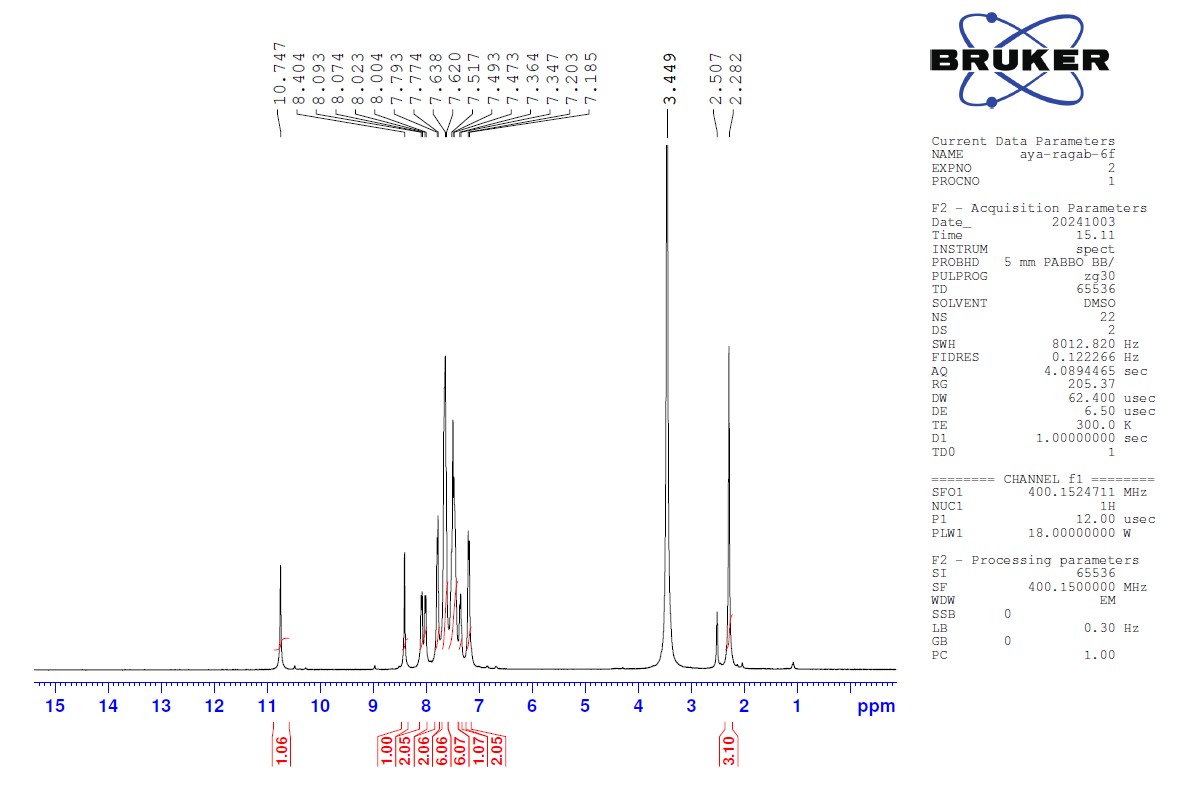
**

**Figure S77**. **^13^C NMR spectrum of compound 6f**

**
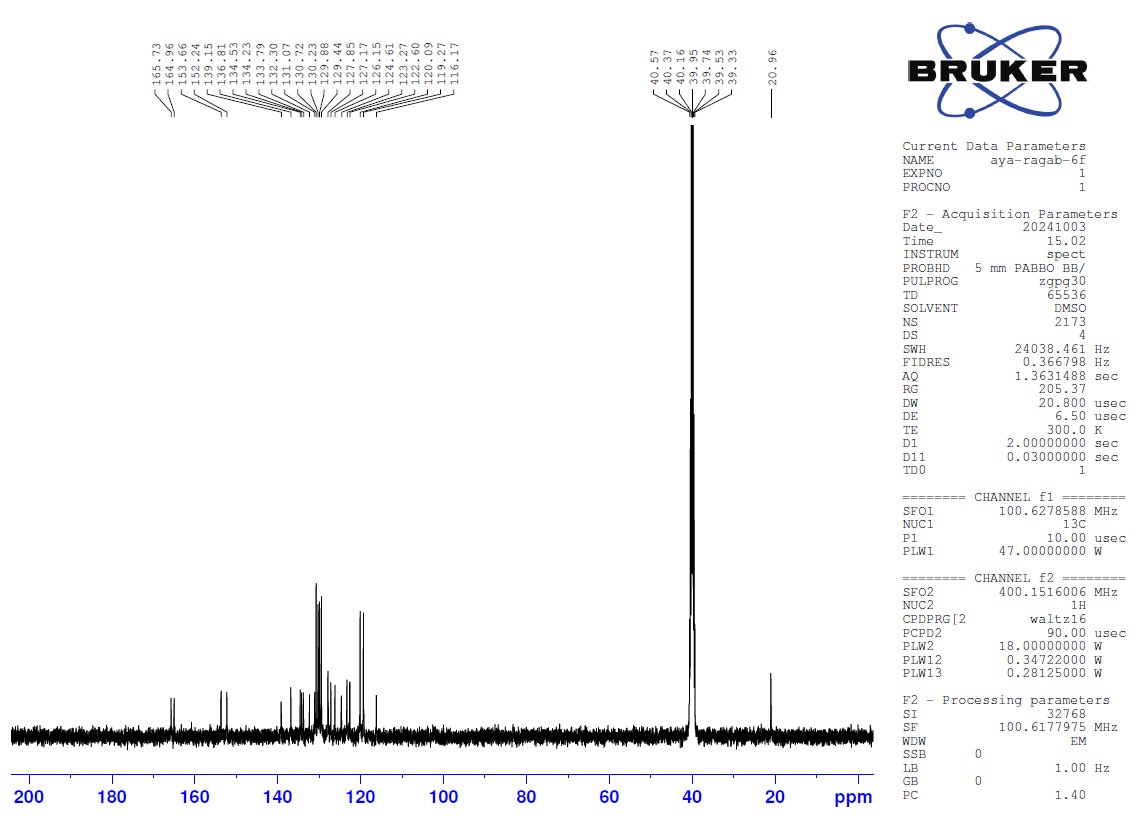
**

**Figure S78**. **IR spectrum of compound 6f**

**
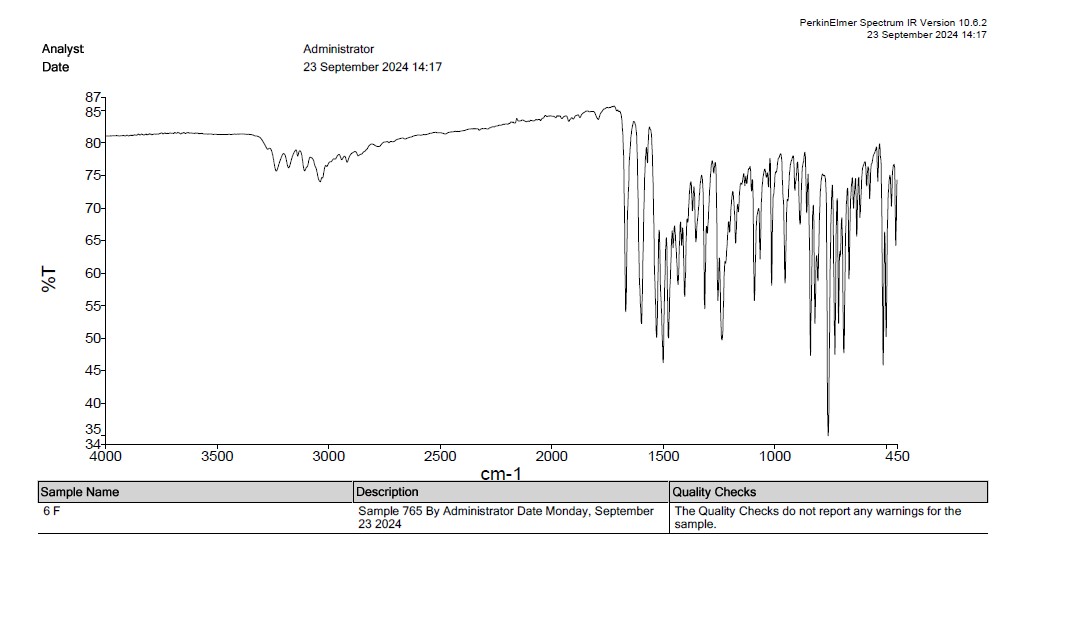
**

**Figure S79**. **^1^H NMR spectrum of compound 6g**

**
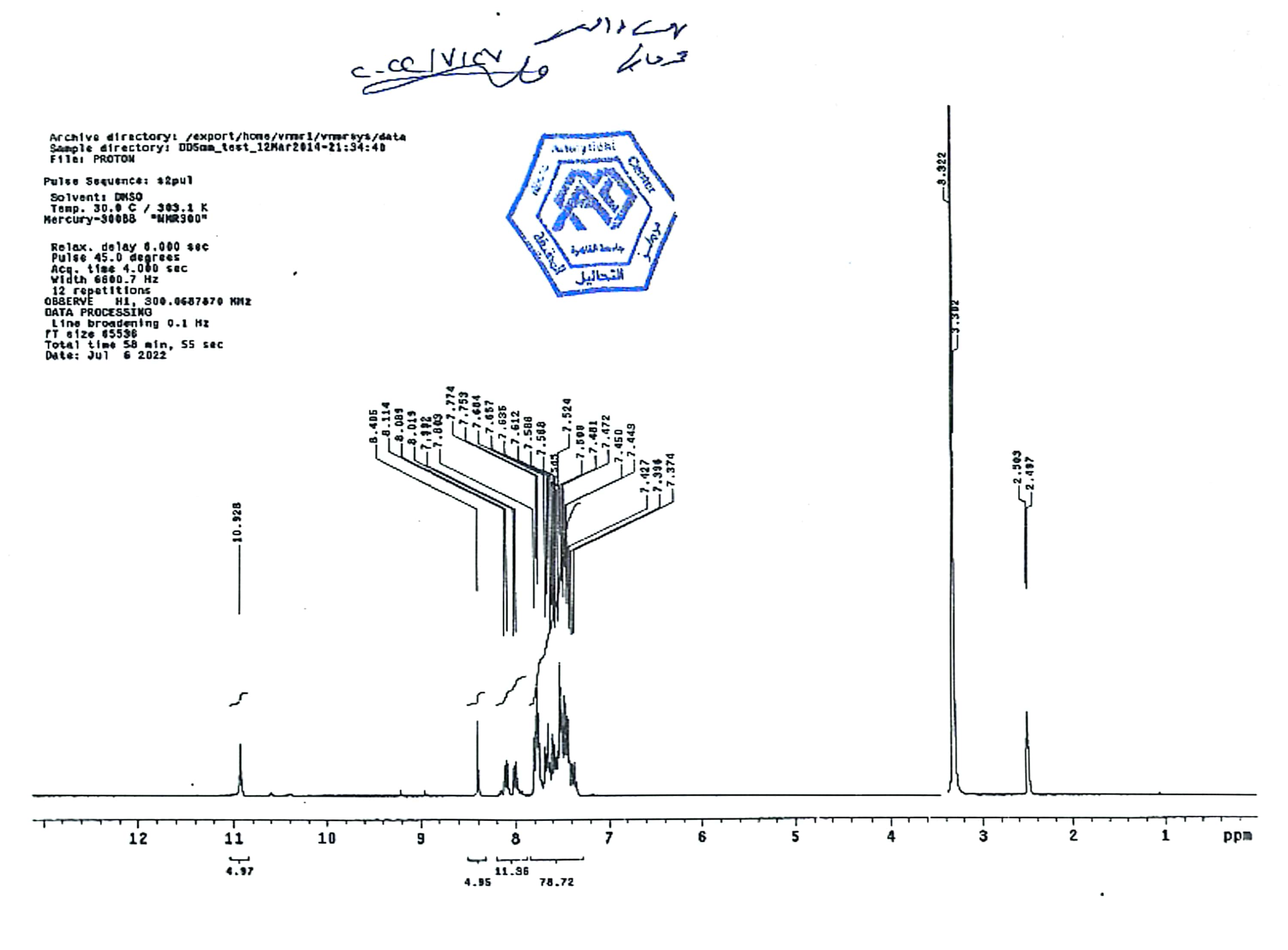
**

**Figure S80**. **^13^C NMR spectrum of compound 6g**

**
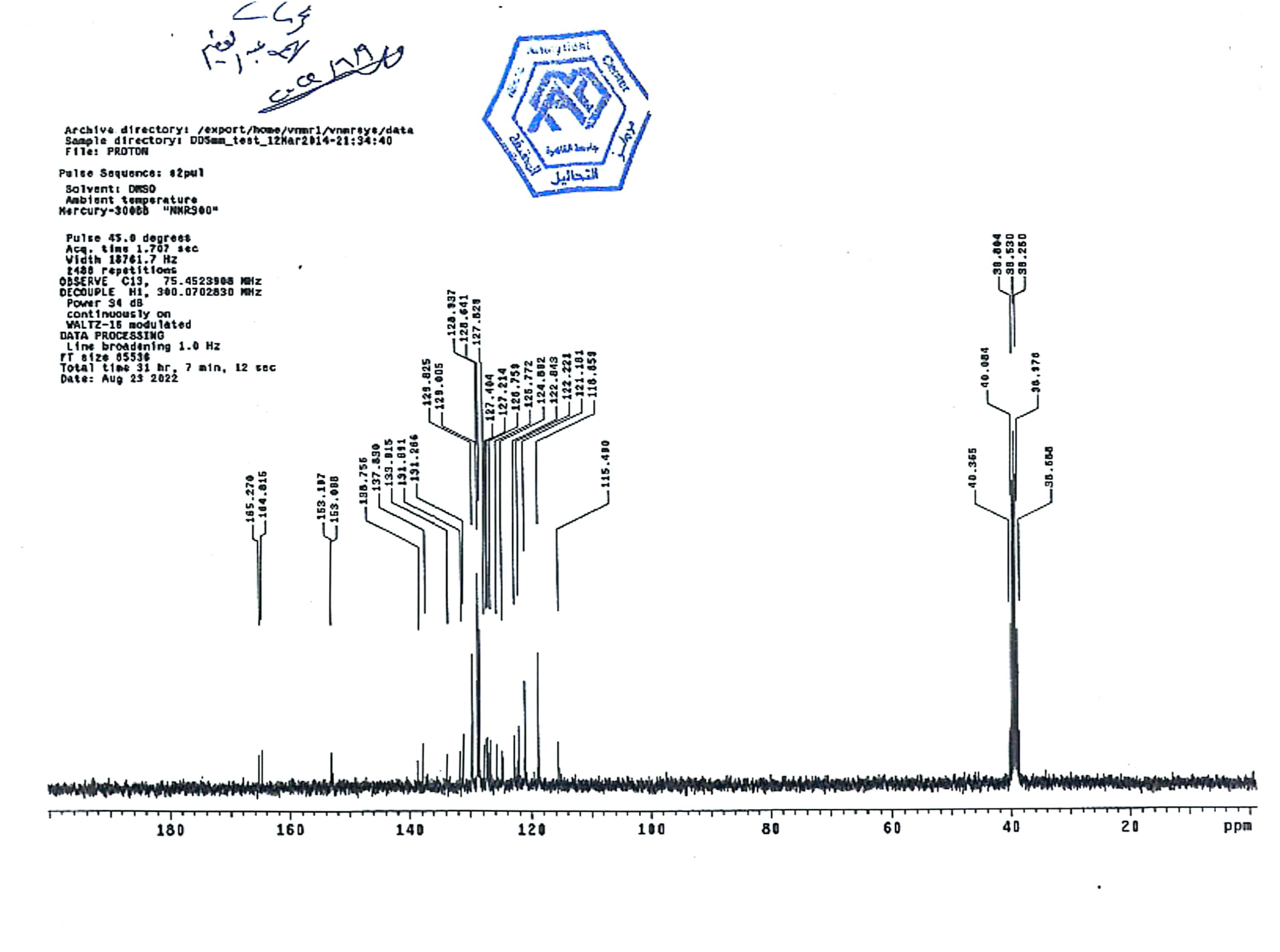
**

**Figure S81**. **IR spectrum of compound 6g**

**
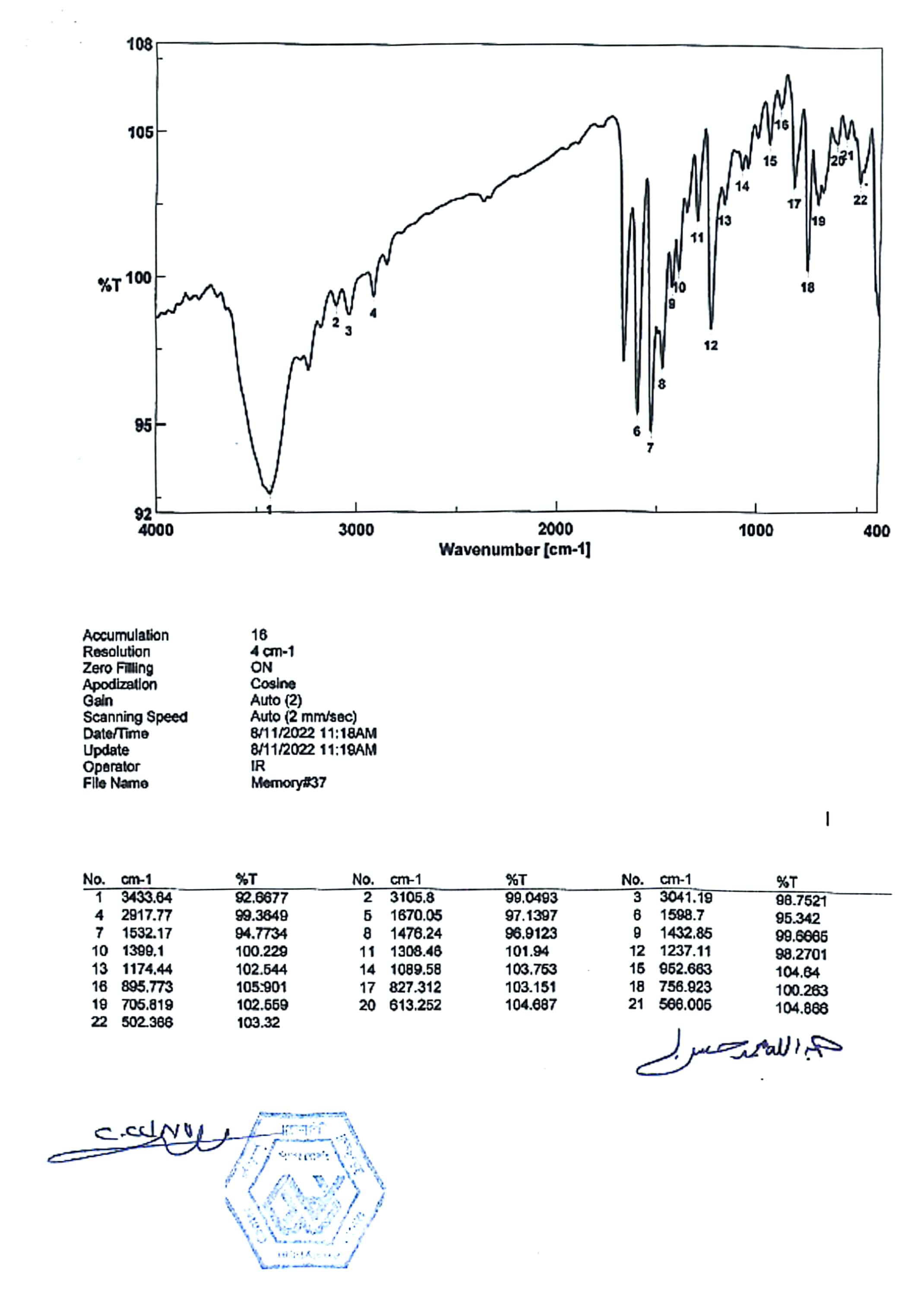
**

**Figure S82**. **Mass spectrum of compound 6g**

**
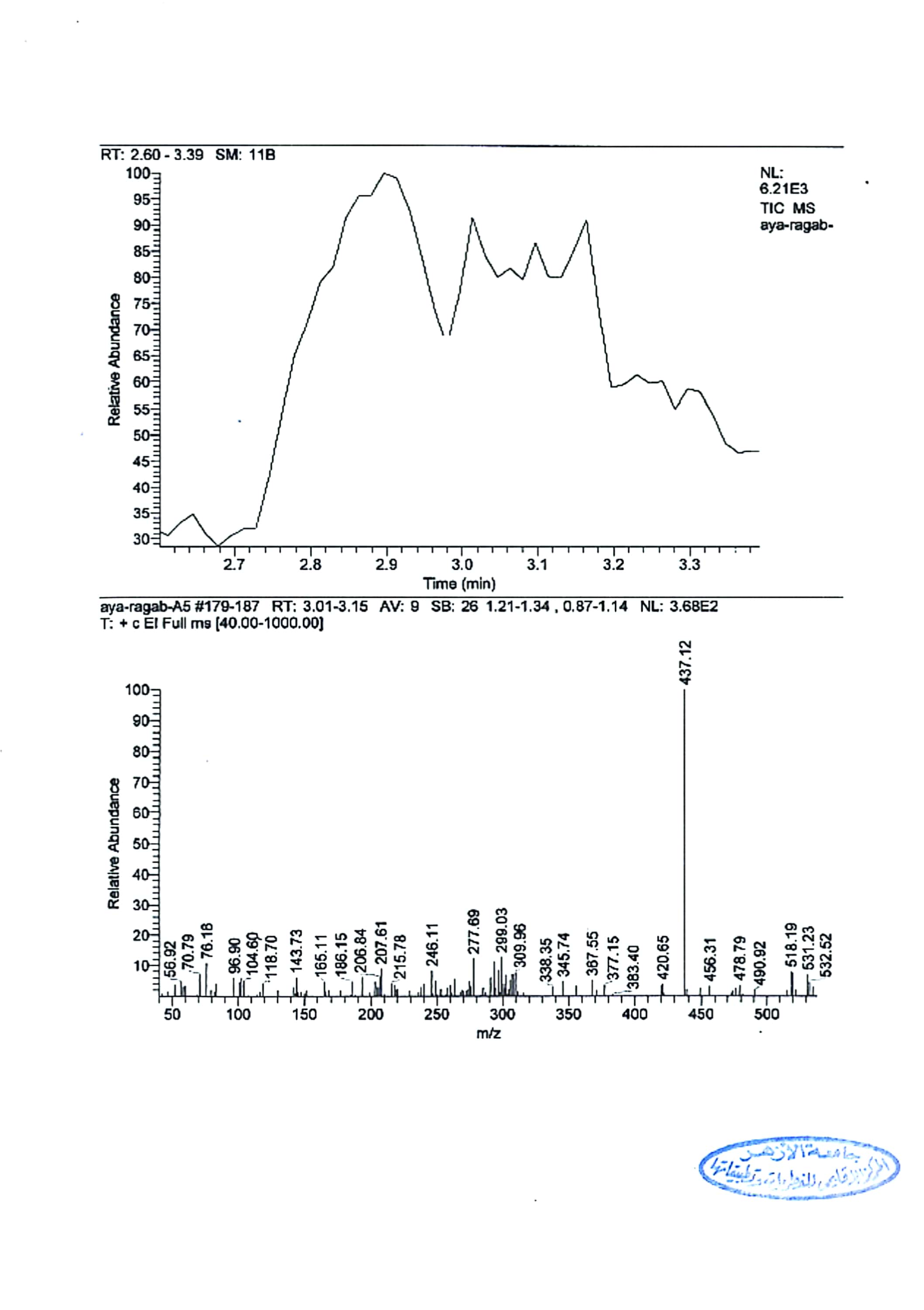
**

**Figure S83**. **^1^H NMR spectrum of compound 6h**

**
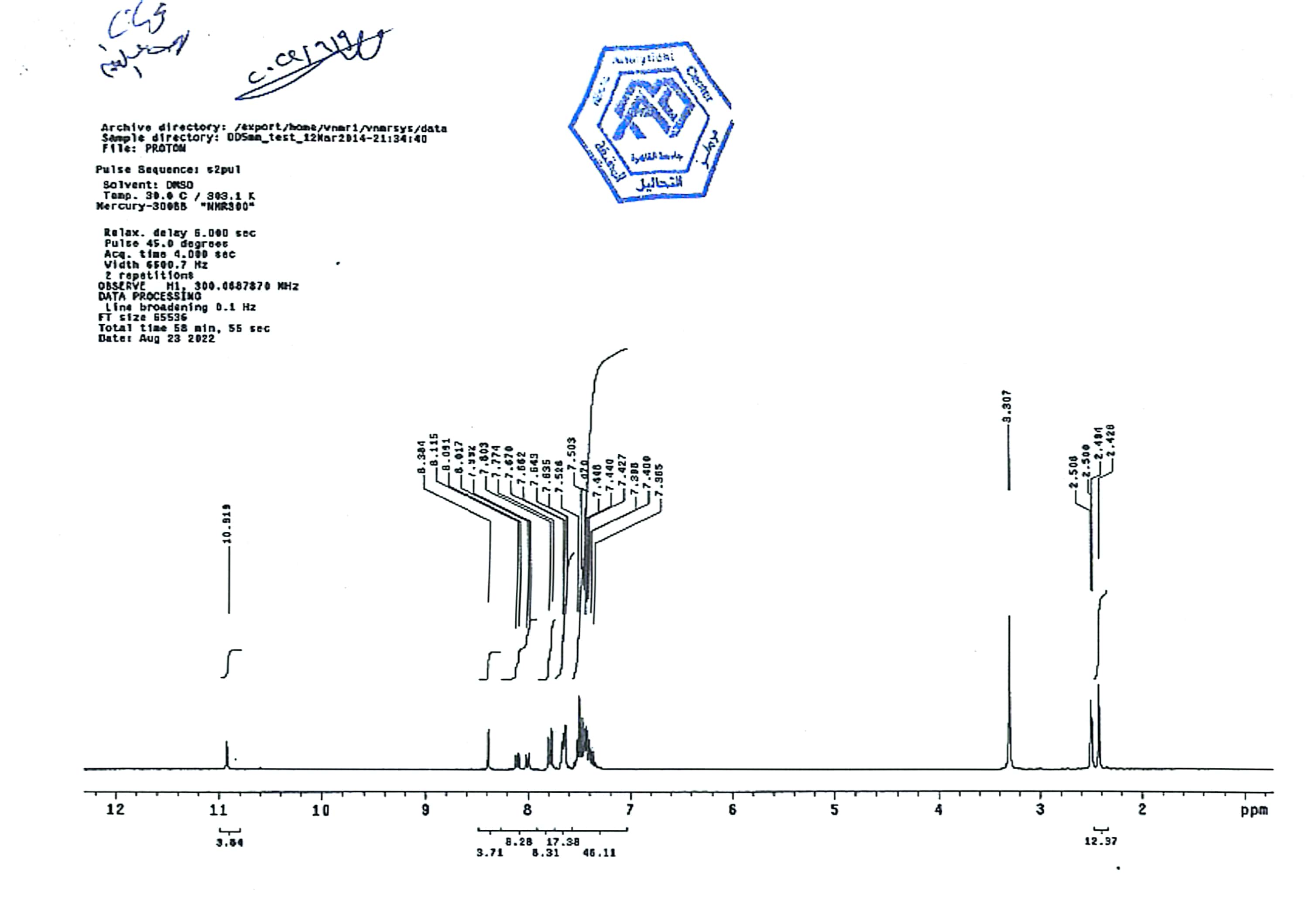
**

**Figure S84**. **^13^C NMR spectrum of compound 6h**

**
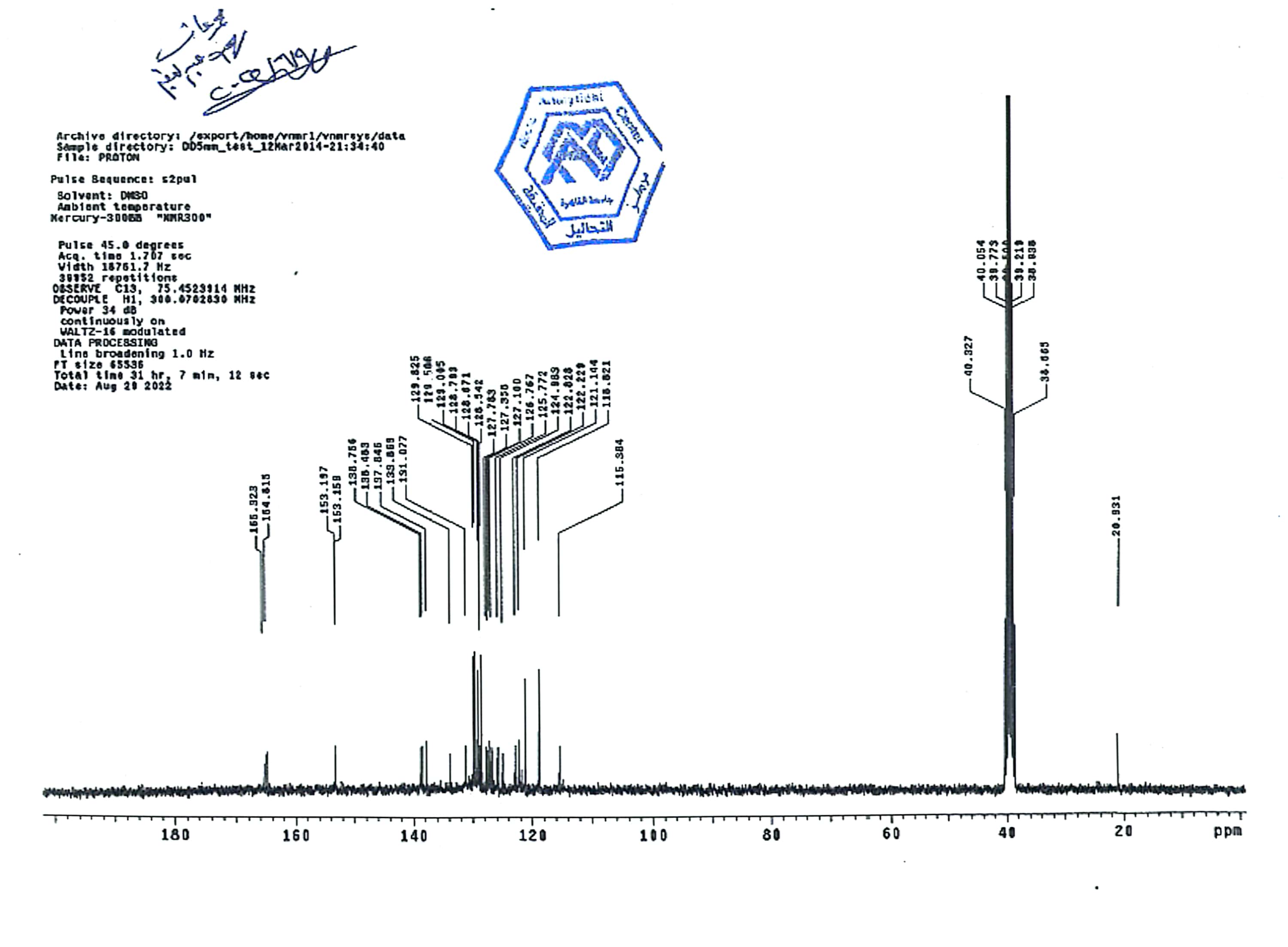
**

**Figure S85**. **^1^H NMR spectrum of compound 6i**

**
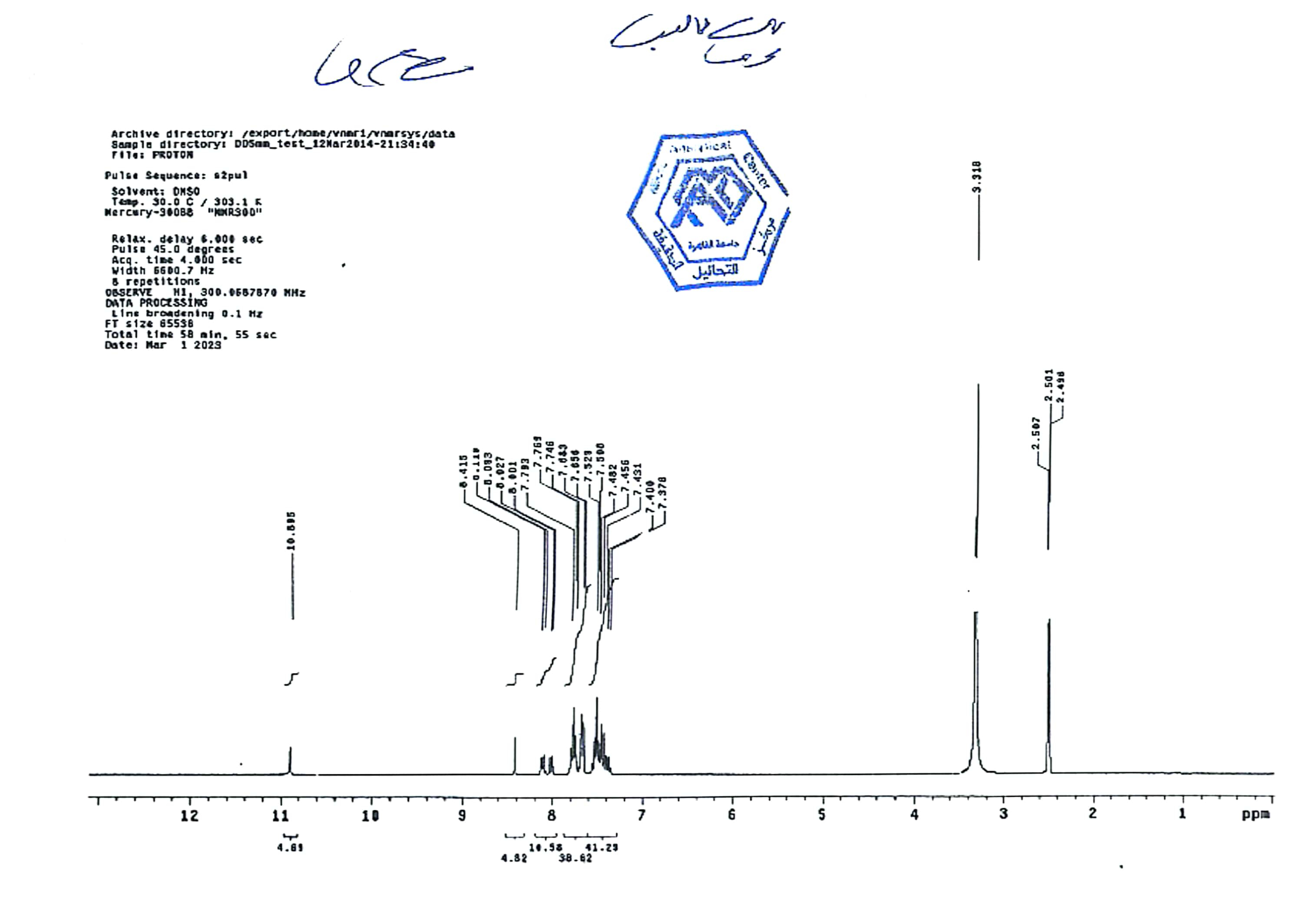
**

**Figure S86**. **^13^C NMR spectrum of compound 6i**

**
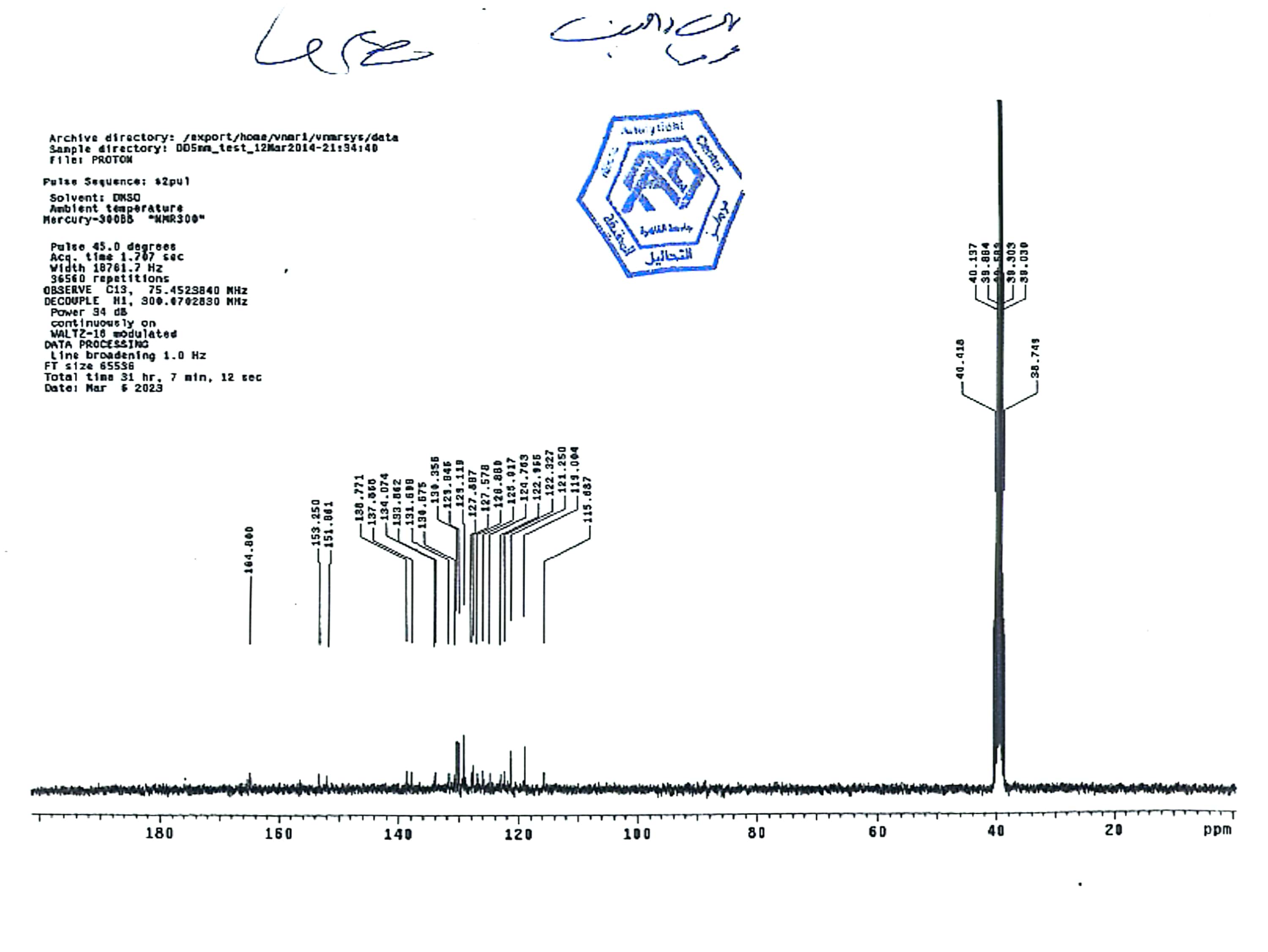
**

**Figure S87**. **IR spectrum of compound 6i**

**
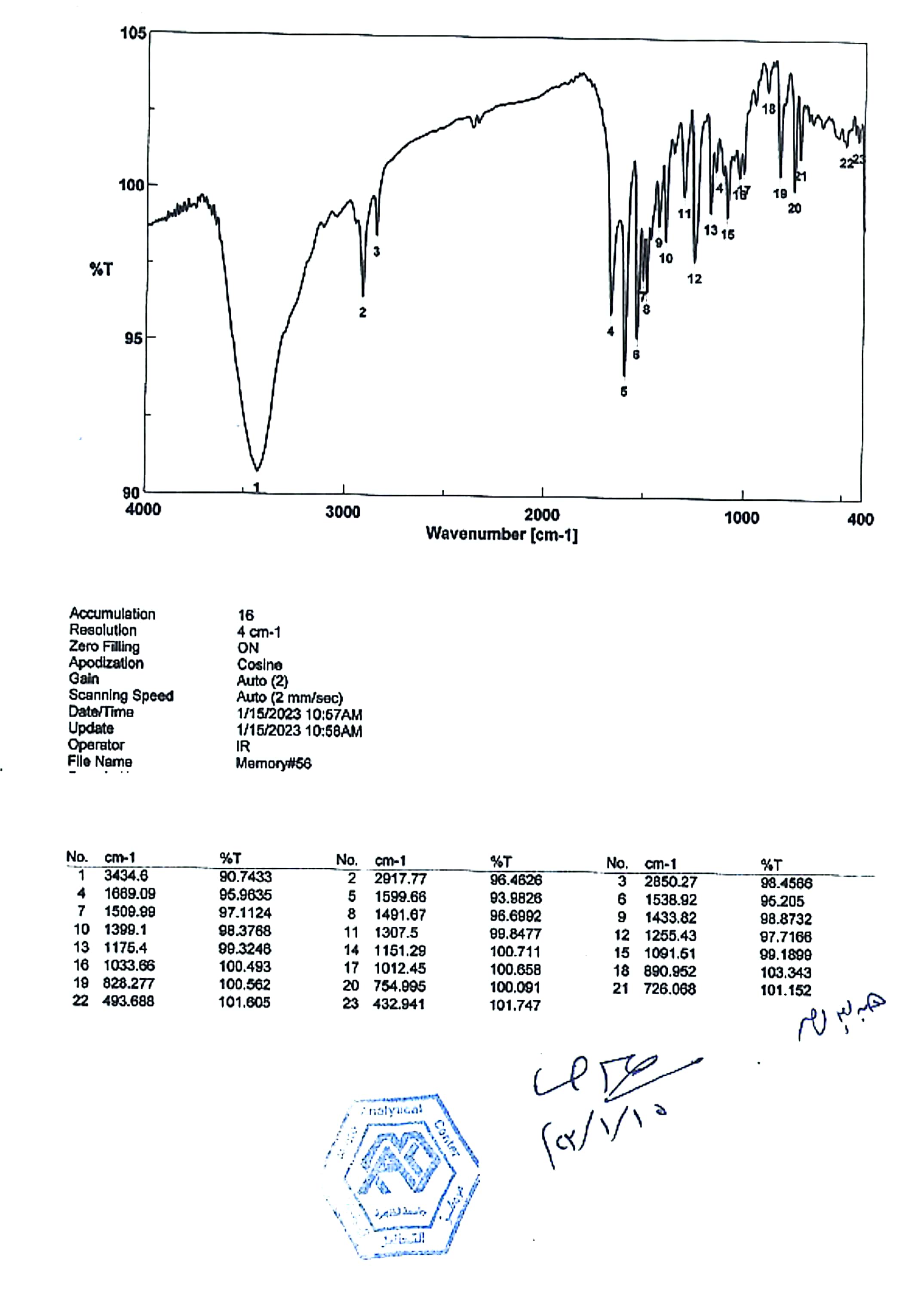
**

**Figure S88**. **Mass spectrum of compound 6i**

**
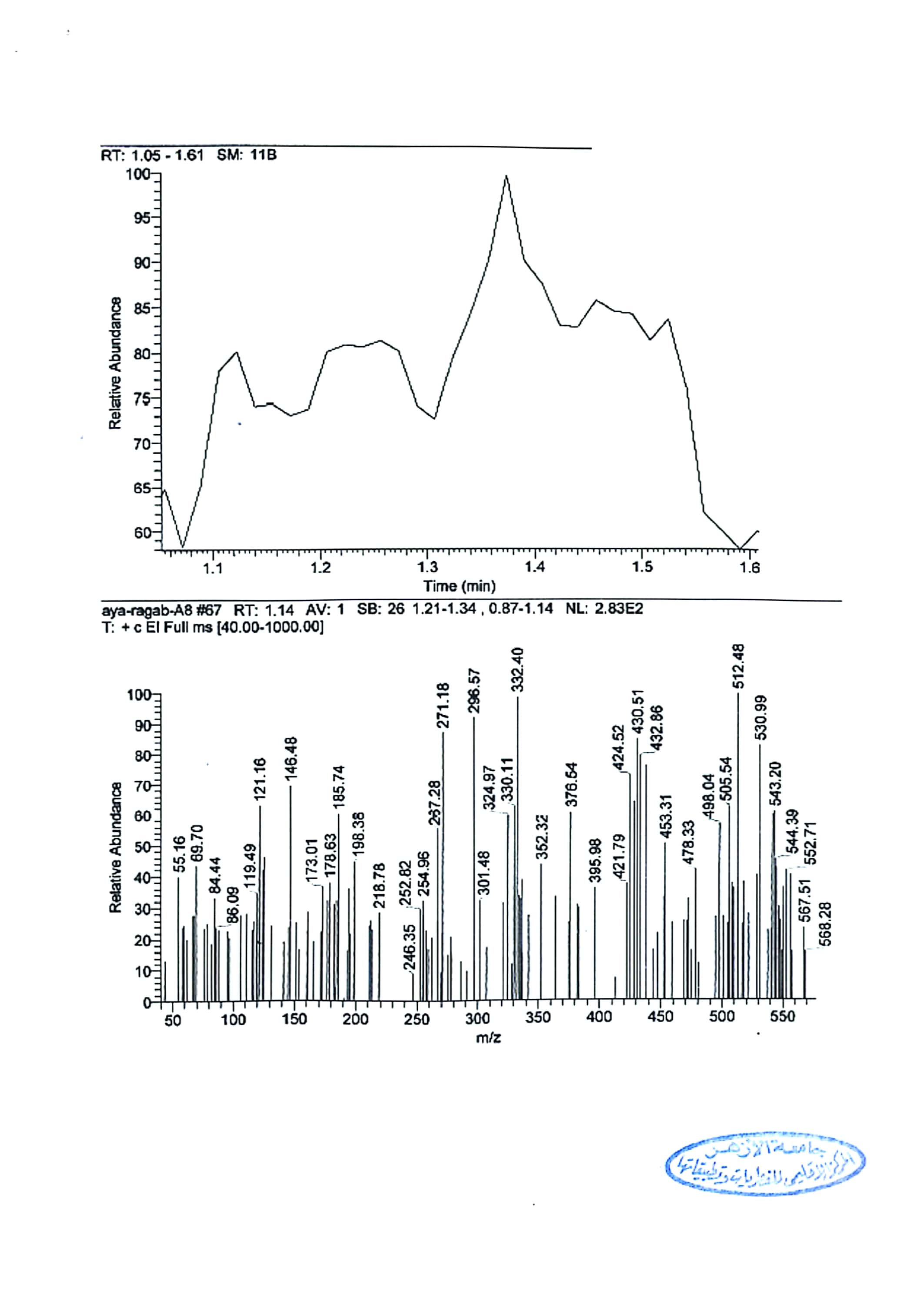
**

**Figure S89**. **IR spectrum of compound 10a**

**
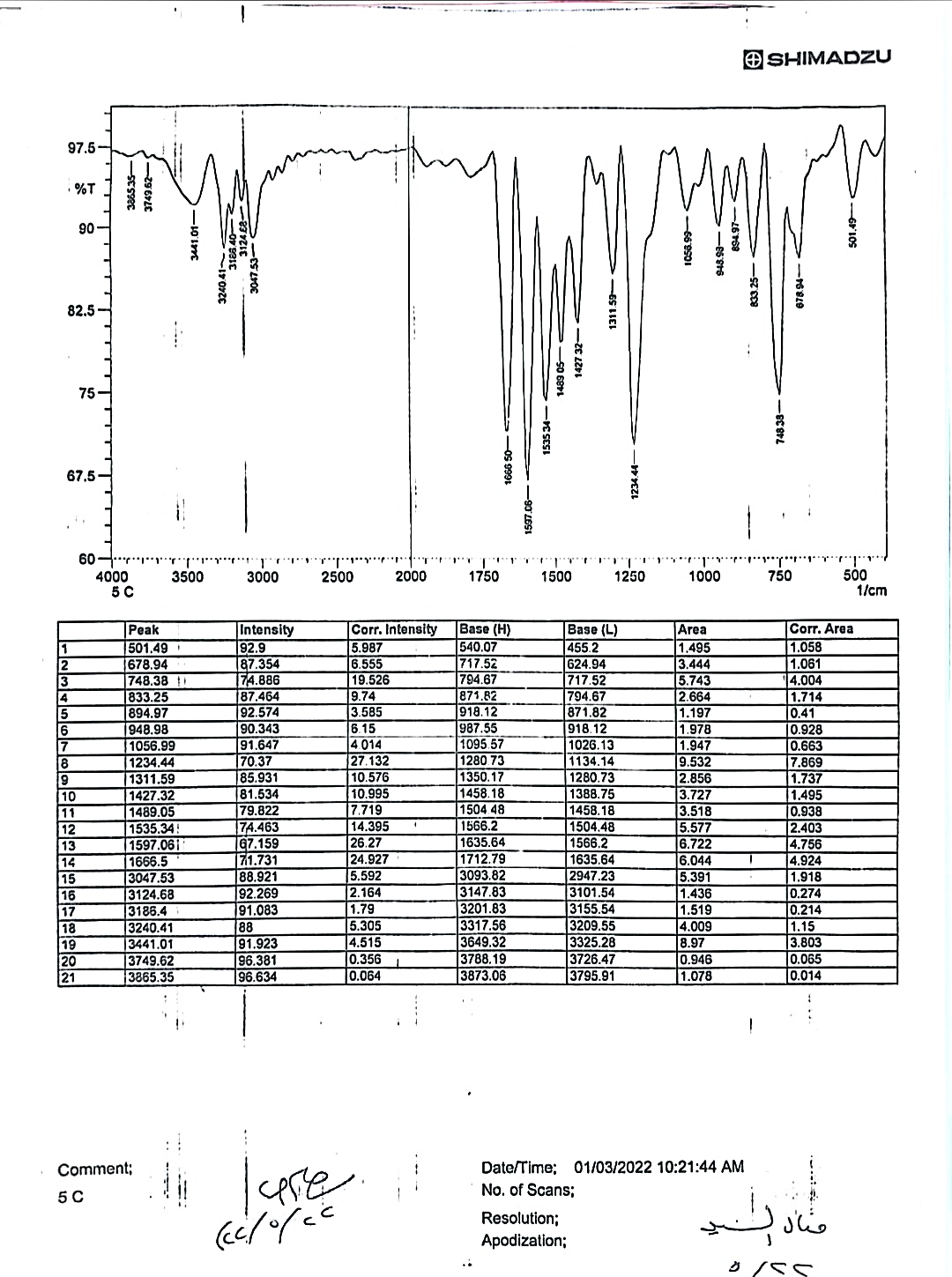
**

**Figure S90**. **IR spectrum of compound 10b**


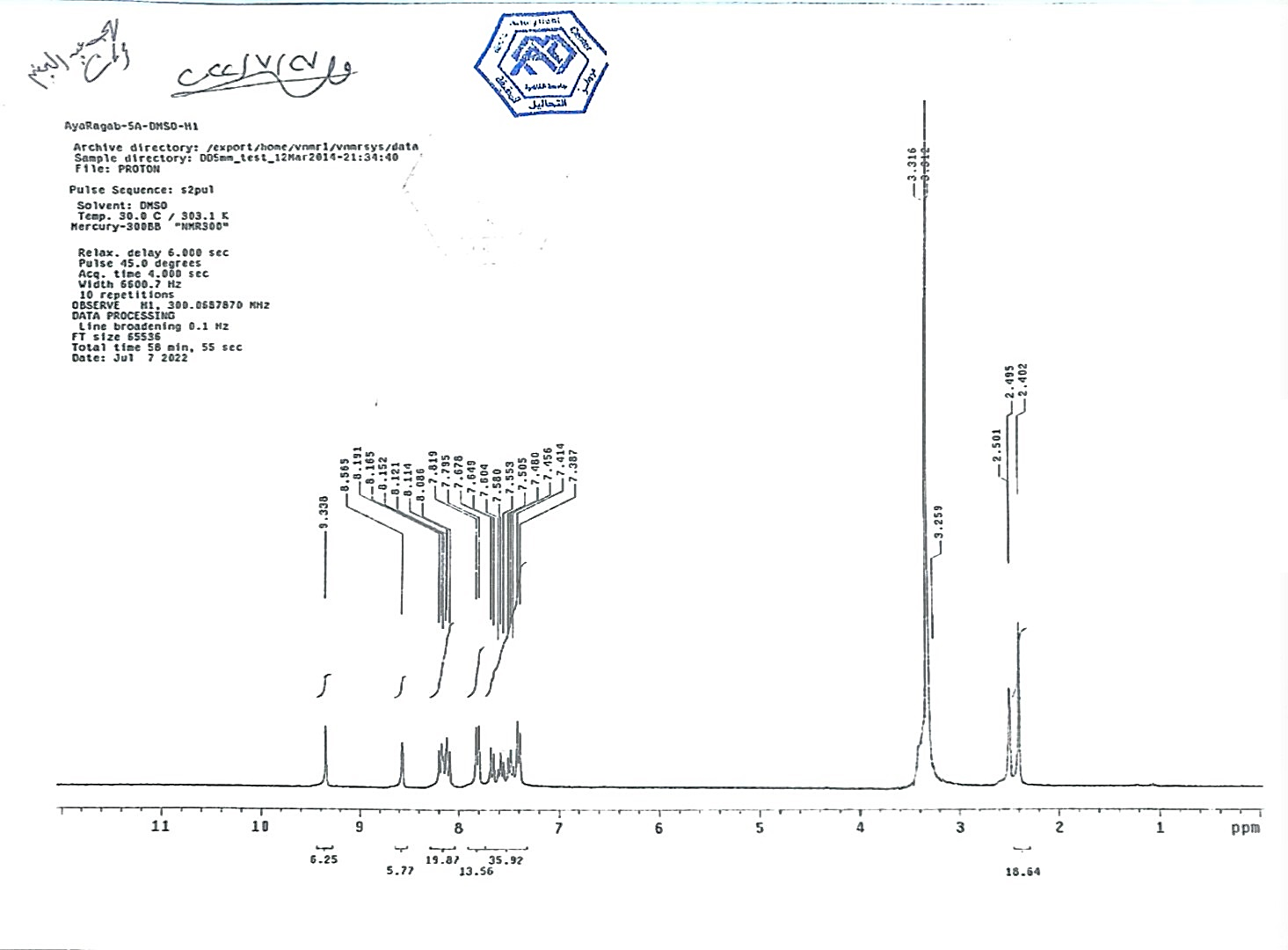


**Figure S91**. **^1^H NMR spectrum of compound 11a**

**
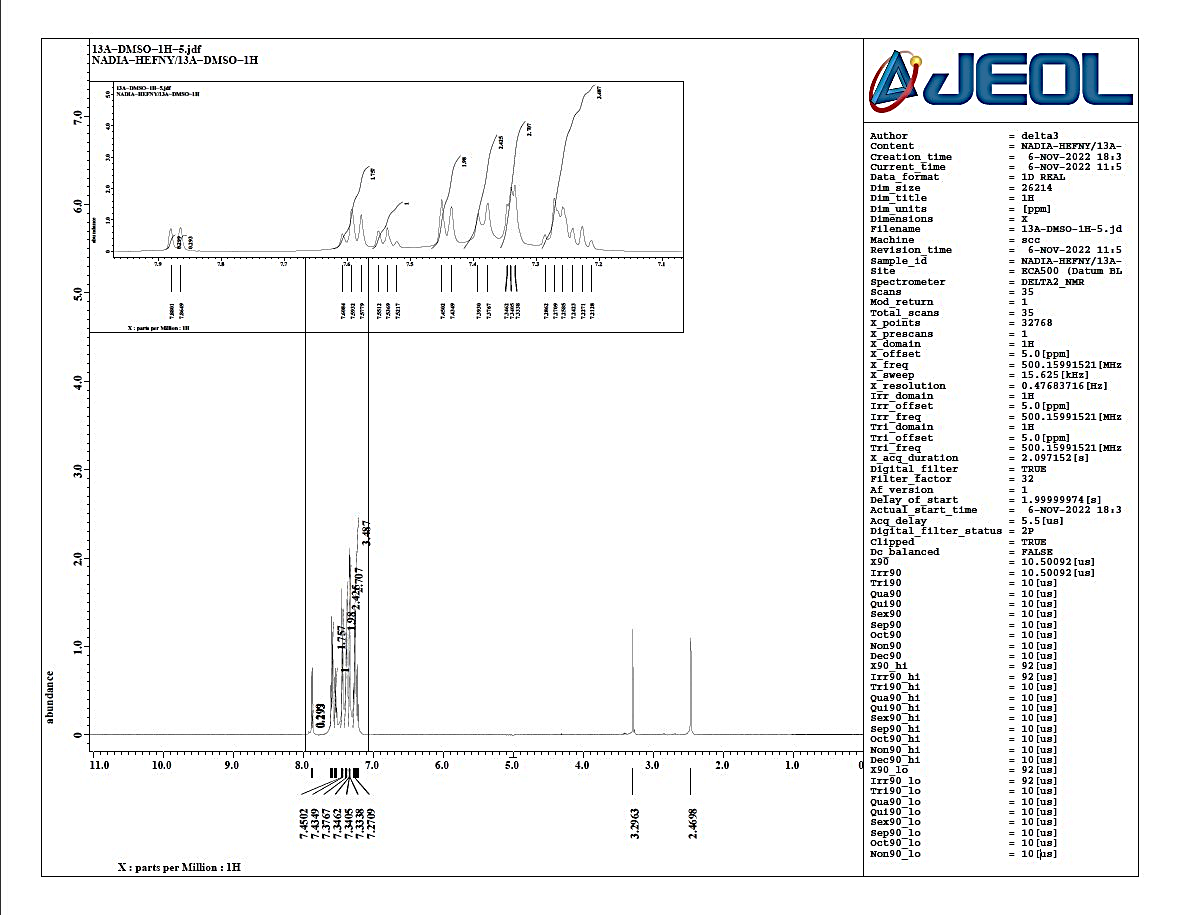
**

**Figure S92**. **Mass spectrum of compound 11a**

**
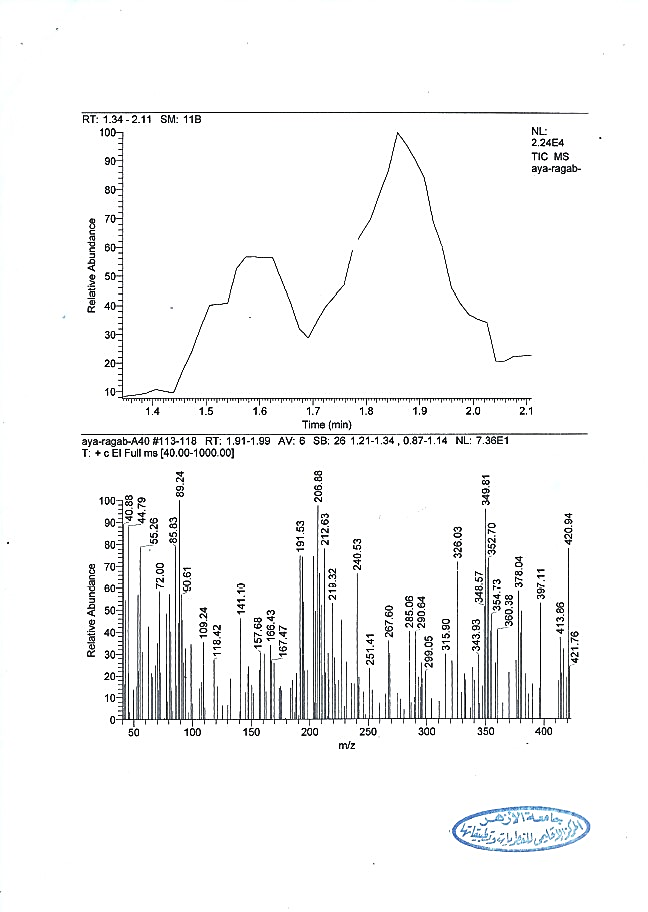
**

**Figure S93**. **^1^H NMR spectrum of compound 11c**

**
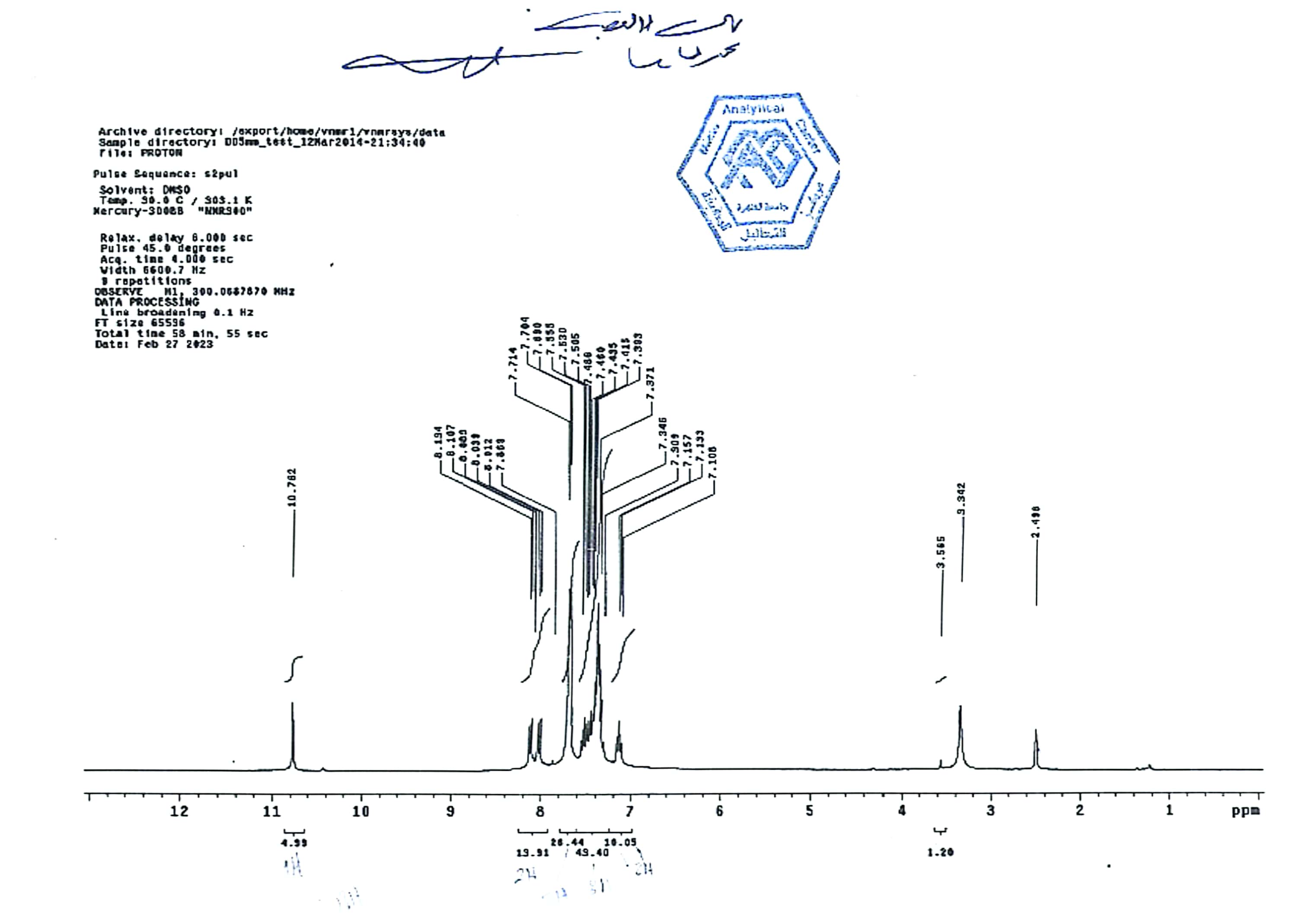
**

**Figure S94**. **^13^C NMR spectrum of compound 11c**

**
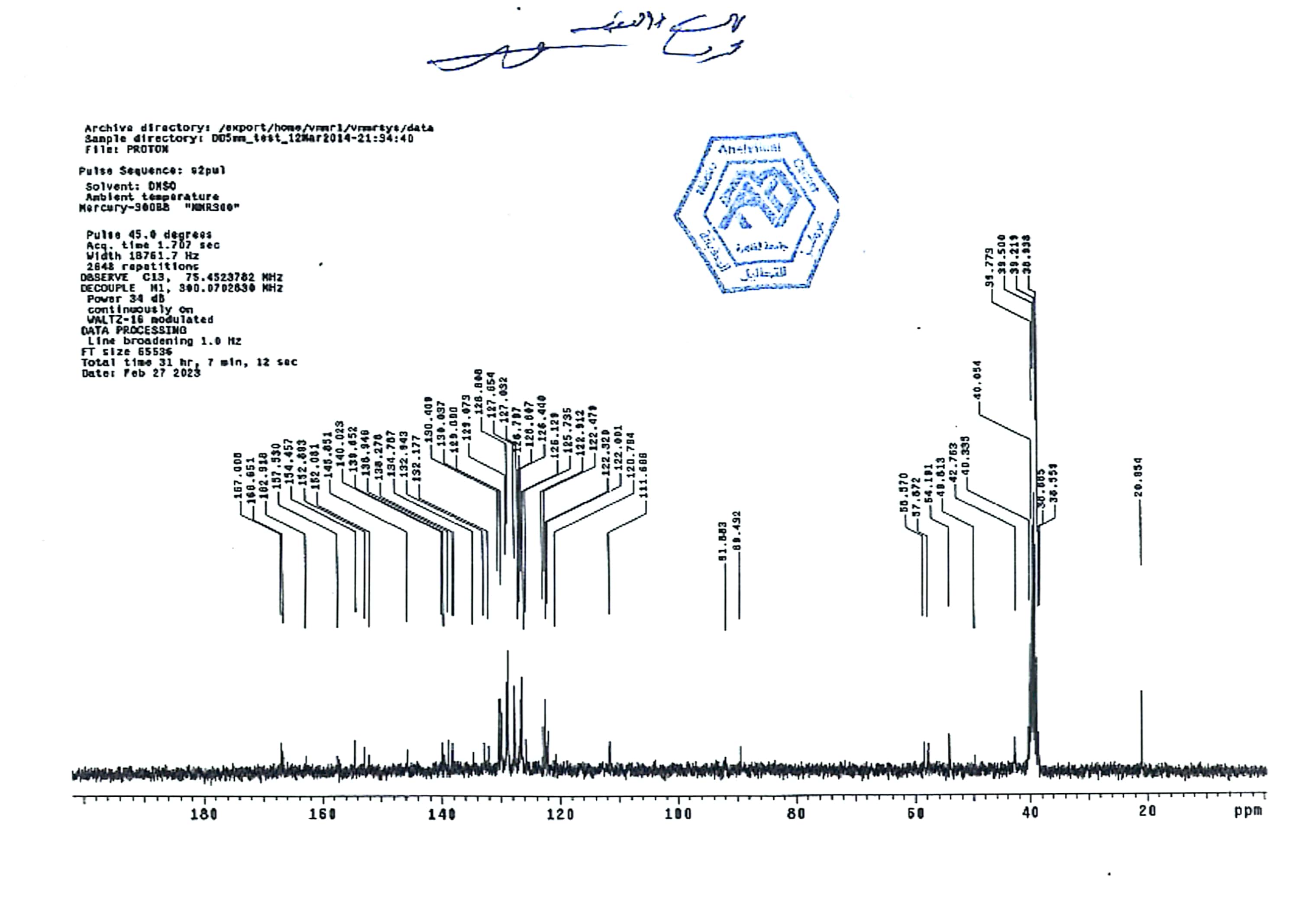
**

**Figure S95**. **IR spectrum of compound 11c**

**
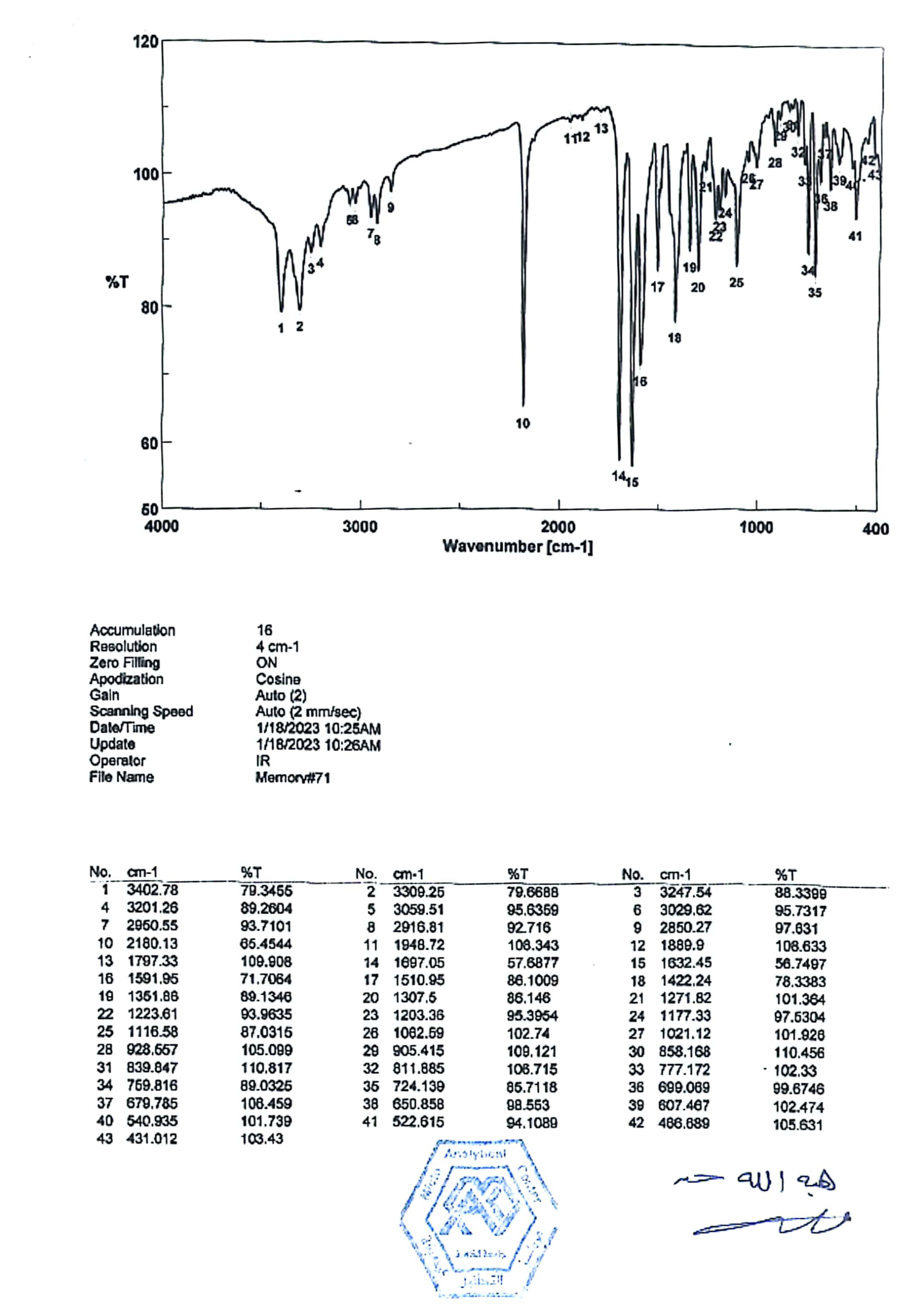
**

**Figure S96**. **IR spectrum of compound 11b**

**
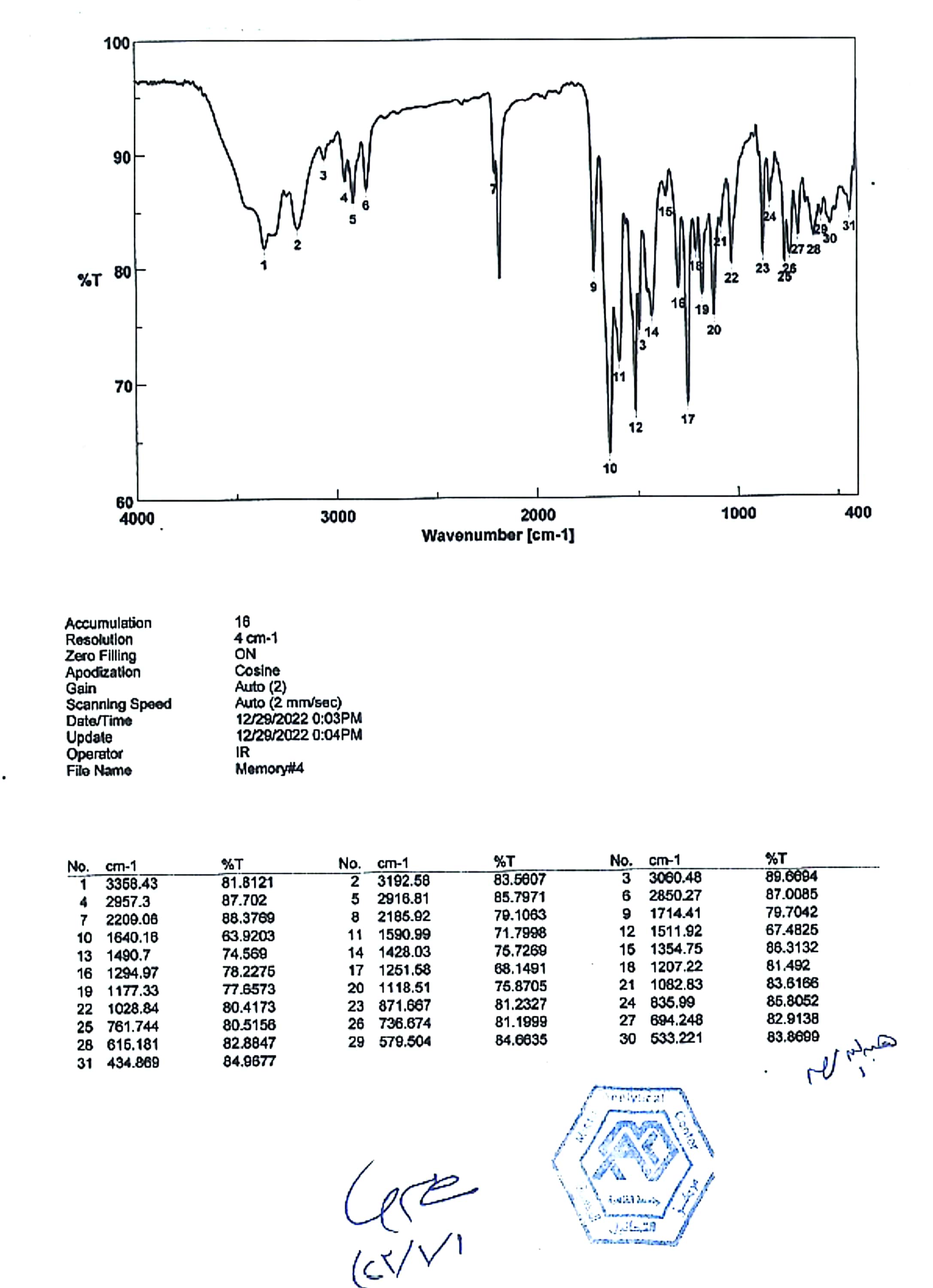
**

**Figure S97**. **^1^H NMR spectrum of compound 11d**

**
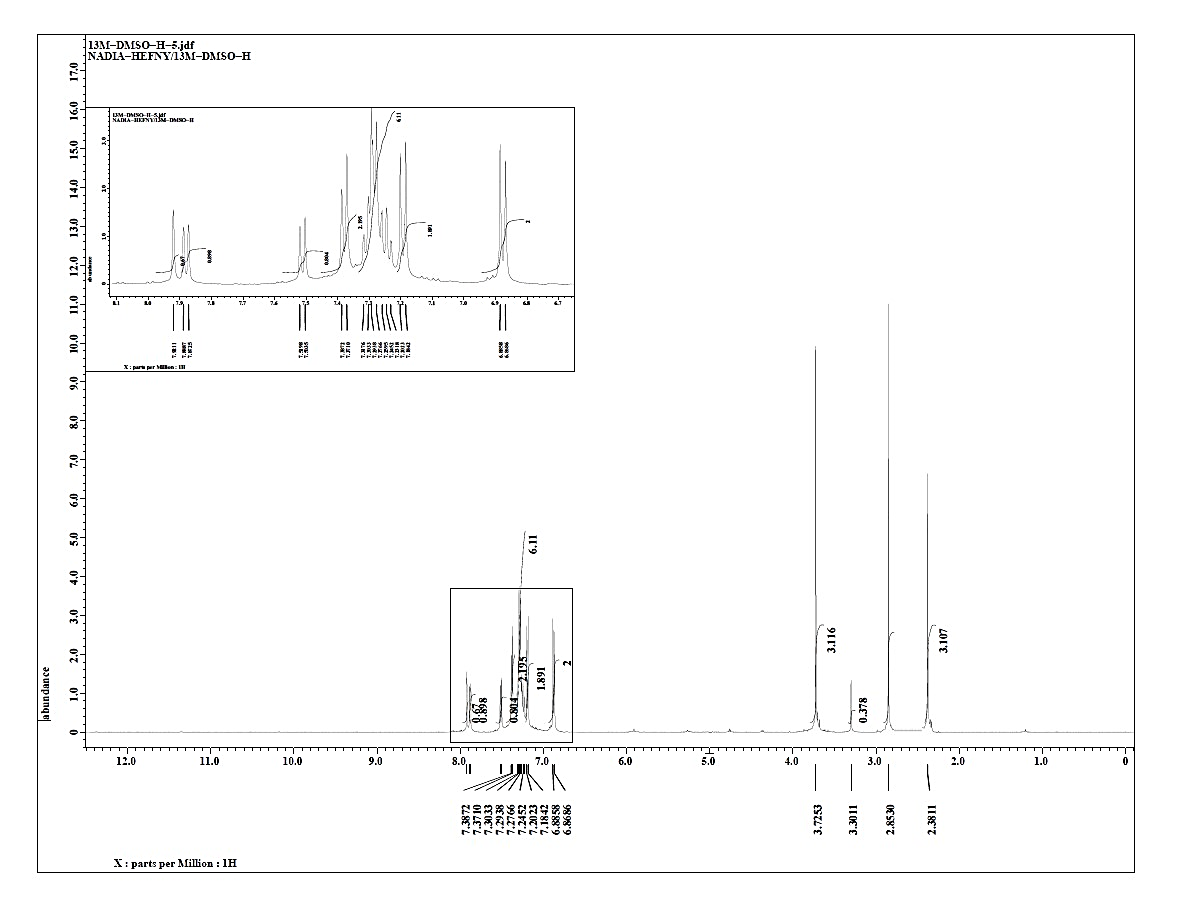
**

**Figure S98**. **IR spectrum of compound 11d**

**
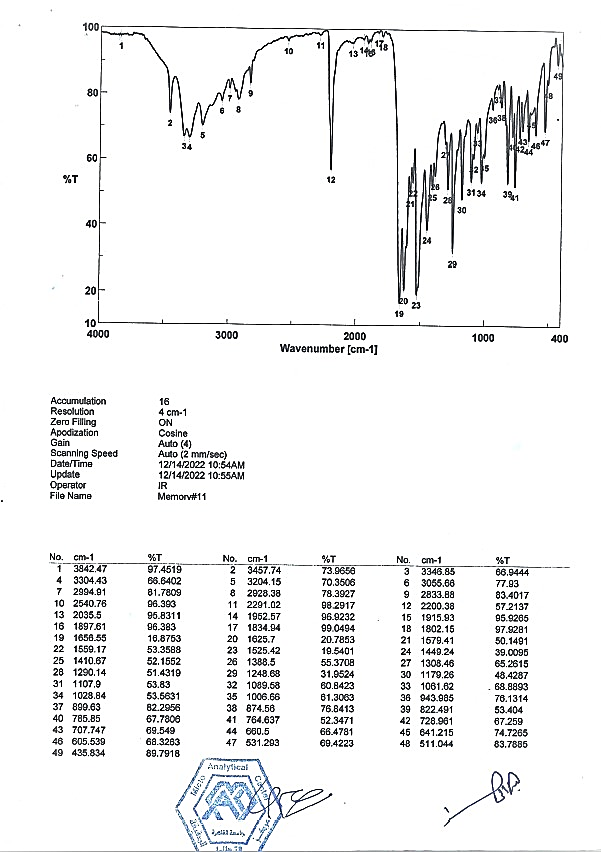
**

**Figure S99**. **^1^H NMR spectrum of compound 11e**

**
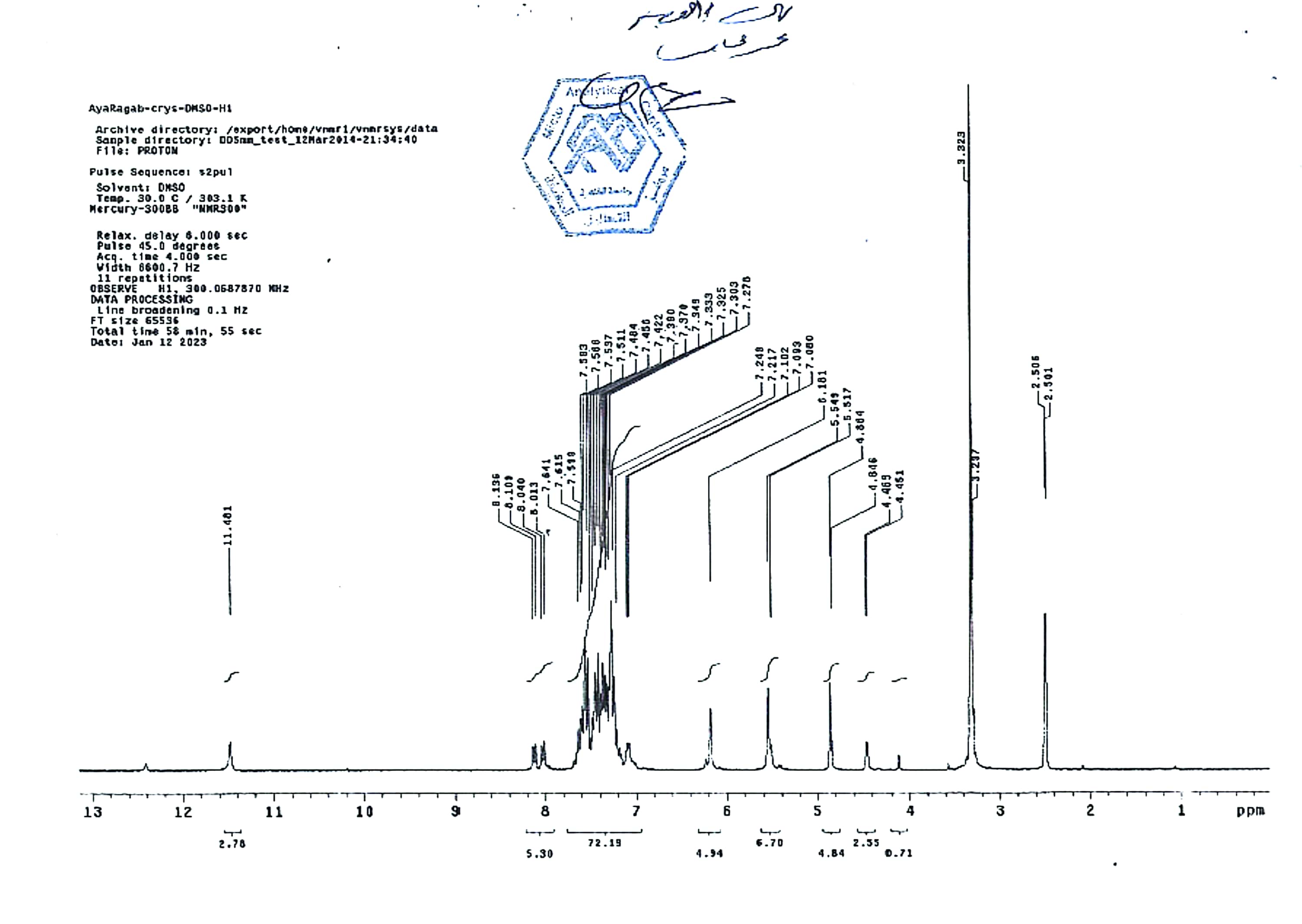
**

**Figure S100**. **^1^H NMR spectrum of compound 11e**

**
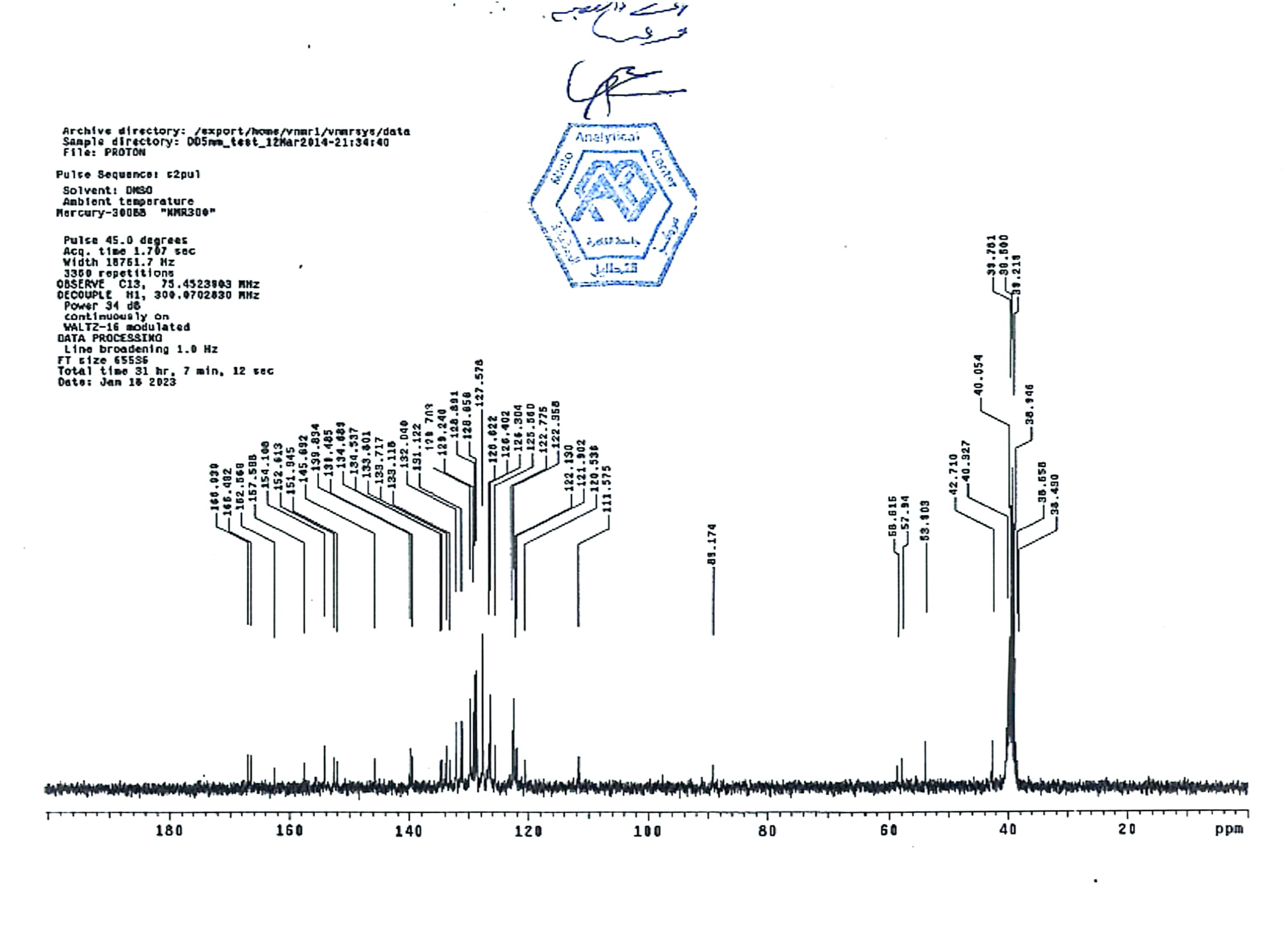
**

**Figure S101**. **IR spectrum of compound 11e**

**Figure S102**. **IR spectrum of compound 11f**

**Figure S103**. **Mass spectrum of compound 11e**
